# Effectiveness of Natural Antioxidants against SARS-CoV-2? Insights from the *In-Silico* World

**DOI:** 10.3390/antibiotics10081011

**Published:** 2021-08-20

**Authors:** Muhammad Fayyaz ur Rehman, Shahzaib Akhter, Aima Iram Batool, Zeliha Selamoglu, Mustafa Sevindik, Rida Eman, Muhammad Mustaqeem, Muhammad Safwan Akram, Fariha Kanwal, Changrui Lu, Mehwish Aslam

**Affiliations:** 1 Department of Chemistry, Chemical Engineering and Biotechnology, Donghua University, Shanghai 201620, China; 2 Institute of Chemistry, University of Sargodha, Sargodha 41600, Pakistan; shahzaibakhter740@gmail.com (S.A.); ridaykhanuos@gmail.com (R.E.); 3 Department of Zoology, University of Sargodha, Sargodha 41600, Pakistan; aima.iram@uos.edu.pk; 4 Department of Medical Biology, Faculty of Medicine, Nigde Omer Halisdemir University, Nigde 51240, Turkey; zselamoglu@ohu.edu.tr; 5 Department of Food Processing, Bahçe Vocational School, Osmaniye Korkut Ata University, Osmaniye 80000, Turkey; sevindik27@gmail.com; 6 Department of Chemistry, University of Sargodha, Bhakkar Campus, Bhakkar 30000, Pakistan; muhammad.mustaqeem@uos.edu.pk; 7 School of Health and Life Sciences, Teesside University, Middlesbrough TS1 3BA, UK; safwan.Akram@tees.ac.uk; 8 National Horizons Centre, Teesside University, Darlington DL1 1HG, UK; 9 Med-X Research Institute, School of Biomedical Engineering, Shanghai Jiao Tong University, Shanghai 201620, China; farihakaanwal@gmail.com; 10 School of Biological Sciences, University of the Punjab, Lahore 54600, Pakistan

**Keywords:** SARS CoV-2, Glycyrrhizin, Baicalin, Hesperidin, natural antivirals, antioxidants for COVID-19, inverse docking, human blood proteins

## Abstract

The SARS CoV-2 pandemic has affected millions of people around the globe. Despite many efforts to find some effective medicines against SARS CoV-2, no established therapeutics are available yet. The use of phytochemicals as antiviral agents provides hope against the proliferation of SARS-CoV-2. Several natural compounds were analyzed by virtual screening against six SARS CoV-2 protein targets using molecular docking simulations in the present study. More than a hundred plant-derived secondary metabolites have been docked, including alkaloids, flavonoids, coumarins, and steroids. SARS CoV-2 protein targets include Main protease (M^Pro^), Papain-like protease (PL^pro^), RNA-dependent RNA polymerase (RdRp), Spike glycoprotein (S), Helicase (Nsp13), and E-Channel protein. Phytochemicals were evaluated by molecular docking, and MD simulations were performed using the YASARA structure using a modified genetic algorithm and AMBER03 force field. Binding energies and dissociation constants allowed the identification of potentially active compounds. Ligand-protein interactions provide an insight into the mechanism and potential of identified compounds. Glycyrrhizin and its metabolite 18-β-glycyrrhetinic acid have shown a strong binding affinity for M^Pro^, helicase, RdRp, spike, and E-channel proteins, while a flavonoid Baicalin also strongly binds against PL^pro^ and RdRp. The use of identified phytochemicals may help to speed up the drug development and provide natural protection against SARS-CoV-2.

## 1. Introduction

SARS-CoV-2 (Severe Acute Respiratory Syndrome Coronavirus 2) originated in the Wuhan province of Central China in December 2019 [1] and its disease, COVID-19, was declared as a pandemic on 11 March 2020, after the infection spread globally [2]. This disease has affected more than 204 million people and claimed more than 4.31 million lives around the globe [3]. Thanks to vaccines, the recovery rate from COVID-19 is getting better from 70 to 90%, but the battle between the virus and humans is continued. Unfortunately, despite all efforts and worldwide scientific contributions, no specific medicines are available. The SARS-CoV-2 variants have worsened the situation, especially in countries like India, USA, and Brazil, where COVID-19 infections have crossed 77 million [3]. The rise of the SARS-CoV-2 delta variant requires better disease management as vaccinated people, including adults and children, seem to be susceptible to infection. Though the mass vaccination in the last months has decreased the severity of the disease [4], there is still a need for effective and broadly accessible remedies. Global vaccination and the emergence of a COVID-19 free world may take years, especially in the least developed countries. Moreover, new SARS-CoV-2 variants may affect the vaccination process by reducing the efficacy and efficiency of the developed vaccines. Furthermore, neutralizing/spike antibodies responses from vaccines are not sufficient to protect the people with health complications/comorbidities from COVID-19 [5]. Herbal medicines and natural therapeutics consisting of phytochemical extracts provide safe interventions to treat various viral infections using novel mechanisms. Secondary metabolites from plants and mushrooms have shown different biological effects including antioxidants, antimicrobials, and anticancer activities [6,7,8]. Natural antivirals with the least toxicity are the best choice against coronaviruses [2,9,10,11]. Many of these phytochemicals not only provide antiviral activity but also act as antioxidants to tame interleukin storm and ROS damage during COVID-19.

SARS-CoV-2 is a beta coronavirus that uses its glycosylated surface spike (S) protein to interact with the angiotensin-converting enzyme II (ACE2) receptors present in the host cells. SARS-CoV-2 attacks the lower respiratory tract to cause viral pneumonia. It can infect the gastrointestinal tract, heart, kidney, liver, and central nervous system, leading to multiple organ failure [2]. The common symptoms of virus infection include high fever, dry cough, muscle pain, fatigue, diarrhea, and shortness of breath, while the complications of COVID-19 include Acute respiratory distress syndrome (ARDS), sepsis, kidney failure, and cardiac injury [12,13,14]. The federal drug authority (FDA) takes an average of 12 years to approve a drug or vaccine for any disease [15]. Therefore, a number of antivirals, antibiotics, and other FDA-approved drugs have been repurposed to treat COVID-19 [16]. A combination of an anti-arthritis drug, Hydroxychloroquine, and an antibiotic, azithromycin, was reported as a treatment of choice for COVID-19 due to their additional antiviral properties against many viruses, including Ebola, HIV, and SARS [17,18,19,20,21,22,23,24].

**Figure 1:**
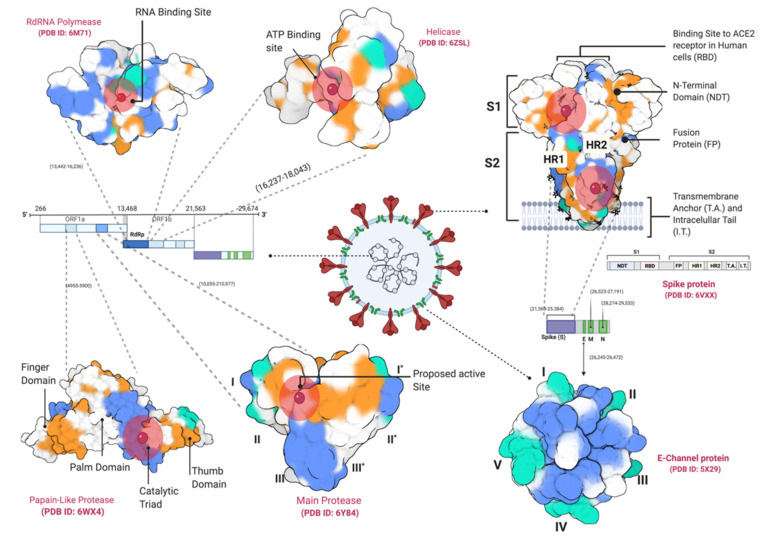
SARS-CoV-2 target proteins including Main Protease (M^Pro^, * shows domains in the second subunit of the enzyme), Papain-like Protease (PL^Pro^), RNA-dependent RNA polymerase (RdRNA polymerase), Spike glycoprotein (S), helicase, and E-Channel protein.

Later, the same drugs were found to increase the mortality rate in the COVID-19 patients [25,26] by causing arrhythmia and heart failure [27,28]. Monoclonal antibodies like Tocilizumab and Sarilumab [29,30,31], convalescent blood plasma [32,33], Camostat mesylate [34], β-interferon [35], Corticosteroids [36] have also been used against COVID-19 patients, but these treatments are costly as well as coming with long term side effects. Recently, a non-FDA-approved antiviral drug, Remdesivir, has been allowed for emergency use against COVID-19, but the drug is costly and still requires large non-randomized trials for safe use [37]. Most of these therapeutics target SAS-CoV-2 key proteins, including main protease (M^Pro^), Papain-like protease (PL^Pro^), RNA-dependent RNA polymerase (RdRNA polymerase), Spike glycoprotein (S), and helicase (Figure 1). In silico studies provide a quicker method to screen large libraries of those naturally existing in a few hours to days. In this study, we have screened more than a hundred natural compounds including Phenolics, Flavonoids, Saponins, Steroids, against six different proteins of SARS-CoV-2, with an aim to identify potential bioactivity, using molecular docking and molecular dynamic simulations (MDS).

## 2. Results

### 2.1. Main Protease (M^Pro^)

**Figure 2:**
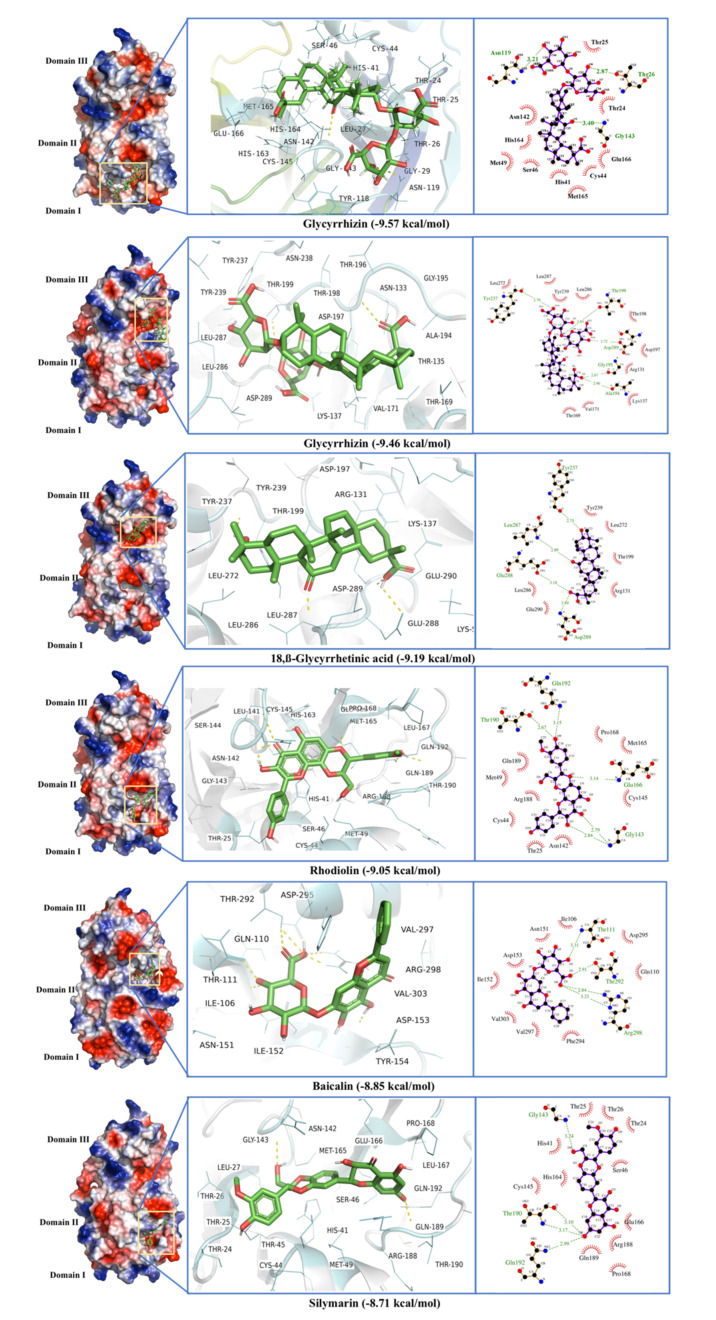
Molecular docking of Glycyrrhizin, 18,β-Glycyrrhetinic acid, Rhodiolin, Baicalin, and Silymarin with SARS-CoV-2 main protease (MPro).

The phytochemical ligands in this investigation were screened against M^Pro^. The ligands with the best docking against the Main Protease are listed in Table 1. Glycyrrhizin, 18,β-Glycyrrhetinic acid, Rhodiolin, Baicalin, and Silymarin were the best five ligands with high binding energies and dissociation constants (Figure 2). Glycyrrhizin showed binding to M^Pro^ at two different binding sites with the highest binding scores of −9.57 and −9.46 kcal·mol^−1^ and a dissociation constant of 0.11 and 0.76 µM. The best Glycyrrhizin binding site is the conventional M^Pro^ active site located between domains I and II. This site mainly consists of Thr^24^, Thr^25^, Thr^26^, Leu^27^, His^41^, Cys^45^, Ser^46^, Met^49^, Tyr1^18^, Asn^119^, Asn^142^, Gly^143^, Cys^145^, His^163^, His^164^, Met^165^, Gly^166^, and Gln^189^, where Thr^26^ and Gly^143^ formed H-bonds with the Glycyrrhizin (Figure 2, Table 1). His^41^ and Cys^145^ are the key residues involved in the enzyme active site and have been previously defined in M^Pro^ active site by an X-ray crystallographic structure (PDB ID 6WQF) obtained at room temperature [38]. The His^41^ and Cys^145^ form a catalytic dyad that interacts with the bound ligand. Other amino acids in the proposed active site Ser^46^, Leu^141^, Asn^142^, Glu^166^, Pro^168^, Gln^189^, Thr^190^, and Ala^191^ [38,39] were found involved in the stabilization of the enzyme active site.

The allosteric binding sites for Glycyrrhizin were also found between domains II and III and included the residues Arg^131^, Lys^137^, Asp^197^, Thr^198^, Thr^199^, Tyr^239^, Leu^271^, Asn^274^, Gly^275^, Met^276^, Asn^277^, Gly^278^, Arg^279^, Ala^285^, Leu^286^, Leu^287^, Glu^288^, Asp^289^, and Glu^29^ where Asp^197^, Thr^199^, and Ala^285^ form H-bonds with Glycyrrhizin (Figure 2, Table 1). The Glycyrrhizin binding to this active site may disrupt the native enzyme structure and affects its activity. This distal pocket has also been reported as a promising inhibitor binding site [40,41]. This site consists of both a loop region and β-strands.

The 18,β-Glycyrrhetinic acid, a derivative of Glycyrrhizin, showed distal allosteric binding with an energy of -9.19 kcal·mol^−1^ and dissociation constant of 0.35 µM. The active residues for 18,β-Glycyrrhetinic acid include Lys^5^, Arg^131^, Lys^137^, Asp^197^, Thr^199^, Tyr^237^, Tyr^239^, Leu^272^, Leu^286^, Leu^287^, Glu^288^, Asp^289^, and Glu^290^. Among these residues, Tyr^237^, Leu^287^, Glu^288^, and Asp^289^ formed H-bonds (Figure 2, Table 1). Although 18,β-Glycyrrhetinic acid does not directly bind to the proposed site of the enzyme, it interacts with the residues which seem present at the dimer interface of the main protease [42] making the binding more interesting to explore (Figure 2).

Other ligands with prominent binding to the main protease include Rhodiolin and silymarin that target the enzyme catalytic site with binding energies of −9.05 and −8.81 kcal·mol^−1^, while the Baicalin binding site includes some of the distal site residues, including Ile^106^, Gln^110^, Thr^111^, Asn^151^, Ile^152^, Asp^153^, Thr^292^, Phe^294^, Val^297^, Arg^298^, and Val^203^ (Figure 2, Table 1). The strong binding of Baicalin to these distal amino acids may reduce the enzyme activity. The worst docking ligands include methyl tridecanoate (binding energy −4.01 kcal·mol^−1^), Margaric acid (binding energy −3.91 kcal·mol^−1^), and docosanoic acid (binding energy −3.86 kcal·mol^−1^) (Table S1).

**Figure 3:**
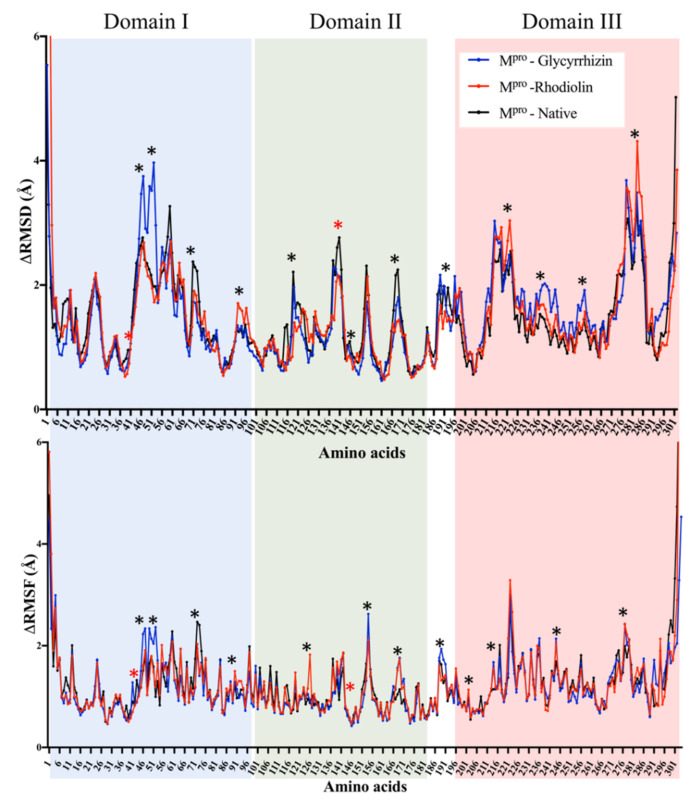
RMSD and RMSF calculated from 30 ns molecular dynamics simulation of Glycyrrhizin (blue) and Rhodiolin (red) docked with SARS-CoV-2 main protease (MPro) The residues with dynamic RMSD and RMSF are mentioned with ‘*’ while red ‘*’ shows the active site residues.

The initial MD simulation in the case of Glycyrrhizin (active site binding) shows that an equilibrium was achieved after 25 ns, so simulations were limited to 30 ns. The RMSD and RMSF values show the flexible residues in two regions, one from residues Arg^40^-Asp^56^ (Domain I) and from Ile^136^ to Asp^153^ (Domain II) (Figure 3). This shows the ligand interactions with the active site residues of the enzyme. The fluctuations between Tyr^237^ and Gly^251^ show that Glycyrrhizin binding to the active site may also induce a conformational change in other parts of the enzyme. This conformational change may be involved in reducing the enzyme activity. The Rhodiolin-M^Pro^ complex showed huge fluctuation around Ile^281^ to Val^296^ while minor fluctuations in a loop region around Val^91^ to Pro^96^ (Figure 3). The radius of gyration (R) at the end of the MD simulation shows the compactness and stability of the Glycyrrhizin- and rhodiolin-enzyme complexes (Figure S1).

### 2.2. Papain-Like Protease

**Figure 4:**
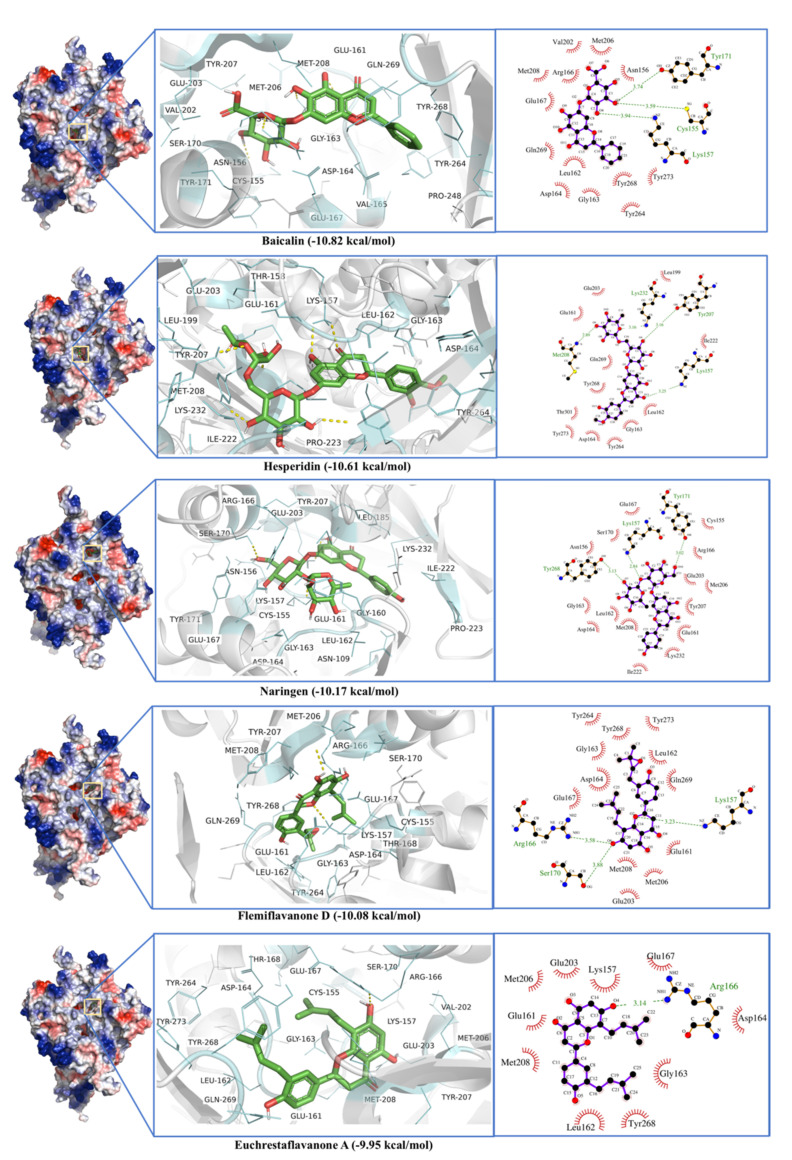
Molecular docking of Baicalin, Hesperidin, Solophenol, Naringen, Flemiflavonone D, and Euchrestaflavanone A against SARS-CoV-2 papain-like protease (PL^Pro^).

Like the M^Pro^, Papain-like Protease is involved in diverse functions that make it a potential drug target [2]. The ligands with the best docking with PL^Pro^ are listed in Table 1. Most of the ligands indirectly interacted with the active site triad Cys^111^, His^272^, and Asp^286^ [43] by binding around the enzyme active site (Figure 4). These triad residues are involved in enzyme activity [43]. Baicalin showed a strong binding with the binding energy of 10.82 kcal·mol^−1^ with a dissociation constant of 10 nM. The low dissociation constant shows strong ligand binding even at low concentrations. The active site residues include Arg^166^, Ser^170^, Val^202^, Glu^203^, Met^206^, Tyr^207^, Met^208^, Cys^155^, Asn^156^, Lys^157^, Glu^161^, Leu^162^, Gly^163^, Asp^164^, Val^165^, Glu^167^, Tyr^171^, Pro^248^, Tyr^264^, Tyr^268^, Gln^269^, and Tyr^273^ (Figure 4). Baicalin shows π-π interactions with Tyr^268^, while H-bonding is observed with Cys^155^, Ly^157^, and Tyr^171^ (Figure 4). Previously, a binding site in crystal structure of papain-like protease (PL^Pro^) (PDB ID 6WX4) was defined by the residues; Trp^106^, Asn^109^, Tyr^112^, Cys^111^, Leu^162^, Asp^164^, Val^165^, Arg^166^, Me^208^, Pro^247^, Pro^248^, Tyr^264^, Gln^269^, Cys^270^, Gly^271^, His^272^, Tyr^273^, and Thr^301^ [44]. This shows although Biacalin does not directly interact with the catalytic triad, it binds in the vicinity of the enzyme active site very strongly and impairs its proper functioning.

Hesperidin showed the second-best docking with PL^pro^ with a binding energy of −10.61 kcal.mol^−1^ and a dissociation constant of 0.02 µM. The active site residues include Arg^183^, Leu^185^, Leu^199^, Glu^203^, Met^206^, Tyr^207^, Met^208^, Gly^209^, Phe^216^, Val^220^, Gln^221^, Ile^222^, Pro^223^, Lys^232^, Lys^157^, Thr^158^, Glu^161^, Leu^162^, Gly^163^, Asp^164^, Val^165^, Glu^167^, Pro^248^, Tyr^264^, Tyr^268^, Gln^269^, Cys^270^, Tyr^273^, and Thr^301^. Among these active site residues, Lys^157^, Tyr^207^, Met^208^, and Lys^232^ formed H-bond with Hesperidin (Figure 4). Lys^157^, Arg^166^, and Tyr^268^ seem to be involved in all top five ligand-enzyme interactions.

The worst docking ligands include 6,9,12-octadecatrienoic acid (binding energy −4.85 kcal.mol^−1^), docosanoic acid (binding energy −4.64 kcal.mol^−1^), and acetohydroxamic acid (binding energy −4.13 kcal.mol^−1^). These compounds were unable to bind the proposed active site (Table S1).

**Figure 5:**
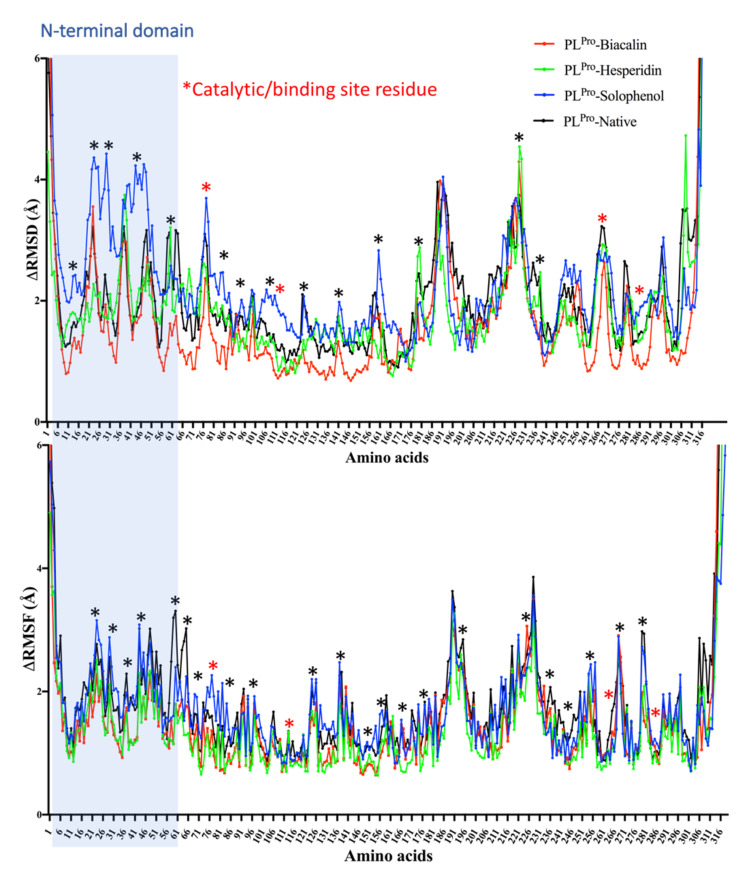
RMSD and RMSF calculated from a 30 ns molecular dynamics simulation of Baicalin (red), Hesperidin (green), and Solophenol (blue) docked with SARS-CoV-2 PL^pro^. The residues with dynamic RMSD and RMSF are mentioned with ‘*’ while red ‘*’ shows the active site residues.

MD simulations of 30 ns were run for Baicalin, Hesperidin, and Solophenol, where considerable RMSD and RMSF fluctuations were found in the active site residues of PL^Pro^ (Figure 5). In the case of Baicalin, strong binding and low dissociation constant for the Baicalin-enzyme complex is also confirmed by MD simulations. N-terminal region from Val^21^ to Pro^46^ is the most flexible region, while regions including Tyr^71^ to Asp^76^, Leu^101^ to Gln^121^, Gly^266^ to Ile^276^, and Thr^281^ to Lys^292^ seem to be involved in Biacalin-PL^pro^ interactions (Figure 5). The Hesperidin-enzyme complex Radius of gyration (R_g_) for Solophenol increased in 15–30 ns of MD simulation but Baicalin and Hesperidin, due to strong binding, show the R_g_ similar to the native enzyme (Figure S2).

### 2.3. RNA-Dependent RNA Polymerase (RdRp)

**Figure 6:**
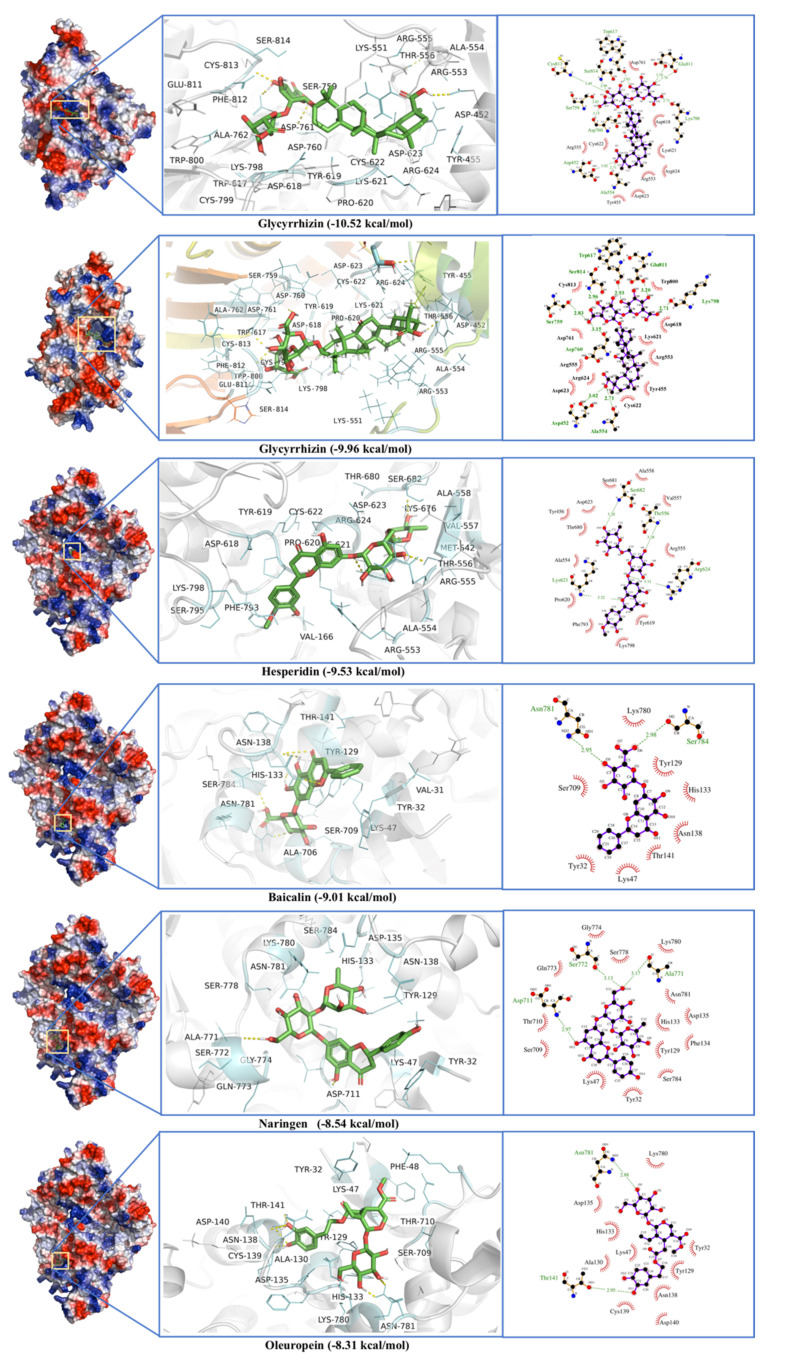
Glycyrrhizin, Hesperidin, Baicalin, Naringen, and Oleuropein docking against SARS-CoV-2 RNA-dependent RNA polymerase (RdRp).

The RNA-dependent RNA Polymerase (RdRp), the main replication enzyme for SARS-CoV-2, was screened against a library of phytochemicals (Table S1). Glycyrrhizin showed the strongest binding to RdRp. Interestingly, Glycyrrhizin strongly interacts with RdRp at two different binding sites with binding energies of −10.27 and −9.96 kcal·mol^−1^ with dissociation constants of 0.03 and 0.05 µM (Table 1, Figure 6). The key residues of the Glycyrrhizin-enzyme complex include Asp^452^, Tyr^455^, Lys^551^, Arg^553^, Ala^554^, Arg^555^, Thr^556^, Trp^617^, Asp^618^, Tyr^619^, Pro^620^, Lys ^621^, Cys^622^, Asp^623^, Arg^624^, Ser^759^, Asp^760^, Asp^761^, Ala^762^, Lys^798^, Cys^799,^ Trp^800^, Glu^811^, Phe^812^, Cys^813^ Ser^814^, where Asp^452^, Ala^554^, Trp^617^, Asp^760^, Lys^798^, Glu^811^, Cys^813^, and Ser^814^ are involved in H-binding (Figure 6). The formation of multiple H-bonds stabilizes the Glycyrrhizin-RdRp interactions.

The second binding site for Glycyrrhizin includes the residues; Ala^46^, Lys^47^, Tyr^129^, His^133^, Phe^134^, Asp^135^, Met^615^, Ser^709^, Thr^710^, Asp^711^, Phe^766^, Ala^771^, Ser ^72^, Gln^773^, Gly^774^, Ser^778^, Ile^779^, Lys^780^, Asn^781^, Ser^784^, and Thr^801^. Among these residues, Tyr^129^, His^133^, Ser^709^, Asp^711^, and Asn^781^ form H-bonds (Figure 6). The binding site of RNA-dependent RNA Polymerase (RdRp) has already been defined by using cryo-EM structures [45] where key catalytic site residues are Lys^500^, Ser^501^, Asn^507^, Lys^545^, Arg^555^, Asp^618^, Ser^759^, Asp^760^, Asp^761^, Cys^813^, Ser^814^, and Gln^815^. It seems that Glycyrrhizin interacts with these residues with a high binding affinity (multiple H-bonds) and may impair the RdRp interactions with the RNA.

Hesperidin, though not interacting with the proposed RdRp active site, binds near to the enzyme active site, where Thr^556^, Lys^621^, Arg^624^, and Ser^628^ form H-bonds and Phe^793^ shows π-π interactions. Hesperidin showed a binding energy of −9.53 kcal·mol^−1^ and with a dissociation constant of 0.1 µM. Baicalin, Naringen, and Oleuropein bind to a completely different site in RdRp, where Thr^141^, Asn^781^, and Ser^784^ are key residues (Figure 6). Baicalin showed the best binding energy of −9.01 kcal·mol^−1^ with a dissociation constant of 0.12 µM. Active site residues are Phe^35^, Asp^36^, Ile^37^, Tyr^38^, Asn^39^, Phe^48^, Leu^49^, Lys^50^, Thr^51^, Asn^52^, Val^204^, Thr^206^, Asp^208^, Asn^209^, Asp^218^, Asp^221^, Tyr^728^. Among these residues, Tyr^38^, Asn^209^, Asp^218^, and Tyr^728^ formed H-bond (Figure 6).

The worst binders include 11-eicosenoic acid (binding energy −4.11 kcal·mol^−1^), Oleic acid (binding energy −3.95 kcal·mol^−1^), and heneicosanoic acid (binding energy −3.76 kcal·mol^−1^) (Table S1).

**Figure 7:**
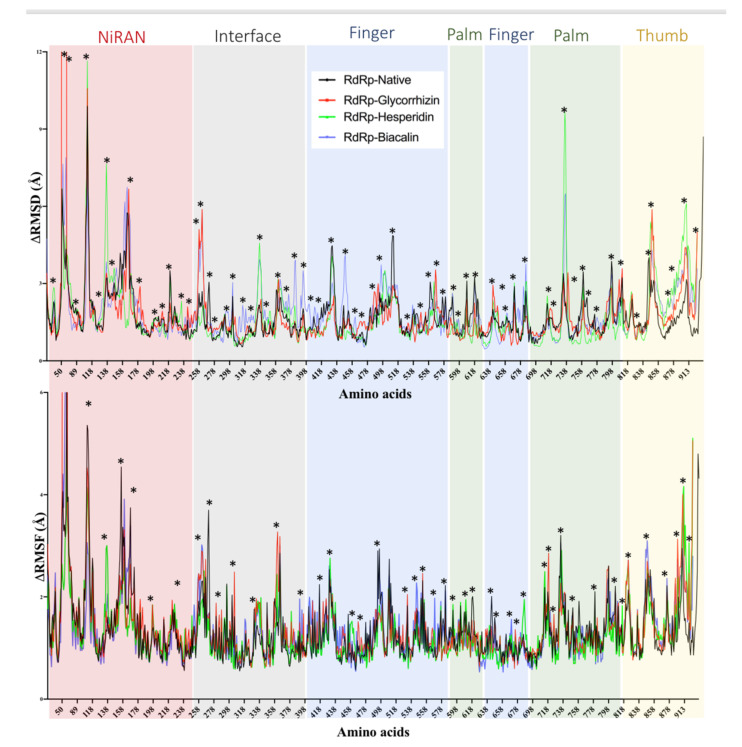
RMSD and RMSF calculated from molecular dynamics simulation of Glycyrrhizin (red), Hesperidin (green), Baicalin (blue) in complex with SARS-CoV-2 RdRp. The residues with dynamic RMSD and RMSF are mentioned with ‘*’.

A 20 ns MD simulation validates the Glycyrrhizin, Hesperidin, and baicalin interactions with RdRP. Glycyrrhizin and Baicalin show RMSD changes in the interface region consisting of residue from Val^258^ to Leu^270^, in the finger region Asp^481^ to Tyr^515^, Thr^738^ to Tyr^770^ (Figure 7). The RMSD and RMSF fluctuations seem larger in Baicalin in comparison to Glycyrrhizin and Hesperidin. In the thumb region, substantial RMSD changes were observed in all three complexes. All three ligand-enzyme complexes seem more stable in terms of potential energy than native enzymes (Figure S3).

### 2.4. Spike Glycoprotein

**Figure 8:**
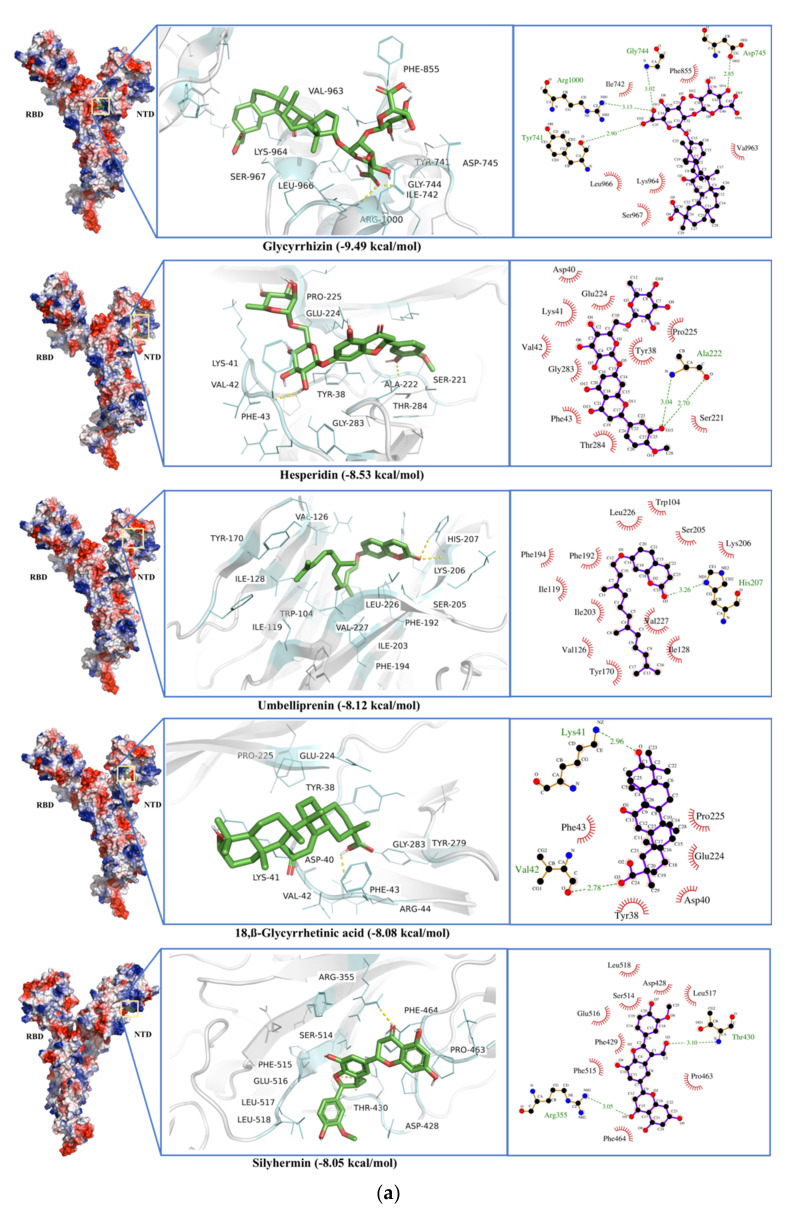

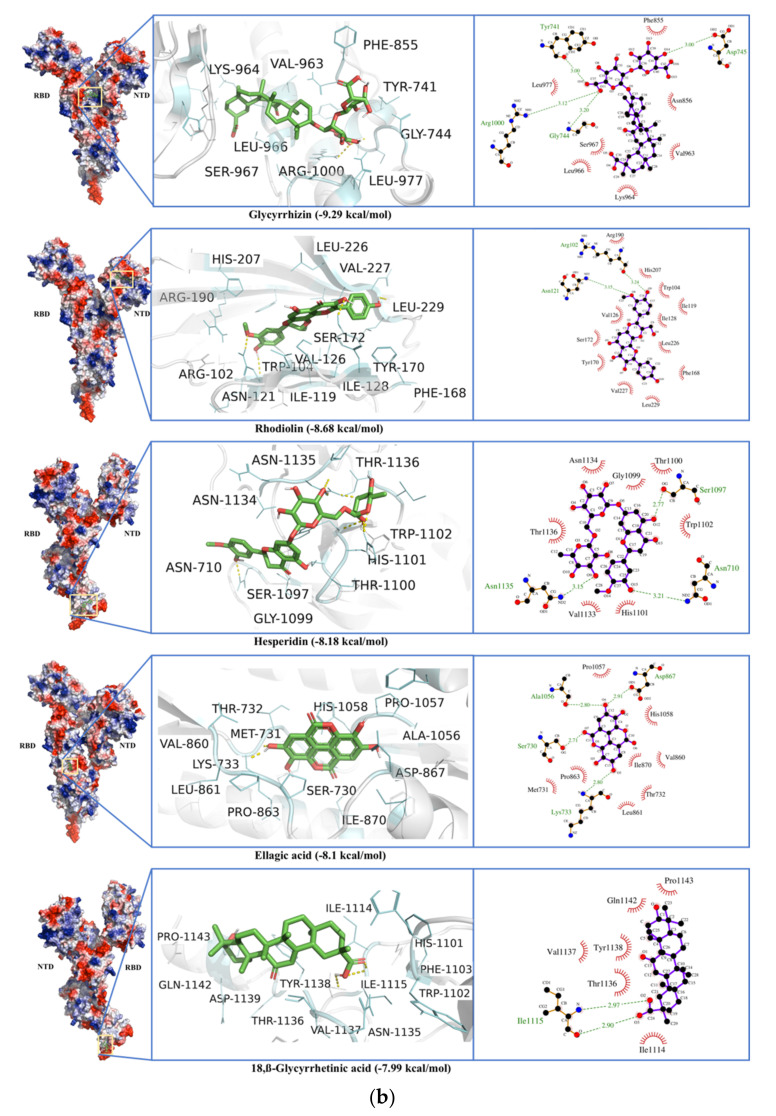
(**a**) Glycyrrhizin, Rhodiolin, Hesperidin, Ellagic acid, and 18,β-Glycyrrhetinic Acid docking to open state of the spike protein of SARS-CoV-2 Spike protein (S). (**b**) Glycyrrhizin, Hesperidin, Umbelliprenin, 18, β-Glycyrrhetinic Acid, and Silyhermin docking to the closed state of the spike protein of SARS-CoV-2 Spike protein (S).

The phytochemical ligands were screened against Spike Glycoprotein’s with both open and closed states of the protein. The ligands with the best docking for spike glycoprotein are listed in Table 1. Glycyrrhizin showed the best binding energy of −9.29 and −9.49 kcal·mol^−1^ and a dissociation constant of 0.16 and 0.11 µM for the open and close state of spike glycoproteins, respectively. The active site residues for the open state include Val^47^, His^49^, Lys^304^, Met^740^, Tyr^741^, Ile^742^, Cys^743^, Gly^744^, Asp^745^, Phe^855^, Asn^856^, Val^963^, Lys^964^, Leu^966^, Ser^967^, Ser^975^, Val^976^, Leu^977^, Asn^978^, and Arg^1000^. H-bonds and π-π interactions were observed in the Glycyrrhizin-Spike protein interactions that show strong binding of the ligand. Among these residues of the active site, Gly^744^, Asp^745^, and Arg^1000^ formed H-bonds, and Tyr^741^, Phe^855^ show π-π interactions in an open protein state (Figure 8a). Glycyrrhizin interacts with Tyr^741^, Ile^742^, Cys^743^, Gly^744^, Asp^745^, Phe^855^, Asn^856^, Val^963^, Lys^964^, Leu^966^, Ser^967^, Ser^975^, Val^976^, Leu^977^, Asn^978^, and Arg^1000^ residues in the close spike protein state, while Tyr^741^, Gly^744^, Asp^745^, and Arg^1000^ formed H-bonds in the closed state of spike glycoprotein (Figure 8b). This shows Glycyrrhizin’s interactions with the S2 subunit of the spike protein.

Rhodiolin and Hesperidin bind to the N-terminal domain of spike protein and show the second-best binding energies, i.e., −8.68 and −8.53 kcal·mol^−1^ and dissociation constants, i.e., 0.43 and 0.56 µM for open and states, respectively. Active site residues for Rhodiolin were Arg^102^, Gly^103^, Trp^104^, Ile^119^, Asn^121^, Val^126^, Ile^128^, Phe^168^, Tyr^170^, Ser^172^, Arg^190^, Phe^192^, Ile^203^, His^207^, Leu^226^, Val^227^, Asp^228^, Leu^229^, where Arg^102^ and Asn^121^ formed H-bonds (Figure 8a). Hesperidin shows interactions with residues, i.e., Tyr^38^, Asp^40^, Lys^41^, Val^42^, Phe^43^, Arg^44^, Lys^206^, Phe^220^, Ser^221^, Ala^222^, Glu^224^, Pro^225^, Leu^226^, Tyr^279^, Gly^283^, and Thr^284^, where Ala^222^ forms a H-bond (Figure 8b).

The worst docking ligands include stearic acid (binding energy −3.38 kcal·mol^−1^), oleic acid (binding energy −3.26 kcal·mol^−1^), and docosanoic acid (binding energy −3.21 kcal·mol^−1^) for open state spike glycoprotein (Table S1). The worst docking ligands include Nervonic acid (binding energy −3.92 kcal·mol^−1^), octadec-9-enyl icosanoate (binding energy −3.87 kcal·mol^−1^), and Tetracosanoic acid (binding energy −3.71 kcal·mol^−1^) for close state spike glycoprotein (Table S1).

**Figure 9:**
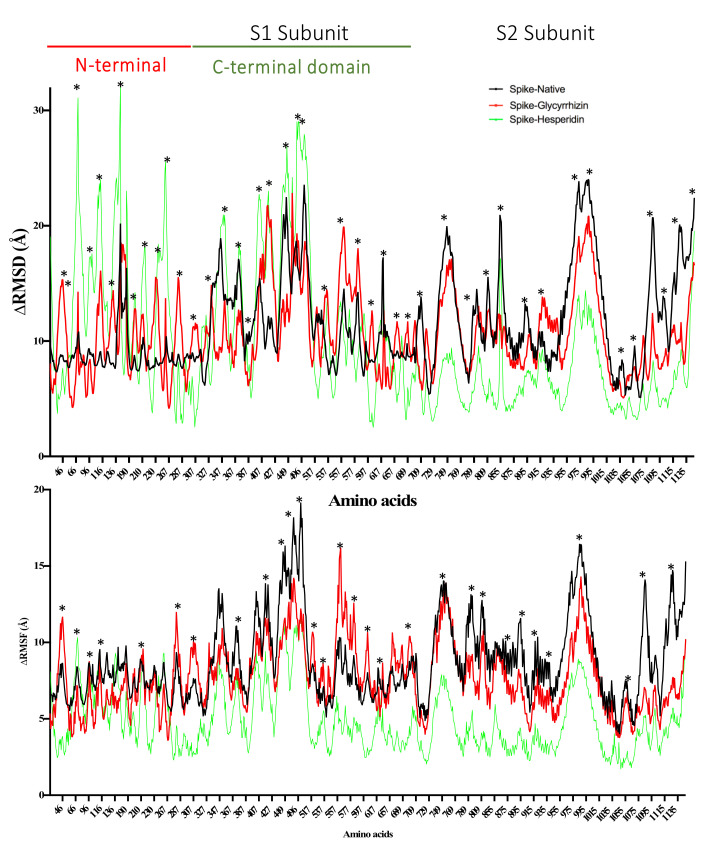
RMSD and RMSF calculated from 30 ns molecular dynamics simulation of Glycyrrhizin (red) and Hesperidin (green) in complex with SARS-CoV-2 spike protein (closed state). The residues with dynamic RMSD and RMSF are mentioned with ‘*’.

MD simulation with a closed state of spike protein shows that Glycyrrhizin-enzyme complexes are more stable than Hesperidin-enzyme complexes in terms of potential energy and R_g_ (Figure S4). RMSD and RMSF fluctuations in N-terminal domain subunit 1 validate the hesperidin interactions with the residue present in this region (Figure 9). Glycyrrhizin and Hesperidin also show large fluctuations in the S2 areas of spike protein (729–769 and 955–1035 a.a).

### 2.5. Helicase (Nsp13) Protein

**Figure 10:**
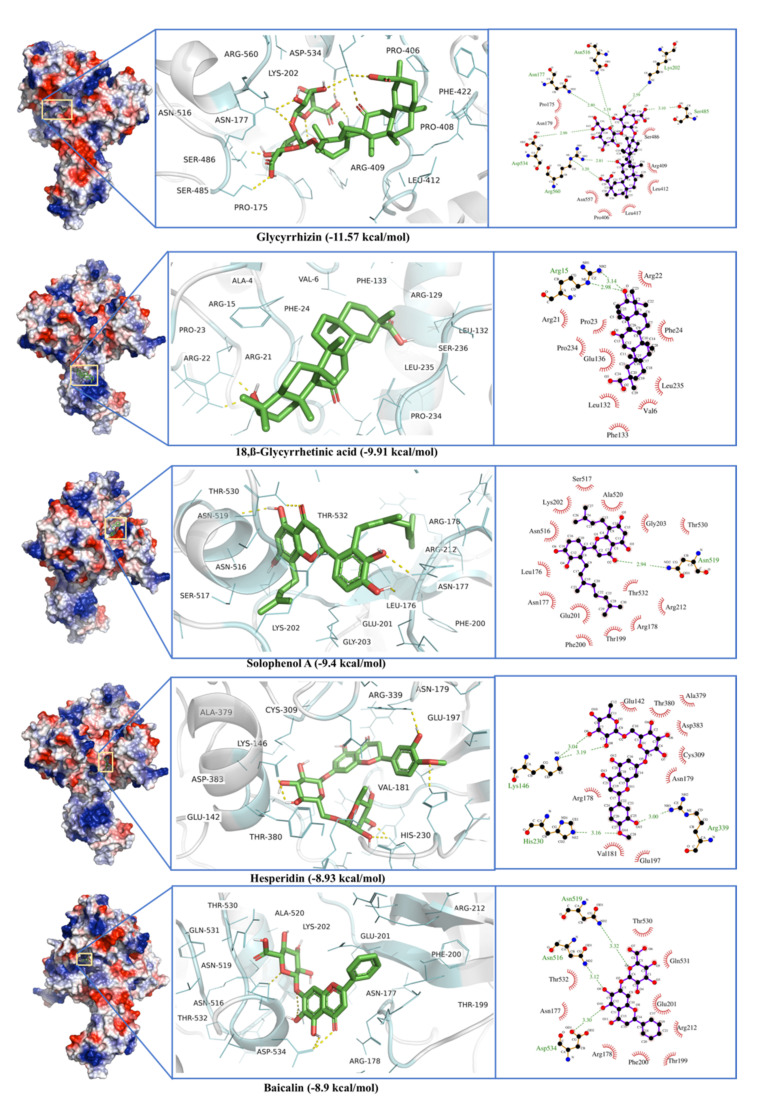
Glycyrrhizin, β-Glycyrrhetinic Acid, Solophenol A, Hesperidin, and Baicalin docking to helicase of SARS-CoV-2 Spike protein (S).

**Figure 11:**
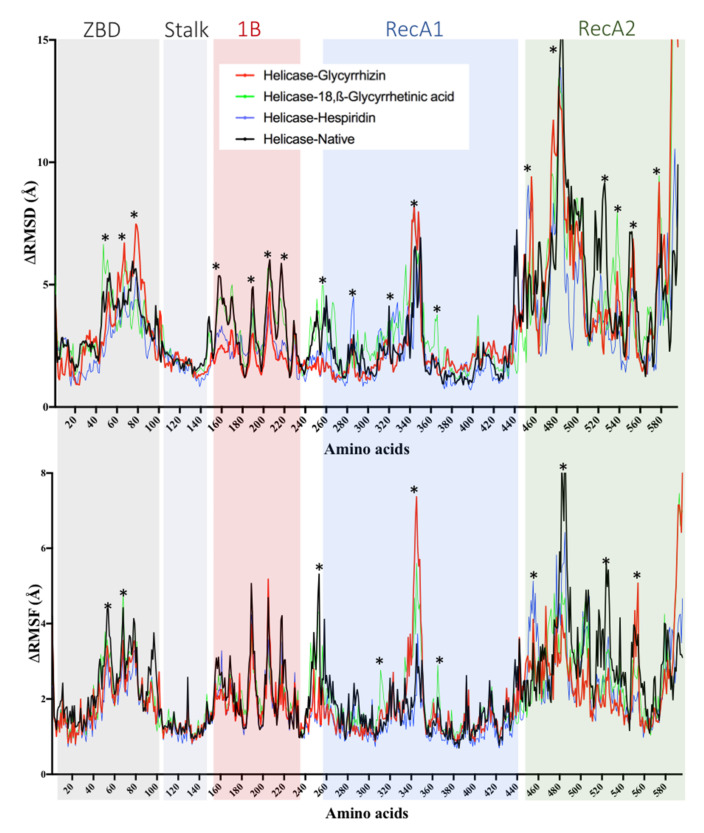
RMSD and RMSF calculated from 30 ns molecular dynamics simulation of Glycyrrhizin (red), Hesperidin (green), Baicalin (blue) in complex with SARS-CoV-2 RdRp. The residues with dynamic RMSD and RMSF are mentioned with ‘*’.

Phytochemicals ligands were docked against Helicase (Nsp13) protein and Glycyrrhizin, β-Glycyrrhetinic Acid, Solophenol A, Hesperidin, and Baicalin were found to be the best docking ligands (Table 1). Interestingly, Glycyrrhizin showed very strong interactions with the helicase with a binding energy of −11.57 kcal·mol^−1^ and a dissociation constant of 3 nM. Active site residues are Pro^175^, Leu^176^, Asn^177^, Lys^202^, Leu^405^, Pro^406^, Ala^407^, Pro^408^, Arg^409^, Leu^412^, Thr^413^, Gly^415^, Thr^416^, Leu^417^, Phe^422^, Ser^485^, Ser^486^, Pro^514^, Tyr^515^, Asn^516^, Asn^519^, Thr^532^, Val^533^, Asp^534^, His^554^, Asn^557^, Asn^559^, and Arg^560^. Among active site six residues, including Asn^177^, Lys^202^, Ser^485^, Asn^516^, Asp^534^, and Arg^560^ form H-bond (Figure 10). Allosteric binding for Glycyrrhizin was observed in a region between RecA1 and RecA2 domains. The same was found to be true in the case of MD simulation, where fluctuations in RMSF and RMSD were observed in a region between RecA1 and RecA2 (Figure 11). The Glycyrrhizin derivative, 18,β-Glycyrrhetinic acid, also showed strong interactions with the second-best binding energy of −9.91 kcal·mol^−1^ and a dissociation constant of 54 nM. The active site residues are Ala^4^, Val^6^, Arg^15^, Arg^21^, Arg^22^, Pro^23^, Phe^24^, Arg^129^, Leu^132^, Phe^133^, Glu^136^, Pro^234^, Leu^235^, and Ser^236^. Among active site residues, Arg^15^ formed two H-bond. Hesperidin shows the binding energy of −8.93 kcal·mol^−1^ binds to enzyme active site where three H-bonds were observed for Lys^146^, His^230^, and Arg^339^ (Figure 10). The worst docking ligands include octadec-9-enyl icosanoat (binding energy −4.30 kcal·mol^−1^), docosanoic acid (binding energy −4.16 kcal·mol^−1^), and acetohydroxamic acid (binding energy −4.11 kcal·mol^−1^) for open state spike glycoprotein. Some of these compounds were even unable to bind the proposed active site (Table S1).

### 2.6. E-Channel (Envelop Small Membrane Protein)

**Figure 12:**
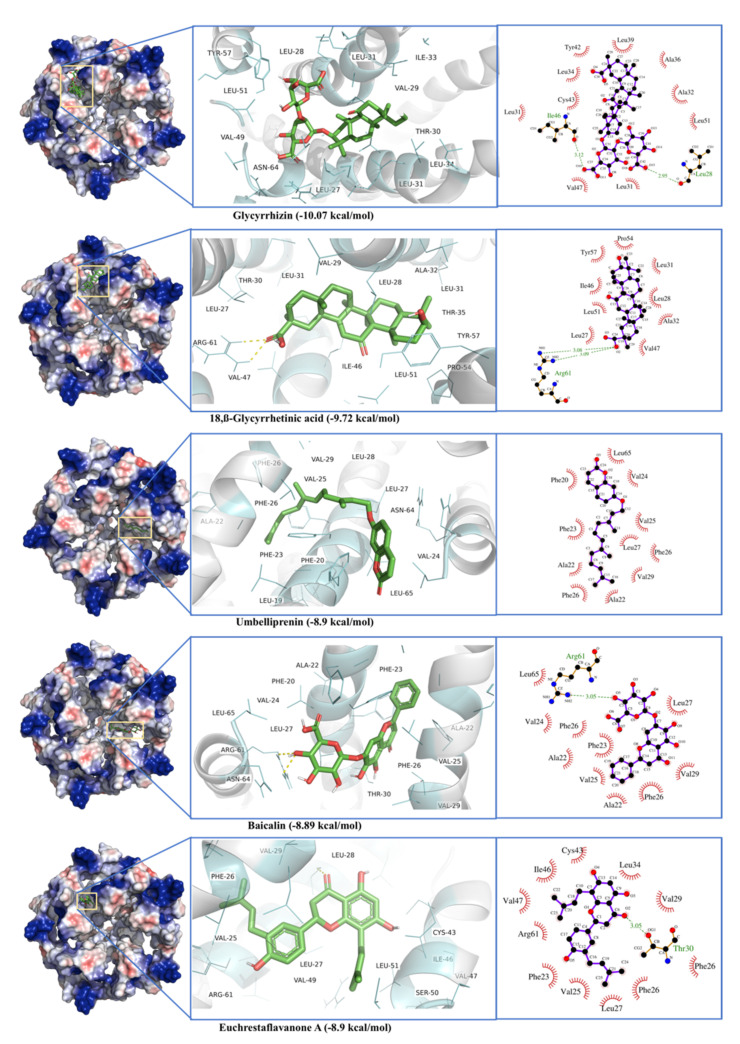
Glycyrrhizin, β-Glycyrrhetinic Acid, Solophenol A, Hesperidin, and Baicalin docking to helicase of SARS-CoV-2 Spike protein (S).

The phytochemical ligands were docked against E-channel protein are listed in Table 1. Glycyrrhizin showed the best binding score of −10.07 kcal·mol^−1^ and dissociation constant of 0.04 µM. Glycyrrhizin interacts with the active site residues present in different chains including Arg^61^, and Asn^64^ from Chain A, Leu^28^, Val^29^, Leu^31^, Ala^32^, Ile^33^, Ala^36^, and Arg^38^ from Chain D, Leu^27^, Thr^30^, Leu^31^, Leu^34^, Leu^37^, Leu^39^, Tyr^42^, Cys^43^, Ile^46^, Val^47^, Val^49^, Ser^50^, Leu^51^, Pro^54^, and Tyr^57^ from Chain E. Among these active site residues, Leu^28^ and Ile^46^ formed H-bonds (Figure 12). 18,β-Glycyrrhetinic acid showed the second-best bind energy of −9.72 kcal·mol^−1^ with a dissociation constant of 0.07 µM. Active site residues are Arg^61^ (Chain A), Leu^28^, Val^29^, Leu^31^, Ala^32^, and Thr^35^ (Chain D), Leu^27^, Thr^30^, Leu^31^, Ile^46^, Val^47^, Leu^51^, Pro^54^, and Tyr^57^ of Chain E. Among active residues, Arg^61^ formed two H-bonds (Figure 12). The worst docking ligands include malic acid (binding energy −4.05 kcal·mol^−1^), oxalic acid (binding energy −3.24 kcal·mol^−1^), and acetohydroxamic acid (binding energy −3.10 kcal·mol^−1^) for open state spike glycoprotein. Some of these compounds were even unable to bind the proposed active site (Table S1).

### 2.7. Non-Specific Interactions of Selected Ligands against Human Blood Proteins

**Figure 13:**
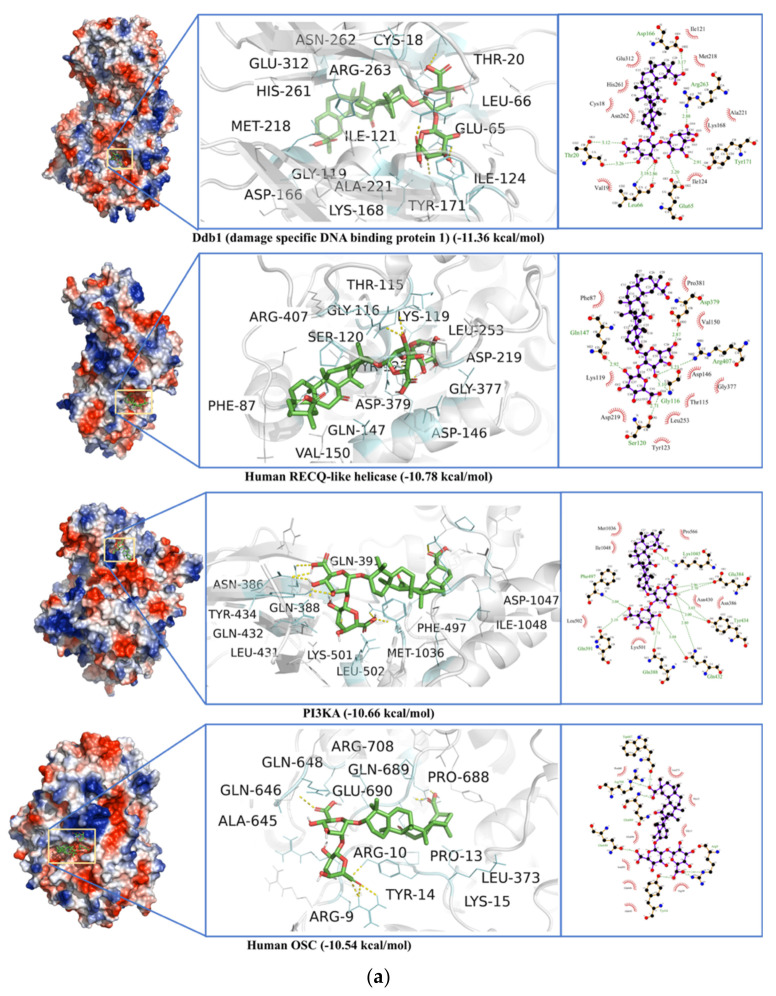

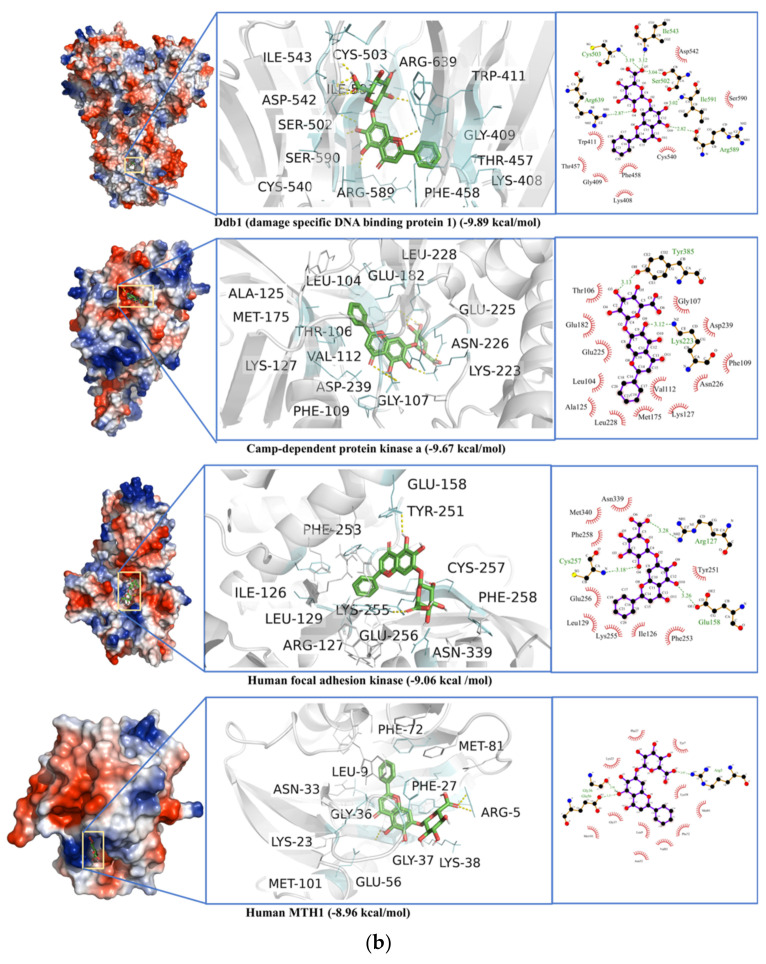

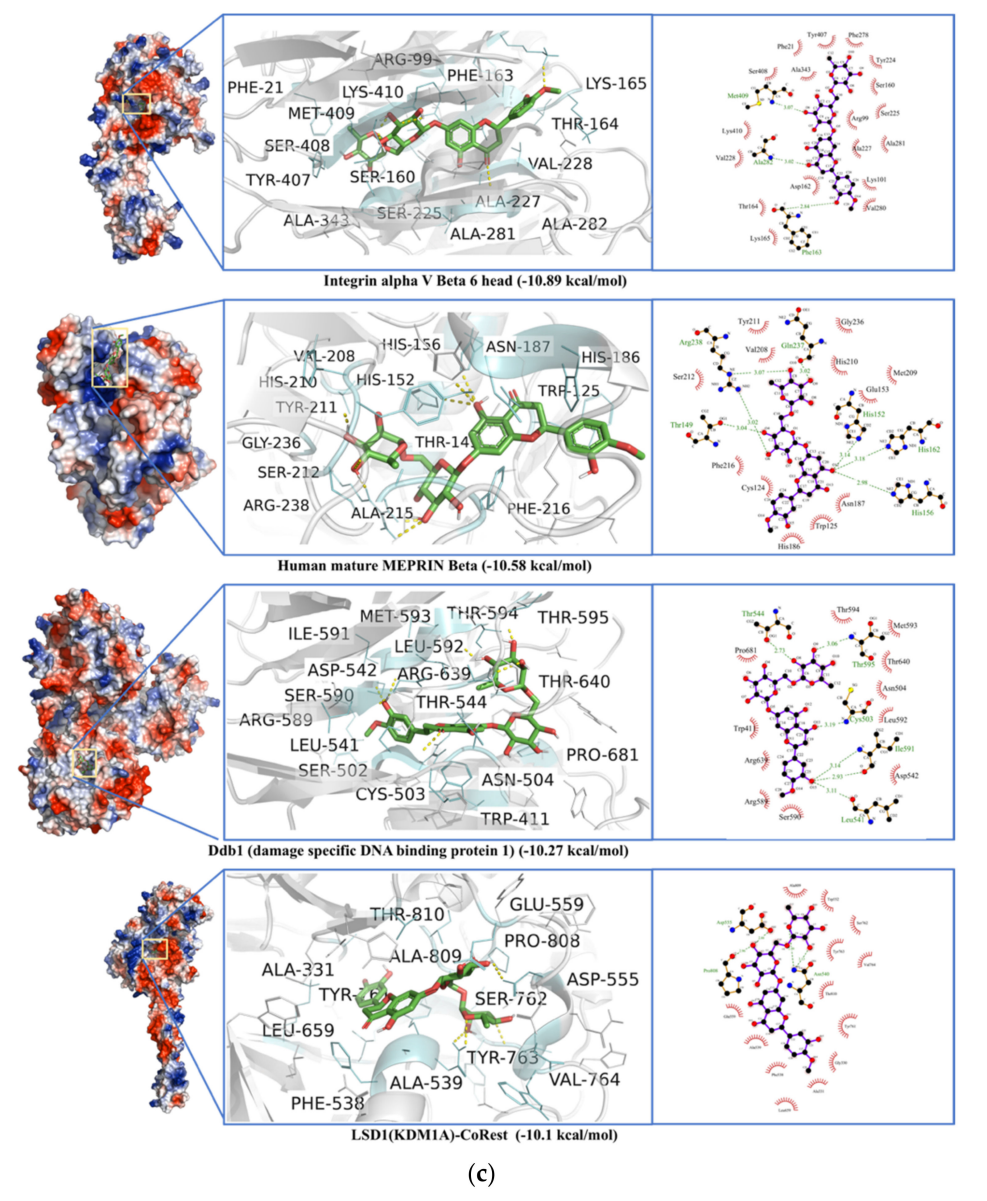
(**a**) Glycyrrhizin non-specific interactions with human proteins including Ddb1 (damage specific DNA binding protein 1), Human RECQ-like helicase, PI3KA (transferase), and Human OSC (an isomerase). (**b**) Baicalin non-specific interactions with damage specific DNA binding protein 1 (Ddb1), Camp-dependent protein kinase a (catalytic alpha subunit), Human focal adhesion kinase, and Human MTH1 (a hydrolase). (**c**) Hesperidin non-specific interactions with Integrin alpha V Beta 6 head, Human mature MEPRIN Beta (a hydrolase), Ddb1 (damage specific DNA binding protein 1), and LSD1(KDM1A)-CoRest (a transcription protein).

Glycyrrhizin, Hesperidin, and Baicalin were docked to a local library of selected human blood proteins (a total of 100 blood proteins) to map non-specific interactions of these ligands with non-specific proteins (Table S2). Glycyrrhizin showed the highest binding affinity against DdB1 (damage-specific DNA binding protein) with a binding energy of −11.36 kcal·mol^−1^ and a dissociation constant of 1.68 nM. The interactions residues included Asn^16^, Gly^17,^ Cys^18^, Val^19^, Thr^20^, Glu^65^, Leu^66^, Thr^118^, Ile^121^, Ile^123^, Ile^124^, Asp^125^, Pro^126^, Asp^166^, Lys^168^, Phe^169^, Tyr^171^, Ser^217^, Met^218^, Ala^221^, Val^259^, Cys^260^, His^261^, Asn^262^, Arg^263^, Glu^312^, Cys^313^, and Leu^314^ (Figure 13a). Baicalin also interacts with DdB1 at the same binding site (Figure 13b). In non-specific interactions, Hesperidin shows the highest binding affinity (binding energy −10.89, and dissociation constant 10.4 nM) for Integrin alpha V Beta 6 head protein normally involved in cell adhesion (Figure 13c). The top four non-specific interacting partners of Glycyrrhizin, Hesperidin, and Baicalin are detailed in Figure 13a–c. In contrast, the binding energies and dissociations constants for all 100 non-specific proteins are given in Table S2.

### 2.8. ADMET Properties of Selected Ligands

Lipinski’s Rule of Five [46], Ghose filter (Amgen) [47], Veber’s (GSK) [48] rules are used to predict ADME properties. According to the pharmacokinetic properties, all compounds show Gastrointestinal low absorption except 18-β glycyrrhetinic acid (GA), lopinavir, and euchrestaflavanone A, which have high absorption. These compounds have the least BBB permeability, and no CYP inhibition was observed (Table 2).

## 3. Discussion

Antioxidants including Glycyrrhizin, 18,β Glycyrrhetinic acid, Rhodiolin, Baicalin, and Hesperidin have shown remarkable potential in targeting various SARS-CoV-2 enzymes and proteins. Glycyrrhizin, largely found in licorice root, has already been found active against many viral proteases, including herpesviruses [49,50], flaviviruses [51], and Human Immunodeficiency Virus [52]. Glycyrrhizin was already used to treat patients with hepatitis C [53] and upper respiratory tract infections [54]. Glycyrrhizin, Glycyrrhizic acid, its other derivatives and Baicalin were the first compounds found active against SARS coronavirus (SARS-CoV-1) [55,56]. Flavonoids and their derivatives have been reported to inhibit various SARS-CoV-2 proteins [57]. Hesperidin and quercetin have also been found good antiviral agents [58,59].

The binding site for M^Pro^ has already been defined by X-ray crystallographic structure (PDB ID 6WQF) obtained at room temperature [38] where His^41^ and Cys^45^ form a catalytic dyad to interact with bound ligand. Other amino acids involved in the stabilization of the active site were Ser^46^, Leu^141^, Asn^142^, Glu^166^, Pro^168^, Gln^189^, Thr^190^, Ala^191^. This active site is situated in a cleft between domains I and II [60,61]. In our study, Glycyrrhizin shows two binding sites; one includes the conventional active site of the enzyme, while the second interactions include an allosteric binding site. A previous docking analysis of Glycyrrhizin against M^Pro^ has shown a binding energy value −7.81 kcal·mol^−1^ where it interacts with the proposed active site of the enzyme [62]. In another study, glycyrrhizic acid, Glabridin and Liquiritigenin show strong binding interactions (with a binding energy of −7.0 to −8.0 kcal·mol^−1^) with M^Pro^ conventional active site [63]. The docking analysis of M^Pro^ with FDA-approved anti-viral compounds and library of active phytochemicals [64] shows Nelfinavir potent against M^Pro^. Many natural compounds are found to be potential inhibitors of M^Pro,^ including Leucoefdin [65], Leupeptin [66], Rutin [67], cannabisin-A, isoacetoside [68], epigallocatechin gallate, and epicatechin gallate [69]. Recently, Glycyrrhizin has been found to indirectly inhibit the SARS-CoV-2 replication by Inhibiting M^Pro^ enzyme activity [70]. In our study, many ligands, including Glycyrrhizin, have been found to interact with Mpro allosteric binding sites (Table 1 and Table S1, Figure 2). In a previous study, 2400 FDA-approved drugs have been screened against M^Pro^ allosteric binding sites, where selinexor, bromocriptine, Dihydroergotamine, nilotinib, entrectinib, digitoxin, and diosmin have shown promising binding to the enzyme [71].

PL^Pro^ consists of an N-terminal ubiquitin-like (Ubl) domain (1–60 a.a) and a catalytic region with a right-handed thumb-palm-fingers architecture. The PL^Pro^ binding site is found in the thumb and palm domain and is characterized by the presence of a catalytic triad (Cys^111^, His^272^, and Asp^286^) [72]. In our study, Baicalin and Hesperidin have been found to be potential PL^pro^ inhibitors that interact with their proposed active site (Figure 4). GRL0617 with PL^pro^ shows binding to the same site [73], whereas π-π interactions with Tyr^268^ have shown definite inhibition of PL^pro^ activity. Natural compounds like Caesalpiniaphenol A, and Sappanone B, also interact with Try^268^. Corylifol A, chromen, darunavir, sofosbuvir and some other drugs were screened against PL^Pro^. These drugs were found to bind near the proposed catalytic triad [74]. Phytochemicals from *Vitex negundo* L. are also found active against PL^Pro^ [75]. Along with natural compounds, many approved antibacterial and antiviral drugs also have been repurposed [76,77,78].

Here, MD simulations validate the role of Tyr^268^ in Baicalin, Hesperidin, and Solophenol enzyme complexes (Figure 5). All catalytic residues seem to interact with these ligands in 30 ns MD simulation. In a previous study, MD simulations for PL^pro^ were found stabilized after 12 ns and remained stable until 50 ns [79]. Baicalin can be extracted from the roots of *Scutellaria baicalensis* Georgi. In another study, six phytochemicals, including Baicalin, rutin, biopterin, licoleafol, luteolin, and quercetin, have shown binding to PL^pro^ [80]. In addition, Baicalin showed antiviral activity against dengue virus (DENV) [81], Influenza A virus (IAV) [82], Zika virus (ZIKV) [83], Chikungunya virus (CHIKV) [84], and Human Immunodeficiency Virus 1 (HIV-1) [85].

Glycyrrhizin, Hesperidin, and Biacalin show strong interactions with RdRp. Glycyrrhizin binds in the enzyme’s potential active site pocket while making H-bonding with Asp^760^ and other residues (Figure 6). Asp^760,761^ have been proposed as active site residues, while Tyr^619^, Cys^622^, Ser^759^, Ala^762^, Glu^811^, Cys^813^, and Ser^814^ are found in potential binding sites for Ribavirin, Remdesivir, and other antivirals interactions [86]. In a screening with flavonoid compounds, Delphinidin 3-O-beta-D-glucoside 5-O-(6-coumaroyl-beta-D-glucoside) complex with RdRp has been found most stable, where Asp^760,761^ have been found in the ligand-binding site [87]. Lanreotide, Argiprestocin, Demoxytocin, and Polymyxin B1 also interact with Asp^760^. Previously, polyphenols with binding energy <7.0 have been reported to interact with RdRp, while Remdesivir showed binding energy of 7.9 kcal·mol^−1^ [88] and interacts with a similar ligand-binding site as for Glycyrrhizin, Hesperidin. This site includes Asp^760,761^, and Glu^811^ for Remdesivir [89,90]. In another study, approved antivirals including Ribavirin, Remdesivir, Sofosbuvir, Galidesivir, and Tenofovir have shown strong interactions with RdRp, where Ribavirin have shown binding energy of −8.7 kcal/mol [86]. Many compounds from the ZINC database have been screened against RdRp and 40 ns MD simulations were performed for Rifabutin, ZINC09128258 and ZINC09883305 [91].

Glycyrrhizin strongly binds to the S2 subunit of the spike protein. Although the binding residues are different from the receptor-binding domain (331–524 a.a.), it is expected that this interaction may bring the conformation change in the protein that may affect the receptor binding. This conformational change is also indicated by MD simulation showing large RMSD and RMSF changes in the receptor-binding region and formation of the close complex (low R_g_) (Figure 8 and Figure S4). A number of flavonoid compounds were docked to spike protein and naringin has been found as the most potent compound against SARS-CoV-2 spike protein [92]. In our study, Glycyrrhizin showed the best binding energy against the open and close state of spike glycoproteins. Most of the Glycyrrhizin’s interactions have been found with the S2 subunit of the spike protein (Figure 8b). In one study, 66 compounds were found to interact with RBD of spike protein and Glycyrrhizic acid was found to be the most potent antiviral and spike protein inhibitor [93]. Rhodiolin and Hesperidin bind to the N-terminal domain of spike protein and show the second-best binding energies (Figure 8b). Mutations in spike protein lead to new SARS-CoV-2 variants. Glycyrrhizin was docked against the spike protein variants initially reported from Brazil (B.1.1.28), South Africa (B.1.351), and the United Kingdom (B.1.1.17). The variant B.1.1.7 was first reported in the United Kingdom on 14 December 2020 [94,95], B.1.1.28 originated from Brazil [96]. Both mutations, B.1.1.7 and B.1.1.28, carry the N501Y mutation in the RBD of the spike protein. Later, the B.1.351 variant (20H/501Y.V2) was first reported in South Africa [97], was marked by mutation of K417N and E484K in the RBD region of the spike protein. Surprisingly, the affinity of the Glycyrrhizin slightly decreased against the spike protein variants, though it still shows considerable binding energy and dissociation constant (Figure S6). Catechins and tamibarotene have been found to interact strongly with the UK variant and triple SARS-CoV-2 variant [98,99].

In the case of SARS-CoV-2 helicase, the interface between RecA1 and RecA2 domains contains the active site residues for helicase enzyme, including Lys^288^, Ser^289^, Asp^374^, Glu^375^, Gln^404^, and Arg^567^ [100,101]. In this study, Glycyrrhizin binds to an allosteric site near the catalytic cleft, while Glycyrrhetinic acid interacts with residues in stalk and 1B domain. Triphenyamine and Darunavir have been reported to bind the same active site [102]. Rutin, xanthones, and many other polyphenols have been reported to be ATPase inhibitors [59]. A database of 14,000 phytochemicals has been docked against helicase using virtual screening; out of them, 368 compounds have been found to be potent helicase inhibitors [103]. Picrasidine M from a herb *Picrasma quassioides* shows the best binding to helicase, where it forms 2 H-bonds with Ser^289^ and one H-bond with Gln^404^ and Arg^567^ [103]. E-channel blockers are demonstrated to be potent antivirals by protecting hosts cells from death. Glycyrrhizin, Glycyrrhetinic acid, and Baicalin have also shown binding to E-channel protein. Proanthocyanidins have been reported to inhibit the MPro and E-channel protein of the SARS-CoV-2 [104].

**Figure 14:**
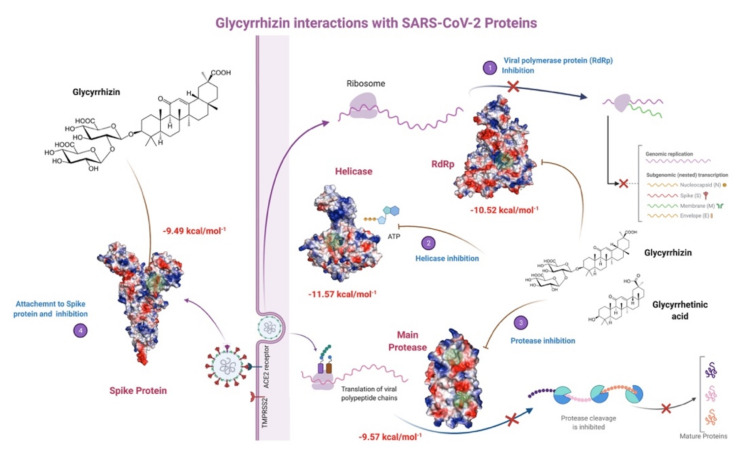
Glycyrrhizin interactions with SARS-CoV-2 proteins (prepared with bio renderer).

Glycyrrhizin seems to interact with four SARS-CoV-2 key proteins with high affinity, where its binding with helicase and RdRp has been found to be more stable (Figure 14). Glycyrrhizin has also been reported to interact with TMPRSS2, involved in viral penetration into the host cells [105]. It also indicates that licorice root, a potential source of Glycyrrhizin, may be used as a possible cure and a household remedy for COVID-19. The non-specific interactions of Glycyrrhizin show that it may interact with Ddb1 and other non-specific human blood proteins. Glycyrrhizin as Glycyrrhizic acid from *Glycyrrhiza glabra* and as a derivative in the form of β-Glycyrrhetinic acid are reported to show penetration through the blood-brain barrier and are non-carcinogenic [63] (Table 2). This study shows that natural antioxidant compounds, either partially purified or in crude form, may provide protection against SARS-CoV-2 severity and complications by interacting with its key enzymes. Although we have performed 30 ns MD simulations as in the case of many protein-ligand complexes equilibrium was achieved and complex was found stable, longer MD simulations (>100 ns) in future studies may help to better understand the behavior and stability of the complexes. In vivo studies for the selected compounds are also recommended to probe the computational result and may help in the formulation of natural antivirals with less toxicity and more efficacy.

## 4. Conclusions

In the current pandemic situation, COVID-19′s battle with humanity is still continued. The availability of various vaccines has eased the aggravating situation, but the rise of SARS-CoV2 variants warns that humans have to live with the virus, evading its devastating effects. Potential natural cures/medications/home remedies without any side effects seems a viable solution in the current circumstances. Our study has examined and screened more than a hundred natural compounds from plants against six SARS CoV-2 proteins by using molecular docking and molecular dynamics simulations to identify potential bioactive compounds. Glycyrrhizin was found as the best ligand showing strong inhibition of five SARS CoV-2 proteins. Glycyrrhizin is present in a large amount in an inexpensive household herb licorice already registered for its magical curative properties against a number of diseases. Other phytochemicals found potent against SARS-CoV2 include Hesperidin and Baicalin present in citrus fruits and many other plants. In our study, Glycyrrhizin, Hesperidin, and Baicalin were docked against non-specific human blood proteins and have shown interactions with DNA binding proteins. Based on our findings, we suggest that further in vivo evaluation of Glycyrrhizin and its sister compounds as potential antivirals will signify their role in the treatment and management of COVID-19.

## 5. Materials and Methods

A total of 115 natural compounds with established therapeutic properties were targeted against six SARS-CoV-2 proteins by molecular docking. The compound structures were obtained from various chemical databases, including PubChem (https://pubchem.ncbi.nlm.nih.gov/, accessed on 25 March 2021), ChemSpider (https://chemspider.com, accessed on 25 March 2021) and MolPort (https://www.molport.com/, accessed on 25 March 2021) (Table S1). The compound structures were obtained in form of The Spatial Data File (SDF) and optimized using the MM1 forcefield in YASARA Structure ver. 20.7.4 [106]. The ligand structures were merged in a single file prepared for virtual screening. Six SARS-CoV-2 proteins were obtained from Protein Data Bank (PDB), including Main Protease (M^Pro^, PDB ID 6Y84), Papain-like protease (PL^pro^, PDB ID 6WX4), RNA-dependent RNA polymerase (RdRp, PDB ID 6M71), Spike Glycoprotein (S, open and closed state, PDB IDs 6VXX and 6VYB), Helicase (Nsp13) (PDB ID 6ZSL), and E-Channel (Envelop small membrane protein) (PDB ID 5 × 29) (Figure 1). Spike protein variants’ structures as well as structures for all selected non-specific proteins were also obtained from PDB (Table S2a–c). The single-chain structures for all proteins were prepared using YASARA Structure ver. 20.7.4 [106] and heteroatoms were removed.

### 5.1. Molecular Docking

The ligand-protein interactions and binding energies were calculated by applying a virtual screening module in YASARA software version 20.7.4 [106] that uses a modified AutoDock-Lamarckian Genetic Algorithm. The parameters used for the virtual screening and molecular docking have been described earlier [107], where AMBER03-FF was used with a hundred global docking runs and by keeping the random seed value of 1000. AutoDock local search (LGA-LS) was also used for selected top five ligands to reassure best binding and energy minimization. Protein-ligand interactions were mapped in terms of binding energies and dissociation constants, while the docking scores were calculated by Equation (1)

ΔG = ΔG_(van der Waals)_ + ΔG_(H-bonding)_+ ΔG_(electrostatic)_+ ΔG _(torsional free energy)_ + ΔG_(desolvation energy)_ (1)

LigPlus [108] was used to obtain and ligand-protein interactions. The specific ligand was selected in the ligand-protein complex file and interactions including H-bonds were mapped. Non-specific Interactions of selected ligands against human blood proteins were studied by an inverse docking procedure where more than 100 human blood protein targets were screened against the selected ligands (Table S2a–c).

### 5.2. Molecular Dynamic Simulations

**Figure 15:**
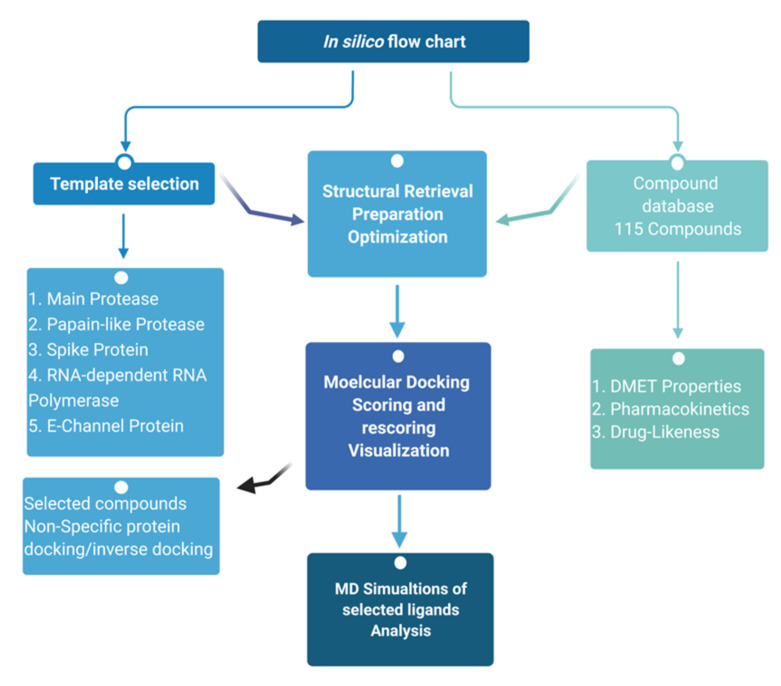
Flow diagram of Methodology.

Molecular Dynamic simulations (MDS) were performed using YASARA Structure ver. 20.7.4 [106] with AMBER14 as a force field as described before [109]. The simulation cell was prepared by providing 20 Å water-filled space around the fully mobile protein with a density of 0.997 g/mL. The system was neutralized with 0.9% NaCl while maintaining 298 K temperature, pH 7.4, periodic boundaries, and 7.86 cut-off for long-range coulomb electrostatics forces. After the initial steepest descent minimization, MDS was performed at the rate of 1.25–2.50 fs time steps, and the simulation snapshot was saved every 100 ps. MDS of 10–30 ns were calculated for different proteins depending on the number of atoms in the simulation cell. The raw data were analyzed using GraphPad Prism ver. 7.0. [110]. RMSD and RMSF values were tabulated and analyzed for the fluctuations. A docking and MD simulation flow chart had been given in Figure 15.

### 5.3. Pharmacokinetics and Drug-Likeness

The pharmacokinetic properties and drug-likeness prediction of the top 10 li were performed by the SwissADME server (http://www.swissadme.ch/, accessed on 20 April 2021). It calculates the topological polar surface area (TPSA), logP (lipophilicity), and logS (solubility). The drug-likeness was predicted by following Lipinski, Ghose, and Veber rules and bioavailability scores [46,47,48]. The Lipinski’s Rule of Five states that the absorption or permeation of a molecule is more likely when the molecular mass is under 500 g/mol, the value of log P is lower than 5, and the molecule has utmost 5 H-donor and 10 H-acceptor atoms [46]. Ghose filter (Amgen) [47] defines drug-likeness based on log P between −0.4–5.6, MW between 160–480, molar refractivity between 40–130, and the total number of atoms between 20–70. Veber (GSK) [48], the rule defines drug-likeness as rotatable bond count ≤10 and polar surface area (PSA) ≤ 140.

## Figures and Tables

**Table 1:** The best five docked ligands to Main Protease, Papain-like protease, RNA-dependent RNA polymerase, Spike Glycoprotein and Helicase, E-Channel; their binding energies, dissociations constants and active site residues.

Target	Phytochemicals	Binding Energy (kcal/mol)	Dissociation Constant (µM)	Active Site Residue
**Main Protease (M^Pro^)**	Glycyrrhizin	−9.57	0.11	Thr^24^, Thr^25^, Thr^26^, Leu^27^, Gly^29^, His^41^, Cys^44^, Ser^46^, Met^49^, Tyr^118^, Asn^119^, Asn^142^, Gly^143^, Cys^145^, His^163^, His^164^, Met^165^, Glu^166^
		−9.46	0.76	Arg^131^, Asn^133^, Thr^135^, Val^137^, Thr^169^, Val^171^, Ala^194^, Gly^195^, Thr^196^, Asp^197^, Thr^198^, Thr^199^, Tyr^237^, Asn^238^, Tyr^239^, Leu^272^, Leu^286^, Leu^287^, Asp^289^
	18,β-Glycyrrhetinic acid	−9.19	0.35	Lys^5^, Arg^131^, Lys^137^, Asp^197^, Thr^199^, Tyr^237^, Tyr^239^, Leu^272^, Leu^286^, Leu^287^, Glu^288^, Asp^289^, Glu^290^
	Rhodiolin	−9.05	0.23	Thr^25^, His^41^, Cys^44^, Thr^45^, Ser^46^, Met^49^, Leu^141^, Asn^142^, Gly^143^, Ser^144^, Cys^145^, His^163^, Met^165^, Glu^166^, Leu^167^, Pro^168^, Arg^188^, Gln^189^, Thr^190^, Gln^192^
	Baicalin	−8.85	0.33	Val^104^, Ile^106^, Gln^110^, Thr^111^, Asn^151^, Ile^152^, Asp^153^, Tyr^154^, Pro^252^, Thr^292^, Phe^294^, Asp^295^, Val^297^, Arg^298^, Val^303^
	Silymarin	−8.71	0.41	Thr^24^, Thr^25^, Thr^26^, Leu^27^, His^41^, Cys^44^, Thr^45^, Ser^46^, Met^49^, Gly^143^, Cys^145^, His^164^, Met^165^, Glu^166^, Leu^167^, Pro^168^, Arg^188^, Gln^189^, Thr^190^, Gln^192^
**Papain like protease (PL^Pro^)**	Baicalin	−10.82	0.01	Cys^155^, Asn^156^, Lys^157^, Glu^161^, Leu^162^, Gly^163^, Asp^164^, Val^165^, Arg^166^, Glu^167^, Tyr^171^, Val^202^, Met^206^, Met^208^, Pro^248^, Tyr^264^, Tyr^268^, Gln^269^, Tyr^273^
	Hesperidin	−10.61	0.02	Lys^157^, Thr^158^, Glu^161^, Leu^162^, Gly^163^, Asp^164^, Val^165^, Glu^167^, Leu^199^, Glu^203^, Tyr^207^, Met^208^, Lys^232^, Pro^248^, Tyr^264^, Tyr^268^, Gln^269^, Cys^270^, Tyr^273^, Thr^301^
	Naringen	−10.17	0.04	Cys^155^, Asn^156^, Glu^161^, Leu^162^, Gly^163^, Asp^164^, Arg^166^, Gln^167^, Ser^170^, Leu^185^, Leu^199^, Val^202^, Glu^203^, Met^206^, Tyr^207^, Met^208^, Ile^222^, Pro^223^, Lys^232^, Tyr^268^
	Flemiflavanone D	−10.07	0.04	Lys^157^, Glu^161^, Leu^162^, Gly^163^, Asp^164^, Arg^166^, Glu^167^, Ser^170^, Val^202^, Glu^203^, Met^206^, Tyr^207^, Met^208^, Tyr^264^, Tyr^268^, Gln^269^, Tyr^273^
	Euchrestaflavanone A	−9.95	0.05	Lys^157^, Glu^161^, Leu^162^, Gly^163^, Asp^164^, Arg^166^, Glu^167^, Ser^170^, Val^202^, Glu^203^, Met^206^, Tyr^207^, Met^208^, Tyr^268^
**RNA-dependent RNA polymerase (RdRP)**	Glycyrrhizin	−10.52	0.03	Asp^452^, Tyr^455^, Lys^551^, Arg^553^, Ala^554^, Arg^555^, Thr^556^, Trp^617^, Asp^618^, Tyr^619^, Pro^620^, Lys ^621^, Cys^622^, Asp^623^, Arg^624^, Ser^759^, Asp^760^, Asp^761^, Ala^762^, Lys^798^, Cys^799,^ Trp^800^, Glu^811^, Phe^812^, Cys^813^ Ser^814^
		−9.96	0.05	Asp^452^, Tyr^455^, Arg^553^, Ala^554^, Arg^555^, Trp^617^, Asp^618^, Lys^621^, Cys^622^, Asp^623^, Arg^624^, Ser^759^, Asp^760^, Asp^761^, Lys^798^, Trp^800^, Glu^811^, Cys^813^, Ser^814^
	Hesperidin	−9.53	0.1	Val^166^, Tyr^456^, Met^542^, Arg^553^, Ala^554^, Arg^555^, Thr^556^, Val^557^, Ala^558^, Asp^618^, Tyr^619^, Pro^620^, Lys^621^, Cys^622^, Asp^623^, Arg^624^, Lys^676^, Thr^680^, Ser^681^, Ser^682^, Phe^793^, Ser^795^, Lys^798^
	Baicalin	−9.01	0.25	Val^31^, Tyr^32^, Lys^47^, Tyr^129^, Ala^130^, His^133^, Phe^134^, Asp^135^, Asn^138^, Cys^139^, Thr^141^, Asn^705^, Ala^706^, Ser^709^, Thr^710^, Lys^780^, Asn^781^, Ser^784^
	Naringen	−8.54	0.55	Tyr^32^, Lys^47^, Tyr^129^, His^133^, Phe^134^, Asp^135^, Asn^138^, Ser^709^, Thr^710^, Asp^711^, Lys^714^, Ala^771^, Ser^772^, Gln^773^, Gly^774^, Ser^778^, Lys^780^, Asn^781^, Ser^784^
	Oleuropein	−8.31	0.81	Tyr^32^, Lys^47^, Phe^48^, Tyr^129^, Ala^130^, His^133^, Phe^134^, Asp^135^, Asn^138^, Cys^139^, Asp^140^, Thr^141^, Leu^142^, Thr^710^, Asp^711^, Lys^714^, Ser^778^, Lys^780^, Asn^781^, Ser^784^
**Spike protein (S)**	Glycyrrhizin	−9.49	0.11	Tyr^741^, Ile^742^, Cys^743^, Gly^744^, Asp^745^, Phe^855^, Asn^856^, Val^963^, Lys^964^, Leu^966^, Ser^967^, Ser^975^, Val^976^, Leu^977^, Asn^978^, Arg^1000^
		−9.29	0.16	Val^47^, His^49^, Lys^304^, Met^740^, Tyr^741^, Ile^742^, Cys^743^, Gly^744^, Asp^745^, Phe^855^, Asn^856^, Val^963^, Lys^964^, Leu^966^, Ser^967^, Ser^975^, Val^976^, Leu^977^, Asn^978^, Arg^1000^
	Rhodiolin	−8.68	0.43	Arg^102^, Gly^103^, Trp^104^, Ile^119^, Asn^121^, Val^126^, Ile^128^, Phe^168^, Tyr^170^, Ser^172^, Arg^190^, Phe^192^, Ile^203^, His^207^, Leu^226^, Val^227^, Asp^228^, Leu^229^
	Hesperidin	−8.53	0.56	Tyr^38^, Asp^40^, Lys^41^, Val^42^, Phe^43^, Arg^44^, Lys^206^, Phe^220^, Ser^221^, Ala^222^, Glu^224^, Pro^225^, Leu^226^, Tyr^279^, Gly^283^, Thr^284^
		−8.18	1.01	Asn^710^, Thr^1076^, Ser^1097^, Gly^1099^, Thr^1100^, His^1101^, Trp^1102^, Ile^1114^, Ile^1115^, Val^1133^, Asn^1134^, Asn^1135^, Thr^1136^, Tyr^1138^
	Umbelliprenin	−8.12	1.11	Trp^104^, Ile^119^, Asn^121^, Val^126^, Ile^128^, Phe^168^, Tyr^170^, Ser^172^, Arg^190^, Phe^192^, Phe^194^, Ile^203^, Ser^205^, Lys^206^, His^207^, Leu^226^, Val^227^, Leu^229^
	18,β-Glycyrrhetinic acid	−8.08	1.2	Tyr^38^, Asp^40^, Lys^41^, Val^42^, Phe^43^, Arg^44^, Glu^224^, Pro^225^, Tyr^279^, Gly^283^
		−7.99	1.38	His^1101^, Trp^1102^, Phe^1103^, Ile^1114^, Ile^1115^, Asn^1135^, Thr^1136^, Val^1137^, Tyr^1138^, Asp^1139^, Gln^1142^, Pro^1143^
	Silyhermin	−8.05	1.25	Arg^355^, Tyr^396^, Pro^426^, Asp^428^, Phe^429^, Thr^430^, Lys^462^, Pro^463^, Phe^464^, Ser^514^, Phe^515^, Glu^516^, Leu^517^, Leu^518^
	Ellagic acid	−8.1	1.16	Ser^730^, Met^731^, Thr^732^, Lys^733^, Gln^774^, Thr^778^, Phe^823^, Val^860^, Leu^861^, Pro^862^, Pro^863^, Asp^867^, Ile^870^, Ala^1056^, Pro^1057^, His^1058^, Gly^1059^
**Helicase**	Glycyrrhizin	−11.57	0.003	Pro^175^, Leu^176^, Asn^177^, Lys^202^, Leu^405^, Pro^406^, Ala^407^, Pro^408^, Arg^409^, Leu^412^, Thr^413^, Gly^415^, Thr^416^, Leu^417^, Phe^422^, Ser^485^, Ser^486^, Pro^514^, Tyr^515^, Asn^516^, Asn^519^, Thr^532^, Val^533^, Asp^534^, His^554^, Asn^557^, Asn^559^, Arg^560^
	18,β-Glycyrrhetinic acid	−9.91	0.054	Ala^4^, Val^6^, Arg^15^, Arg^21^, Arg^22^, Pro^23^, Phe^24^, Arg^129^, Leu^132^, Phe^133^, Glu^136^, Pro^234^, Leu^235^, Ser^236^
	Solophenol A	−9.4	0.13	Pro^175^, Leu^176^, Asn^177^, Arg^178^, Thr^199^, Phe^200^, Glu^201^, Lys^202^, Gly^203^, Asp^204^, Val^210^, Tyr^211^, Arg^212^, Val^484^, Ser^486^, Asn^516^, Ser^517^, Asn^519^, Ala^520^, Thr^530^, Thr^532^, Asp^534^
	Hesperidin	−8.93	0.283	Lys^139^, Glu^142^, Glu^143^, Lys^146^, Arg^178^, Asn^179^, Val^181^, Glu^197^, Thr^228^, His^230^, Cys^309^, Arg^337^, Arg^339^, Met^378^, Ala^379^, Thr^380^, Tyr^382^, Asp^383^, Ala^407^, Pro^408^, Thr^410^
	Baicalin	−8.9	0.29	Asn^177^, Arg^178^, Thr^199^, Phe^200^, Glu^201^, Lys^202^, Arg^212^, Asn^516^, Asn^519^, Ala^520^, Thr^530^, Gln^531^, Thr^532^, Asp^534^
**E-channel protein**	Glycyrrhizin	−10.07	0.04	Arg^61^, Asn^64^, leu^28^, val^29^, leu^31^, ala^32^, ile^33^, ala^36^, arg^38^, Leu^27^, Thr^30^, Leu^31^, Leu^34^, Leu^37^, Leu^39^, Tyr^42^, Cys^43^, Ile^46^, Val^47^, Val^49^, Ser^50^, Leu^51^, Pro^54^, Tyr^57^
	18,β-Glycyrrhetinic acid	−9.72	0.07	Arg^61^, Leu^28^, Val^29^, Leu^31^, Ala^32^, Thr^35^, Leu^27^, Thr^30^, Leu^31^, Ile^46^, Val^47^, Leu^51^, Pro^54^, Tyr^57^
	Umbelliprenin	−8.90	0.3	Ala^22^, Val^25^, Phe^26^, Leu^28^, Val^29^, Leu^19^, Phe^20^, Ala^22^, Phe^23^, Val^24^, Phe^26^, Leu^27^, Asn^64^, Leu^65^
	Euchrestaflavanone A	−8.90	0.3	Arg^61^, Asn^64^dval^25^dphe^26^dleu^28^dval^29^, Ala^22^, Phe^23^, Phe^26^, Leu^27^, Thr^30^, Leu^34^, Cys^43^, Ile^46^, Val^47^, Val^49^, Ser^50^, Leu^51^
	Baicalin	−8.89	0.3	Ala^22^, Val^25^, Phe^26^, Val^29^, Phe^20^, Ala^22^, Phe^23^, Val^24^, Phe^26^, Leu^27^, Thr^30^, Arg^61^, Asn^64^, Leu^65^
	Silibinin A	−8.13	1.1	Leu^28^, Leu^31^, Ala^32^, Thr^35^, Cys^40^, Ile^46^, Ser^50^, Leu^51^, Lys^53^, Pro^54^, Phe^56^, Tyr^57^, Tyr^59^, Ser^60^

**Table 2:** ADMET properties of selected ligands.

Compounds	Molecular Formula	Molecular Weight	HBA	HBD	TPSA	Log P−0.7–5	Log S 0–6	GI Absorption	BBB Permeant	CYPP1A2 Inhibitor	CYP2D6 Inhibitor	Log Kp (Skin Permeation), cm/s	Lipinski Violations	Ghose Violations	Veber Violations	Bioavailability Score
**Glycyrrhizin**	C_42_H_62_O_16_	822.93	16	8	267.04	1.49	−6.24	Low	No	No	No	−9.33	3 MW > 500, NorO > 10, NHorOH > 5	3 MW > 480, MR > 130, atoms > 70	1 TPSA > 140	0.11
**18,β Glycyrrhetinic acid**	C_30_H_46_O_5_	470.68	4	2	74.6	5.13	−6.15	High	No	No	No	−4.27	1 MLOGP.4.16	3 WLOGP > 5.6, MR > 130.	Yes	1.56
**Rhodiolin**	C_25_H_20_O_10_	480.42	10	5	159.05	2.3	−4.99	Low	No	No	No	−7.02	0	1 MW > 480	1 TPSA > 140	0.55
**Baicalin**	C_21_H_18_O_11_	446.36	11	6	187.12	0.25	−3.41	Low	No	No	No	−8.23	3 NorO.10, NHorOH > 5	Yes	1 TPSA > 140	0.11
**Hesperidin**	C_28_H_34_O_15_	610.56	15	8	234.29	−1.06	−3.28	Low	No	No	No	−10.12	3 MW > 500, NorO > 10, NHorOH > 5	4 MW > 480, WLOGP < 0.4, MR > 130, #atoms > 70	1 TPSA > 140	0.17
**Solophenol A**	C_30_H_36_O_6_	492.6	6	4	107.22	5.69	−7.5	Low	No	No	No	−3.8	0	4 MW > 480, WLOGP < 0.4, MR > 130, #atoms > 71	1 TPSA > 140	0.55
**Naringin**	C_26_H_30_O_14_	566.51	14	8	225.06	−0.99	−2.68	Low	No	No	No	−10.39	3 MW > 500, NorO > 10, NHorOH > 5	3MW > 480, WLOGP < 0.4 MR > 130,	1 TPSA > 140	0.17
**Lopinavir**	C_37_H_48_N_4_O_5_	628.8	5	4	120	4.37	−6.64	High	No	No	No	−5.93	1 MW > 500	3 MW > 480, MR > 130, #atoms > 70	1 Rotors > 10	0.55

HBA: Number of Hydrogen bond acceptors HBD: Number of hydrogen bond donor, TPSA: Tropological polar surface area.

## Supplementary Materials

The following are available online at https://www.mdpi.com/article/10.3390/antibiotics10081011/s1, Figure S1: 30 ns Molecular dynamics simulation of Glycyrrhizin (blue) and Rhodiolin (red) docked with SARS-CoV-2 main protease (MPro), Figure S2: 30 ns Molecular dynamics simulation of Baicalin (red), Hesperidin (green), and Solophenol (blue) docked with SARS-CoV-2 PLpro, Figure S3: 30 ns Molecular dynamics simulation of Glycyrrhizin (red), Hesperidin (green), Baicalin (blue) in complex with SARS-CoV-2 RdRp. Figure S4: 30 ns Molecular dynamics simulation of Glycyrrhizin (red) and Hesperidin (green) in complex with SARS-CoV-2 spike protein (closed state) Figure S5: 30 ns Molecular dynamics simulation of Glycyrrhizin (red), Hesperidin (green), Baicalin (blue) in complex with SARS-CoV-2 helicase Figure S6: Glycyrrhizin interactions with variants of Spike protein of SARS-CoV-2, Table S1: Ligands docked to the SARS-CoV-2 proteins; binding energies, dissociation constants and active sites, Table S2a: Glycyrrhizin docking to nonspecific human blood proteins, Table S2b: Baicalin docking to nonspecific human blood proteins, Table S2c: Hesperidine docking to nonspecific human blood proteins and Table S3.
