# Supplementary material for: Effectiveness of Natural Antioxidants against SARS-CoV-2? Insights from the In-Silico World
Source: Antibiotics (Basel). 2021 Aug 20;10(8):1011. doi: 10.3390/antibiotics10081011 (PMC8388999; doi:10.3390/antibiotics10081011)
Supplement: Supplementary file 1 [file antibiotics-10-01011-s001.zip › Suppl Material/Suppl Material - Fig.pdf]

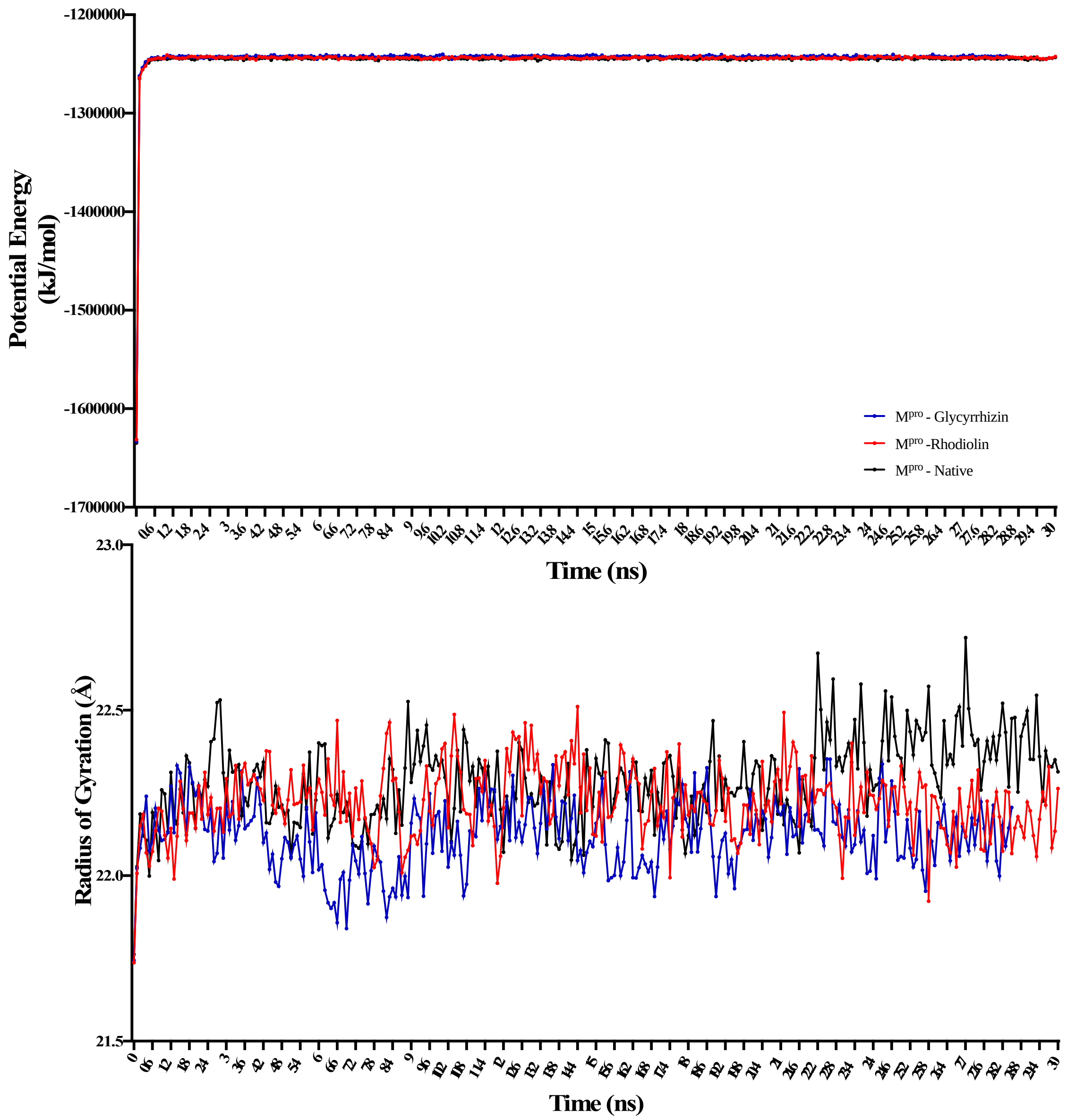

Figure S1: 30 ns Molecular dynamics simulation of Glycyrrhizin (blue) and Rhodiolin (red) docked with SARS-CoV-2 main protease (MPro)

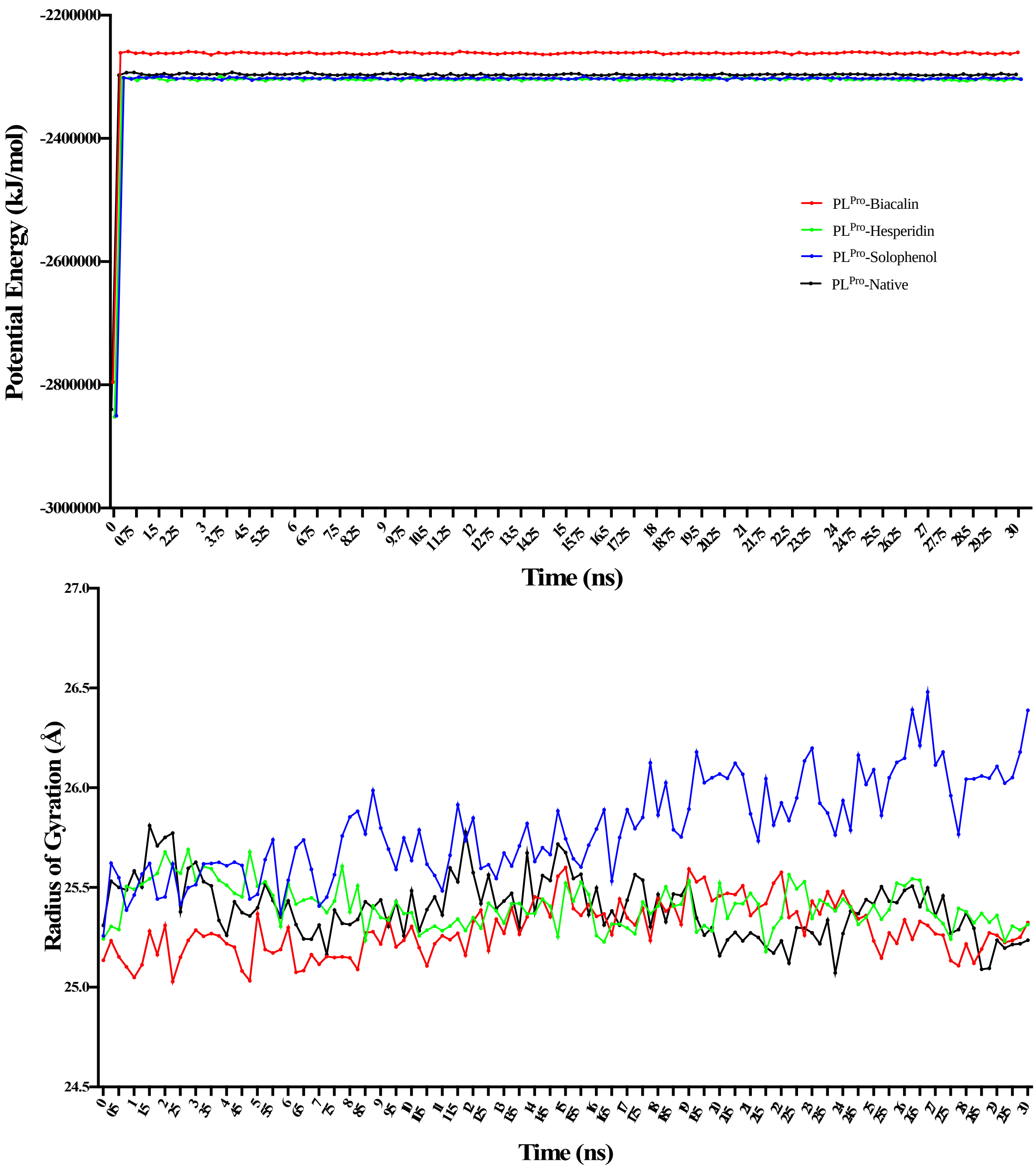

Figure S2: 30 ns Molecular dynamics simulation of Baicalin (red), Hesperidin (green), and Solophenol (blue) docked with SARS-CoV-2 PLpro

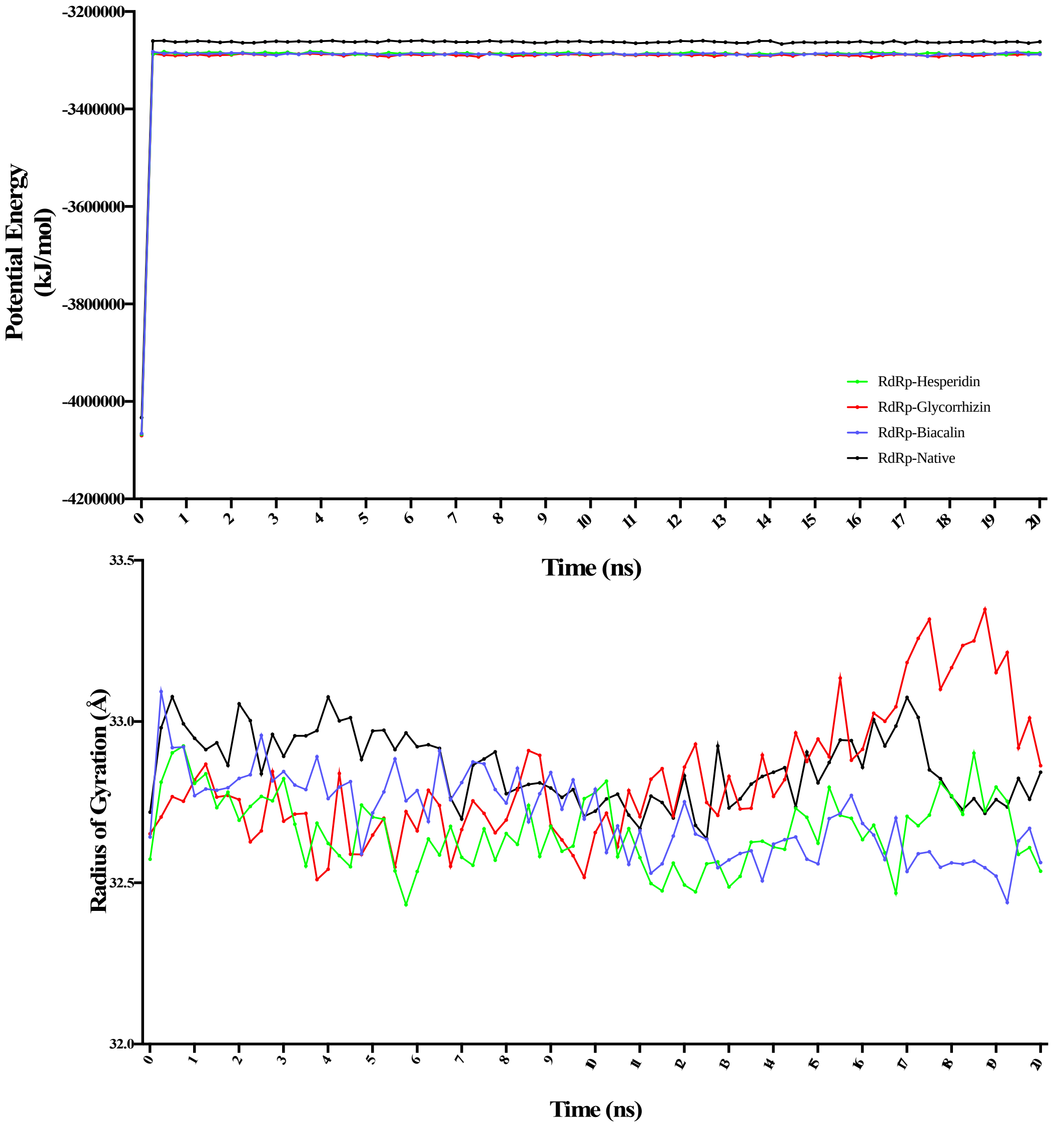

Figure S3: 30 ns Molecular dynamics simulation of Glycyrrhizin (red), Hesperidin (green), Baicalin (blue) in complex with SARS-CoV-2 RdRp.

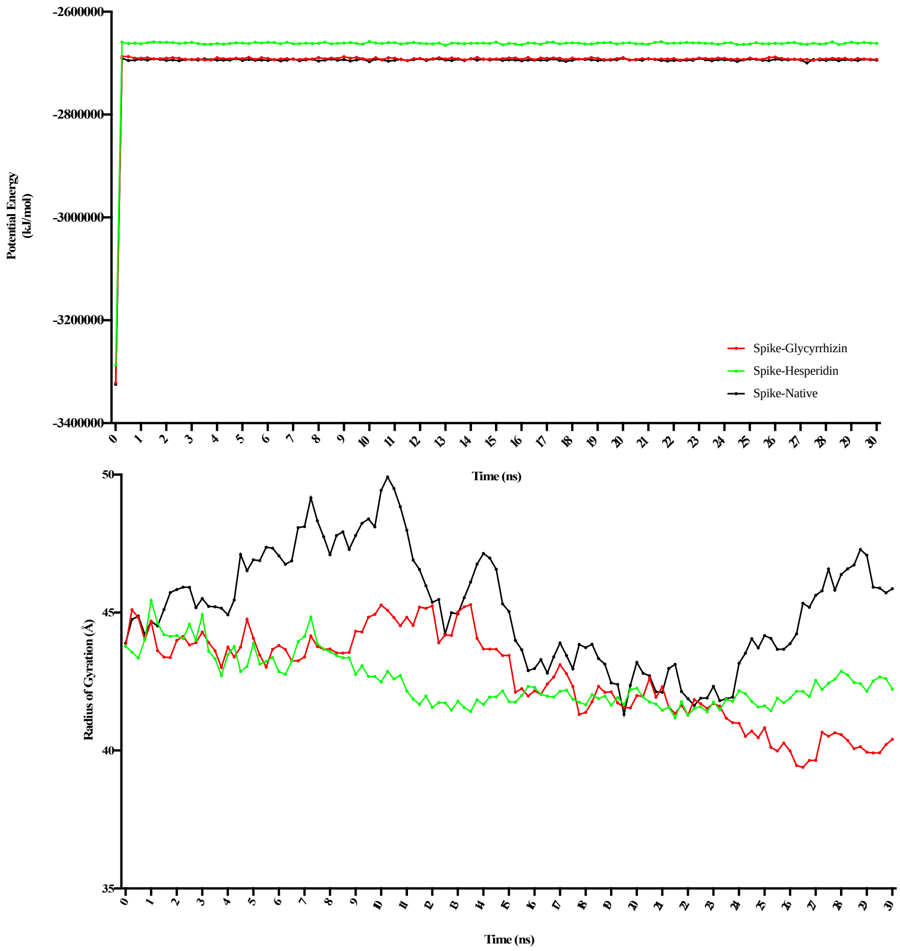

Figure S4: 30 ns Molecular dynamics simulation of Glycyrrhizin (red) and Hesperidin (green) in complex with SARS-CoV-2 spike protein (closed state)

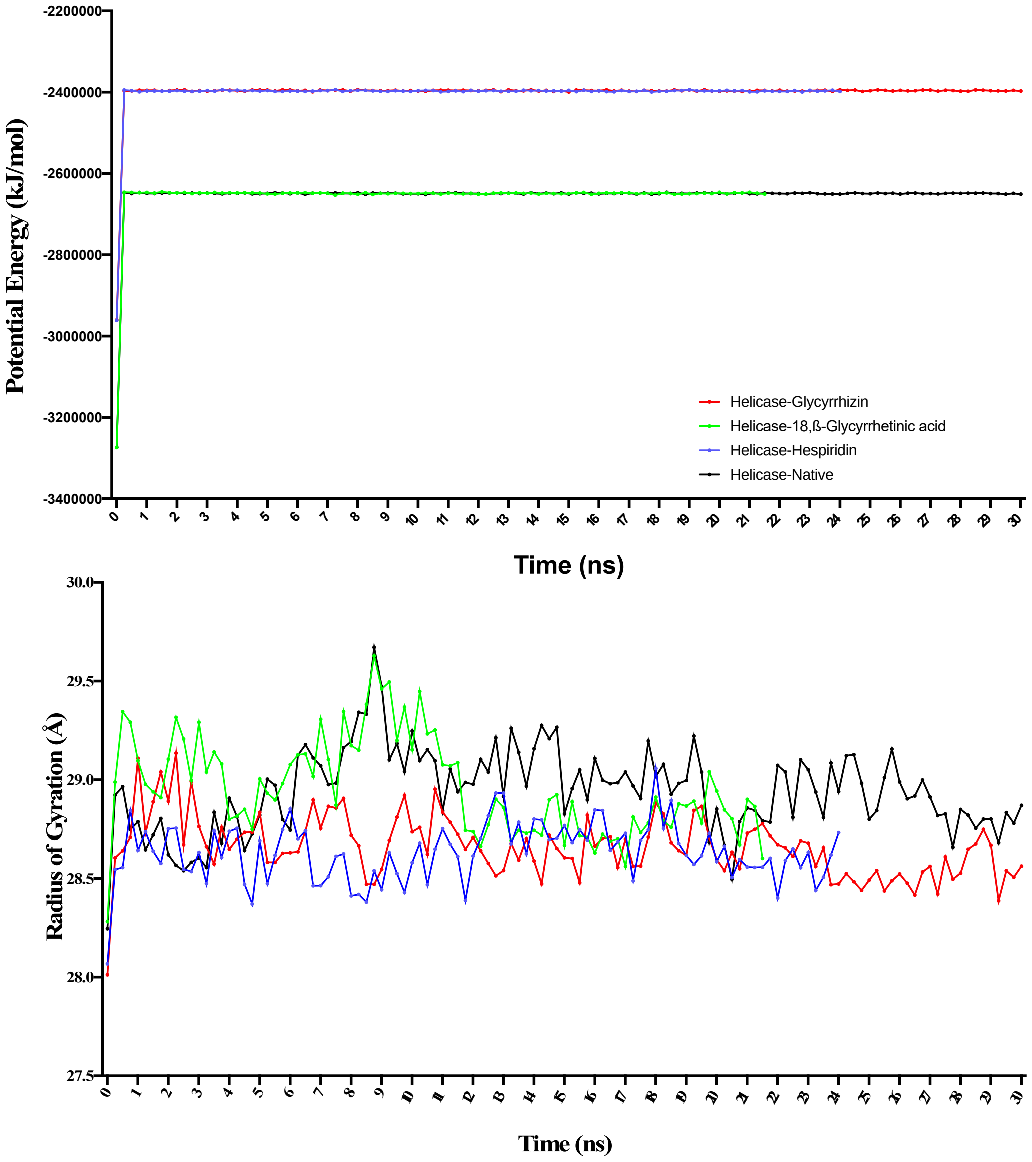

Figure S5: 30 ns Molecular dynamics simulation of Glycyrrhizin (red), Hesperidin (green), Baicalin (blue) in complex with SARS-CoV-2 helicase

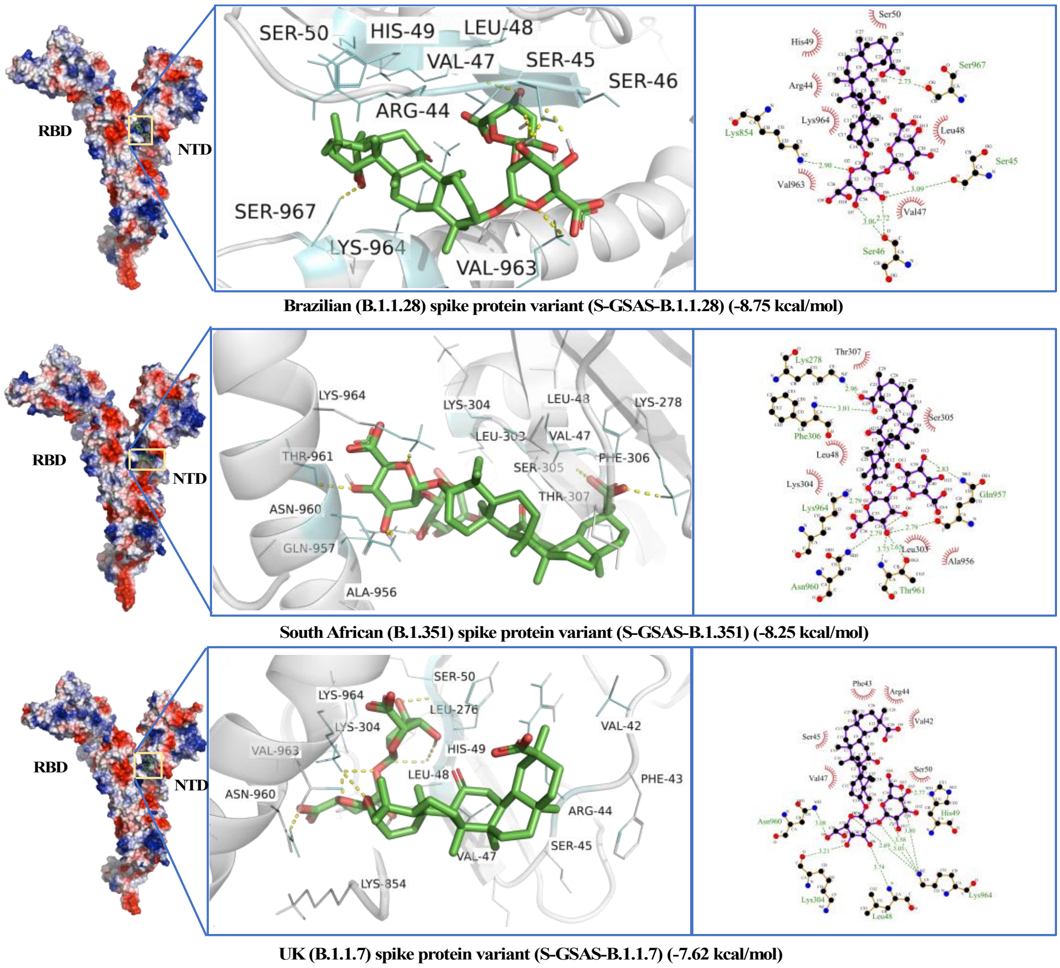

Figure S6: Glycyrrhizin interactions with variants of Spike protein of SARS-CoV-2
