# Supplementary material for: Effectiveness of Natural Antioxidants against SARS-CoV-2? Insights from the In-Silico World
Source: Antibiotics (Basel). 2021 Aug 20;10(8):1011. doi: 10.3390/antibiotics10081011 (PMC8388999; doi:10.3390/antibiotics10081011)
Supplement: Supplementary file 1 [file antibiotics-10-01011-s001.zip › Suppl Material/Table S1.pdf]

Table S 1 Ligands docked to the SARS-CoV-2 proteins; binding energies, dissociation constants and active sites

Sr.	Ligands	PubChem ID	Proteins	Binding energy (kcal/mol)	Dissociation Constant (µM)	Active site residues
1	4-Caffeoylquinic acid	124136972	Main Protease (MPro)	-7.96	1.45	Thr^{25}, Thr^{26}, Leu^{27}, His^{41}, Cys^{44}, Met^{49}, Asn^{142}, Gly^{143}, Cys^{145}, His^{164}, Met^{165}, Glu^{166}, Pro^{168}, Arg^{188}, Gln^{189}, Thr^{190}, Gln^{192}
			Papain like protease (PLPro)	-8.15	1.07	Arg^{166}, Ser^{170}, Val^{202}, Glu^{203}, Met^{206}, Tyr^{207}, Met^{208}, Gly^{209}, Ile^{222}, Pro^{223}
			RNA-dependent RNA polymerase (RdRP)	-7.02	7.1	Tyr^{149}, Ala^{176}, Asn^{177}, Leu^{178}, Gly^{179}, Glu^{180}, Arg^{181}, Gln^{184}, Asn^{213}, Arg^{249}, Thr^{252}, Leu^{261}
			Helicase	-8.3	0.826	Pro^{175}, Asn^{177}, Asn^{179}, Tyr^{180}, Pro^{406}, Ala^{407}, Pro^{408}, Arg^{409}, Thr^{410}, Leu^{412}, Thr^{416}, Leu^{417}, Glu^{418}, Pro^{419}, Phe^{422}, Asn^{557}, Asn^{559}, Arg^{560}
			E-channel proteins	-7.43	3.55	Ile^{46}, Leu^{51}, Pro^{54}, Phe^{56}, Tyr^{57}
			Spike Open Protein	-7.35	4.1	Gly^{908}, Ile^{909}, Gly^{910}, Gln^{1036}, Ser^{1037}, Lys^{1038}, Arg^{1039}, Val^{1040}, Asp^{1041}, Tyr^{1047}, His^{1048}, Glu^{1092}, Gly^{1093}, Arg^{1107}, Asn^{1108}
			Spike Close Protein	-7.09	6.32	Phe^{329}, Pro^{330}, Asn^{331}, Ile^{332}, Thr^{333}, Leu^{335}, Val^{362}, Asp^{364}, Gly^{526}, Pro^{527}, Lys^{528}, Lys^{529}, Ser^{530}, Gln^{580}
2	(1S,4R,5R)-3-[3-(3,4-Dihydroxyphenyl)prop-2-enoyloxy]-1,4,5-trihydroxycyclohexane-1-carboxylic acid	60074140	Main Protease (MPro)	-8.01	1.36	Arg^{131}, Lys^{137}, Asp^{197}, Thr^{198}, Thr^{199}, Tyr^{239}, Leu^{271}, Leu^{272}, Gly^{275}, Met^{276}, Ala^{285}, Leu^{286}, Leu^{287}, Asp^{289}, Glu^{290}
			Papain like protease (PLPro)	-9.23	0.17	Arg^{166}, Ser^{170}, Val^{202}, Glu^{203}, Met^{206}, Tyr^{207}, Met^{208}
			RNA-dependent RNA polymerase (RdRP)	-8.29	0.85	Val^{588}, Ile^{589}, Gly^{590}, Thr^{591}, Ser^{592}, Lys^{593}, Phe^{594}, Leu^{758}, Gln^{815}, Pro^{830}, Tyr^{831}, Pro^{832}, Asp^{865}, Pro^{868}, Trp^{916}
			Helicase	-7.95	1.495	Pro^{175}, Asn^{177}, Asn^{179}, Tyr^{180}, Leu^{405}, Pro^{406}, Pro^{408}, Arg^{409}, Thr^{410}, Leu^{412}, Thr^{413}, Leu^{417}, Pro^{419}, Phe^{422}, Asn^{557}, Asn^{559}, Arg^{560}
			E-channel proteins	-7.69	2.33	No binding site was found
			Spike Open Protein	-7.21	5.15	Met^{740}, Tyr^{741}, Ile^{742}, Gly^{744}, Asn^{856}, Val^{963}, Lys^{964}, Leu^{966}, Ser^{967}, Ser^{975}, Val^{976}, Leu^{977}, Asn^{978}, Arg^{1000}

Sr.	Ligands	PubChem ID	Proteins	Binding energy	Dissociation Constant	Active site residues
				(kcal/mol)	(µM)	
			Spike Close Protein	-6.84	9.7	Arg^{355}, Pro^{426}, Asp^{428}, Phe^{429}, Thr^{430}, Lys^{462}, Pro^{463}, Phe^{464}, Glu^{465}, Ser^{514}, Phe^{515}, Glu^{516}, Leu^{517}
**3**	1,2,4-Cyclopentanetrione	534963	Main Protease (MPro)	-6.25	26.05	Thr^{25}, Thr^{26}, Leu^{27}, His^{41}, Cys^{44}, Thr^{45}, Ser^{46}, Met^{49}, Asn^{142}, Gly^{143}, Cys^{145}, His^{164}, Met^{165}, Glu^{166}, Arg^{188}, Gln^{189}
			Papain like protease (PLPro)	-8.2	0.97	Phe^{69}, His^{73}, Thr^{74}, Thr^{75}, Asp^{76}, Phe^{79}, Arg^{82}, Tyr^{154}, Tyr^{171}, Gln^{174}, His^{175}
			RNA-dependent RNA polymerase (RdRP)	-6.02	38.86	Ala^{46}, Lys^{47}, Tyr^{129}, His^{133}, Asp^{135}, Asn^{705}, Ala^{706}, Ser^{709}, Thr^{710}, Ala^{771}, Ser^{772}, Gln^{773}, Gly^{774}, Ser^{778}, Lys^{780}, Asn^{781}, Ser^{784}
			Helicase	-6.68	12.67	Ala^{4}, Cys^{5}, Val^{6}, Arg^{15}, Arg^{21}, Arg^{22}, Pro^{23}, Phe^{24}, Arg^{129}, Leu^{132}, Phe^{133}, Glu^{136}, Pro^{234}, Leu^{235}, Ser^{236}
			E-channel proteins	-6.72	11.8	Leu^{19}, Ala^{22}, Phe^{23}
			Spike Open Protein	-5.87	50.05	Ile^{788}, Tyr^{789}, Lys^{790}, Thr^{791}, Pro^{792}, Pro^{793}, Lys^{795}, Leu^{806}, Pro^{807}, Pro^{809}, Lys^{814}, Gln^{872}, Ser^{875}, Ala^{879}
			Spike Close Protein	-7.31	4.41	Glu^{96}, Ile^{101}, Arg^{102}, Gly^{103}, Trp^{104}, Ile^{119}, Asn^{121}, Val^{126}, Ile^{128}, Tyr^{170}, Ser^{172}, Arg^{190}, Phe^{192}, Phe^{194}, Ile^{203}, Ser^{205}, His^{207}, Leu^{226}, Val^{227}
**4**	11,14,17-Eicosatrienoic acid	5312529	Main Protease (MPro)	-4.53	477.24	Thr^{25}, Thr^{26}, Leu^{27}, His^{41}, Cys^{44}, Thr^{45}, Ser^{46}, Met^{49}, Asn^{142}, Gly^{143}, Cys^{145}, His^{164}, Met^{165}, Glu^{166}, Leu^{167}, Pro^{168}, Arg^{188}, Gln^{189}, Thr^{190}, Gln^{192}
			Papain like protease (PLPro)	-6.74	11.45	Arg^{166}, Tyr^{268}
			RNA-dependent RNA polymerase (RdRP)	-4.79	308.76	Phe^{35}, Asp^{36}, Ile^{37}, Tyr^{38}, Asn^{39}, Val^{42}, Phe^{48}, Lys^{50}, Lys^{73}, Thr^{206}, Asp^{208}, Asn^{209}, Tyr^{217}, Asp^{218}, Asp^{221}, Asn^{713}
			Helicase	-4.99	220.3	Ala^{4}, Val^{6}, Arg^{15}, Arg^{21}, Arg^{22}, Pro^{23}, Phe^{24}, Arg^{129}, Leu^{132}, Phe^{133}, Glu^{136}, Pro^{234}, Leu^{235}
			E-channel proteins	-5.74	61.81	Leu^{19}, Ala^{22}, Phe^{23}

Sr.	Ligands	PubChem ID	Proteins	Binding energy (kcal/mol)	Dissociation Constant (µM)	Active site residues
			Spike Open Protein	-3.95	1280.98	Pro^{1079}, Phe^{1089}, Pro^{1090}, Arg^{1107}, Phe^{1121}, Ser^{1123}, Gly^{1124}, Asn^{1125}, Val^{1128}, Val^{1129}, Ile^{1130}
			Spike Close Protein	-4.95	236.08	Trp^{104}, Ile^{119}, Asn^{121}, Val^{126}, Ile^{128}, Phe^{168}, Tyr^{170}, Ser^{172}, Phe^{192}, Phe^{194}, Ile^{203}, Leu^{226}, Val^{227}, Asp^{228}, Leu^{229}
5	11-Eicosenoic acid	5282768	Main Protease (MPro)	-4.11	979.48	Trp^{218}, Phe^{219}, Leu^{220}, Asn^{221}, Arg^{222}, Phe^{223}, Ser^{267}, Glu^{270}, Leu^{271}, Asn^{274}, Gly^{275}, Met^{276}, Asn^{277}, Arg^{279}
			Papain like protease (PLPro)	-6.42	19.65	Asp^{164}, Arg^{166}, Met^{208}, Ser^{245}, Ala^{246}, Pro^{247}, Pro^{248}, Tyr^{268}, Tyr^{273}, Thr^{301}
			RNA-dependent RNA polymerase (RdRP)	-4.11	969.61	Phe^{441}, Lys^{545}, Tyr^{546}, Ala^{547}, Ile^{548}, Arg^{836}, Ala^{840}, Phe^{843}, Val^{844}, Asp^{845}, Arg^{858}, Leu^{862}
			Helicase	-4.76	322.608	Asn^{179}, Pro^{406}, Ala^{407}, Pro^{408}, Arg^{409}, Thr^{410}, Leu^{412}, Gly^{415}, Thr^{416}, Leu^{417}, Phe^{422}, His^{554}, Asn^{557}, Asn^{559}, Arg^{560}
			E-channel proteins	-6.07	35.41	No binding site was found
			Spike Open Protein	-4.01	1155.66	Ile^{119}, Val^{126}, Ile^{128}, Phe^{168}, Tyr^{170}, Ser^{172}, Ile^{203}, Leu^{226}, Val^{227}, Asp^{228}, Leu^{229}, Pro^{230}
			Spike Close Protein	-6.21	28.2	Glu^{96}, Asn^{99}, Ile^{101}, Arg^{102}, Trp^{104}, Ile^{119}, Asn^{121}, Val^{126}, Ile^{128}, Phe^{168}, Tyr^{170}, Ser^{172}, Arg^{190}, Phe^{192}, Phe^{194}, Ile^{203}, Leu^{226}, Val^{227}, Leu^{229}
6	13,16-Docosadienoic acid	5282807	Main Protease (MPro)	-4.08	1026.88	Trp^{218}, Phe^{219}, Leu^{220}, Asn^{221}, Phe^{223}, Glu^{270}, Leu^{271}, Asn^{274}, Gly^{275}, Asn^{277}, Arg^{279}
			Papain like protease (PLPro)	-6.26	25.61	Lys^{157}, Glu^{161}, Leu^{162}, Gly^{163}, Asp^{164}, Val^{165}, Pro^{247}, Pro^{248}, Tyr^{264}, Asn^{267}, Tyr^{268}, Gln^{269}, Tyr^{273}
			RNA-dependent RNA polymerase (RdRP)	-5.03	204.88	Ser^{255}, Tyr^{265}, Ile^{266}, Lys^{267}, Trp^{268}, Leu^{270}, Val^{320}, Phe^{321}, Pro^{322}, Pro^{323}, Thr^{324}, Leu^{387}, Leu^{388}, Leu^{389}, Asp^{390}, Thr^{394}, Cys^{395}, Phe^{396}, Ser^{397}

Sr.	Ligands	PubChem ID	Proteins	Binding energy (kcal/mol)	Dissociation Constant (µM)	Active site residues
6	13,16-Docosadienoic acid	[illegible]	Helicase	-4.34	655.449	Asp^{328}, Lys^{329}, Cys^{330}, Ser^{331}, Arg^{332}, Lys^{345}, Phe^{346}, Lys^{347}, Asn^{349}, Ser^{350}, Thr^{351}, Leu^{352}, Glu^{353}, Gln^{354}
			E-channel proteins	-6.28	24.84	No binding site was found
			Spike Open Protein	-3.64	2161.61	Met^{740}, Tyr^{741}, Ile^{742}, Cys^{743}, Gly^{744}, Asn^{856}, Val^{963}, Lys^{964}, Leu^{966}, Ser^{967}, Ser^{975}, Val^{976}, Leu^{977}, Asn^{978}, Arg^{1000}
			Spike Close Protein	-4.5	500.33	Leu^{335}, Cys^{336}, Phe^{338}, Gly^{339}, Phe^{342}, Asn^{343}, Val^{362}, Ala^{363}, Asp^{364}, Val^{367}, Leu^{368}, Ser^{371}, Ser^{373}, Phe^{374}, Trp^{436}, Arg^{509}
7	18,ß-Glycyrrhetinic acid	404621453	Main Protease (MPro)	-9.19	0.35	Lys^{5}, Arg^{131}, Lys^{137}, Asp^{197}, Thr^{199}, Tyr^{237}, Tyr^{239}, Leu^{272}, Leu^{286}, Leu^{287}, Glu^{288}, Asp^{289}, Glu^{290}
			Papain like protease (PLPro)	-8.71	0.41	No binding site was found
			RNA-dependent RNA polymerase (RdRP)	-8.14	1.07	Arg^{249}, Thr^{252}, Ser^{255}, Tyr^{265}, Thr^{319}, Val^{320}, Phe^{321}, Pro^{323}, Arg^{349}, Thr^{394}, Cys^{395}, Phe^{396}, Tyr^{456}, Arg^{457}, Asn^{459}, Leu^{460}, Pro^{461}, Pro^{677}
			Helicase	-9.91	0.054	Ala^{4}, Val^{6}, Arg^{15}, Arg^{21}, Arg^{22}, Pro^{23}, Phe^{24}, Arg^{129}, Leu^{132}, Phe^{133}, Glu^{136}, Pro^{234}, Leu^{235}, Ser^{236}
			E-channel proteins	-9.72	0.07	No binding site was found
			Spike Open Protein	-7.99	1.38	His^{1101}, Trp^{1102}, Phe^{1103}, Ile^{1114}, Ile^{1115}, Asn^{1135}, Thr^{1136}, Val^{1137}, Tyr^{1138}, Asp^{1139}, Gln^{1142}, Pro^{1143}
			Spike Close Protein	-8.08	1.2	Tyr^{38}, Asp^{40}, Lys^{41}, Val^{42}, Phe^{43}, Arg^{44}, Glu^{224}, Pro^{225}, Tyr^{279}, Gly^{283}
			Main Protease (MPro)	-4.55	462.17	Arg^{4}, Lys^{5}, Met^{6}, Ala^{7}, Phe^{8}, Pro^{9}, Gln^{127}, Phe^{291}, Asp^{295}, Arg^{298}, Gln^{299}
			Papain like protease (PLPro)	-4.86	276.21	No binding site was found
			RNA-dependent RNA polymerase (RdRP)	-4.39	607.51	Lys^{593}, Phe^{594}, Gln^{815}, Pro^{830}, Tyr^{831}, Pro^{832}, Ile^{864}, Asp^{865}, Pro^{868}, Trp^{916}

Sr.	Ligands	PubChem ID	Proteins	Binding energy (kcal/mol)	Dissociation Constant (µM)	Active site residues
**8**	1-Octen-3-ol	18827	Helicase	-4.47	525.43	Gln^{281}, Asp^{401}, Pro^{402}, Ala^{403}, Pro^{419}, Lys^{430}, Pro^{434}, Phe^{437}, Tyr^{457}, Asp^{458}, Asn^{459}, Asn^{559}
			E-channel proteins	-4.5	503.72	No binding site was found
			Spike Open Protein	-4.16	900.21	Val^{729}, Ser^{730}, Met^{731}, Thr^{732}, Lys^{733}, Gln^{774}, Thr^{778}, Phe^{782}, Leu^{861}, Pro^{862}, Pro^{863}, Leu^{865}, Asp^{867}, Ile^{870}, Ala^{1056}, Pro^{1057}, His^{1058}, Gly^{1059}
			Spike Close Protein	-4.15	906.31	Met^{740}, Tyr^{741}, Ile^{742}, Cys^{743}, Gly^{744}, Asn^{856}, Leu^{966}, Ser^{975}, Val^{976}, Leu^{977}, Asn^{978}, Arg^{1000}
**9**	2,5-dihydroxyterephthalic acid	69131	Main Protease (MPro)	-7.22	5.09	Met^{6}, Ala^{7}, Phe^{8}, Pro^{9}, Gln^{127}, Phe^{291}, Asp^{295}, Arg^{298}, Gln^{299}, Gly^{302}, Val^{303}
			Papain like protease (PLPro)	-7.22	5.14	Thr^{74}, Thr^{75}, Asp^{76}, Tyr^{154}, Asn^{156}, Tyr^{171}, Gln^{174}, His^{175}
			RNA-dependent RNA polymerase (RdRP)	-6.49	17.58	Gly^{616}, Trp^{617}, Asp^{618}, Asp^{761}, Ala^{762}, Lys^{798}, Cys^{799}, Trp^{800}, Glu^{811}, Phe^{812}, Cys^{813}, Ser^{814}
			Helicase	-6.3	24.305	Val^{6}, Arg^{21}, Arg^{22}, Pro^{23}, Phe^{24}, Arg^{129}, Leu^{132}, Phe^{133}, Glu^{136}, Leu^{235}
			E-channel proteins	-5.95	43.44	Leu^{51}, Pro^{54}, Phe^{56}, Tyr^{57}
			Spike Open Protein	-6.53	16.29	Arg^{403}, Tyr^{453}, Ser^{494}, Tyr^{495}, Gly^{496}, Phe^{497}, Asn^{501}, Tyr^{505}
			Spike Close Protein	-6.37	21.42	Tyr^{38}, Asp^{40}, Lys^{41}, Val^{42}, Phe^{43}, Arg^{44}, Glu^{224}, Pro^{225}, Tyr^{279}, Asn^{282}, Gly^{283}, Thr^{284}
**10**	2-Hydroxy-5-sulfobenzoic acid	7322	Main Protease (MPro)	-6.44	19.03	Phe^{8}, Val^{104}, Ile^{106}, Gln^{110}, Thr^{111}, Asn^{151}, Asp^{153}, Ser^{158}, Thr^{292}, Phe^{294}, Asp^{295}, Arg^{298}
			Papain like protease (PLPro)	-7.05	6.76	Arg^{166}, Ser^{170}, Val^{202}, Glu^{203}, Met^{206}, Tyr^{207}, Met^{208}
			RNA-dependent RNA polymerase (RdRP)	-5.91	46.71	Ala^{383}, Ser^{384}, Gly^{385}, Asn^{386}
			Helicase	-6.74	11.546	Gln^{281}, Asp^{401}, Pro^{402}, Ala^{403}, Pro^{419}, Met^{429}, Lys^{430}, Gly^{433}, Pro^{434}, Tyr^{457}, Asp^{458}, Asn^{459}
			E-channel proteins	-5.77	59.26	Pro^{54}, Phe^{56}, Tyr^{57}, Ser^{60}

Sr.	Ligands	PubChem ID	Proteins	Binding energy (kcal/mol)	Dissociation Constant (µM)	Active site residues
			Spike Open Protein	-5.08	189.89	Ser^{704}, Val^{705}, Ala^{706}, Tyr^{707}, Ser^{708}, Ser^{711}, Ile^{712}, Ala^{713}, Asn^{1074}
			Spike Close Protein	-6.42	19.68	Met^{740}, Tyr^{741}, Ile^{742}, Cys^{743}, Gly^{744}, Asp^{745}, Phe^{855}, Asn^{856}, Leu^{966}, Leu^{977}, Asn^{978}, Arg^{1000}
**11**	2-Hydroxycinnamic acid	637540	Main Protease (MPro)	-6.47	18.12	Phe^{3}, Met^{6}, Ala^{7}, Phe^{8}, Pro^{9}, Gln^{127}, Phe^{291}, Asp^{295}, Val^{296}, Arg^{298}, Gln^{299}, Val^{303}, Thr^{304}
			Papain like protease (PLPro)	-6.71	12.15	No binding site was found
			RNA-dependent RNA polymerase (RdRP)	-6.15	30.84	Lys^{411}, Pro^{412}, Gly^{413}, Asn^{414}, Tyr^{546}, Val^{844}, Asp^{845}, Asp^{846}cser^{15}cgln^{19}dmet^{87}
			Helicase	-6.32	23.262	Pro^{406}, Ala^{407}, Arg^{409}, Leu^{412}, Thr^{416}, Leu^{417}, Pro^{419}, Phe^{422}, Asn^{557}, Asn^{559}, Arg^{560}
			E-channel proteins	-6.27	25.52	No binding site was found
			Spike Open Protein	-5.96	42.71	Ser^{730}, Met^{731}, Thr^{732}, Lys^{733}, Gln^{774}, Val^{860}, Leu^{861}, Pro^{862}, Pro^{863}, Asp^{867}, Ile^{870}, Ala^{1056}, Pro^{1057}, His^{1058}, Gly^{1059}
			Spike Close Protein	-6.26	25.78	Val^{382}, Leu^{390}, Cys^{391}, Thr^{393}, Leu^{517}, Leu^{518}, His^{519}, Ala^{520}, Ala^{522}, Asn^{544}, Gly^{545}, Phe^{565}
**12**	2R,3S-Catechin	9064	Main Protease (MPro)	-8.49	0.6	Met^{6}, Ala^{7}, Phe^{8}, Pro^{9}, Gln^{127}, Asp^{295}, Arg^{298}, Gln^{299}, Gly^{302}, Val^{303}, Thr^{304}
			Papain like protease (PLPro)	-8.11	1.14	Arg^{166}, Glu^{167}, Ser^{170}, Val^{202}, Glu^{203}, Met^{206}, Tyr^{207}, Met^{208}, Lys^{232}
			RNA-dependent RNA polymerase (RdRP)	-7.25	4.84	Phe^{441}, Lys^{545}, Tyr^{546}, Ala^{547}, Ile^{548}, Ser^{549}, Arg^{555}, Arg^{836}, Ala^{840}, Val^{844}, Asp^{845}, Arg^{858}, Leu^{862}
			Helicase	-7.84	1.794	Pro^{406}, Ala^{407}, Pro^{408}, Arg^{409}, Leu^{412}, Thr^{413}, Lys^{414}, Gly^{415}, Thr^{416}, Leu^{417}, Phe^{422}, His^{554}
			E-channel proteins	-6.78	10.79	Ala^{32}, Ile^{33}, Ala^{36}

Sr.	Ligands	PubChem ID	Proteins	Binding energy	Dissociation Constant	Active site residues
				(kcal/mol)	(µM)	
			Spike Open Protein	-6.89	8.87	Met^{740}, Tyr^{741}, Ile^{742}, Cys^{743}, Gly^{744}, Asn^{856}, Val^{963}, Lys^{964}, Leu^{966}, Ser^{967}, Ser^{975}, Val^{976}, Leu^{977}, Asn^{978}, Arg^{1000}
			Spike Close Protein	-6.95	8.02	Met^{740}, Tyr^{741}, Ile^{742}, Cys^{743}, Gly^{744}, Asn^{856}, Val^{963}, Lys^{964}, Leu^{966}, Ser^{967}, Ser^{975}, Val^{976}, Leu^{977}, Asn^{978}, Arg^{1000}
13	2S,3R-Catechin	73160	Main Protease (MPro)	-8.09	1.18	Met^{6}, Ala^{7}, Phe^{8}, Pro^{9}, Ser^{113}, Gln^{127}, Asp^{295}, Arg^{298}, Gln^{299}, Gly^{302}, Val^{303}, Thr^{304}
			Papain like protease (PLPro)	-9.26	0.16	Thr^{74}, Thr^{75}, Asp^{76}, Tyr^{154}, Tyr^{171}, Gln^{174}, His^{175}
			RNA-dependent RNA polymerase (RdRP)	-7.06	6.72	Lys^{593}, Phe^{594}, Cys^{813}, Ser^{814}, Gln^{815}, Pro^{830}, Tyr^{831}, Pro^{832}, Arg^{836}, Asp^{865}, Pro^{868}, Trp^{916}
			Helicase	-7.54	2.972	Asn^{179}, Pro^{406}, Pro^{408}, Arg^{409}, Thr^{410}, Leu^{412}, Gly^{415}, Thr^{416}, Leu^{417}, Pro^{419}, Phe^{422}, Asn^{557}, Asn^{559}, Arg^{560}
			E-channel proteins	-7.74	2.11	Leu^{51}, Pro^{54}, Phe^{56}, Tyr^{57}, Ser^{60}, Arg^{61}
			Spike Open Protein	-6.92	8.45	Met^{740}, Tyr^{741}, Ile^{742}, Cys^{743}, Gly^{744}, Asn^{856}, Val^{963}, Leu^{966}, Ser^{967}, Ser^{975}, Val^{976}, Leu^{977}, Asn^{978}, Arg^{1000}
			Spike Close Protein	-7.24	4.94	Met^{740}, Tyr^{741}, Ile^{742}, Cys^{743}, Gly^{744}, Asn^{856}, Val^{963}, Leu^{966}, Ser^{967}, Ser^{975}, Val^{976}, Leu^{977}, Asn^{978}, Arg^{1000}
14	3,4-Dihydroxybenzoic acid	72	Main Protease (MPro)	-5.94	44.4	Met^{6}, Ala^{7}, Phe^{8}, Pro^{9}, Ser^{113}, Gln^{127}, Asp^{295}, Arg^{298}, Gln^{299}, Val^{303}, Thr^{304}
			Papain like protease (PLPro)	-6.85	9.61	No binding site was found
			RNA-dependent RNA polymerase (RdRP)	-6.13	32.22	Gly^{616}, Trp^{617}, Asp^{618}, Asp^{761}, Ala^{762}, Val^{763}, Lys^{798}, Cys^{799}, Trp^{800}, Glu^{811}, Phe^{812}, Cys^{813}, Ser^{814}
			Helicase	-6.65	13.35	Tyr^{120}, Ala^{123}, Asn^{124}, Ala^{135}, Leu^{138}, Thr^{380}, Asn^{381}, Tyr^{382}, Arg^{409}, Leu^{417}, Tyr^{421}, Phe^{422}
			E-channel proteins	-5.47	98.32	Leu^{51}, Pro^{54}, Tyr^{57}
			Spike Open Protein	-5.8	55.76	Met^{740}, Tyr^{741}, Ile^{742}, Cys^{743}, Gly^{744}, Leu^{966}, Ser^{967}, Ser^{975}, Val^{976}, Leu^{977}, Asn^{978}, Arg^{1000}

Sr.	Ligands	PubChem ID	Proteins	Binding energy (kcal/mol)	Dissociation Constant (µM)	Active site residues
			Spike Close Protein	-6.22	27.54	Tyr^{38}, Asp^{40}, Lys^{41}, Val^{42}, Phe^{43}, Arg^{44}, Glu^{224}, Pro^{225}, Asn^{282}, Gly^{283}, Thr^{284}
15	3,4-Dimethoxybenzoic acid	7121	Main Protease (MPro)	-6.29	24.43	Met^{6}, Ala^{7}, Phe^{8}, Pro^{9}, Thr^{111}, Gln^{127}, Phe^{291}, Asp^{295}, Arg^{298}, Gln^{299}, Gly^{302}, Val^{303}
			Papain like protease (PLPro)	-6.06	36.02	Arg^{166}, Ser^{170}, Val^{202}, Glu^{203}, Met^{206}, Tyr^{207}, Met^{208}
			RNA-dependent RNA polymerase (RdRP)	-5.35	120.8	Gly^{616}, Trp^{617}, Asp^{618}, Asp^{761}, Lys^{798}, Cys^{799}, Trp^{800}, Glu^{811}, Phe^{812}, Cys^{813}, Ser^{814}
			Helicase	-5.71	64.911	Ala^{4}, Cys^{5}, Val^{6}, Arg^{15}, Arg^{21}, Arg^{22}, Pro^{23}, Phe^{24}, Arg^{129}, Leu^{132}, Phe^{133}, Glu^{136}, Leu^{235}
			E-channel proteins	-5.57	82.35	Leu^{28}, Leu^{31}, Ala^{32}, Thr^{35}, Cys^{40}
			Spike Open Protein	-5.25	141.33	Met^{740}, Tyr^{741}, Gly^{744}, Leu^{966}, Ser^{967}, Ser^{975}, Val^{976}, Leu^{977}, Asn^{978}, Arg^{1000}
			Spike Close Protein	-5.44	103.25	Leu^{335}, Cys^{336}, Pro^{337}, Phe^{338}, Gly^{339}, Phe^{342}, Asn^{343}, Val^{362}, Ala^{363}, Asp^{364}, Val^{367}, Leu^{368}
16	3-Acetoxy-4,5-dihydroxy	129855414	Main Protease (MPro)	-6.99	7.58	Arg^{40}, Pro^{52}, Asn^{53}, Tyr^{54}, Glu^{55}, Asp^{56}, Met^{82}, Asn^{84}, Cys^{85}, Thr^{175}, Gly^{179}, Asn^{180}, Phe^{181}, Phe^{185}, Val^{186}, Asp^{187}, Arg^{188}
	-3-prenyldihydrostilbene		Papain like protease (PLPro)	-9	0.25	Lys^{157}, Glu^{161}, Leu^{162}, Gly^{163}, Asp^{164}, Glu^{167}, Pro^{248}, Tyr^{264}, Tyr^{268}, Gln^{269}, Tyr^{273}, Thr^{301}
			RNA-dependent RNA polymerase (RdRP)	-7.11	6.19	Arg^{33}, Phe^{35}, Ile^{37}, Asn^{39}, Val^{42}, Phe^{48}, Leu^{49}, Lys^{50}, Lys^{73}, Thr^{123}, Asp^{208}, Asn^{209}, Tyr^{217}, Asp^{218}, Asn^{713}
			Helicase	-7.23	5.007	Pro^{175}, Asn^{177}, Asn^{179}, Tyr^{180}, Pro^{406}, Ala^{407}, Pro^{408}, Arg^{409}, Thr^{410}, Leu^{412}, Thr^{413}, Gly^{415}, Thr^{416}, Leu^{417}, Phe^{422}, His^{554}, Asn^{557}, Asn^{559}, Arg^{560}
			E-channel proteins	-8.62	0.48	No binding site was found
			Spike Open Protein	-7.63	2.54	Ile^{105}, Leu^{118}, Ile^{119}, Val^{120}, Asn^{122}, Val^{127}, Ile^{128}, Lys^{129}, Phe^{133}, Gln^{134}, Phe^{135}, Cys^{136}, Pro^{139}, Phe^{140}, Leu^{141}, Leu^{241}

Sr.	Ligands	PubChem ID	Proteins	Binding energy (kcal/mol)	Dissociation Constant (µM)	Active site residues
			Spike Close Protein	-7.48	3.27	Tyr^{38}, Asp^{40}, Lys^{41}, Val^{42}, Phe^{43}, Arg^{44}, Glu^{224}, Pro^{225}, Tyr^{279}, Asn^{282}, Gly^{283}, Thr^{284}
**17**	3-Deazaneplanocin A	73087	Main Protease (MPro)	-6.46	18.46	Thr^{24}, Thr^{25}, Thr^{26}, His^{41}, Cys^{44}, Thr^{45}, Ser^{46}, Met^{49}, His^{164}, Met^{165}, Glu^{166}, Gln^{189}
			Papain like protease (PLPro)	-7.62	2.61	Tyr^{268}
			RNA-dependent RNA polymerase (RdRP)	-6.43	19.32	Glu^{136}, Gly^{137}, Asn^{138}, Cys^{139}, Asp^{140}, Lys^{143}, Phe^{157}, Asn^{158}, Lys^{159}, Lys^{160}, Asp^{161}, Trp^{162}, Tyr^{163}
			Helicase	-6.91	8.608	Pro^{406}, Arg^{409}, Leu^{412}, Thr^{416}, Leu^{417}, Glu^{418}, Pro^{419}, Phe^{422}, His^{554}, Asn^{557}, Asn^{559}, Arg^{560}
			E-channel proteins	-5.78	57.77	Val^{29}, Ala^{32}, Ile^{33}, Ala^{36}, Arg^{38}
			Spike Open Protein	-5.78	58.46	Tyr^{396}, Pro^{426}, Asp^{428}, Phe^{429}, Thr^{430}, Phe^{464}, Ser^{514}, Phe^{515}, Glu^{516}, Leu^{517}, Leu^{518}
			Spike Close Protein	-6.27	25.4	Phe^{329}, Pro^{330}, Asn^{331}, Ile^{332}, Thr^{333}, Val^{362}, Gly^{526}, Pro^{527}, Lys^{528}, Lys^{529}, Ser^{530}
**18**	4,7,10,13,16-Docosapentaenoic acid	5282848	Main Protease (MPro)	-4.86	272.04	Trp^{218}, Phe^{219}, Leu^{220}, Asn^{221}, Phe^{223}, Ser^{267}, Glu^{270}, Leu^{271}, Asn^{274}, Gly^{275}, Met^{276}, Asn^{277}, Arg^{279}
			Papain like protease (PLPro)	-7.77	2.03	Lys^{157}, Leu^{162}, Gly^{163}, Asp^{164}, Arg^{166}, Met^{208}, Pro^{247}, Pro^{248}, Tyr^{264}, Tyr^{268}, Gln^{269}, Tyr^{273}, Thr^{301}
			RNA-dependent RNA polymerase (RdRP)	-5.11	180.21	Lys^{426}, Gly^{427}, Phe^{428}, Phe^{429}, Lys^{430}, Ser^{433}, Ser^{434}, Val^{435}, Glu^{876}, Asp^{879}, His^{882}, Leu^{883}, Gln^{886}
			Helicase	-5.85	51.772	Asn^{179}, Pro^{406}, Ala^{407}, Pro^{408}, Arg^{409}, Thr^{410}, Leu^{412}, Gly^{415}, Thr^{416}, Leu^{417}, Phe^{422}, Asp^{534}, His^{554}, Asn^{557}, Asn^{559}, Arg^{560}
			E-channel proteins	-7.04	6.89	No binding site was found
			Spike Open Protein	-5.12	177.5	Trp^{104}, Ile^{119}, Asn^{121}, Val^{126}, Ile^{128}, Tyr^{170}, Ser^{172}, Arg^{190}, Phe^{192}, Phe^{194}, Ile^{203}, Ser^{205}, His^{207}, Leu^{226}, Val^{227}

Sr.	Ligands	PubChem ID	Proteins	Binding energy (kcal/mol)	Dissociation Constant (µM)	Active site residues
			Spike Close Protein	-6.18	29.56	Trp^{104}, Ile^{119}, Asn^{121}, Val^{126}, Ile^{128}, Phe^{168}, Tyr^{170}, Ser^{172}, Arg^{190}, Phe^{192}, Phe^{194}, Ile^{203}, Leu^{226}, Val^{227}, Asp^{228}, Leu^{229}
**19**	4-Hydroxybenzoic acid	135	Main Protease (MPro)	-6.04	37.57	Met^{6}, Ala^{7}, Phe^{8}, Pro^{9}, Thr^{111}, Gln^{127}, Phe^{291}, Asp^{295}, Arg^{298}, Gln^{299}, Val^{303}
			Papain like protease (PLPro)	-6.06	36.08	His^{73}, Thr^{74}, Asp^{76}, Phe^{79}, Arg^{82}, Ala^{153}, Tyr^{154}, Cys^{155}, Asn^{156}, His^{175}
			RNA-dependent RNA polymerase (RdRP)	-5.45	101.18	Gly^{616}, Trp^{617}, Asp^{618}, Asp^{761}, Ala^{762}, Lys^{798}, Cys^{799}, Trp^{800}, Glu^{811}, Phe^{812}, Cys^{813}, Ser^{814}
			Helicase	-5.97	42.28	Tyr^{120}, Ala^{123}, Asn^{124}, Ala^{135}, Leu^{138}, Thr^{380}, Asn^{381}, Tyr^{382}, Arg^{409}, Leu^{417}, Tyr^{421}, Phe^{422}
			E-channel proteins	-5.42	106.8	Leu^{51}, Pro^{54}, Tyr^{57}
			Spike Open Protein	-5.32	127.07	Met^{740}, Tyr^{741}, Ile^{742}, Cys^{743}, Gly^{744}, Asn^{856}, Leu^{966}, Ser^{967}, Ser^{975}, Val^{976}, Leu^{977}, Asn^{978}, Arg^{1000}
			Spike Close Protein	-5.92	45.69	Tyr^{38}, Asp^{40}, Lys^{41}, Val^{42}, Phe^{43}, Arg^{44}, Glu^{224}, Pro^{225}, Tyr^{279}, Gly^{283}, Thr^{284}
**20**	4-Hydroxycinnamic acid	637542	Main Protease (MPro)	-6.68	12.71	Phe^{3}, Lys^{5}, Met^{6}, Phe^{8}, Pro^{9}, Gln^{127}, Phe^{291}, Asp^{295}, Arg^{298}, Gln^{299}, Gly^{302}, Val^{303}, Thr^{304}
			Papain like protease (PLPro)	-6.83	9.79	His^{73}, Thr^{74}, Asp^{76}, Phe^{79}, Arg^{82}, Ala^{153}, Tyr^{154}, Cys^{155}, Asn^{156}, His^{175}
			RNA-dependent RNA polymerase (RdRP)	-6.33	23.07	Pro^{323}, Thr^{324}, Phe^{326}, Gly^{327}, Tyr^{346}, His^{347}, Phe^{348}, Arg^{349}, Glu^{350}, Phe^{396}, Asn^{628}, Ser^{664}, Val^{675}, Lys^{676}, Pro^{677}basn^{118}
			Helicase	-6.18	29.612	Val^{496}, Leu^{500}, Thr^{501}, Asn^{503}, Trp^{506}, Arg^{507}, Ala^{509}, Leu^{526}, Gly^{527}, Leu^{528}, Tyr^{543}, Ile^{545}, Leu^{573}
			E-channel proteins	-6.29	24.51	No binding site was found
			Spike Open Protein	-6.49	17.55	Tyr^{38}, Asp^{40}, Lys^{41}, Val^{42}, Phe^{43}, Arg^{44}, Glu^{224}, Pro^{225}, Tyr^{279}, Gly^{283}

Sr.	Ligands	PubChem ID	Proteins	Binding energy (kcal/mol)	Dissociation Constant (µM)	Active site residues
			Spike Close Protein	-6.65	13.31	Tyr^{38}, Asp^{40}, Lys^{41}, Val^{42}, Phe^{43}, Arg^{44}, Glu^{224}, Pro^{225}, Tyr^{279}, Asn^{282}, Gly^{283}, Thr^{284}
**21**	4-o-Methylglycyrrhisoflavone	637542	Main Protease (MPro)	-6.91	8.56	Arg^{131}, Thr^{199}, Ile^{200}, Tyr^{237}, Tyr^{239}, Leu^{271}, Leu^{272}, Gly^{275}, Met^{276}, Ala^{285}, Leu^{286}, Leu^{287}, Asp^{289}, Glu^{290}
			Papain like protease (PLPro)	-8.61	0.49	Pro^{247}, Pro^{248}, Asn^{267}, Tyr^{268}, Gln^{269}
			RNA-dependent RNA polymerase (RdRP)	-7.16	5.65	Arg^{197}, Gly^{230}, Val^{231}, Pro^{232}, Phe^{283}, Asp^{284}, Lys^{288}, Tyr^{289}, Trp^{290}, Asp^{291}, Gln^{292}, Thr^{293}, Tyr^{294}
			Helicase	-7.6	2.682	Ala^{1}, Val^{6}, Arg^{15}, Arg^{21}, Arg^{22}, Pro^{23}, Phe^{24}, Arg^{129}, Leu^{130}, Leu^{132}, Phe^{133}, Glu^{136}, Pro^{234}, Leu^{235}, Ser^{236}
			E-channel proteins	-7.95	1.48	No binding site was found
			Spike Open Protein	-6.58	15.15	Ser^{730}, Thr^{778}, Phe^{823}, Val^{826}, Thr^{827}, Pro^{863}, Asp^{867}, Ile^{870}, Ala^{1056}, Pro^{1057}, His^{1058}, Gly^{1059}
			Spike Close Protein	-6.17	30.17	His^{1101}, Trp^{1102}, Phe^{1103}, Ile^{1114}, Ile^{1115}, Thr^{1136}, Val^{1137}, Tyr^{1138}
**22**	4-o-Methylneobavaisoflavone	637542	Main Protease (MPro)	-7.07	6.6	Arg^{131}, Lys^{137}, Asp^{197}, Thr^{199}, Tyr^{237}, Asn^{238}, Tyr^{239}, Leu^{271}, Leu^{272}, Gly^{275}, Met^{276}, Leu^{286}, Leu^{287}, Asp^{289}
			Papain like protease (PLPro)	-8.99	0.26	Arg^{166}, Ser^{170}, Val^{202}, Glu^{203}, Met^{206}, Met^{208}
			RNA-dependent RNA polymerase (RdRP)	-6.52	16.51	Ser^{255}, Tyr^{265}, Ile^{266}, Lys^{267}, Trp^{268}, Leu^{270}, Val^{320}, Phe^{321}, Pro^{322}, Thr^{324}
			Helicase	-7.84	1.782	Asn^{179}, Tyr^{180}, Pro^{406}, Pro^{408}, Arg^{409}, Thr^{410}, Leu^{412}, Thr^{413}, Leu^{417}, Pro^{419}, Phe^{422}, Asp^{534}, Asn^{557}, Asn^{559}, Arg^{560}
			E-channel proteins	-8.15	1.06	No binding site was found
			Spike Open Protein	-5.89	47.82	Leu^{54}, Phe^{86}, Asn^{87}, Asp^{88}, Asn^{196}, Ile^{197}, Asn^{234}, Ile^{235}, Thr^{236}, Pro^{272}
			Spike Close Protein	-6.52	16.68	Trp^{886}, Gly^{889}, Ala^{890}, Tyr^{904}, Ser^{1030}, Glu^{1031}, Leu^{1034}, Gly^{1035}, Gln^{1036}, Ser^{1037}, Lys^{1038}, Arg^{1039}

Sr.	Ligands	PubChem ID	Proteins	Binding energy (kcal/mol)	Dissociation Constant (µM)	Active site residues
**23**	6,9,12-Octadecatrienoic acid	5353640	Main Protease (MPro)	-4.45	544.39	Trp^{218}, Phe^{219}, Leu^{220}, Asn^{221}, Arg^{222}, Phe^{223}, Ser^{267}, Glu^{270}, Leu^{271}, Asn^{274}, Gly^{275}, Met^{276}, Asn^{277}
			Papain like protease (PLPro)	-4.85	276.68	Asp^{164}, Arg^{166}, Met^{208}, Pro^{247}, Pro^{248}, Tyr^{268}
			RNA-dependent RNA polymerase (RdRP)	-4.88	266.59	Phe^{441}, Tyr^{546}, Ala^{547}, Ile^{548}, Arg^{836}, Ile^{837}, Ala^{840}, Phe^{843}, Val^{844}, Asp^{845}, Arg^{858}, Ser^{861}, Leu^{862}, Asp^{865}
			Helicase	-5.63	74.42	Ala^{4}, Val^{6}, Arg^{21}, Arg^{22}, Pro^{23}, Phe^{24}, Glu^{128}, Arg^{129}, Leu^{132}, Phe^{133}, Glu^{136}, Pro^{234}, Leu^{235}, Ser^{236}, Pro^{238}
			E-channel proteins	-6.26	25.74	Phe^{26}, Val^{29}, Thr^{30}
			Spike Open Protein	-3.78	1698.07	His^{66}, Ala^{67}, Ile^{68}, Pro^{82}, Asp^{138}, Pro^{139}, Phe^{140}, Leu^{141}, Gln^{239}, Thr^{240}, Leu^{241}, Leu^{242}, Tyr^{265}
			Spike Close Protein	-5.3	130.33	Trp^{104}, Ile^{119}, Asn^{121}, Val^{126}, Ile^{128}, Phe^{168}, Tyr^{170}, Phe^{192}, Phe^{194}, Ile^{203}, Leu^{226}, Val^{227}, Asp^{228}, Leu^{229}
**24**	7,10 ,13,16-Docosatetraenoic acid	2033	Main Protease (MPro)	-4.64	395.7	Arg^{131}, Thr^{199}, Tyr^{237}, Tyr^{239}, Leu^{271}, Leu^{272}, Gly^{275}, Met^{276}, Leu^{286}, Leu^{287}, Glu^{288}, Asp^{289}
			Papain like protease (PLPro)	-5.61	76.72	Pro^{247}, Pro^{248}, Asn^{267}, Tyr^{268}
			RNA-dependent RNA polymerase (RdRP)	-5.36	117.58	Arg^{583}, Ser^{592}, Phe^{594}, Tyr^{595}, Ser^{861}, Ile^{864}, Asp^{865}, Tyr^{915}, Pro^{918}, Glu^{919}
			Helicase	-5.67	69.327	Ala^{4}, Cys^{5}, Val^{6}, Arg^{21}, Arg^{22}, Pro^{23}, Phe^{24}, Arg^{129}, Leu^{132}, Phe^{133}, Glu^{136}, Pro^{234}, Leu^{235}, Ser^{236}
			E-channel proteins	-6.74	11.57	No binding site was found
			Spike Open Protein	-3.45	2963.8	Leu^{54}, Phe^{86}, Asn^{87}, Asp^{88}, Asn^{196}, Ile^{197}, Asp^{198}, Gly^{199}, Ile^{233}, Asn^{234}, Ile^{235}, Pro^{272}
			Spike Close Protein	-5.22	148.92	Val^{327}, Val^{382}, Ser^{383}, Lys^{386}, Asp^{389}, Leu^{390}, Cys^{391}, Phe^{392}, Thr^{393}, Leu^{517}, Leu^{518}, His^{519}, Ala^{520}, Ala^{522}, Lys^{528}, Asn^{542}, Asn^{544}, Gly^{545}, Leu^{546}, Thr^{547}, Phe^{565}

Sr.	Ligands	PubChem ID	Proteins	Binding energy (kcal/mol)	Dissociation Constant (µM)	Active site residues
**25**	7,10,13,16-Docosatetraenoic acid, methyl ester	5352981	Main Protease (MPro)	-4.43	570.73	Thr^{199}, Tyr^{237}, Tyr^{239}, Leu^{271}, Leu^{272}, Asn^{274}, Gly^{275}, Met^{276}, Asn^{277}, Gly^{278}, Arg^{279}, Ala^{285}, Leu^{286}, Leu^{287}
			Papain like protease (PLPro)	-6.55	15.78	Asp^{164}, Val^{165}, Arg^{166}, Met^{208}, Ser^{245}, Ala^{246}, Pro^{247}, Pro^{248}, Tyr^{264}, Tyr^{268}, Tyr^{273}, Thr^{301}, Asp^{302}
			RNA-dependent RNA polymerase (RdRP)	-5.45	100.67	Val^{335}, Asp^{336}, Val^{338}, Pro^{339}, Phe^{340}, Arg^{365}, Leu^{366}, Leu^{371}
			Helicase	-4.79	309.801	Ala^{4}, Cys^{5}, Val^{6}, Arg^{15}, Arg^{21}, Arg^{22}, Pro^{23}, Phe^{24}, Arg^{129}, Leu^{132}, Phe^{133}, Glu^{136}, Pro^{234}, Leu^{235}, Ser^{236}
			E-channel proteins	-6.57	15.38	Leu^{19}, Phe^{20}, Ala^{22}, Phe^{23}, Val^{24}, Phe^{26}, Leu^{27}, Thr^{30}
			Spike Open Protein	-4.15	910.91	Met^{740}, Tyr^{741}, Ile^{742}, Cys^{743}, Gly^{744}, Phe^{855}, Asn^{856}, Val^{963}, Leu^{966}, Ser^{967}, Ser^{975}, Val^{976}, Leu^{977}, Asn^{978}, Arg^{1000}
			Spike Close Protein	-5.68	69.09	Cys^{336}, Phe^{338}, Gly^{339}, Phe^{342}, Asn^{343}, Asp^{364}, Val^{367}, Leu^{368}, Ser^{371}, Ala^{372}, Ser^{373}, Phe^{374}, Ser^{375}, Trp^{436}, Asn^{437}, Ser^{438}, Asn^{440}, Leu^{441}, Arg^{509}
**26**	8-Prenylmucronulatol	637051	Main Protease (MPro)	-6.99	7.48	Thr^{25}, Leu^{27}, His^{41}, Cys^{44}, Thr^{45}, Ser^{46}, Met^{49}, Leu^{141}, Asn^{142}, Gly^{143}, Ser^{144}, Cys^{145}, His^{164}, Met^{165}, Glu^{166}, Leu^{167}, Pro^{168}, Arg^{188}, Gln^{189}, Thr^{190}, Gln^{192}
			Papain like protease (PLPro)	-9.01	0.25	Pro^{247}, Pro^{248}, Asn^{267}, Tyr^{268}
			RNA-dependent RNA polymerase (RdRP)	-7.24	4.9	Tyr^{32}, Lys^{47}, Tyr^{129}, Ala^{130}, His^{133}, Phe^{134}, Asp^{135}, Asn^{138}, Cys^{139}, Ser^{709}, Thr^{710}, Asp^{711}, Lys^{714}, Gln^{773}, Gly^{774}, Asn^{781}, Ser^{784}
			Helicase	-7.39	3.835	Pro^{175}, Asn^{177}, Asn^{179}, Tyr^{180}, Gln^{404}, Leu^{405}, Pro^{406}, Ala^{407}, Pro^{408}, Arg^{409}, Thr^{410}, Leu^{412}, Thr^{413}, Thr^{416}, Leu^{417}, Phe^{422}, Asn^{557}, Asn^{559}, Arg^{560}
			E-channel proteins	-7.8	1.93	No binding site was found
			Spike Open Protein	-6.91	8.61	Arg^{355}, Tyr^{396}, Pro^{426}, Asp^{428}, Phe^{429}, Thr^{430}, Pro^{463}, Phe^{464}, Glu^{465}, Ser^{514}, Phe^{515}, Glu^{516}, Leu^{517}, Leu^{518}

Sr.	Ligands	PubChem ID	Proteins	Binding energy (kcal/mol)	Dissociation Constant (µM)	Active site residues
			Spike Close Protein	-7.07	6.58	Leu^{335}, Cys^{336}, Pro^{337}, Phe^{338}, Gly^{339}, Glu^{340}, Phe^{342}, Asn^{343}, Asp^{364}, Val^{367}, Leu^{368}, Ser^{371}, Ser^{373}, Phe^{374}, Trp^{436}
**27**	Aconitic acid	309	Main Protease (MPro)	-5.75	61.5	Met^{6}, Ala^{7}, Phe^{8}, Pro^{9}, Asp^{295}, Arg^{298}, Gln^{299}, Gly^{302}, Val^{303}, Thr^{304}
			Papain like protease (PLPro)	-6.53	16.35	No binding site was found
			RNA-dependent RNA polymerase (RdRP)	-5.95	43.73	Gly^{616}, Trp^{617}, Asp^{618}, Asp^{761}, Ala^{762}, Lys^{798}, Cys^{799}, Trp^{800}, Glu^{811}, Phe^{812}, Cys^{813}, Ser^{814}
			Helicase	-5.7	65.904	Gln^{281}, Asp^{401}, Pro^{402}, Ala^{403}, Pro^{419}, Met^{429}, Lys^{430}, Pro^{434}, Tyr^{457}, Asp^{458}, Asn^{459}, Asn^{559}
			E-channel proteins	-5.05	198.41	Leu^{51}, Pro^{54}, Tyr^{57}
			Spike Open Protein	-5.33	124.74	Tyr^{38}, Asp^{40}, Lys^{41}, Val^{42}, Phe^{43}, Arg^{44}, Glu^{224}, Pro^{225}, Tyr^{279}, Gly^{283}, Thr^{284}
			Spike Close Protein	-5.45	100.67	Gly^{744}, Phe^{855}, Asn^{856}, Leu^{966}, Ser^{975}, Val^{976}, Leu^{977}, Asn^{978}, Arg^{1000}
**28**	Agaritine	439517	Main Protease (MPro)	-6.6	14.55	Met^{6}, Ala^{7}, Phe^{8}, Pro^{9}, Gln^{127}, Ile^{152}, Asp^{295}, Arg^{298}, Gln^{299}, Gly^{302}, Val^{303}, Thr^{304}
			Papain like protease (PLPro)	-7.1	6.24	Cys^{155}, Asn^{156}, Lys^{157}, Glu^{161}, Leu^{162}, Glu^{167}, Tyr^{171}
			RNA-dependent RNA polymerase (RdRP)	-6.14	31.36	Lys^{47}, Tyr^{129}, Ala^{130}, His^{133}, Phe^{134}, Asp^{135}, Asn^{138}, Cys^{139}, Asp^{140}, Thr^{141}, Leu^{142}, Asn^{705}, Ala^{706}, Ser^{709}, Lys^{780}, Asn^{781}, Ser^{784}
			Helicase	-6.64	13.623	Val^{6}, Arg^{15}, Arg^{21}, Arg^{22}, Pro^{23}, Phe^{24}, Arg^{129}, Leu^{132}, Phe^{133}, Glu^{136}, Pro^{234}, Leu^{235}
			E-channel proteins	-6.2	28.63	Leu^{28}, Val^{29}, Ala^{32}
			Spike Open Protein	-5.79	57.1	Arg^{355}, Tyr^{396}, Pro^{426}, Asp^{428}, Phe^{429}, Thr^{430}, Phe^{464}, Ser^{514}, Phe^{515}, Glu^{516}

Sr.	Ligands	PubChem ID	Proteins	Binding energy (kcal/mol)	Dissociation Constant (µM)	Active site residues
			Spike Close Protein	-5.76	60.37	Pro^{426}, Asp^{428}, Phe^{429}, Thr^{430}, Pro^{463}, Phe^{464}, Ser^{514}, Phe^{515}, Glu^{516}
**29**	Agnuside	442416	Main Protease (MPro)	-7.28	4.61	Ile^{106}, Gln^{110}, Thr^{111}, Asn^{151}, Asp^{153}, Thr^{292}, Phe^{294}, Asp^{295}, Val^{297}, Arg^{298}, Ser^{301}, Val^{303}
			Papain like protease (PLPro)	-9.07	0.22	Phe^{69}, His^{73}, Thr^{74}, Thr^{75}, Asp^{76}, Phe^{79}, Arg^{82}, Tyr^{154}, Gln^{174}, His^{175}
			RNA-dependent RNA polymerase (RdRP)	-6.17	29.86	Asp^{269}, Leu^{271}, Lys^{272}, Tyr^{273}, Asp^{274}, Phe^{275}, Glu^{277}
			Helicase	-7.38	3.874	Ala^{4}, Val^{6}, Arg^{15}, Arg^{21}, Arg^{22}, Pro^{23}, Phe^{24}, Arg^{129}, Leu^{132}, Phe^{133}, Glu^{136}, Pro^{234}, Leu^{235}
			E-channel proteins	-7.69	2.33	Ile^{46}, Leu^{51}, Pro^{54}, Phe^{56}, Tyr^{57}, Ser^{60}
			Spike Open Protein	-6	40.12	Trp^{104}, Ile^{119}, Asn^{121}, Val^{126}, Ile^{128}, Tyr^{170}, Ser^{172}, Phe^{192}, Phe^{194}, Ile^{203}, Ser^{205}, Leu^{226}, Val^{227}
			Spike Close Protein	-6.14	31.41	Met^{740}, Tyr^{741}, Gly^{744}, Asp^{745}, Phe^{855}, Asn^{856}, Leu^{966}, Ser^{967}, Ser^{975}, Val^{976}, Leu^{977}, Asn^{978}, Arg^{1000}
**30**	Alizarin	6293	Main Protease (MPro)	-7.42	3.65	Val^{104}, Ile^{106}, Gln^{110}, Thr^{111}, Asn^{151}, Asp^{153}, Ser^{158}, Thr^{292}, Phe^{294}, Asp^{295}, Arg^{298}
			Papain like protease (PLPro)	-7.28	4.58	Val^{187}, Gln^{195}, Thr^{197}, Ile^{222}, Pro^{223}, Cys^{224}, Thr^{225}, Ala^{230}, Lys^{232}
			RNA-dependent RNA polymerase (RdRP)	-7.19	5.37	Lys^{411}, Pro^{412}, Gly^{413}, Asn^{414}, Tyr^{546}, Val^{844}, Asp^{845}, Asp^{846}cser^{15}cgln^{18}cgln^{19}
			Helicase	-7.75	2.071	Leu^{405}, Pro^{406}, Arg^{409}, Thr^{410}, Leu^{412}, Leu^{417}, Pro^{419}, Phe^{422}, Asn^{557}, Asn^{559}, Arg^{560}
			E-channel proteins	-7.24	4.92	No binding site was found
			Spike Open Protein	-6.58	15.15	Glu^{554}, Ser^{555}, Asn^{556}, Lys^{557}, Lys^{558}, Phe^{559}, Leu^{560}, Arg^{577}, Leu^{582}, Glu^{583}, Ile^{584}

Sr.	Ligands	PubChem ID	Proteins	Binding energy (kcal/mol)	Dissociation Constant (µM)	Active site residues
			Spike Close Protein	-7.05	6.82	Tyr^{38}, Asp^{40}, Lys^{41}, Val^{42}, Phe^{43}, Arg^{44}, Glu^{224}, Pro^{225}, Tyr^{279}, Gly^{283}
**31**	Alpha pienene	6654	Main Protease (MPro)	-5.37	116.2	Trp^{218}, Phe^{219}, Asn^{221}, Phe^{223}, Ser^{267}, Glu^{270}, Leu^{271}, Asn^{274}, Gly^{275}, Asn^{277}
			Papain like protease (PLPro)	-5.38	113.68	Asp^{164}, Val^{165}, Pro^{247}, Pro^{248}, Tyr^{264}, Asn^{267}, Tyr^{268}, Tyr^{273}, Thr^{301}
			RNA-dependent RNA polymerase (RdRP)	-4.91	250.87	Ser^{592}, Phe^{594}, Tyr^{595}, Ile^{864}, Tyr^{915}
			Helicase	-6.18	29.512	Val^{496}, Phe^{499}, Leu^{500}, Thr^{501}, Asn^{503}, Trp^{506}, Arg^{507}, Ala^{509}, Leu^{526}, Leu^{528}, Tyr^{543}, Ile^{545}, Leu^{573}
			E-channel proteins	-5.87	49.47	No binding site was found
			Spike Open Protein	-5.68	68.86	Tyr^{38}, Asp^{40}, Lys^{41}, Val^{42}, Phe^{43}, Arg^{44}, Glu^{224}, Pro^{225}, Tyr^{279}, Gly^{283}
			Spike Close Protein	-5.9	47.42	Tyr^{38}, Asp^{40}, Lys^{41}, Val^{42}, Phe^{43}, Arg^{44}, Glu^{224}, Pro^{225}, Tyr^{279}, Gly^{283}
**32**	Anhydrosecoisolariciresinol	9974771	Main Protease (MPro)	-6.55	15.83	Arg^{131}, Lys^{137}, Asp^{197}, Thr^{199}, Tyr^{237}, Tyr^{239}, Leu^{271}, Leu^{272}, Gly^{275}, Met^{276}, Ala^{285}, Leu^{286}, Leu^{287}, Asp^{289}, Glu^{290}
			Papain like protease (PLPro)	-7.23	5.02	Lys^{157}, Glu^{161}, Leu^{162}, Gly^{163}, Asp^{164}, Glu^{167}, Tyr^{268}, Gln^{269}
			RNA-dependent RNA polymerase (RdRP)	-6.42	19.78	Asp^{717}, Tyr^{719}, Val^{720}, Asn^{722}, Leu^{723}, Arg^{726}, Glu^{744}, Ala^{747}, Tyr^{748}, Lys^{751}, His^{752}, Tyr^{770}
			Helicase	-7.18	5.504	Gly^{3}, Ala^{4}, Val^{6}, Arg^{21}, Pro^{23}, Phe^{24}, Arg^{129}, Leu^{132}, Phe^{133}, Glu^{136}, Pro^{234}, Leu^{235}, Ser^{236}
			E-channel proteins	-6.48	17.85	Leu^{28}, Val^{29}, Ala^{32}
			Spike Open Protein	-5.78	58.36	Arg^{403}, Tyr^{453}, Ser^{494}, Tyr^{495}, Gly^{496}, Phe^{497}, Asn^{501}, Tyr^{505}
			Spike Close Protein	-6.17	30.22	Trp^{104}, Ile^{119}, Asn^{121}, Val^{126}, Ile^{128}, Tyr^{170}, Ser^{172}, Arg^{190}, Glu^{191}, Phe^{192}, Phe^{194}, Ile^{203}, Ser^{205}, Lys^{206}, His^{207}, Leu^{226}, Val^{227}

Sr.	Ligands	PubChem ID	Proteins	Binding energy (kcal/mol)	Dissociation Constant (µM)	Active site residues
**33**	Apigenin	5280443	Main Protease (MPro)	-7.26	4.74	Met^{49}, Phe^{140}, Leu^{141}, Asn^{142}, Gly^{143}, Ser^{144}, Cys^{145}, His^{163}, Met^{165}, Glu^{166}, Leu^{167}, Pro^{168}, His^{172}, Arg^{188}, Gln^{189}, Thr^{190}, Gln^{192}
			Papain like protease (PLPro)	-9.21	0.18	Phe^{69}, His^{73}, Thr^{74}, Thr^{75}, Asp^{76}, Phe^{79}, Arg^{82}, Ala^{153}, Tyr^{154}, Asn^{156}, Gln^{174}, His^{175}
			RNA-dependent RNA polymerase (RdRP)	-7.48	3.28	Gly^{590}, Thr^{591}, Ser^{592}, Lys^{593}, Phe^{594}, Gln^{815}, Pro^{830}, Tyr^{831}, Pro^{832}, Asp^{865}, Pro^{868}, Trp^{916}
			Helicase	-7.45	3.489	Asn^{179}, Pro^{406}, Pro^{408}, Arg^{409}, Thr^{410}, Leu^{412}, Thr^{416}, Leu^{417}, Phe^{422}, Asn^{557}, Asn^{559}, Arg^{560}
			E-channel proteins	-7.37	3.99	Ile^{46}, Ser^{50}, Leu^{51}, Pro^{54}, Phe^{56}, Tyr^{57}
			Spike Open Protein	-6.86	9.4	Arg^{355}, Tyr^{396}, Pro^{426}, Asp^{428}, Phe^{429}, Thr^{430}, Phe^{464}, Ser^{514}, Phe^{515}, Glu^{516}, Leu^{517}, Leu^{518}
			Spike Close Protein	-7.38	3.89	Glu^{96}, Asn^{99}, Ile^{101}, Arg^{102}, Gly^{103}, Trp^{104}, Ile^{119}, Asn^{121}, Val^{126}, Arg^{190}, Phe^{192}, Ser^{205}, Lys^{206}, His^{207}, Leu^{226}
**34**	Ascorbic acid	54670067	Main Protease (MPro)	-4.96	232.53	Arg^{131}, Lys^{137}, Asp^{197}, Thr^{198}, Thr^{199}, Leu^{286}, Leu^{287}, Glu^{288}, Asp^{289}, Glu^{290}
			Papain like protease (PLPro)	-5.66	71.1	Cys^{155}, Asn^{156}, Lys^{157}, Glu^{167}, Tyr^{171}
			RNA-dependent RNA polymerase (RdRP)	-5.95	43.29	Tyr^{456}, Arg^{555}, Thr^{556}, Val^{557}, Asp^{623}, Arg^{624}, Lys^{676}, Thr^{680}, Ser^{681}, Ser^{682}
			Helicase	-6.51	16.994	Trp^{114}, Tyr^{120}, Ala^{123}, Asn^{124}, Lys^{131}, Ala^{134}, Ala^{135}, Leu^{138}, Thr^{380}, Asn^{381}, Tyr^{382}, Arg^{409}, Leu^{417}, Tyr^{421}, Phe^{422}, Asn^{423}
			E-channel proteins	-4.54	471.63	No binding site was found
			Spike Open Protein	-5.79	56.62	Val^{729}, Ser^{730}, Met^{731}, Thr^{732}, Lys^{733}, Thr^{778}, Phe^{782}, Pro^{863}, Leu^{865}, Asp^{867}, Ile^{870}, Ala^{1056}, Pro^{1057}, His^{1058}, Gly^{1059}
			Spike Close Protein	-5.54	86.92	Phe^{329}, Pro^{330}, Asn^{331}, Ile^{332}, Thr^{333}, Val^{362}, Cys^{525}, Gly^{526}, Pro^{527}, Lys^{528}, Lys^{529}, Ser^{530}, Gln^{580}

Sr.	Ligands	PubChem ID	Proteins	Binding energy (kcal/mol)	Dissociation Constant (µM)	Active site residues
**35**	Baicalin	64982	Main Protease (MPro)	-8.85	0.33	Val^{104}, Ile^{106}, Gln^{110}, Thr^{111}, Asn^{151}, Ile^{152}, Asp^{153}, Tyr^{154}, Pro^{252}, Thr^{292}, Phe^{294}, Asp^{295}, Val^{297}, Arg^{298}, Val^{303}
			Papain like protease (PLPro)	-10.82	0.01	Cys^{155}, Asn^{156}, Lys^{157}, Glu^{161}, Leu^{162}, Gly^{163}, Asp^{164}, Val^{165}, Arg^{166}, Glu^{167},
						Tyr^{171}, Val^{202}, Met^{206}, Met^{208}, Pro^{248}, Tyr^{264}, Tyr^{268}, Gln^{269}, Tyr^{273}
			RNA-dependent RNA polymerase (RdRP)	-9.01	0.25	Val^{31}, Tyr^{32}, Lys^{47}, Tyr^{129}, Ala^{130}, His^{133}, Phe^{134}, Asp^{135}, Asn^{138}, Cys^{139}, Thr^{141}, Asn^{705}, Ala^{706}, Ser^{709}, Thr^{710}, Lys^{780}, Asn^{781}, Ser^{784}
			Helicase	-8.9	0.29	Asn^{177}, Arg^{178}, Thr^{199}, Phe^{200}, Glu^{201}, Lys^{202}, Arg^{212}, Asn^{516}, Asn^{519}, Ala^{520}, Thr^{530}, Gln^{531}, Thr^{532}, Asp^{534}
			E-channel proteins	-8.89	0.3	No binding site was found
			Spike Open Protein	-7.47	3.37	Met^{740}, Gly^{744}, Asp^{745}, Phe^{855}, Asn^{856}, Gly^{857}, Val^{963}, Leu^{966}, Ser^{967}, Ser^{975}, Val^{976}, Leu^{977}, Asn^{978}, Arg^{1000}
			Spike Close Protein	-7.95	1.48	Tyr^{789}, Lys^{790}, Thr^{791}, Lys^{795}, Ser^{803}, Gln^{804}, Leu^{806}, Pro^{807}, Asp^{808}, Pro^{809}, Ser^{816}, Phe^{817}, Ser^{875}, Ala^{876}, Ala^{879}
**36**	Balsacone A	71602340	Main Protease (MPro)	-7.37	3.93	Thr^{24}, Thr^{25}, Thr^{26}, His^{41}, Cys^{44}, Thr^{45}, Ser^{46}, Met^{49}, Asn^{142}, Gly^{143}, His^{164}, Met^{165}, Glu^{166}, Leu^{167}, Pro^{168}, Arg^{188}, Gln^{189}, Thr^{190}, Gln^{192}
			Papain like protease (PLPro)	-9.55	0.1	Leu^{162}, Gly^{163}, Asp^{164}, Val^{165}, Pro^{247}, Pro^{248}, Tyr^{264}, Gly^{266}, Asn^{267}, Tyr^{268}, Tyr^{273}, Thr^{301}
			RNA-dependent RNA polymerase (RdRP)	-7.54	3	Tyr^{32}, Ala^{46}, Lys^{47}, Tyr^{129}, His^{133}, Phe^{134}, Asp^{135}, Asn^{138}, Ala^{706}, Ser^{709}, Thr^{710}, Asp^{711}, Lys^{714}, Gln^{773}, Gly^{774}, Lys^{780}, Asn^{781}, Ser^{784}
			Helicase	-7.69	2.319	Ala^{4}, Val^{6}, Arg^{15}, Arg^{21}, Arg^{22}, Pro^{23}, Phe^{24}, Arg^{129}, Leu^{132}, Phe^{133}, Glu^{136}, Pro^{234}, Leu^{235}, Ser^{236}

Sr.	Ligands	PubChem ID	Proteins	Binding energy (kcal/mol)	Dissociation Constant (µM)	Active site residues
			E-channel proteins	-8.52	0.57	Phe23, Phe26, Leu27, Thr30, Ile46, Leu51, Pro54, Tyr57
			Spike Open Protein	-6.79	10.63	Arg44, His49, Met740, Tyr741, Ile742, Gly744, Asn856, Val963, Lys964, Leu966, Ser967, Ser975, Val976, Leu977, Asn978, Arg1000
			Spike Close Protein	-6.83	9.85	Gly908, Ile909, Gly910, Ser1037, Lys1038, Val1040, Asp1041, Lys1045, Gly1046, Tyr1047, His1048, Arg1107, Asn1108
**37**	Behenic acid	8215	Main Protease (MPro)	-4.12	959.84	Arg131, Thr199, Tyr237, Tyr239, Leu271, Leu272, Gly275, Met276, Asn277, Ala285, Leu286, Leu287, Glu288, Asp289
			Papain like protease (PLPro)	-5.6	78.02	Asp164, Arg166, Met208, Pro247, Pro248, Tyr264, Gly266, Asn267, Tyr268, Tyr273, Thr301
			RNA-dependent RNA polymerase (RdRP)	-4.19	854.32	His613, Phe766, Ser768, Ala771, Ser772, Gln773, Gly774, Ser778, Ile779, Lys780, Glu796, Cys799, Thr801, Thr803
			Helicase	-4.51	493.62	Gly3, Ala4, Val6, Arg21, Arg22, Pro23, Phe24, Arg129, Leu132, Phe133, Glu136, Pro234, Leu235, Ser236
			E-channel proteins	-5.55	86.05	No binding site was found
			Spike Open Protein	-3.21	4414.08	Arg355, Asn394, Tyr396, Pro426, Asp428, Phe429, Thr430, Pro463, Phe464, Ser514, Phe515, Glu516, Leu518
			Spike Close Protein	-5.15	168.16	Leu335, Cys336, Phe338, Gly339, Phe342, Asn343, Val362, Ala363, Asp364, Val367, Leu368, Ser371, Ser373, Phe374, Trp436, Asn437, Leu441
**38**	Benzoic acid	243	Main Protease (MPro)	-5.93	45.23	Met6, Ala7, Phe8, Pro9, Thr111, Gln127, Asp295, Arg298, Gln299, Val303
			Papain like protease (PLPro)	-6.11	33.44	No binding site was found
			RNA-dependent RNA polymerase (RdRP)	-6.48	17.67	No binding site was found
			Helicase	-6.02	38.531	Tyr120, Ala123, Asn124, Lys131, Ala134, Ala135, Leu138, Thr380, Asn381, Tyr382, Arg409, Leu417, Tyr421, Phe422
			E-channel proteins	-5.57	83.05	Leu51, Pro54, Tyr57

Sr.	Ligands	PubChem ID	Proteins	Binding energy (kcal/mol)	Dissociation Constant (µM)	Active site residues
			Spike Open Protein	-5	215.52	Met^{740}, Tyr^{741}, Ile^{742}, Cys^{743}, Gly^{744}, Asn^{856}, Leu^{966}, Ser^{975}, Val^{976}, Leu^{977}, Asn^{978}, Arg^{1000}
			Spike Close Protein	-5.82	53.91	Tyr^{38}, Asp^{40}, Lys^{41}, Val^{42}, Phe^{43}, Arg^{44}, Glu^{224}, Pro^{225}, Tyr^{279}, Gly^{283}
39	Biochanin	5280373	Main Protease (MPro)	-6.85	9.59	Thr^{25}, Thr^{26}, Leu^{27}, His^{41}, Met^{49}, Leu^{141}, Asn^{142}, Gly^{143}, Ser^{144}, Cys^{145}, His^{164}, Met^{165}, Glu^{166}, Leu^{167}, Pro^{168}, Arg^{188}, Gln^{189}, Thr^{190}, Gln^{192}
			Papain like protease (PLPro)	-9.04	0.24	Thr^{74}, Asp^{76}, Phe^{79}, Arg^{82}, Ala^{153}, Tyr^{154}, Asn^{156}, Tyr^{171}, Gln^{174}, His^{175}
			RNA-dependent RNA polymerase (RdRP)	-7.51	3.12	Arg^{33}, Phe^{35}, Asp^{36}, Ile^{37}, Tyr^{38}, Lys^{50}, Val^{71}, Lys^{73}, Thr^{123}, Thr^{206}, Asp^{208}, Asn^{209}, Tyr^{217}, Asp^{218}, Asp^{221}
			Helicase	-7.31	4.397	Asn^{177}, Asn^{179}, Glu^{201}, Pro^{514}, Tyr^{515}, Asn^{516}, Asn^{519}, Ala^{520}, Thr^{530}, Thr^{532}, Val^{533}, Asp^{534}, Arg^{560}
			E-channel proteins	-6.81	10.19	No binding site was found
			Spike Open Protein	-6.29	24.59	Tyr^{38}, Lys^{41}, Val^{42}, Phe^{43}, Arg^{44}, Glu^{224}, Pro^{225}, Leu^{226}, Gly^{283}
			Spike Close Protein	-6.78	10.77	Phe^{329}, Pro^{330}, Asn^{331}, Thr^{333}, Leu^{335}, Val^{362}, Asp^{364}, Cys^{525}, Gly^{526}, Pro^{527}, Lys^{528}, Lys^{529}, Ser^{530}, Gln^{580}
40	Butein	5281222	Main Protease (MPro)	-7.74	2.14	Phe^{3}, Met^{6}, Ala^{7}, Phe^{8}, Pro^{9}, Gln^{127}, Ile^{152}, Phe^{291}, Asp^{295}, Arg^{298}, Gln^{299}, Val^{303}, Thr^{304}
			Papain like protease (PLPro)	-7.76	2.04	Pro^{59}, Asp^{62}, Arg^{65}, Val^{66}, Ala^{68}, Phe^{69}, Tyr^{72}, Thr^{74}, Thr^{75}, Asp^{76}, Pro^{77}, Phe^{79}, Leu^{80}
			RNA-dependent RNA polymerase (RdRP)	-7.39	3.83	Lys^{411}, Pro^{412}, Gly^{413}, Asn^{414}, Tyr^{546}, Asp^{845}, Asp^{846}, Ile^{847}
			Helicase	-7.09	6.342	Pro^{175}, Asn^{177}, Asn^{179}, Tyr^{180}, Pro^{406}, Ala^{407}, Pro^{408}, Arg^{409}, Thr^{410}, Leu^{412}, Thr^{413}, Thr^{416}, Leu^{417}, Phe^{422}, Asn^{557}, Arg^{560}
			E-channel proteins	-7.28	4.64	No binding site was found

Sr.	Ligands	PubChem ID	Proteins	Binding energy (kcal/mol)	Dissociation Constant (µM)	Active site residues
			Spike Open Protein	-6.77	10.88	Arg^{355}, Tyr^{396}, Pro^{426}, Asp^{428}, Phe^{429}, Thr^{430}, Phe^{464}, Ser^{514}, Phe^{515}, Glu^{516}, Leu^{517}, Leu^{518}
			Spike Close Protein	-7.57	2.81	Tyr^{789}, Lys^{790}, Thr^{791}, Pro^{792}, Asp^{796}, Phe^{797}, Ala^{879}, Ile^{882}, Thr^{883}, Ala^{893}, Gln^{895}, Ile^{896}, Pro^{897}, Phe^{898}
**41**	Caffeic acid	689043	Main Protease (MPro)	-7.09	6.4	Phe^{3}, Met^{6}, Phe^{8}, Gln^{127}, Phe^{291}, Asp^{295}, Arg^{298}, Gln^{299}, Gly^{302}, Val^{303}
			Papain like protease (PLPro)	-7.28	4.59	His^{73}, Thr^{74}, Thr^{75}, Gln^{174}, His^{175}
			RNA-dependent RNA polymerase (RdRP)	-6.76	11.13	Ser^{592}, Lys^{593}, Phe^{594}, Gln^{815}, Pro^{830}, Tyr^{831}, Pro^{832}, Ile^{864}, Asp^{865}, Ala^{866}, Pro^{868}, Trp^{916}
			Helicase	-7.15	5.731	Tyr^{120}, Ala^{123}, Asn^{124}, Ala^{135}, Leu^{138}, Lys^{139}, Thr^{380}, Asn^{381}, Tyr^{382}, Arg^{409}, Leu^{417}, Tyr^{421}, Phe^{422}
			E-channel proteins	-6.39	20.77	No binding site was found
			Spike Open Protein	-6.65	13.37	Tyr^{38}, Asp^{40}, Lys^{41}, Val^{42}, Phe^{43}, Glu^{224}, Pro^{225}, Asn^{282}, Gly^{283}, Thr^{284}
			Spike Close Protein	-6.69	12.58	Tyr^{38}, Asp^{40}, Lys^{41}, Val^{42}, Phe^{43}, Arg^{44}, Glu^{224}, Pro^{225}, Tyr^{279}, Asn^{282}, Gly^{283}, Thr^{284}
**42**	Cinnamic acid	444539	Main Protease (MPro)	-6.44	19.09	Phe^{3}, Lys^{5}, Met^{6}, Phe^{8}, Pro^{9}, Gln^{127}, Phe^{291}, Asp^{295}, Arg^{298}, Gln^{299}, Gly^{302}, Val^{303}, Thr^{304}
			Papain like protease (PLPro)	-6.81	10.26	Lys^{157}, Leu^{162}, Gly^{163}, Asp^{164}, Val^{165}, Pro^{248}, Tyr^{264}, Tyr^{268}, Tyr^{273}, Thr^{301}
			RNA-dependent RNA polymerase (RdRP)	-6.23	27.17	Pro^{323}, Thr^{324}, Phe^{326}, Gly^{327}, Tyr^{346}, His^{347}, Phe^{348}, Arg^{349}, Glu^{350}, Phe^{396}, Asn^{628}, Ser^{664}, Val^{675}, Lys^{676}, Pro^{677}basn^{118}
			Helicase	-6.24	26.535	Pro^{406}, Ala^{407}, Arg^{409}, Leu^{412}, Thr^{416}, Leu^{417}, Pro^{419}, Phe^{422}, Asn^{557}, Asn^{559}, Arg^{560}
			E-channel proteins	-6.41	19.92	No binding site was found

Sr.	Ligands	PubChem ID	Proteins	Binding energy (kcal/mol)	Dissociation Constant (µM)	Active site residues
			Spike Open Protein	-6.19	29.21	Tyr^{38}, Asp^{40}, Lys^{41}, Val^{42}, Phe^{43}, Arg^{44}, Glu^{224}, Pro^{225}, Tyr^{279}, Gly^{283}
			Spike Close Protein	-6.15	30.94	Trp^{104}, Ile^{119}, Asn^{121}, Val^{126}, Ile^{128}, Arg^{190}, Phe^{192}, Phe^{194}, Ile^{203}, Val^{227}
**43**	Citric acid	311	Main Protease (MPro)	-5.24	145.44	Phe^{8}, Ile^{106}, Gln^{110}, Thr^{111}, Asn^{151}, Ile^{152}, Asp^{153}, Thr^{292}, Phe^{294}, Asp^{295}, Arg^{298}
			Papain like protease (PLPro)	-6.18	29.36	His^{73}, Thr^{74}, Thr^{75}, Asp^{76}, Phe^{79}, Arg^{82}, Ala^{153}, Tyr^{154}, Asn^{156}, Tyr^{171}, Gln^{174}, His^{175}
			RNA-dependent RNA polymerase (RdRP)	-5.34	122.03	Lys^{593}, Phe^{594}, Gln^{815}, Pro^{830}, Tyr^{831}, Pro^{832}, Asp^{865}, Pro^{868}, Trp^{916}
			Helicase	-5.74	61.706	Leu^{405}, Pro^{406}, Thr^{416}, Leu^{417}, Pro^{419}, Phe^{422}, Asn^{557}, Asn^{559}, Arg^{560}
			E-channel proteins	-4.83	286.66	No binding site was found
			Spike Open Protein	-5.27	138.26	Met^{740}, Tyr^{741}, Ile^{742}, Cys^{743}, Gly^{744}, Asn^{856}, Leu^{966}, Ser^{975}, Val^{976}, Leu^{977}, Asn^{978}, Arg^{1000}
			Spike Close Protein	-5.71	64.8	Phe^{329}, Pro^{330}, Asn^{331}, Ile^{332}, Thr^{333}, Val^{362}, Cys^{525}, Gly^{526}, Pro^{527}, Lys^{528}, Lys^{529}, Ser^{530}, Gln^{580}
**44**	Cryptochlorogenic acid	9798666	Main Protease (MPro)	-7.99	1.4	Arg^{131}, Lys^{137}, Asp^{197}, Thr^{198}, Thr^{199}, Tyr^{239}, Leu^{271}, Leu^{272}, Gly^{275}, Met^{276}, Ala^{285}, Leu^{286}, Leu^{287}, Asp^{289}, Glu^{290}
			Papain like protease (PLPro)	-9.25	0.16	Arg^{166}, Ser^{170}, Val^{202}, Glu^{203}, Met^{206}, Tyr^{207}, Met^{208}
			RNA-dependent RNA polymerase (RdRP)	-7.39	3.82	Tyr^{149}, Cys^{151}, Ala^{176}, Asn^{177}, Leu^{178}, Gly^{179}, Glu^{180}, Arg^{181}, Gln^{184}, Arg^{249}, Thr^{252}, Leu^{261}
			Helicase	-7.95	1.501	Pro^{175}, Asn^{177}, Asn^{179}, Tyr^{180}, Pro^{406}, Pro^{408}, Arg^{409}, Thr^{410}, Leu^{412}, Thr^{413}, Leu^{417}, Pro^{419}, Phe^{422}, Asn^{557}, Asn^{559}, Arg^{560}
			E-channel proteins	-7.52	3.08	Ile^{46}, Leu^{51}, Pro^{54}, Phe^{56}, Tyr^{57}, Ser^{60}, Arg^{61}

Sr.	Ligands	PubChem ID	Proteins	Binding energy (kcal/mol)	Dissociation Constant (µM)	Active site residues
			Spike Open Protein	-7.26	4.81	Met^{740}, Tyr^{741}, Ile^{742}, Cys^{743}, Gly^{744}, Asn^{856}, Val^{963}, Lys^{964}, Leu^{966}, Ser^{967}, Ser^{975}, Val^{976}, Leu^{977}, Asn^{978}, Arg^{1000}
			Spike Close Protein	-7.14	5.8	Gly^{908}, Ile^{909}, Gly^{910}, Gln^{1036}, Ser^{1037}, Lys^{1038}, Val^{1040}, Gly^{1046}, Tyr^{1047}, His^{1048}, Glu^{1092}, Gly^{1093}, Gln^{1106}, Arg^{1107}, Asn^{1108}
**45**	curcumin	969516	Main Protease (MPro)	-7.28	4.62	Lys^{5}, Arg^{131}, Lys^{137}, Thr^{199}, Tyr^{237}, Tyr^{239}, Leu^{271}, Leu^{272}, Met^{276}, Ala^{285}, Leu^{286}, Leu^{287}, Glu^{288}, Asp^{289}, Glu^{290}
			Papain like protease (PLPro)	-9.09	0.22	Asp^{164}, Arg^{166}, Met^{208}, Ala^{246}, Pro^{247}, Pro^{248}, Tyr^{264}, Tyr^{268}, Tyr^{273}, Thr^{301}
			RNA-dependent RNA polymerase (RdRP)	-7.3	4.46	Tyr^{32}, Lys^{47}, Tyr^{122}, Tyr^{129}, Ala^{130}, His^{133}, Phe^{134}, Asn^{138}, Cys^{139}, Asp^{140}, Thr^{141}, Leu^{142}
			Helicase	-7.4	3.79	Leu^{176}, Asn^{177}, Arg^{178}, Thr^{199}, Phe^{200}, Glu^{201}, Lys^{202}, Gly^{203}, Arg^{212}, Asn^{516}, Ser^{517}, Asn^{519}, Ala^{520}, Thr^{530}, Gln^{531}, Thr^{532}, Asp^{534}
			E-channel proteins	-8.27	0.87	Ile^{46}, Ser^{50}, Leu^{51}, Pro^{54}, Phe^{56}, Tyr^{57}, Ser^{60}
			Spike Open Protein	-6.63	13.93	Ile^{105}, Leu^{118}, Val^{120}, Asn^{122}, Ala^{123}, Asn^{125}, Val^{127}, Lys^{129}, Phe^{135}, Cys^{136}, Pro^{139}, Phe^{140}, Leu^{141}, Val^{171}, Leu^{241}
			Spike Close Protein	-7.32	4.29	Asn^{99}, Ile^{101}, Arg^{102}, Gly^{103}, Trp^{104}, Ile^{119}, Asn^{121}, Val^{126}, Ile^{128}, Tyr^{170}, Ser^{172}, Arg^{190}, Phe^{192}, Phe^{194}, Ile^{203}, Leu^{226}, Val^{227}
			Main Protease (MPro)	-4.84	282.81	Arg^{131}, Thr^{199}, Tyr^{237}, Asn^{238}, Tyr^{239}, Leu^{271}, Leu^{272}, Gly^{275}, Met^{276}, Ala^{285}, Leu^{286}, Leu^{287}, Glu^{288}, Asp^{289}
			Papain like protease (PLPro)	-6.34	22.45	Asp^{164}, Arg^{166}, Met^{208}, Pro^{247}, Pro^{248}, Tyr^{264}, Tyr^{268}, Tyr^{273}, Thr^{301}
			RNA-dependent RNA polymerase (RdRP)	-4.33	672.25	Arg^{305}, Glu^{474}, Asp^{477}, Lys^{478}, Phe^{480}, Asp^{481}, Val^{637}, Arg^{640}, Lys^{641}, Thr^{643}, Val^{737}, Thr^{739}, Val^{742}, Asn^{743}
**46**	Eicosadienoic acid	6439848	Helicase	-5.06	194.762	Val^{6}, Arg^{15}, Arg^{21}, Arg^{22}, Pro^{23}, Phe^{24}, Arg^{129}, Leu^{132}, Phe^{133}, Glu^{136}, Pro^{234}, Leu^{235}, Ser^{236}

Sr.	Ligands	PubChem ID	Proteins	Binding energy (kcal/mol)	Dissociation Constant (µM)	Active site residues
			E-channel proteins	-6.04	37.19	No binding site was found
			Spike Open Protein	-4.37	630.49	Ser^{730}, Met^{731}, Thr^{732}, Lys^{733}, Thr^{734}, Val^{860}, Leu^{861}, Pro^{862}, Pro^{863}, Val^{952}, Asn^{955}, Ala^{956}, Leu^{959}, Asn^{960}, His^{1058}
			Spike Close Protein	-6.17	29.86	Glu^{96}, Asn^{99}, Ile^{101}, Arg^{102}, Trp^{104}, Ile^{119}, Asn^{121}, Val^{126}, Ile^{128}, Phe^{168}, Tyr^{170}, Ser^{172}, Arg^{190}, Phe^{192}, Phe^{194}, Ile^{203}, Leu^{226}, Val^{227}, Leu^{229}
**47**	Ellagic acid	5281855	Main Protease (MPro)	-7.93	1.54	Phe^{8}, Lys^{102}, Val^{104}, Ile^{106}, Gln^{110}, Thr^{111}, Asn^{151}, Ile^{152}, Asp^{153}, Ser^{158}, Thr^{292}, Phe^{294}, Asp^{295}, Arg^{298}
			Papain like protease (PLPro)	-8.41	0.69	Lys^{157}, Glu^{161}, Leu^{162}
			RNA-dependent RNA polymerase (RdRP)	-7.59	2.72	Lys^{47}, Tyr^{129}, His^{133}, Phe^{134}, Asp^{135}, Asn^{138}, Ala^{706}, Ser^{709}, Thr^{710}, Asp^{711}, Lys^{780}, Asn^{781}, Ser^{784}
			Helicase	-8.08	1.189	Pro^{406}, Pro^{408}, Arg^{409}, Thr^{410}, Leu^{412}, Gly^{415}, Thr^{416}, Leu^{417}, Asn^{557}, Asn^{559}, Arg^{560}
			E-channel proteins	-6.77	10.98	Leu^{28}, Val^{29}, Ala^{32}, Ile^{33}
			Spike Open Protein	-8.1	1.16	Ser^{730}, Met^{731}, Thr^{732}, Lys^{733}, Gln^{774}, Thr^{778}, Phe^{823}, Val^{860}, Leu^{861}, Pro^{862}, Pro^{863}, Asp^{867}, Ile^{870}, Ala^{1056}, Pro^{1057}, His^{1058}, Gly^{1059}
			Spike Close Protein	-7.41	3.71	Arg^{355}, Tyr^{396}, Pro^{426}, Asp^{428}, Phe^{429}, Thr^{430}, Pro^{463}, Phe^{464}, Ser^{514}, Phe^{515}, Glu^{516}
**48**	Epicatechin	72276	Main Protease (MPro)	-7.18	5.45	Arg^{131}, Lys^{137}, Asp^{197}, Thr^{199}, Val^{204}, Tyr^{237}, Asn^{238}, Tyr^{239}, Leu^{272}, Leu^{286}, Leu^{287}, Glu^{288}, Asp^{289}
			Papain like protease (PLPro)	-9.28	0.16	Thr^{74}, Thr^{75}, Asp^{76}, Tyr^{154}, Tyr^{171}, Gln^{174}, His^{175}
			RNA-dependent RNA polymerase (RdRP)	-7.39	3.84	Gly^{590}, Thr^{591}, Ser^{592}, Lys^{593}, Phe^{594}, Gln^{815}, Pro^{830}, Tyr^{831}, Pro^{832}, Asp^{865}, Pro^{868}, Trp^{916}
			Helicase	-7.22	5.127	Asn^{179}, Pro^{406}, Ala^{407}, Pro^{408}, Arg^{409}, Thr^{410}, Leu^{412}, Thr^{416}, Leu^{417}, Glu^{418}, Pro^{419}, Phe^{422}, Asn^{557}, Asn^{559}, Arg^{560}

Sr.	Ligands	PubChem ID	Proteins	Binding energy (kcal/mol)	Dissociation Constant (µM)	Active site residues
			E-channel proteins	-7.06	6.65	Ile^{46}, Leu^{51}, Pro^{54}, Phe^{56}, Tyr^{57}, Ser^{60}
			Spike Open Protein	-7.01	7.26	Tyr^{38}, Pro^{39}, Asp^{40}, Lys^{41}, Val^{42}, Phe^{43}, Arg^{44}, Glu^{224}, Pro^{225}, Leu^{226}, Tyr^{279}, Gly^{283}
			Spike Close Protein	-7.21	5.18	Asp^{420}, Tyr^{421}, Asn^{422}, Tyr^{423}, Lys^{424}, Lys^{462}, Phe^{464}, Glu^{465}, Arg^{466}, Asp^{467}
**49**	Eriodictyol	440735	Main Protease (MPro)	-7.62	2.58	Met^{6}, Ala^{7}, Phe^{8}, Pro^{9}, Ser^{113}, Gln^{127}, Asp^{295}, Arg^{298}, Gln^{299}, Gly^{302}, Val^{303}, Thr^{304}
			Papain like protease (PLPro)	-8.13	1.11	Arg^{183}, Leu^{185}, Val^{187}, Thr^{197}, Leu^{199}, Glu^{203}, Met^{206}, Tyr^{207}, Met^{208}, Lys^{232}
			RNA-dependent RNA polymerase (RdRP)	-7.61	2.65	Thr^{591}, Ser^{592}, Lys^{593}, Phe^{594}, Gln^{815}, Pro^{830}, Tyr^{831}, Pro^{832}, Asp^{865}, Pro^{868}, Trp^{916}
			Helicase	-7.77	2.03	Asn^{179}, Tyr^{180}, Leu^{405}, Pro^{406}, Pro^{408}, Arg^{409}, Thr^{410}, Leu^{412}, Thr^{413}, Thr^{416}, Leu^{417}, Phe^{422}, Asn^{557}, Asn^{559}, Arg^{560}
			E-channel proteins	-7.25	4.86	Ile^{46}, Ser^{50}, Leu^{51}, Pro^{54}, Phe^{56}, Tyr^{57}
			Spike Open Protein	-7.2	5.29	Met^{740}, Tyr^{741}, Ile^{742}, Cys^{743}, Gly^{744}, Asn^{856}, Leu^{966}, Ser^{967}, Ser^{975}, Val^{976}, Leu^{977}, Asn^{978}, Arg^{1000}
			Spike Close Protein	-7	7.44	Ala^{958}, Thr^{961}, Leu^{962}, Gln^{965}, Phe^{970}, Gly^{999}, Gln^{1002}, Ser^{1003}, Thr^{1006}, Tyr^{1007}, Gln^{1010}
**50**	Euchrestaflavanone A	484588	Main Protease (MPro)	-7.94	1.51	Ser^{46}, Met^{49}, Phe^{140}, Leu^{141}, Asn^{142}, Gly^{143}, Ser^{144}, Cys^{145}, His^{163}, His^{164}, Met^{165}, Glu^{166}, Leu^{167}, Pro^{168}, Arg^{188}, Gln^{189}, Thr^{190}, Ala^{191}, Gln^{192}
			Papain like protease (PLPro)	-9.95	0.05	Lys^{157}, Glu^{161}, Leu^{162}, Gly^{163}, Asp^{164}, Arg^{166}, Glu^{167}, Ser^{170}, Val^{202}, Glu^{203}, Met^{206}, Tyr^{207}, Met^{208}, Tyr^{268}
			RNA-dependent RNA polymerase (RdRP)	-8.25	0.9	Phe^{35}, Asp^{36}, Ile^{37}, Tyr^{38}, Asn^{39}, Val^{42}, Phe^{48}, Leu^{49}, Lys^{50}, Thr^{206}, Asp^{208}, Asn^{209}, Asp^{218}, Asp^{221}, Asn^{713}

Sr.	Ligands	PubChem ID	Proteins	Binding energy (kcal/mol)	Dissociation Constant (µM)	Active site residues
			Helicase	-8.88	0.308	Pro^{175}, Leu^{176}, Asn^{177}, Glu^{201}, Lys^{202}, Gly^{203}, Asp^{204}, Val^{484}, Asn^{516}, Ser^{517}, Asn^{519}, Ala^{520}, Thr^{530}, Thr^{532}, Val^{533}, Asp^{534}
			E-channel proteins	-8.9	0.3	Arg^{61}, Asn^{64}
			Spike Open Protein	-7.58	2.78	Tyr^{38}, Asp^{40}, Lys^{41}, Val^{42}, Phe^{43}, Arg^{44}, Glu^{224}, Pro^{225}, Leu^{226}, Tyr^{279}, Asn^{282}, Gly^{283}, Thr^{284}
			Spike Close Protein	-7.14	5.83	Arg^{355}, Asn^{394}, Tyr^{396}, Pro^{426}, Asp^{428}, Phe^{429}, Thr^{430}, Lys^{462}, Pro^{463}, Phe^{464}, Ser^{514}, Phe^{515}, Glu^{516}
51	Ferulic acid	445858	Main Protease (MPro)	-6.29	24.35	Arg^{4}, Met^{6}, Ala^{7}, Phe^{8}, Pro^{9}, Gln^{127}, Phe^{291}, Asp^{295}, Arg^{298}, Gln^{299}, Gly^{302}, Val^{303}
			Papain like protease (PLPro)	-6.62	14.09	Asp^{164}, Arg^{166}, Met^{208}, Pro^{247}, Pro^{248}, Tyr^{264}, Asn^{267}, Tyr^{268}, Thr^{301}
			RNA-dependent RNA polymerase (RdRP)	-6.08	34.76	Lys^{411}, Pro^{412}, Gly^{413}, Asn^{414}, Phe^{441}, Tyr^{546}, Val^{844}, Asp^{845}, Asp^{846}
			Helicase	-6.24	26.715	Pro^{406}, Ala^{407}, Arg^{409}, Leu^{412}, Thr^{413}, Gly^{415}, Thr^{416}, Leu^{417}, Pro^{419}, Phe^{422}, Asn^{557}, Asn^{559}, Arg^{560}
			E-channel proteins	-6.35	22	No binding site was found
			Spike Open Protein	-5.67	70.03	Met^{740}, Tyr^{741}, Gly^{744}, Asn^{856}, Leu^{966}, Ser^{967}, Ser^{975}, Val^{976}, Leu^{977}, Asn^{978}, Arg^{1000}
			Spike Close Protein	-6.94	8.25	Tyr^{38}, Asp^{40}, Lys^{41}, Val^{42}, Phe^{43}, Arg^{44}, Glu^{224}, Pro^{225}, Tyr^{279}, Asn^{282}, Gly^{283}, Thr^{284}
			Main Protease (MPro)	-8.12	1.12	Thr^{24}, Thr^{25}, Thr^{26}, His^{41}, Cys^{44}, Thr^{45}, Ser^{46}, Met^{49}, Phe^{140}, Leu^{141}, Asn^{142}, Gly^{143}, Ser^{144}, Cys^{145}, His^{163}, Met^{165}, Glu^{166}, Arg^{188}, Gln^{189}
			Papain like protease (PLPro)	-10.08	0.04	Lys^{157}, Glu^{161}, Leu^{162}, Gly^{163}, Asp^{164}, Arg^{166}, Glu^{167}, Ser^{170}, Val^{202}, Glu^{203}, Met^{206}, Tyr^{207}, Met^{208}, Tyr^{264}, Tyr^{268}, Gln^{269}, Tyr^{273}

Sr.	Ligands	PubChem ID	Proteins	Binding energy (kcal/mol)	Dissociation Constant (µM)	Active site residues
**52**	Flemiflavanone D	42607928	RNA-dependent RNA polymerase (RdRP)	-8.05	1.27	Leu^{270}, Pro^{322}, Pro^{323}, Thr^{324}, Arg^{349}, Leu^{387}, Leu^{388}, Leu^{389}, Asp^{390}, Thr^{393}, Thr^{394}, Cys^{395}, Phe^{396}, Ser^{397}
			Helicase	-8.21	0.959	Leu^{176}, Asn^{177}, Arg^{178}, Asn^{179}, Glu^{201}, Lys^{202}, Gly^{203}, Asp^{204}, Asn^{516}, Asn^{519}, Ala^{520}, Thr^{530}, Gln^{531}, Thr^{532}, Asp^{534}, Ser^{535}
			E-channel proteins	-8.18	1.01	No binding site was found
			Spike Open Protein	-7.81	1.89	Ser^{730}, Met^{731}, Thr^{732}, Lys^{733}, Thr^{778}, Phe^{823}, Val^{826}, Thr^{827}, Val^{860}, Leu^{861}, Pro^{862}, Pro^{863}, Asp^{867}, Ile^{870}, Val^{952}, Pro^{1057}, His^{1058}
			Spike Close Protein	-8.01	1.34	Tyr^{38}, Asp^{40}, Lys^{41}, Val^{42}, Phe^{43}, Arg^{44}, Ser^{45}, Glu^{224}, Pro^{225}, Asn^{282}, Gly^{283}, Thr^{284}
**53**	Formononetin	5280378	Main Protease (MPro)	-7.04	6.95	Thr^{25}, His^{41}, Cys^{44}, Thr^{45}, Ser^{46}, Met^{49}, Met^{165}, Glu^{166}, Leu^{167}, Pro^{168}, Arg^{188}, Gln^{189}, Thr^{190}, Gln^{192}
			Papain like protease (PLPro)	-8.17	1.02	Lys^{157}, Leu^{162}, Gly^{163}, Asp^{164}, Pro^{248}, Tyr^{264}, Tyr^{268}, Gln^{269}, Tyr^{273}
			RNA-dependent RNA polymerase (RdRP)	-6.54	16.13	Phe^{35}, Ile^{37}, Phe^{48}, Leu^{49}, Lys^{50}, Thr^{206}, Asp^{208}, Asn^{209}, Asp^{218}, Asp^{221}
			Helicase	-7.31	4.375	Pro^{175}, Asn^{179}, Tyr^{180}, Pro^{406}, Pro^{408}, Arg^{409}, Thr^{410}, Leu^{412}, Leu^{417}, Phe^{422}, Asn^{557}, Asn^{559}, Arg^{560}
			E-channel proteins	-7.15	5.75	Leu^{28}, Val^{29}, Leu^{31}, Ala^{32}, Thr^{35}, Cys^{40}
			Spike Open Protein	-6.4	20.29	Tyr^{38}, Lys^{41}, Val^{42}, Phe^{43}, Arg^{44}, Glu^{224}, Pro^{225}, Leu^{226}, Tyr^{279}, Gly^{283}
			Spike Close Protein	-6.76	11.11	Phe^{329}, Pro^{330}, Asn^{331}, Ile^{332}, Thr^{333}, Leu^{335}, Val^{362}, Asp^{364}, Gly^{526}, Pro^{527}, Lys^{528}, Lys^{529}, Ser^{530}, Gln^{580}
			Main Protease (MPro)	-4.79	310.85	Met^{6}, Ala^{7}, Phe^{8}, Gln^{127}, Phe^{291}, Asp^{295}, Arg^{298}, Gln^{299}, Val^{303}
			Papain like protease (PLPro)	-5.63	74.8	No binding site was found

Sr.	Ligands	PubChem ID	Proteins	Binding energy (kcal/mol)	Dissociation Constant (µM)	Active site residues
54	Fumaric acid	444972	RNA-dependent RNA polymerase (RdRP)	-5.6	79.08	Asp^{452}, Tyr^{453}, Tyr^{455}, Tyr^{456}, Met^{542}, Thr^{556}, Val^{557}, Ala^{558}, Asp^{623}, Arg^{624}, Thr^{680}, Ser^{681}, Ser^{682}
			Helicase	-6.17	30.065	Tyr^{120}, Ala^{123}, Asn^{124}, Ala^{135}, Leu^{138}, Thr^{380}, Asn^{381}, Tyr^{382}, Arg^{409}, Tyr^{421}, Phe^{422}, Asn^{423}
			E-channel proteins	-4.39	601.39	No binding site was found
			Spike Open Protein	-4.73	342.82	Ser^{730}, Met^{731}, Thr^{732}, Lys^{733}, Gln^{774}, Thr^{778}, Pro^{863}, Ile^{870}, Pro^{1057}, His^{1058}, Gly^{1059}
			Spike Close Protein	-5.06	195.09	Ser^{730}, Thr^{778}, Pro^{863}, Leu^{865}, Thr^{866}, Asp^{867}, Ile^{870}, Pro^{1057}, His^{1058}
55	Galangin	5281616	Main Protease (MPro)	-7.88	1.68	Met^{6}, Ala^{7}, Phe^{8}, Pro^{9}, Ser^{113}, Gln^{127}, Asp^{295}, Arg^{298}, Gln^{299}, Gly^{302}, Val^{303}, Thr^{304}
			Papain like protease (PLPro)	-8.49	0.6	Arg^{166}, Met^{208}
			RNA-dependent RNA polymerase (RdRP)	-7.21	5.21	Lys^{411}, Pro^{412}, Gly^{413}, Asn^{414}, Tyr^{546}, Val^{844}, Asp^{845}, Asp^{846}
			Helicase	-7.21	5.214	Ala^{135}, Leu^{138}, Lys^{139}, Glu^{142}, Asn^{179}, Tyr^{180}, Met^{378}, Ala^{379}, Thr^{380}, Tyr^{382}, Asp^{383}, Ala^{407}, Pro^{408}, Arg^{409}, Thr^{410}
			E-channel proteins	-7.37	3.94	Leu^{28}, Val^{29}, Ala^{32}, Ile^{33}, Ala^{36}
			Spike Open Protein	-7.24	4.94	Trp^{104}, Ile^{119}, Asn^{121}, Val^{126}, Ile^{128}, Arg^{190}, Phe^{192}, Phe^{194}, Phe^{201}, Ile^{203}, His^{207}, Leu^{226}, Val^{227}
			Spike Close Protein	-7.03	7.03	Leu^{335}, Cys^{336}, Phe^{338}, Gly^{339}, Phe^{342}, Asn^{343}, Val^{362}, Ala^{363}, Asp^{364}, Val^{367}, Leu^{368}, Ser^{371}, Ser^{373}, Phe^{374}
			Main Protease (MPro)	-6.59	14.9	Met^{6}, Ala^{7}, Phe^{8}, Gln^{127}, Phe^{291}, Asp^{295}, Arg^{298}, Gln^{299}, Gly^{302}, Val^{303}
			Papain like protease (PLPro)	-7.1	6.25	No binding site was found
			RNA-dependent RNA polymerase (RdRP)	-6.5	17.31	Gly^{616}, Trp^{617}, Asp^{618}, Asp^{761}, Ala^{762}, Val^{763}, Lys^{798}, Cys^{799}, Trp^{800}, Glu^{811}, Phe^{812}, Cys^{813}, Ser^{814}

Sr.	Ligands	PubChem ID	Proteins	Binding energy (kcal/mol)	Dissociation Constant (µM)	Active site residues
**56**	Gallic acid	370	Helicase	-5.84	52.21	Val^{496}, Arg^{497}, Leu^{500}, Thr^{501}, Trp^{506}, Arg^{507}, Ala^{509}, Leu^{526}, Gly^{527}, Leu^{528}, Tyr^{543}, Ile^{545}, Leu^{573}
			E-channel proteins	-5.23	147.92	No binding site was found
			Spike Open Protein	-5.72	63.82	Gly^{908}, Ile^{909}, Gln^{1036}, Ser^{1037}, Lys^{1038}, Arg^{1039}, Val^{1040}, Asp^{1041}, Gly^{1046}, Tyr^{1047}, His^{1048}
			Spike Close Protein	-6.52	16.63	Cys^{391}, Phe^{392}, Thr^{393}, Glu^{516}, Leu^{517}, Leu^{518}, His^{519}, Ala^{520}, Pro^{521}, Ala^{522}, Thr^{523}, Phe^{543}, Asn^{544}, Gly^{545}, Leu^{546}, Gln^{564}, Phe^{565}
**57**	Gensitic acid	3469	Main Protease (MPro)	-6.59	14.7	Phe^{3}, Met^{6}, Ala^{7}, Phe^{8}, Gln^{127}, Phe^{291}, Asp^{295}, Val^{296}, Arg^{298}, Gln^{299}, Gly^{302}, Val^{303}
			Papain like protease (PLPro)	-6.56	15.44	No binding site was found
			RNA-dependent RNA polymerase (RdRP)	-6.11	33.16	Phe^{326}, Gly^{327}, Tyr^{346}, His^{347}, Phe^{348}, Arg^{349}, Glu^{350}, Asn^{628}, Ser^{664}, Val^{675}, Lys^{676}, Pro^{677}, Gly^{678}
			Helicase	-7.09	6.396	Tyr^{120}, Ala^{123}, Asn^{124}, Lys^{131}, Ala^{134}, Ala^{135}, Leu^{138}, Thr^{380}, Asn^{381}, Tyr^{382}, Arg^{409}, Leu^{417}, Tyr^{421}, Phe^{422}
			E-channel proteins	-5.56	84.61	Leu^{51}, Pro^{54}, Tyr^{57}
			Spike Open Protein	-6.05	36.63	Tyr^{38}, Asp^{40}, Lys^{41}, Val^{42}, Phe^{43}, Arg^{44}, Glu^{224}, Pro^{225}, Tyr^{279}, Gly^{283}, Thr^{284}
			Spike Close Protein	-6.01	39.65	Tyr^{38}, Asp^{40}, Lys^{41}, Val^{42}, Phe^{43}, Arg^{44}, Glu^{224}, Pro^{225}, Tyr^{279}, Gly^{283}, Thr^{284}
			Main Protease (MPro)	-6.45	18.71	Met^{6}, Ala^{7}, Phe^{8}, Pro^{9}, Thr^{111}, Gln^{127}, Phe^{291}, Asp^{295}, Arg^{298}, Gln^{299}, Val^{303}
			Papain like protease (PLPro)	-6.51	16.82	No binding site was found
			RNA-dependent RNA polymerase (RdRP)	-6.16	30.42	Arg^{305}, Arg^{467}, Leu^{470}, Phe^{471}, Glu^{474}, Cys^{730}, Leu^{731}, Arg^{733}, Asn^{734}, Arg^{735}, Val^{737}, Asp^{738}

Sr.	Ligands	PubChem ID	Proteins	Binding energy (kcal/mol)	Dissociation Constant (µM)	Active site residues
**58**	Gentisic acid	3469	Helicase	-5.99	40.807	Val^{496}, Arg^{497}, Phe^{499}, Leu^{500}, Thr^{501}, Trp^{506}, Arg^{507}, Ala^{509}, Leu^{526}, Tyr^{543}, Ile^{545}, Leu^{573}
			E-channel proteins	-5.56	84.32	Leu^{51}, Pro^{54}, Tyr^{57}
			Spike Open Protein	-5.89	47.99	Tyr^{38}, Asp^{40}, Lys^{41}, Val^{42}, Phe^{43}, Arg^{44}, Glu^{224}, Pro^{225}, Tyr^{279}, Gly^{283}
			Spike Close Protein	-6.25	26.09	Tyr^{38}, Asp^{40}, Lys^{41}, Val^{42}, Phe^{43}, Arg^{44}, Glu^{224}, Pro^{225}, Tyr^{279}, Gly^{283}, Thr^{284}
**59**	Glycyrrhisoflavone	5317764	Main Protease (MPro)	-7.65	2.49	Leu^{27}, His^{41}, Met^{49}, Asn^{142}, Gly^{143}, Cys^{145}, Met^{165}, Glu^{166}, Leu^{167}, Pro^{168}, Gly^{170}, Arg^{188}, Gln^{189}, Thr^{190}, Gln^{192}
			Papain like protease (PLPro)	-9.34	0.14	Cys^{155}, Asn^{156}, Lys^{157}, Leu^{162}, Gly^{163}, Asp^{164}, Glu^{167}, Tyr^{171}, Tyr^{268}, Gln^{269}
			RNA-dependent RNA polymerase (RdRP)	-8.01	1.34	Arg^{33}, Phe^{35}, Asp^{36}, Ile^{37}, Tyr^{38}, Asn^{39}, Lys^{50}, Lys^{73}, Thr^{123}, Thr^{206}, Asp^{208}, Asn^{209}, Tyr^{217}, Asp^{218}, Asp^{221}
			Helicase	-8.58	0.514	Asn^{177}, Asn^{179}, Glu^{201}, Lys^{202}, Gly^{203}, Asp^{204}, Val^{484}, Ser^{486}, Pro^{514}, Tyr^{515}, Asn^{516}, Ser^{517}, Asn^{519}, Ala^{520}, Thr^{530}, Thr^{532}, Val^{533}, Asp^{534}, Arg^{560}
			E-channel proteins	-8.06	1.23	Arg^{61}, Asn^{64}, Leu^{65}
			Spike Open Protein	-7.33	4.22	His^{1101}, Trp^{1102}, Phe^{1103}, Ile^{1114}, Ile^{1115}, Thr^{1116}, Asn^{1135}, Thr^{1136}, Tyr^{1138}, Pro^{1140}
			Spike Close Protein	-7.88	1.67	Arg^{102}, Trp^{104}, Ile^{119}, Asn^{121}, Val^{126}, Ile^{128}, Arg^{190}, Phe^{192}, Phe^{194}, Ile^{203}, Ser^{205}, Lys^{206}, His^{207}, Leu^{226}, Val^{227}
			Main Protease (MPro)	-9.57	0.11	Thr^{24}, Thr^{25}, Thr^{26}, Leu^{27}, Gly^{29}, His^{41}, Cys^{44}, Ser^{46}, Met^{49}, Tyr^{118}, Asn^{119},
						Asn^{142}, Gly^{143}, Cys^{145}, His^{163}, His^{164}, Met^{165}, Glu^{166}
				-9.46	0.76	Arg^{131}, Asn^{133}, Thr^{135}, Val^{137}, Thr^{169}, Val^{171}, Ala^{194}, Gly^{195}, Thr^{196}, Asp^{197},

Sr.	Ligands	PubChem ID	Proteins	Binding energy (kcal/mol)	Dissociation Constant (µM)	Active site residues
60	Glycyrrhizin	14982		[illegible]	[illegible]	Thr^{198}, Thr^{199}, Tyr^{237}, Asn^{238}, Tyr^{239}, Leu^{272}, Leu^{286}, Leu^{287}, Asp^{289}
			Papain like protease (PLPro)	-9.23	0.17	Gly^{209}, Thr^{210}, Leu^{211}, Ser^{212}, Tyr^{213}, Glu^{214}, Gln^{215}, Val^{220}, Gln^{221}, Ala^{249}, Gln^{250}, Tyr^{251}, Glu^{252}, Leu^{253}, Lys^{254}, Thr^{257}, Phe^{258}, Val^{303}, Tyr^{305}
			RNA Polymerase	-9.96	0.05	Asp^{452}, Tyr^{455}, Arg^{553}, Ala^{554}, Arg^{555}, Trp^{617}, Asp^{618}, Lys^{621}, Cys^{622}, Asp^{623},
						Arg^{624}, Ser^{759}, Asp^{760}, Asp^{761}, Lys^{798}, Trp^{800}, Glu^{811}, Cys^{813}, Ser^{814}
				-10.52	0.03	Asp^{452}, Tyr^{455}, Lys^{551}, Arg^{553}, Ala^{554}, Arg^{555}, Thr^{556}, Trp^{617}, Asp^{618}, Tyr^{619}, Pro^{620}, Lys^{621}, Cys^{622}, Asp^{623}, Arg^{624}, Ser^{759}, Asp^{760}, Asp^{761}, Ala^{762}, Lys^{798}, Cys^{799}, Trp^{800}, Glu^{811}, Phe^{812}, Cys^{813}, Ser^{814}
			Helicase	-11.57	0.003	Pro^{175}, Leu^{176}, Asn^{177}, Lys^{202}, Leu^{405}, Pro^{406}, Ala^{407}, Pro^{408}, Arg^{409}, Leu^{412}, Thr^{413}, Gly^{415}, Thr^{416}, Leu^{417}, Phe^{422}, Ser^{485}, Ser^{486}, Pro^{514}, Tyr^{515}, Asn^{516}, Asn^{519}, Thr^{532}, Val^{533}, Asp^{534}, His^{554}, Asn^{557}, Asn^{559}, Arg^{560}
			E-channel proteins	-10.07	0.04	Arg^{61}, Asn^{64}
			Spike Open Protein	-9.29	0.16	Val^{47}, His^{49}, Lys^{304}, Met^{740}, Tyr^{741}, Ile^{742}, Cys^{743}, Gly^{744}, Asp^{745}, Phe^{855}, Asn^{856}, Val^{963}, Lys^{964}, Leu^{966}, Ser^{967}, Ser^{975}, Val^{976}, Leu^{977}, Asn^{978}, Arg^{1000}
			Spike Close Protein	-9.49	0.11	Tyr^{741}, Ile^{742}, Cys^{743}, Gly^{744}, Asp^{745}, Phe^{855}, Asn^{856}, Val^{963}, Lys^{964}, Leu^{966}, Ser^{967}, Ser^{975}, Val^{976}, Leu^{977}, Asn^{978}, Arg^{1000}
			Main Protease (MPro)	-4.09	997.83	Trp^{218}, Phe^{219}, Leu^{220}, Asn^{221}, Arg^{222}, Phe^{223}, Ser^{267}, Glu^{270}, Leu^{271}, Asn^{274}, Gly^{275}, Met^{276}, Asn^{277}, Arg^{279}
			Papain like protease (PLPro)	-5.58	80.7	Arg^{65}, Val^{66}, Ala^{68}, Phe^{69}, Glu^{70}, Thr^{74}, Thr^{75}

Sr.	Ligands	PubChem ID	Proteins	Binding energy	Dissociation Constant	Active site residues
				(kcal/mol)	(µM)	
**61**	Heneicosanoic acid	16898	RNA-dependent RNA polymerase (RdRP)	-3.76	1744.55	Phe^{35}, Asp^{36}, Ile^{37}, Phe^{48}, Leu^{49}, Lys^{50}, Lys^{73}, Thr^{206}, Asp^{208}, Asn^{209}, Asp^{218}, Asp^{221}
			Helicase	-4.81	300.024	Ala^{4}, Val^{6}, Arg^{15}, Arg^{21}, Arg^{22}, Pro^{23}, Phe^{24}, Arg^{129}, Leu^{132}, Phe^{133}, Glu^{136}, Pro^{234}, Leu^{235}, Ser^{236}
			E-channel proteins	-5.67	69.68	No binding site was found
			Spike Open Protein	-3.45	2943.86	Pro^{426}, Asp^{428}, Phe^{429}, Thr^{430}, Phe^{464}, Ser^{514}, Phe^{515}, Glu^{516}, Leu^{517}, Leu^{518}
			Spike Close Protein	-4.45	547.15	Tyr^{38}, Asp^{40}, Lys^{41}, Val^{42}, Phe^{43}, Glu^{224}, Pro^{225}, Leu^{226}, Asn^{282}, Gly^{283}, Thr^{284}
**62**	Hesperetin	72281	Main Protease (MPro)	-7.33	4.22	Met^{49}, Phe^{140}, Leu^{141}, Asn^{142}, Gly^{143}, Ser^{144}, Cys^{145}, His^{163}, Met^{165}, Glu^{166}, Leu^{167}, Pro^{168}, His^{172}, Arg^{188}, Gln^{189}, Thr^{190}, Gln^{192}
			Papain like protease (PLPro)	-8.34	0.77	Cys^{155}, Asn^{156}, Lys^{157}, Glu^{161}, Leu^{162}, Gly^{163}, Glu^{167}, Tyr^{171}
			RNA-dependent RNA polymerase (RdRP)	-7.59	2.74	Thr^{591}, Ser^{592}, Lys^{593}, Phe^{594}, Gln^{815}, Pro^{830}, Tyr^{831}, Pro^{832}, Asp^{865}, Pro^{868}, Trp^{916}
			Helicase	-7.8	1.933	Asn^{177}, Asn^{179}, Tyr^{180}, Leu^{405}, Pro^{406}, Pro^{408}, Arg^{409}, Thr^{410}, Leu^{411}, Leu^{412}, Thr^{413}, Thr^{416}, Leu^{417}, Phe^{422}, Asn^{557}, Asn^{559}, Arg^{560}
			E-channel proteins	-7.42	3.62	Ile^{46}, Ser^{50}, Leu^{51}, Pro^{54}, Phe^{56}, Tyr^{57}, Ser^{60}
			Spike Open Protein	-6.88	9.1	Arg^{355}, Asn^{394}, Tyr^{396}, Pro^{426}, Asp^{428}, Phe^{429}, Thr^{430}, Phe^{464}, Ser^{514}, Phe^{515}, Glu^{516}, Leu^{517}, Leu^{518}
			Spike Close Protein	-7.1	6.28	Met^{740}, Tyr^{741}, Ile^{742}, Cys^{743}, Gly^{744}, Asn^{856}, Val^{963}, Lys^{964}, Leu^{966}, Ser^{967}, Val^{976}, Leu^{977}, Asn^{978}, Arg^{1000}
			Main Protease (MPro)	-8.02	1.33	Thr^{24}, Thr^{25}, Thr^{26}, His^{41}, Thr^{45}, Ser^{46}, Met^{49}, Phe^{140}, Leu^{141}, Asn^{142}, Gly^{143}, Ser^{144}, Cys^{145}, His^{163}, His^{164}, Met^{165}, Glu^{166}, His^{172}, Gln^{189}

Sr.	Ligands	PubChem ID	Proteins	Binding energy (kcal/mol)	Dissociation Constant (µM)	Active site residues
63	Hesperidin	10621	Papain like protease (PLPro)	-10.61	0.02	Lys^{157}, Thr^{158}, Glu^{161}, Leu^{162}, Gly^{163}, Asp^{164}, Val^{165}, Glu^{167}, Leu^{199}, Glu^{203}, Tyr^{207}, Met^{208}, Lys^{232}, Pro^{248}, Tyr^{264}, Tyr^{268}, Gln^{269}, Cys^{270}, Tyr^{273}, Thr^{301}
			RNA-dependent RNA polymerase (RdRP)	-9.53	0.1	Val^{166}, Tyr^{456}, Met^{542}, Arg^{553}, Ala^{554}, Arg^{555}, Thr^{556}, Val^{557}, Ala^{558}, Asp^{618}, Tyr^{619}, Pro^{620}, Lys^{621}, Cys^{622}, Asp^{623}, Arg^{624}, Lys^{676}, Thr^{680}, Ser^{681}, Ser^{682}, Phe^{793}, Ser^{795}, Lys^{798}
			Helicase	-8.93	0.283	Lys^{139}, Glu^{142}, Glu^{143}, Lys^{146}, Arg^{178}, Asn^{179}, Val^{181}, Glu^{197}, Thr^{228}, His^{230}, Cys^{309}, Arg^{337}, Arg^{339}, Met^{378}, Ala^{379}, Thr^{380}, Tyr^{382}, Asp^{383}, Ala^{407}, Pro^{408}, Thr^{410}
			E-channel proteins	-8.82	0.34	Leu^{28}, Val^{29}, Leu^{31}, Ala^{32}, Leu^{34}, Thr^{35}, Ala^{36}, Cys^{40}
			Spike Open Protein	-8.18	1.01	Asn^{710}, Thr^{1076}, Ser^{1097}, Gly^{1099}, Thr^{1100}, His^{1101}, Trp^{1102}, Ile^{1114}, Ile^{1115}, Val^{1133}, Asn^{1134}, Asn^{1135}, Thr^{1136}, Tyr^{1138}
			Spike Close Protein	-8.53	0.56	Tyr^{38}, Asp^{40}, Lys^{41}, Val^{42}, Phe^{43}, Arg^{44}, Lys^{206}, Phe^{220}, Ser^{221}, Ala^{222}, Glu^{224}, Pro^{225}, Leu^{226}, Tyr^{279}, Gly^{283}, Thr^{284}
64	Homogentisic acid	780	Main Protease (MPro)	8.99	8.99	Leu^{51}, Pro^{54}, Tyr^{57}
			Papain like protease (PLPro)	-6.59	14.87	No binding site was found
			RNA-dependent RNA polymerase (RdRP)	-6.65	13.31	Pro^{323}, Thr^{324}, Phe^{326}, Gly^{327}, Tyr^{346}, His^{347}, Phe^{348}, Arg^{349}, Glu^{350}, Asn^{628}, Ser^{664}, Val^{675}, Lys^{676}, Pro^{677}
			Helicase	-6.2	28.774	Val^{496}, Phe^{499}, Leu^{500}, Thr^{501}, Asn^{503}, Trp^{506}, Arg^{507}, Ala^{509}, Leu^{526}, Leu^{528}, Tyr^{543}, Ile^{545}, Leu^{573}
			E-channel proteins	-5.52	89.3	Leu^{51}, Pro^{54}, Tyr^{57}
			Spike Open Protein	-5.09	187.35	Gly^{885}, Trp^{886}, Thr^{887}, Ile^{896}, Met^{900}, Gln^{901}, Ala^{903}, Tyr^{904}, Arg^{905}
			Spike Close Protein	-6	39.85	Tyr^{38}, Asp^{40}, Lys^{41}, Val^{42}, Phe^{43}, Arg^{44}, Glu^{224}, Pro^{225}, Tyr^{279}, Gly^{283}, Thr^{284}

Sr.	Ligands	PubChem ID	Proteins	Binding energy (kcal/mol)	Dissociation Constant (µM)	Active site residues
**65**	Hydroxytyrosol	82755	Main Protease (MPro)	-6.48	17.73	Met^{6}, Ala^{7}, Phe^{8}, Gln^{127}, Phe^{291}, Asp^{295}, Arg^{298}, Gln^{299}, Gly^{302}, Val^{303}
			Papain like protease (PLPro)	-6.34	22.57	Gln^{174}, His^{175}, Val^{202}
			RNA-dependent RNA polymerase (RdRP)	-5.59	80.29	Lys^{593}, Phe^{594}, Gln^{815}, Pro^{830}, Tyr^{831}, Pro^{832}, Asp^{865}, Pro^{868}, Trp^{916}
			Helicase	-5.38	114.062	Ala^{4}, Cys^{5}, Val^{6}, Arg^{21}, Pro^{23}, Phe^{24}, Arg^{129}, Leu^{132}, Phe^{133}, Glu^{136}
			E-channel proteins	-5.3	129.89	No binding site was found
			Spike Open Protein	-5.5	92.68	Tyr^{38}, Pro^{39}, Asp^{40}, Lys^{41}, Val^{42}, Phe^{43}, Arg^{44}, Glu^{224}, Pro^{225}, Tyr^{279}, Gly^{283}, Thr^{284}
			Spike Close Protein	-5.06	197.08	Met^{740}, Tyr^{741}, Ile^{742}, Cys^{743}, Gly^{744}, Asn^{856}, Leu^{966}, Ser^{967}, Ser^{975}, Val^{976}, Leu^{977}, Arg^{1000}
**66**	Isoliquiritin	5318591	Main Protease (MPro)	-7.87	1.69	Thr^{24}, Thr^{25}, Thr^{26}, His^{41}, Cys^{44}, Thr^{45}, Ser^{46}, Met^{49}, Asn^{142}, Gly^{143}, Cys^{145}, His^{164}, Met^{165}, Glu^{166}, Leu^{167}, Pro^{168}, Val^{186}, Arg^{188}, Gln^{189}, Thr^{190}, Ala^{191}, Gln^{192}
			Papain like protease (PLPro)	-9.12	0.21	Met^{208}, Asn^{267}, Tyr^{268}, Gln^{269}
			RNA-dependent RNA polymerase (RdRP)	-7.71	2.23	Tyr^{265}, Ile^{266}, Lys^{267}, Trp^{268}, Leu^{270}, Pro^{322}, Pro^{323}, Thr^{324}, Leu^{388}, Leu^{389}, Asp^{390}, Thr^{393}, Thr^{394}, Cys^{395}, Phe^{396}, Ser^{397}
			Helicase	-7.85	1.75	Asn^{177}, Asn^{179}, Leu^{405}, Pro^{406}, Ala^{407}, Pro^{408}, Arg^{409}, Leu^{412}, Thr^{416}, Leu^{417}, Phe^{422}, Pro^{514}, Tyr^{515}, Asn^{516}, Asn^{519}, Thr^{532}, Asp^{534}, Asn^{557}, Asn^{559}, Arg^{560}
			E-channel proteins	-8.42	0.68	Leu^{27}, Ile^{46}, Val^{47}, Ser^{50}, Leu^{51}, Pro^{54}, Phe^{56}, Tyr^{57}, Ser^{60}
			Spike Open Protein	-6.8	10.29	Asn^{30}, Thr^{33}, Phe^{58}, Phe^{59}, Ser^{60}, Ala^{288}, Val^{289}, Asp^{290}, Leu^{293}, Asp^{294}, Leu^{296}, Ser^{297}, Lys^{300}, Asn^{606}
			Spike Close Protein	-6.98	7.64	Ile^{105}, Leu^{118}, Val^{120}, Asn^{122}, Asn^{125}, Val^{127}, Lys^{129}, Phe^{133}, Gln^{134}, Phe^{135}, Cys^{136}, Pro^{139}, Phe^{140}, Leu^{141}, Val^{171}, Leu^{241}

Sr.	Ligands	PubChem ID	Proteins	Binding energy (kcal/mol)	Dissociation Constant (µM)	Active site residues
**67**	Isorhamnetin	5281654	Main Protease (MPro)	-7.52	3.08	Met^{6}, Ala^{7}, Phe^{8}, Pro^{9}, Ser^{113}, Gln^{127}, Asp^{295}, Arg^{298}, Gln^{299}, Gly^{302}, Val^{303}, Thr^{304}
			Papain like protease (PLPro)	-8.46	0.63	Arg^{166}, Ser^{170}, Leu^{199}, Val^{202}, Glu^{203}, Met^{206}, Tyr^{207}, Met^{208}, Lys^{232}
			RNA-dependent RNA polymerase (RdRP)	-7.42	3.66	Thr^{591}, Ser^{592}, Lys^{593}, Phe^{594}, Gln^{815}, Pro^{830}, Tyr^{831}, Pro^{832}, Asp^{865}, Pro^{868}, Trp^{916}
			Helicase	-7.51	3.116	Asn^{177}, Arg^{178}, Glu^{201}, Lys^{202}, Gly^{203}, Tyr^{515}, Asn^{516}, Asn^{519}, Ala^{520}, Thr^{530}, Thr^{532}, Asp^{534}
			E-channel proteins	-6.9	8.73	Leu^{28}, Leu^{31}, Ala^{32}, Thr^{35}, Ala^{36}, Arg^{38}
			Spike Open Protein	-6.62	14.14	Lys^{535}, Glu^{554}, Ser^{555}, Asn^{556}, Lys^{557}, Lys^{558}, Phe^{559}, Arg^{577}, Leu^{582}, Glu^{583}, Ile^{584}, Leu^{585}
			Spike Close Protein	-6.84	9.65	Met^{740}, Tyr^{741}, Ile^{742}, Cys^{743}, Gly^{744}, Lys^{854}, Asn^{856}, Val^{963}, Lys^{964}, Leu^{966}, Ser^{967}, Val^{976}, Leu^{977}, Asn^{978}, Arg^{1000}
**68**	Kaempferol	5280863	Main Protease (MPro)	-7.66	2.44	Met^{6}, Ala^{7}, Phe^{8}, Pro^{9}, Ser^{113}, Gln^{127}, Asp^{295}, Arg^{298}, Gln^{299}, Gly^{302}, Val^{303}, Thr^{304}
			Papain like protease (PLPro)	-8.13	1.1	Arg^{166}, Ser^{170}, Val^{202}, Glu^{203}, Met^{206}, Tyr^{207}, Met^{208}, Lys^{232}
			RNA-dependent RNA polymerase (RdRP)	-7.14	5.83	Tyr^{32}, Lys^{47}, Tyr^{129}, His^{133}, Asp^{135}, Asn^{138}, Lys^{780}, Asn^{781}, Ser^{784}
			Helicase	-7.48	3.317	Ala^{135}, Leu^{138}, Lys^{139}, Glu^{142}, Asn^{179}, Thr^{380}, Tyr^{382}, Asp^{383}, Ala^{407}, Pro^{408}, Arg^{409}, Thr^{410}
			E-channel proteins	-7.02	7.13	No binding site was found
			Spike Open Protein	-6.82	9.97	Lys^{535}, Glu^{554}, Ser^{555}, Asn^{556}, Lys^{557}, Lys^{558}, Phe^{559}, Arg^{577}, Leu^{582}, Glu^{583}, Ile^{584}, Leu^{585}
			Spike Close Protein	-7.31	4.37	Leu^{335}, Cys^{336}, Phe^{338}, Gly^{339}, Phe^{342}, Asn^{343}, Ala^{363}, Asp^{364}, Val^{367}, Leu^{368}, Ser^{371}, Ser^{373}, Phe^{374}
			Main Protease (MPro)	-5.99	41.01	Phe^{8}, Lys^{102}, Val^{104}, Arg^{105}, Ile^{106}, Gln^{107}, Gln^{110}, Thr^{111}, Phe^{112}, Asn^{151}, Ile^{152}, Asp^{153}, Ser^{158}, Phe^{294}, Arg^{298}

Sr.	Ligands	PubChem ID	Proteins	Binding energy (kcal/mol)	Dissociation Constant (µM)	Active site residues
**69**	Kanzonol Y	10001604	Papain like protease (PLPro)	-9.37	0.14	Cys^{155}, Asn^{156}, Lys^{157}, Glu^{161}, Leu^{162}, Gly^{163}, Asp^{164}, Glu^{167}, Tyr^{171}, Tyr^{268}, Gln^{269}
			RNA-dependent RNA polymerase (RdRP)	-7.06	6.68	Ala^{46}, Lys^{47}, Tyr^{129}, Ala^{130}, His^{133}, Phe^{134}, Asp^{135}, Asn^{138}, Cys^{139}, Thr^{141}, Leu^{142}, Ala^{706}, Ser^{709}, Thr^{710}, Asp^{711}, Gln^{773}, Gly^{774}, Ser^{778}, Lys^{780}, Asn^{781}, Ser^{784}
			Helicase	-7.58	2.797	Ala^{4}, Cys^{5}, Val^{6}, Arg^{21}, Arg^{22}, Pro^{23}, Phe^{24}, Arg^{129}, Leu^{132}, Phe^{133}, Glu^{136}, Pro^{234}, Leu^{235}, Ser^{236}
			E-channel proteins	-7.8	1.91	Leu^{19}, Ala^{22}, Phe^{23}, Phe^{26}
			Spike Open Protein	-5.39	112.15	Trp^{886}, Gly^{889}, Ala^{890}, Tyr^{904}, Ser^{1030}, Glu^{1031}, Leu^{1034}, Gly^{1035}, Gln^{1036}, Ser^{1037}, Lys^{1038}
			Spike Close Protein	-6.94	8.14	Arg^{102}, Gly^{103}, Trp^{104}, Ile^{119}, Asn^{121}, Val^{126}, Ile^{128}, Tyr^{170}, Ser^{172}, Arg^{190}, Phe^{192}, Phe^{194}, Ile^{203}, Ser^{205}, His^{207}, Leu^{226}, Val^{227}
**70**	Lauric acid	3893	Main Protease (MPro)	-4.17	877.71	His^{41}, Ser^{46}, Met^{49}, Cys^{145}, His^{164}, Met^{165}, Glu^{166}, Leu^{167}, Pro^{168}, Arg^{188}, Gln^{189}, Thr^{190}, Gln^{192}
			Papain like protease (PLPro)	-5.28	133.9	Asp^{164}, Val^{165}, Arg^{166}, Met^{208}, Pro^{247}, Pro^{248}, Tyr^{264}, Gly^{266}, Asn^{267}, Tyr^{268}, Tyr^{273}, Thr^{301}
			RNA-dependent RNA polymerase (RdRP)	-4.74	335.94	Val^{335}, Asp^{336}, Val^{338}, Phe^{340}, Arg^{365}, Leu^{366}
			Helicase	-4.91	253.856	Pro^{406}, Ala^{407}, Pro^{408}, Arg^{409}, Leu^{412}, Thr^{413}, Gly^{415}, Thr^{416}, Leu^{417}, Glu^{418}, Pro^{419}, Phe^{422}, His^{554}, Asn^{557}, Asn^{559}, Arg^{560}
			E-channel proteins	-5.12	176.6	No binding site was found
			Spike Open Protein	-5.12	177.2	Asn^{99}, Arg^{102}, Gly^{103}, Trp^{104}, Ile^{119}, Asn^{121}, Val^{126}, Ile^{128}, Arg^{190}, Phe^{192}, Phe^{194}, Ile^{203}, Ser^{205}, His^{207}, Leu^{226}, Val^{227}
			Spike Close Protein	-5.08	187.98	Glu^{96}, Asn^{99}, Ile^{101}, Arg^{102}, Trp^{104}, Ile^{119}, Asn^{121}, Val^{126}, Ile^{128}, Arg^{190}, Phe^{192}, Phe^{194}, Ile^{203}, Leu^{226}, Val^{227}

Sr.	Ligands	PubChem ID	Proteins	Binding energy (kcal/mol)	Dissociation Constant (µM)	Active site residues
**71**	Linoleic acid	5280450	Main Protease (MPro)	-4.56	453.67	Trp^{218}, Phe^{219}, Leu^{220}, Asn^{221}, Arg^{222}, Phe^{223}, Ser^{267}, Glu^{270}, Leu^{271}, Asn^{274}, Gly^{275}, Met^{276}, Asn^{277}
			Papain like protease (PLPro)	-6.66	13.08	Lys^{157}, Leu^{162}, Gly^{163}, Asp^{164}, Met^{208}, Pro^{247}, Pro^{248}, Tyr^{264}, Asn^{267}, Tyr^{268}, Gln^{269}, Tyr^{273}, Thr^{301}
			RNA-dependent RNA polymerase (RdRP)	-5.24	144.71	Arg^{33}, Ala^{34}, Phe^{35}, Ile^{37}, Phe^{48}, Leu^{49}, Lys^{50}, Lys^{73}, Thr^{123}, Asp^{208}, Asn^{209}, Tyr^{217}, Asp^{218}, Asn^{713}
			Helicase	-5.02	208.717	Leu^{405}, Pro^{406}, Ala^{407}, Pro^{408}, Arg^{409}, Leu^{412}, Thr^{416}, Leu^{417}, Phe^{422}, His^{554}, Asn^{557}, Asn^{559}, Arg^{560}
			E-channel proteins	-6.24	26.71	No binding site was found
			Spike Open Protein	-4.23	797.2	Arg^{355}, Asn^{394}, Tyr^{396}, Pro^{426}, Phe^{429}, Thr^{430}, Pro^{463}, Phe^{464}, Ser^{514}, Phe^{515}, Glu^{516}, Leu^{517}
			Spike Close Protein	-5.68	69.09	Trp^{104}, Ile^{119}, Asn^{121}, Val^{126}, Ile^{128}, Phe^{168}, Tyr^{170}, Ser^{172}, Arg^{190}, Phe^{192}, Phe^{194}, Ile^{203}, Leu^{226}, Val^{227}, Leu^{229}
**72**	Linolenic acid	5280934	Main Protease (MPro)	-4.81	299.52	Trp^{218}, Phe^{219}, Leu^{220}, Asn^{221}, Arg^{222}, Phe^{223}, Ser^{267}, Glu^{270}, Leu^{271}, Asn^{274}, Gly^{275}, Met^{276}, Asn^{277}, Arg^{279}
			Papain like protease (PLPro)	-7.03	7.01	Asp^{164}, Pro^{247}, Pro^{248}, Tyr^{264}, Asn^{267}, Tyr^{268}, Tyr^{273}
			RNA-dependent RNA polymerase (RdRP)	-5.23	145.69	Phe^{35}, Asp^{36}, Ile^{37}, Tyr^{38}, Asn^{39}, Val^{42}, Phe^{48}, Leu^{49}, Lys^{50}, Thr^{206}, Asp^{208}, Asn^{713}
			Helicase	-5.25	141.807	Ala^{4}, Cys^{5}, Val^{6}, Arg^{15}, Arg^{21}, Arg^{22}, Pro^{23}, Phe^{24}, Arg^{129}, Leu^{132}, Phe^{133}, Glu^{136}, Pro^{234}, Leu^{235}, Ser^{236}
			E-channel proteins	-6.45	18.58	No binding site was found
			Spike Open Protein	-4.68	369.87	Leu^{390}, Cys^{391}, Leu^{517}, His^{519}, Phe^{543}, Asn^{544}, Gly^{545}, Leu^{546}, Thr^{547}, Gln^{564}, Phe^{565}, Thr^{573}, Val^{576}, Arg^{577}, Pro^{579}
			Spike Close Protein	-5.32	127.07	Val^{382}, Leu^{390}, Cys^{391}, Phe^{392}, Thr^{393}, Leu^{517}, Leu^{518}, His^{519}, Ala^{520}, Ala^{522}, Phe^{543}, Asn^{544}, Gly^{545}, Leu^{546}, Thr^{547}, Phe^{565}

Sr.	Ligands	PubChem ID	Proteins	Binding energy (kcal/mol)	Dissociation Constant (µM)	Active site residues
**73**	Liquiritigenin	114829	Main Protease (MPro)	-7.04	6.89	Met^{6}, Ala^{7}, Phe^{8}, Pro^{9}, Ser^{113}, Gln^{127}, Asp^{295}, Arg^{298}, Gln^{299}, Gly^{302}, Val^{303}, Thr^{304}
			Papain like protease (PLPro)	-9.11	0.21	Thr^{74}, Thr^{75}, Asp^{76}, Tyr^{154}, Tyr^{171}, Gln^{174}, His^{175}
			RNA-dependent RNA polymerase (RdRP)	-6.95	8.03	Phe^{407}, Gln^{408}, Thr^{409}, Asp^{445}, Gly^{446}, Asn^{447}
			Helicase	-7.2	5.25	Asn^{179}, Leu^{405}, Pro^{406}, Ala^{407}, Pro^{408}, Arg^{409}, Leu^{412}, Thr^{416}, Leu^{417}, Phe^{422}, Asp^{534}, Asn^{557}, Asn^{559}, Arg^{560}
			E-channel proteins	-7.44	3.54	No binding site was found
			Spike Open Protein	-6.94	8.2	Tyr^{38}, Asp^{40}, Lys^{41}, Val^{42}, Phe^{43}, Arg^{44}, Lys^{206}, Glu^{224}, Pro^{225}, Tyr^{279}, Gly^{283}, Thr^{284}
			Spike Close Protein	-7.13	5.93	Phe^{329}, Pro^{330}, Asn^{331}, Ile^{332}, Thr^{333}, Leu^{335}, Val^{362}, Cys^{525}, Gly^{526}, Pro^{527}, Lys^{528}, Lys^{529}, Ser^{530}, Gln^{580}
**74**	Luteolin	5280445	Main Protease (MPro)	-7.62	2.59	Met^{6}, Ala^{7}, Phe^{8}, Pro^{9}, Ser^{113}, Gln^{127}, Asp^{295}, Arg^{298}, Gln^{299}, Gly^{302}, Val^{303}, Thr^{304}
			Papain like protease (PLPro)	-8.47	0.62	Cys^{155}, Asn^{156}, Lys^{157}, Glu^{161}, Leu^{162}, Gly^{163}, Glu^{167}, Tyr^{171}
			RNA-dependent RNA polymerase (RdRP)	-7.84	1.79	Phe^{407}, Thr^{409}, Asp^{445}, Gly^{446}, Asn^{447}
			Helicase	-7.83	1.831	Asn^{179}, Tyr^{180}, Leu^{405}, Pro^{406}, Pro^{408}, Arg^{409}, Thr^{410}, Leu^{411}, Leu^{412}, Thr^{413}, Thr^{416}, Leu^{417}, Phe^{422}, Asn^{557}, Asn^{559}, Arg^{560}
			E-channel proteins	-7.36	4.04	Ile^{46}, Ser^{50}, Leu^{51}, Pro^{54}, Phe^{56}, Tyr^{57}
			Spike Open Protein	-7.05	6.83	Arg^{355}, Asn^{394}, Tyr^{396}, Pro^{426}, Asp^{428}, Phe^{429}, Thr^{430}, Phe^{464}, Ser^{514}, Phe^{515}, Glu^{516}, Leu^{517}, Leu^{518}
			Spike Close Protein	-7.28	4.64	Val^{382}, Leu^{390}, Cys^{391}, Phe^{392}, Thr^{393}, Glu^{516}, Leu^{517}, Leu^{518}, His^{519}, Ala^{520}, Pro^{521}, Ala^{522}, Asn^{544}, Gly^{545}, Leu^{546}, Thr^{547}, Phe^{565}
			Main Protease (MPro)	-4.65	391.05	Ile^{106}, Gln^{110}, Thr^{111}, Asn^{151}, Thr^{292}, Phe^{294}, Asp^{295}, Arg^{298}
			Papain like protease (PLPro)	-6.01	39.06	Asp^{164}, Tyr^{268}

Sr.	Ligands	PubChem ID	Proteins	Binding energy (kcal/mol)	Dissociation Constant (µM)	Active site residues
**75**	Malic acid	525	RNA-dependent RNA polymerase (RdRP)	-5.5	92.52	Arg^{305}, Arg^{467}, Leu^{470}, Phe^{471}, Glu^{474}, Cys^{730}, Leu^{731}, Arg^{733}, Asn^{734}, Arg^{735}, Asp^{736}, Val^{737}
			Helicase	-5.63	75.177	Tyr^{120}, Ala^{123}, Asn^{124}, Lys^{131}, Ala^{135}, Leu^{138}, Thr^{380}, Asn^{381}, Tyr^{382}, Arg^{409}, Tyr^{421}, Phe^{422}, Asn^{423}
			E-channel proteins	-4.05	1078.39	No binding site was found
			Spike Open Protein	-4.82	294.5	Ser^{730}, Thr^{778}, Pro^{863}, Leu^{865}, Asp^{867}, Ile^{870}, Ala^{1056}, Pro^{1057}, His^{1058}
			Spike Close Protein	-4.88	266.59	Leu^{390}, Cys^{391}, Leu^{517}, Leu^{518}, His^{519}, Ala^{520}, Ala^{522}, Phe^{543}, Asn^{544}, Gly^{545}, Leu^{546}, Phe^{565}
**76**	Margaric acid	10465	Main Protease (MPro)	-3.91	1354.36	Trp^{218}, Phe^{219}, Leu^{220}, Asn^{221}, Arg^{222}, Phe^{223}, Ser^{267}, Glu^{270}, Leu^{271}, Asn^{274}, Gly^{275}, Asn^{277}
			Papain like protease (PLPro)	-5.91	46.79	Asp^{164}, Pro^{247}, Pro^{248}, Tyr^{264}, Tyr^{268}, Tyr^{273}, Thr^{301}
			RNA-dependent RNA polymerase (RdRP)	-4.62	408.6	Glu^{474}, Asp^{477}, Lys^{478}, Phe^{480}, Asp^{481}, Val^{637}, Arg^{640}, Lys^{641}, Thr^{643}, Val^{737}, Thr^{739}, Val^{742}, Asn^{743}
			Helicase	-4.72	345.14	Ala^{4}, Val^{6}, Arg^{15}, Arg^{21}, Arg^{22}, Pro^{23}, Phe^{24}, Arg^{129}, Leu^{132}, Phe^{133}, Glu^{136}, Pro^{234}, Leu^{235}, Ser^{236}
			E-channel proteins	-5.51	92.21	No binding site was found
			Spike Open Protein	-3.7	1943.56	Asn^{394}, Tyr^{396}, Pro^{426}, Asp^{428}, Phe^{429}, Thr^{430}, Pro^{463}, Phe^{464}, Ser^{514}, Phe^{515}, Glu^{516}, Leu^{517}, Leu^{518}
			Spike Close Protein	-5.04	203.5	Trp^{104}, Ile^{119}, Val^{126}, Ile^{128}, Phe^{168}, Tyr^{170}, Ser^{172}, Phe^{192}, Phe^{194}, Ile^{203}, Leu^{226}, Val^{227}, Asp^{228}, Leu^{229}
			Main Protease (MPro)	-7.53	3.03	Met^{49}, Phe^{140}, Leu^{141}, Asn^{142}, Gly^{143}, Ser^{144}, Cys^{145}, His^{163}, Met^{165}, Glu^{166}, Leu^{167}, Pro^{168}, His^{172}, Arg^{188}, Gln^{189}, Thr^{190}, Gln^{192}
			Papain like protease (PLPro)	-9.65	0.09	Thr^{74}, Thr^{75}, Asp^{76}, Arg^{82}, Tyr^{154}, Asn^{156}, Tyr^{171}, Gln^{174}, His^{175}

Sr.	Ligands	PubChem ID	Proteins	Binding energy (kcal/mol)	Dissociation Constant (µM)	Active site residues
**77**	Myricetin	5281672	RNA-dependent RNA polymerase (RdRP)	-7.66	2.44	Asp^{452}, Tyr^{456}, Arg^{553}, Ala^{554}, Arg^{555}, Thr^{556}, Val^{557}, Ala^{558}, Lys^{621}, Cys^{622}, Asp^{623}, Arg^{624}, Lys^{676}, Thr^{680}, Ser^{681}, Ser^{682}, Gly^{683}
			Helicase	-7.68	2.331	Asn^{177}, Arg^{178}, Glu^{201}, Tyr^{515}, Asn^{516}, Asn^{519}, Ala^{520}, Thr^{530}, Gln^{531}, Thr^{532}, Asp^{534}
			E-channel proteins	-6.85	9.49	Leu^{28}, Leu^{31}, Ala^{32}, Thr^{35}, Ala^{36}, Arg^{38}
			Spike Open Protein	-7.12	6.05	Ser^{730}, Met^{731}, Thr^{732}, Lys^{733}, Thr^{778}, Val^{860}, Leu^{861}, Pro^{862}, Pro^{863}, Asp^{867}, Ile^{870}, Ala^{1056}, Pro^{1057}, His^{1058}, Gly^{1059}
			Spike Close Protein	-7.33	4.22	Met^{740}, Tyr^{741}, Ile^{742}, Cys^{743}, Gly^{744}, Asn^{856}, Val^{963}, Lys^{964}, Leu^{966}, Ser^{967}, Ser^{975}, Val^{976}, Leu^{977}, Asn^{978}, Arg^{1000}
**78**	Myristic acid	11005	Main Protease (MPro)	-4.03	1107.91	Trp^{218}, Phe^{219}, Leu^{220}, Asn^{221}, Phe^{223}, Ser^{267}, Glu^{270}, Leu^{271}, Asn^{274}, Gly^{275}, Met^{276}, Asn^{277}, Arg^{279}
			Papain like protease (PLPro)	-5.52	89.3	No binding site was found
			RNA-dependent RNA polymerase (RdRP)	-4.45	548.07	Phe^{35}, Asp^{36}, Ile^{37}, Asn^{39}, Val^{42}, Phe^{48}, Lys^{50}, Thr^{206}, Asp^{208}, Asn^{209}, Asp^{218}, Asp^{221}, Asn^{713}
			Helicase	-4.66	386.462	Leu^{405}, Pro^{406}, Ala^{407}, Pro^{408}, Arg^{409}, Leu^{412}, Thr^{416}, Leu^{417}, Phe^{422}, Asn^{557}, Asn^{559}, Arg^{560}
			E-channel proteins	-4.85	280.44	No binding site was found
			Spike Open Protein	-3.71	1923.97	His^{1101}, Trp^{1102}, Phe^{1103}, Ile^{1114}, Ile^{1115}, Asn^{1135}, Thr^{1136}, Tyr^{1138}
			Spike Close Protein	-4.9	256.87	Tyr^{38}, Asp^{40}, Lys^{41}, Val^{42}, Phe^{43}, Arg^{44}, Glu^{224}, Pro^{225}, Tyr^{279}, Asn^{282}, Gly^{283}, Thr^{284}
			Main Protease (MPro)	-4.51	496.96	Phe^{8}, Val^{104}, Arg^{105}, Ile^{106}, Gln^{107}, Gln^{110}, Thr^{111}, Asn^{151}, Asp^{153}, Ser^{158}, Thr^{292}, Phe^{294}, Asp^{295}, Arg^{298}
			Papain like protease (PLPro)	-5.82	54.46	Asp^{164}, Arg^{166}, Met^{208}, Ser^{245}, Ala^{246}, Pro^{247}, Pro^{248}, Tyr^{268}, Thr^{301}, Asp^{302}

Sr.	Ligands	PubChem ID	Proteins	Binding energy (kcal/mol)	Dissociation Constant (µM)	Active site residues
79	Myristoleic acid	5281119	RNA-dependent RNA polymerase (RdRP)	-5.32	125.16	Val^{335}, Asp^{336}, Val^{338}, Phe^{340}, Leu^{366}
			Helicase	-4.86	273.426	Pro^{406}, Ala^{407}, Pro^{408}, Arg^{409}, Leu^{412}, Thr^{416}, Leu^{417}, Pro^{419}, Phe^{422}, His^{554}, Asn^{557}, Asn^{559}, Arg^{560}
			E-channel proteins	-5.48	95.7	No binding site was found
			Spike Open Protein	-5.28	135.26	Ser^{94}, Glu^{96}, Ile^{101}, Arg^{102}, Gly^{103}, Trp^{104}, Ile^{119}, Asn^{121}, Val^{126}, Ile^{128}, Arg^{190}, Phe^{192}, Phe^{194}, Ile^{203}, Ser^{205}, His^{207}, Leu^{226}, Val^{227}
			Spike Close Protein	-5.43	104.48	Glu^{96}, Asn^{99}, Ile^{101}, Arg^{102}, Trp^{104}, Ile^{119}, Asn^{121}, Val^{126}, Ile^{128}, Arg^{190}, Phe^{192}, Phe^{194}, Ile^{203}, Ser^{205}, Leu^{226}, Val^{227}
80	Naringen	442428	Main Protease (MPro)	-8.05	1.26	Thr^{24}, Thr^{25}, Thr^{26}, Leu^{27}, His^{41}, Thr^{45}, Ser^{46}, Met^{49}, Asn^{142}, Gly^{143}, Ser^{144}, Cys^{145}, His^{164}, Met^{165}, Glu^{166}, Leu^{167}, Pro^{168}, Arg^{188}, Gln^{189}, Thr^{190}, Gln^{192}
			Papain like protease (PLPro)	-10.17	0.04	Cys^{155}, Asn^{156}, Glu^{161}, Leu^{162}, Gly^{163}, Asp^{164}, Arg^{166}, Gln^{167}, Ser^{170}, Leu^{185},
						Leu^{199}, Val^{202}, Glu^{203}, Met^{206}, Tyr^{207}, Met^{208}, Ile^{222}, Pro^{223}, Lys^{232}, Tyr^{268}
			RNA-dependent RNA polymerase (RdRP)	-8.54	0.55	Tyr^{32}, Lys^{47}, Tyr^{129}, His^{133}, Phe^{134}, Asp^{135}, Asn^{138}, Ser^{709}, Thr^{710}, Asp^{711}, Lys^{714}, Ala^{771}, Ser^{772}, Gln^{773}, Gly^{774}, Ser^{778}, Lys^{780}, Asn^{781}, Ser^{784}
			Helicase	-8.42	0.673	Asn^{177}, Asn^{179}, Tyr^{180}, Pro^{408}, Arg^{409}, Thr^{410}, Leu^{412}, Thr^{413}, Gly^{415}, Thr^{416}, Leu^{417}, Pro^{514}, Tyr^{515}, Asn^{516}, Asn^{519}, Thr^{532}, Val^{533}, Asp^{534}, His^{554}, Ser^{555}, Arg^{560}
			E-channel proteins	-8.53	0.56	Phe^{23}, Phe^{26}, Leu^{27}, Val^{29}, Thr^{30}, Leu^{31}, Ile^{33}, Leu^{34}, Leu^{39}, Cys^{43}, Ile^{46}, Val^{47}

Sr.	Ligands	PubChem ID	Proteins	Binding energy (kcal/mol)	Dissociation Constant (µM)	Active site residues
			Spike Open Protein	-7.52	3.07	Pro^{330}, Asn^{331}, Ile^{332}, Thr^{333}, Asn^{334}, Asn^{360}, Pro^{521}, Ala^{522}, Thr^{523}, Asn^{544}, Gln^{564}, Arg^{577}, Asp^{578}, Pro^{579}, Gln^{580}, Thr^{581}, Leu^{582}
			Spike Close Protein	-7.64	2.52	His^{49}, Met^{740}, Tyr^{741}, Ile^{742}, Cys^{743}, Gly^{744}, Asn^{856}, Val^{963}, Lys^{964}, Leu^{966}, Ser^{967}, Ser^{975}, Val^{976}, Leu^{977}, Asn^{978}, Arg^{1000}
**81**	Naringenin	932	Main Protease (MPro)	-7.36	4.01	Met^{6}, Ala^{7}, Phe^{8}, Pro^{9}, Gln^{127}, Asp^{295}, Arg^{298}, Gln^{299}, Val^{303}, Thr^{304}
			Papain like protease (PLPro)	-9.36	0.14	Phe^{69}, His^{73}, Thr^{74}, Thr^{75}, Asp^{76}, Phe^{79}, Arg^{82}, Ala^{153}, Tyr^{154}, Asn^{156}, Gln^{174}, His^{175}
			RNA-dependent RNA polymerase (RdRP)	-7.42	3.62	Thr^{591}, Ser^{592}, Lys^{593}, Phe^{594}, Gln^{815}, Pro^{830}, Tyr^{831}, Pro^{832}, Asp^{865}, Pro^{868}, Trp^{916}
			Helicase	-7.45	3.478	Pro^{175}, Leu^{176}, Asn^{177}, Glu^{201}, Lys^{202}, Gly^{203}, Asp^{204}, Asn^{516}, Asn^{519}, Ala^{520}, Thr^{530}, Thr^{532}
			E-channel proteins	-7.4	3.75	Ile^{46}, Leu^{51}, Pro^{54}, Phe^{56}, Tyr^{57}
			Spike Open Protein	-6.61	14.4	Ser^{730}, Thr^{732}, Thr^{778}, Phe^{823}, Val^{860}, Pro^{862}, Pro^{863}, Asp^{867}, Ile^{870}, Ala^{1056}, Pro^{1057}, His^{1058}, Gly^{1059}
			Spike Close Protein	-6.95	8.03	Tyr^{38}, Asp^{40}, Lys^{41}, Val^{42}, Phe^{43}, Arg^{44}, Glu^{224}, Pro^{225}, Leu^{226}, Tyr^{279}, Gly^{283}
			Main Protease (MPro)	-8.04	1.28	Thr^{25}, Leu^{27}, His^{41}, Cys^{44}, Thr^{45}, Ser^{46}, Met^{49}, Asn^{142}, Gly^{143}, Cys^{145}, His^{164}, Met^{165}, Glu^{166}, Leu^{167}, Pro^{168}, Arg^{188}, Gln^{189}, Thr^{190}, Gln^{192}
			Papain like protease (PLPro)	-8.81	0.35	Arg^{183}, Leu^{185}, Val^{187}, Gln^{195}, Thr^{197}, Leu^{199}, Glu^{203}, Met^{206}, Tyr^{207}, Met^{208}, Ile^{222}, Thr^{225}, Lys^{232}
**82**	Neochlorogenic acid	5280633	RNA-dependent RNA polymerase (RdRP)	-7.82	1.87	Tyr^{32}, Ala^{46}, Lys^{47}, Tyr^{129}, Ala^{130}, His^{133}, Phe^{134}, Asp^{135}, Asn^{138}, Cys^{139}, Asp^{140}, Thr^{141}, Leu^{142}, Ser^{709}, Thr^{710}, Asp^{711}, Lys^{714}

Sr.	Ligands	PubChem ID	Proteins	Binding energy (kcal/mol)	Dissociation Constant (µM)	Active site residues
[illegible]	Neochlorogenic acid	[illegible]	Helicase	-7.79	1.962	Lys^{139}, Glu^{142}, Glu^{143}, Lys^{146}, Asn^{179}, Tyr^{180}, Val^{181}, Glu^{197}, Thr^{214}, His^{230}, Ala^{336}, Arg^{337}, Arg^{339}, Thr^{410}
			E-channel proteins	-7.73	2.15	Ile^{46}, Ser^{50}, Leu^{51}, Pro^{54}, Phe^{56}, Tyr^{57}, Ser^{60}, Arg^{61}
			Spike Open Protein	-6.57	15.33	Tyr^{38}, Asp^{40}, Lys^{41}, Val^{42}, Phe^{43}, Arg^{44}, Lys^{206}, Glu^{224}, Pro^{225}, Tyr^{279}, Gly^{283}, Thr^{284}
			Spike Close Protein	-7.38	3.9	Tyr^{38}, Asp^{40}, Lys^{41}, Val^{42}, Phe^{43}, Arg^{44}, Glu^{224}, Pro^{225}, Leu^{226}, Val^{227}, Asp^{228}, Tyr^{279}, Gly^{283}
**83**	Nishindaside	10310589	Main Protease (MPro)	-7.55	2.91	His^{41}, Met^{49}, Phe^{140}, Leu^{141}, Asn^{142}, Ser^{144}, Cys^{145}, His^{163}, His^{164}, Met^{165}, Glu^{166}, Leu^{167}, Pro^{168}, Arg^{188}, Gln^{189}, Thr^{190}, Gln^{192}
			Papain like protease (PLPro)	-7.53	3.03	Asp^{108}, Asn^{109}, Gly^{160}, Glu^{161}, Leu^{162}, Gln^{269}
			RNA-dependent RNA polymerase (RdRP)	-6.75	11.28	Tyr^{129}, His^{133}, Phe^{134}, Asp^{135}, Asn^{138}, Ser^{709}, Thr^{710}, Ala^{771}, Ser^{772}, Gln^{773}, Gly^{774}, Ser^{778}, Lys^{780}, Asn^{781}, Ser^{784}
			Helicase	-7.19	5.412	Gly^{3}, Ala^{4}, Cys^{5}, Val^{6}, Arg^{21}, Arg^{22}, Pro^{23}, Phe^{24}, Arg^{129}, Leu^{130}, Leu^{132}, Phe^{133}, Glu^{136}, Pro^{234}, Leu^{235}, Ser^{236}
			E-channel proteins	-7.04	6.9	Leu^{31}, Ala^{32}, Thr^{35}, Ala^{36}, Arg^{38}, Cys^{40}
			Spike Open Protein	-5.94	43.95	Asp^{364}, Tyr^{365}, Ser^{366}, Tyr^{369}, Asn^{370}, Pro^{384}, Thr^{385}, Leu^{387}, Asn^{388}, Pro^{527}, Lys^{528}
			Spike Close Protein	-7.14	5.83	Glu^{96}, Asn^{99}, Ile^{101}, Arg^{102}, Gly^{103}, Trp^{104}, Ile^{119}, Val^{120}, Asn^{121}, Val^{126}, Ile^{128}, Tyr^{170}, Ser^{172}, Arg^{190}, Phe^{192}, Val^{227}
			Main Protease (MPro)	-7.43	3.57	His^{41}, Met^{49}, Phe^{140}, Leu^{141}, Asn^{142}, Ser^{144}, Cys^{145}, His^{163}, His^{164}, Met^{165}, Glu^{166}, Leu^{167}, Pro^{168}, Arg^{188}, Gln^{189}, Thr^{190}, Gln^{192}
			Papain like protease (PLPro)	-7.2	5.24	Tyr^{268}, Gln^{269}
			RNA-dependent RNA polymerase (RdRP)	-6.36	21.63	Tyr^{719}, Val^{720}, Leu^{723}, Asn^{743}, Glu^{744}, Phe^{745}, Ala^{747}, Tyr^{748}, Lys^{751}, His^{752}

Sr.	Ligands	PubChem ID	Proteins	Binding energy (kcal/mol)	Dissociation Constant (µM)	Active site residues
**84**	Nordihydroguaiaretic	4534	Helicase	-7.01	7.247	Gly^{3}, Ala^{4}, Val^{6}, Arg^{21}, Arg^{22}, Pro^{23}, Phe^{24}, Arg^{129}, Leu^{132}, Phe^{133}, Glu^{136}, Pro^{234}, Leu^{235}
			E-channel proteins	-6.57	15.23	Ile^{46}, Leu^{51}, Pro^{54}, Tyr^{57}
			Spike Open Protein	-6.13	32	Pro^{330}, Ile^{332}, Asn^{360}, Pro^{521}, Ala^{522}, Thr^{523}, Asn^{544}, Gln^{564}, Arg^{577}, Asp^{578}, Pro^{579}, Gln^{580}, Leu^{582}
			Spike Close Protein	-6.25	26.27	Met^{740}, Tyr^{741}, Ile^{742}, Cys^{743}, Gly^{744}, Asp^{745}, Phe^{855}, Asn^{856}, Leu^{966}, Ser^{975}, Val^{976}, Leu^{977}, Asn^{978}, Arg^{1000}
**85**	Oleic acid	445639	Main Protease (MPro)	-4.34	663.24	Thr^{199}, Tyr^{237}, Tyr^{239}, Leu^{271}, Leu^{272}, Gly^{275}, Met^{276}, Asn^{277}, Gly^{278}, Ala^{285}, Leu^{286}, Leu^{287}
			Papain like protease (PLPro)	-6.33	22.8	Asp^{164}, Pro^{247}, Pro^{248}, Tyr^{264}, Asn^{267}, Tyr^{268}, Tyr^{273}, Thr^{301}
			RNA-dependent RNA polymerase (RdRP)	-3.95	1268.08	Tyr^{129}, His^{133}, Phe^{134}, Asp^{135}, Asn^{705}, Ala^{706}, Ser^{709}, Thr^{710}, Ala^{771}, Gly^{774}, Ser^{778}, Lys^{780}, Asn^{781}, Ser^{784}
			Helicase	-4.49	515.764	Ala^{4}, Val^{6}, Asn^{9}, Arg^{21}, Arg^{22}, Pro^{23}, Phe^{24}, Arg^{129}, Leu^{132}, Phe^{133}, Glu^{136}, Pro^{234}, Leu^{235}, Ser^{236}
			E-channel proteins	-5.78	57.87	No binding site was found
			Spike Open Protein	-3.26	4084.33	Thr^{33}, Phe^{58}, Phe^{59}, Gln^{218}, Gly^{219}, Phe^{220}, Asp^{287}, Ala^{288}, Val^{289}, Asp^{290}, Leu^{293}, Asp^{294}, Leu^{296}, Ser^{297}, Lys^{300}, Asn^{606}
			Spike Close Protein	-5.06	194.76	Trp^{104}, Ile^{119}, Asn^{121}, Val^{126}, Ile^{128}, Arg^{190}, Phe^{192}, Phe^{194}, Ile^{203}, Ser^{205}, Lys^{206}, His^{207}, Leu^{226}, Val^{227}
			Main Protease (MPro)	-7.8	1.92	Thr^{25}, Thr^{26}, Leu^{27}, His^{41}, Thr^{45}, Ser^{46}, Met^{49}, Phe^{140}, Leu^{141}, Asn^{142}, Gly^{143}, Ser^{144}, Cys^{145}, His^{163}, His^{164}, Met^{165}, Glu^{166}, His^{172}, Gln^{189}
			Papain like protease (PLPro)	-8.5	0.58	Asp^{164}, Arg^{166}, Met^{208}, Ala^{246}, Pro^{247}, Pro^{248}, Tyr^{264}, Asn^{267}, Tyr^{268}, Tyr^{273}, Thr^{301}

Sr.	Ligands	PubChem ID	Proteins	Binding energy (kcal/mol)	Dissociation Constant (µM)	Active site residues
**86**	Oleuropein	5281544	RNA-dependent RNA polymerase (RdRP)	-8.31	0.81	Tyr^{32}, Lys^{47}, Phe^{48}, Tyr^{129}, Ala^{130}, His^{133}, Phe^{134}, Asp^{135}, Asn^{138}, Cys^{139}, Asp^{140}, Thr^{141}, Leu^{142}, Thr^{710}, Asp^{711}, Lys^{714}, Ser^{778}, Lys^{780}, Asn^{781}, Ser^{784}
			Helicase	-7.77	2.003	Ala^{4}, Val^{6}, Arg^{15}, Arg^{21}, Arg^{22}, Pro^{23}, Phe^{24}, Arg^{129}, Leu^{132}, Phe^{133}, Glu^{136}, Pro^{234}, Leu^{235}, Ser^{236}
			E-channel proteins	-8.3	0.82	Arg^{61}, Asn^{64}, Leu^{65}
			Spike Open Protein	-6.8	10.42	Pro^{330}, Asn^{331}, Ile^{332}, Thr^{333}, Asn^{334}, Asn^{360}, His^{519}, Pro^{521}, Ala^{522}, Thr^{523}, Asn^{544}, Gln^{564}, Arg^{577}, Asp^{578}, Pro^{579}, Leu^{582}
			Spike Close Protein	-7.12	6.05	Arg^{355}, Asn^{394}, Tyr^{396}, Pro^{426}, Asp^{427}, Asp^{428}, Phe^{429}, Thr^{430}, Pro^{463}, Phe^{464}, Ser^{514}, Phe^{515}, Glu^{516}, Leu^{518}
**87**	Oxalic acid	971	Main Protease (MPro)	-4.55	461.39	Met^{6}, Phe^{8}, Gln^{127}, Asp^{295}, Arg^{298}, Gln^{299}
			Papain like protease (PLPro)	-5.14	171.03	No binding site was found
			RNA-dependent RNA polymerase (RdRP)	-4.89	259.93	Phe^{440}, Phe^{441}, Phe^{442}, Gln^{444}, Lys^{545}, Tyr^{546}, Ala^{547}, Ser^{549}, Lys^{551}, Asn^{552}, Arg^{553}, Ala^{554}, Arg^{555}
			Helicase	-4.98	222.918	Tyr^{120}, Ala^{123}, Asn^{124}, Leu^{138}, Asn^{381}, Arg^{409}, Leu^{417}, Tyr^{421}, Phe^{422}, Asn^{423}
			E-channel proteins	-3.24	4189.06	No binding site was found
			Spike Open Protein	-3.69	1983.32	Gln^{784}, Val^{785}, Lys^{786}, Gln^{787}, Tyr^{789}, Phe^{888}, Gly^{891}, Ala^{892}, Ala^{893}
			Spike Close Protein	-4.5	506.28	Asp^{420}, Tyr^{421}, Asn^{422}, Tyr^{423}, Lys^{424}, Lys^{462}, Glu^{465}, Arg^{466}, Asp^{467}
			Main Protease (MPro)	-4.57	450.62	Phe^{8}, Val^{104}, Ile^{106}, Gln^{110}, Thr^{111}, Asn^{151}, Ile^{152}, Asp^{153}, Ser^{158}, Thr^{292}, Phe^{294}, Asp^{295}, Arg^{298}
			Papain like protease (PLPro)	-5.58	80.7	Cys^{155}, Asn^{156}, Lys^{157}, Thr^{158}, Val^{159}, Glu^{161}, Glu^{167}, Tyr^{171}
			RNA-dependent RNA polymerase (RdRP)	-4.35	649.94	Phe^{441}, Ala^{547}, Ile^{548}, Arg^{836}, Ile^{837}, Ala^{840}, Gly^{841}, Phe^{843}, Val^{844}, Asp^{845}, Arg^{858}, Ser^{861}, Leu^{862}

Sr.	Ligands	PubChem ID	Proteins	Binding energy (kcal/mol)	Dissociation Constant (µM)	Active site residues
**88**	Palmitoleic acid	445638	Helicase	-4.75	330.322	Ala^{4}, Cys^{5}, Val^{6}, Arg^{21}, Pro^{23}, Phe^{24}, Arg^{129}, Leu^{130}, Leu^{132}, Phe^{133}, Glu^{136}, Pro^{234}, Leu^{235}, Ser^{236}
			E-channel proteins	-5.88	48.97	No binding site was found
			Spike Open Protein	-4.21	814.89	Tyr^{707}, Ser^{708}, Asn^{709}, Asn^{710}, Ser^{711}, Ile^{712}, Thr^{1077}, Ala^{1078}, Pro^{1079}, Phe^{1089}, Pro^{1090}, Gly^{1093}, Val^{1094}, Phe^{1095}, Arg^{1107}, Phe^{1121}
			Spike Close Protein	-4.55	462.96	Ile^{105}, Leu^{118}, Val^{120}, Asn^{122}, Val^{127}, Lys^{129}, Phe^{133}, Phe^{135}, Cys^{136}, Pro^{139}, Phe^{140}, Leu^{141}, Leu^{241}
**89**	p-Cymene	7463	Main Protease (MPro)	-5.62	76.07	Met^{6}, Ala^{7}, Phe^{8}, Pro^{9}, Gln^{127}, Phe^{291}, Asp^{295}, Arg^{298}, Gln^{299}, Val^{303}, Thr^{304}
			Papain like protease (PLPro)	-6.67	12.95	Leu^{162}, Gly^{163}, Asp^{164}, Pro^{247}, Pro^{248}, Tyr^{264}, Tyr^{268}, Tyr^{273}, Thr^{301}
			RNA-dependent RNA polymerase (RdRP)	-5.5	93.78	Pro^{323}, Phe^{326}, Gly^{327}, Pro^{328}, Tyr^{346}, His^{347}, Phe^{348}, Arg^{349}, Phe^{396}, Val^{398}, Ser^{664}, Glu^{665}, Met^{666}, Val^{675}, Lys^{676}, $Pro^{677}basn^{118}$
			Helicase	-5.83	53.64	Trp^{114}, Tyr^{120}, Ala^{123}, Asn^{124}, Ala^{134}, Ala^{135}, Leu^{138}, Lys^{139}, Thr^{380}, Asn^{381}, Tyr^{382}, Arg^{409}, Leu^{417}, Tyr^{421}, Phe^{422}, Asn^{423}
			E-channel proteins	-6.27	25.44	No binding site was found
			Spike Open Protein	-5.75	61.5	Ser^{884}, Gly^{885}, Trp^{886}, Thr^{887}, Ile^{896}, Met^{900}, Gln^{901}, Tyr^{904}, Arg^{905}, Met^{1050}
			Spike Close Protein	-5.68	68.4	Tyr^{38}, Asp^{40}, Lys^{41}, Val^{42}, Phe^{43}, Arg^{44}, Glu^{224}, Pro^{225}, Tyr^{279}, Gly^{283}, Thr^{284}
			Main Protease (MPro)	-8.25	0.9	Thr^{25}, His^{41}, Met^{49}, Phe^{140}, Leu^{141}, Asn^{142}, Gly^{143}, Ser^{144}, Cys^{145}, His^{163}, His^{164}, Met^{165}, Glu^{166}, Leu^{167}, Pro^{168}, Arg^{188}, Gln^{189}, Thr^{190}, Gln^{192}
			Papain like protease (PLPro)	-8.86	0.32	Arg^{166}, Ser^{170}, Val^{202}, Glu^{203}, Met^{206}, Tyr^{207}, Met^{208}

Sr.	Ligands	PubChem ID	Proteins	Binding energy (kcal/mol)	Dissociation Constant (µM)	Active site residues
90	Phloridzin dihydrate	9912668	RNA-dependent RNA polymerase (RdRP)	-7.25	4.87	Glu^{474}, Asp^{477}, Lys^{478}, Phe^{480}, Asp^{481}, Tyr^{483}, Asp^{484}, Gly^{485}, Arg^{640}, Lys^{641}, Thr^{643}, Thr^{644}, Thr^{739}, Val^{742}
			Helicase	-7.68	2.363	Lys^{139}, Glu^{142}, Glu^{143}, Lys^{146}, Asn^{179}, Tyr^{180}, Val^{181}, Leu^{227}, Thr^{228}, His^{230}, Cys^{309}, Ser^{310}, Met^{378}, Ala^{379}, Thr^{380}, Tyr^{382}, Asp^{383}, Ala^{407}, Pro^{408}, Arg^{409}, Thr^{410}, Leu^{411}
			E-channel proteins	-7.34	4.2	Arg^{61}, Asn^{64}
			Spike Open Protein	-6.16	30.32	Met^{740}, Tyr^{741}, Ile^{742}, Cys^{743}, Gly^{744}, Asn^{856}, Leu^{966}, Ser^{967}, Ser^{975}, Val^{976}, Leu^{977}, Asn^{978}, Arg^{1000}
			Spike Close Protein	-7.14	5.84	Glu^{96}, Asn^{99}, Ile^{101}, Arg^{102}, Gly^{103}, Trp^{104}, Ile^{119}, Asn^{121}, Val^{126}, Ile^{128}, Arg^{190}, Phe^{192}, Ile^{203}, Ser^{205}, His^{207}, Leu^{226}, Val^{227}
91	Phlorizin	6072	Main Protease (MPro)	-8.75	0.39	Thr^{24}, Thr^{25}, Thr^{26}, Leu^{27}, His^{41}, Cys^{44}, Thr^{45}, Ser^{46}, Met^{49}, Asn^{142}, Gly^{143}, Cys^{145}, His^{164}, Met^{165}, Glu^{166}, Leu^{167}, Pro^{168}, Arg^{188}, Gln^{189}, Thr^{190}, Gln^{192}
			Papain like protease (PLPro)	-9.01	0.25	Arg^{65}, Val^{66}, Phe^{69}, Glu^{70}, Thr^{75}
			RNA-dependent RNA polymerase (RdRP)	-7.91	1.58	Ser^{255}, Tyr^{265}, Ser^{318}, Thr^{319}, Val^{320}, Phe^{321}, Pro^{322}, Pro^{323}, Arg^{349}, Cys^{395}, Phe^{396}, Tyr^{456}, Arg^{457}, Tyr^{458}, Asn^{459}, Leu^{460}, Pro^{461}, Pro^{677}
			Helicase	-8.66	0.447	Asn^{177}, Asn^{179}, Glu^{201}, Lys^{202}, Gly^{203}, Asp^{204}, Pro^{514}, Tyr^{515}, Asn^{516}, Asn^{519}, Ala^{520}, Thr^{530}, Gln^{531}, Thr^{532}, Val^{533}, Asp^{534}, His^{554}, Arg^{560}
			E-channel proteins	-8.66	0.31	No binding site was found
			Spike Open Protein	-7.2	5.27	Ala^{520}, Pro^{521}, Lys^{557}, Leu^{560}, Phe^{562}, Gln^{563}, Gln^{564}, Phe^{565}, Gly^{566}, Arg^{567}, Asp^{568}, Ile^{569}, Asp^{571}
			Spike Close Protein	-7.56	2.89	Phe^{342}, Asn^{343}, Phe^{347}, Leu^{368}, Ser^{371}, Ser^{373}, Phe^{374}, Ser^{375}, Trp^{436}, Asn^{437}, Ser^{438}, Asn^{440}, Leu^{441}, Arg^{509}
			Main Protease (MPro)	-5.91	46.94	Met^{6}, Ala^{7}, Phe^{8}, Gln^{127}, Phe^{291}, Asp^{295}, Arg^{298}, Gln^{299}

Sr.	Ligands	PubChem ID	Proteins	Binding energy (kcal/mol)	Dissociation Constant (µM)	Active site residues
**92**	Pyrogallol	1057	Papain like protease (PLPro)	-5.7	65.9	Thr^{74}, Asp^{76}, Phe^{79}, Arg^{82}, Ala^{153}, Tyr^{154}, Cys^{155}, Asn^{156}, His^{175}
			RNA-dependent RNA polymerase (RdRP)	-5.69	67.59	Lys^{593}, Phe^{594}, Gln^{815}, Pro^{830}, Tyr^{831}, Pro^{832}, Asp^{865}, Pro^{868}, Trp^{916}
			Helicase	-6.06	36.016	Tyr^{120}, Ala^{123}, Asn^{124}, Lys^{131}, Ala^{134}, Ala^{135}, Leu^{138}, Thr^{380}, Asn^{381}, Tyr^{382}, Arg^{409}, Tyr^{421}, Phe^{422}, Asn^{423}
			E-channel proteins	-4.61	418.37	Ser^{16}, Leu^{19}, Phe^{20}, $Phe^{23}eleu^{21}eval^{24}eval^{25}eleu^{28}$
			Spike Open Protein	-4.94	240.51	Ala^{958}, Thr^{961}, Leu^{962}, Gln^{965}, Ser^{1003}, Thr^{1006}, Tyr^{1007}, Gln^{1010}
			Spike Close Protein	-5.75	61.5	Tyr^{38}, Asp^{40}, Lys^{41}, Val^{42}, Phe^{43}, Arg^{44}, Glu^{224}, Pro^{225}, Tyr^{279}, Gly^{283}
**93**	Quercetin	5280343	Main Protease (MPro)	-7.49	3.25	His^{41}, Met^{49}, Phe^{140}, Leu^{141}, Asn^{142}, Gly^{143}, Ser^{144}, Cys^{145}, His^{163}, Met^{165}, Glu^{166}, Leu^{167}, Pro^{168}, His^{172}, Arg^{188}, Gln^{189}, Thr^{190}, Gln^{192}
			Papain like protease (PLPro)	-8.54	0.55	Arg^{166}, Ser^{170}, Val^{202}, Glu^{203}, Met^{206}, Tyr^{207}, Met^{208}, Lys^{232}
			RNA-dependent RNA polymerase (RdRP)	-7.52	3.06	Thr^{591}, Ser^{592}, Lys^{593}, Phe^{594}, Gln^{815}, Pro^{830}, Tyr^{831}, Pro^{832}, Asp^{865}, Pro^{868}, Trp^{916}
			Helicase	-7.56	2.888	Asn^{177}, Arg^{178}, Glu^{201}, Asn^{516}, Ser^{517}, Asn^{519}, Ala^{520}, Thr^{530}, Thr^{532}, Asp^{534}
			E-channel proteins	-7.03	7.07	Leu^{27}, Leu^{28}, Val^{29}, Leu^{31}, Ala^{32}, Thr^{35}, Ala^{36}, Arg^{38}
			Spike Open Protein	-7.14	5.8	Tyr^{38}, Asp^{40}, Lys^{41}, Val^{42}, Phe^{43}, Arg^{44}, Glu^{224}, Pro^{225}, Leu^{226}, Tyr^{279}, Gly^{283}
			Spike Close Protein	-7.43	3.57	Leu^{335}, Cys^{336}, Phe^{338}, Gly^{339}, Phe^{342}, Asn^{343}, Ala^{363}, Asp^{364}, Tyr^{365}, Val^{367}, Leu^{368}, Ser^{371}, Ser^{373}, Phe^{374}
			Main Protease (MPro)	-5.44	102.21	Trp^{218}, Phe^{219}, Leu^{220}, Asn^{221}, Phe^{223}, Ser^{267}, Glu^{270}, Leu^{271}, Asn^{274}, Gly^{275}
			Papain like protease (PLPro)	-6.75	11.31	No binding site was found

Sr.	Ligands	PubChem ID	Proteins	Binding energy (kcal/mol)	Dissociation Constant (µM)	Active site residues
94	Quinic acid	6508	RNA-dependent RNA polymerase (RdRP)	-6.15	30.84	Asp^{452}, Tyr^{456}, Arg^{555}, Thr^{556}, Val^{557}, Ala^{558}, Asp^{623}, Arg^{624}, Lys^{676}, Thr^{680}, Ser^{681}, Ser^{682}
			Helicase	-5.93	44.701	Pro^{406}, Leu^{412}, Thr^{416}, Leu^{417}, Glu^{418}, Pro^{419}, Phe^{422}, Asn^{557}, Asn^{559}, Arg^{560}
			E-channel proteins	-5	216.25	Leu^{51}, Pro^{54}, Tyr^{57}
			Spike Open Protein	-5.16	166.47	Tyr^{396}, Pro^{426}, Asp^{428}, Phe^{429}, Thr^{430}, Phe^{464}, Ser^{514}, Phe^{515}, Glu^{516}, Leu^{517}
			Spike Close Protein	-5.77	58.86	Met^{740}, Tyr^{741}, Ile^{742}, Cys^{743}, Gly^{744}, Asn^{856}, Leu^{966}, Ser^{975}, Val^{976}, Leu^{977}, Asn^{978}, Arg^{1000}
95	Reserpine	5770	Main Protease (MPro)	-7.64	2.5	Thr^{24}, Thr^{25}, Thr^{26}, Leu^{27}, His^{41}, Cys^{44}, Thr^{45}, Ser^{46}, Met^{49}, Phe^{140}, Leu^{141}, Asn^{142}, Gly^{143}, Ser^{144}, Cys^{145}, His^{163}, Met^{165}, Glu^{166}, Gln^{189}
			Papain like protease (PLPro)	-8.54	0.55	Arg^{166}, Ser^{170}, Leu^{185}, Val^{187}, Cys^{192}, Gln^{195}, Thr^{197}, Leu^{199}, Val^{202}, Glu^{203}, Met^{206}, Tyr^{207}, Met^{208}, Gly^{209}, Ile^{222}, Thr^{225}, $Lys^{232}zzn^{500}$
			RNA-dependent RNA polymerase (RdRP)	-8	1.36	Val^{335}, Asp^{336}, Val^{338}, Phe^{340}, Arg^{365}, Leu^{366}, Ser^{367}, Phe^{368}, Leu^{371}
			Helicase	-8.63	0.47	Phe^{145}, Tyr^{149}, Arg^{173}, Pro^{174}, Pro^{175}, Asn^{177}, Asn^{179}, Tyr^{180}, Gln^{404}, Leu^{405}, Pro^{406}, Pro^{408}, Arg^{409}, Thr^{410}, Leu^{412}, Thr^{413}, Gly^{415}, Thr^{416}, Leu^{417}, Phe^{422}, Ser^{485}, His^{554}, Asn^{557}, Asn^{559}, Arg^{560}
			E-channel proteins	-8.93	0.29	No binding site was found
			Spike Open Protein	-7.4	3.75	Ile^{788}, Lys^{790}, Thr^{791}, Pro^{793}, Lys^{795}, Ser^{803}, Leu^{806}, Pro^{807}, Asp^{808}, Pro^{809}, Lys^{814}, Gln^{872}, Ser^{875}

Sr.	Ligands	PubChem ID	Proteins	Binding energy (kcal/mol)	Dissociation Constant (µM)	Active site residues
			Spike Close Protein	-8.13	1.11	Glu^{96}, Asn^{99}, Ile^{101}, Trp^{104}, Ile^{119}, Asn^{121}, Val^{126}, Ile^{128}, Phe^{168}, Tyr^{170}, Ser^{172}, Arg^{190}, Phe^{192}, Phe^{194}, Ile^{203}, Leu^{226}, Val^{227}, Asp^{228}, Leu^{229}, Pro^{230}
96	Resveratrol	445154	Main Protease (MPro)	-7.61	2.65	Met^{6}, Ala^{7}, Phe^{8}, Pro^{9}, Thr^{111}, Gln^{127}, Phe^{291}, Asp^{295}, Arg^{298}, Gln^{299}, Val^{303}, Thr^{304}
			Papain like protease (PLPro)	-8.21	0.95	Phe^{69}, His^{73}, Thr^{74}, Asp^{76}, Phe^{79}, Arg^{82}, Ala^{153}, Tyr^{154}, Asn^{156}, Gln^{174}, His^{175}
			RNA-dependent RNA polymerase (RdRP)	-6.92	8.52	Lys^{411}, Pro^{412}, Gly^{413}, Asn^{414}, Tyr^{546}, Asp^{845}, Asp^{846}, Ile^{847}
			Helicase	-6.88	9.04	Lys^{473}, Phe^{475}, Arg^{579}, Tyr^{582}, Asp^{583}, Leu^{585}, Gln^{586}, Phe^{587}, Thr^{588}, Ser^{589}
			E-channel proteins	-7.6	2.69	No binding site was found
			Spike Open Protein	-6.24	26.62	Arg^{355}, Tyr^{396}, Pro^{426}, Asp^{428}, Phe^{429}, Thr^{430}, Phe^{464}, Ser^{514}, Phe^{515}, Glu^{516}, Leu^{517}, Leu^{518}
			Spike Close Protein	-6.7	12.25	Glu^{96}, Asn^{99}, Ile^{101}, Arg^{102}, Gly^{103}, Trp^{104}, Ile^{119}, Asn^{121}, Val^{126}, Ile^{128}, Arg^{190}, Phe^{192}, Ile^{203}, Val^{227}
97	Rhamnetin	5281691	Main Protease (MPro)	-7.55	2.91	Met^{49}, Phe^{140}, Leu^{141}, Asn^{142}, Gly^{143}, Ser^{144}, Cys^{145}, His^{163}, Met^{165}, Glu^{166}, Leu^{167}, Pro^{168}, His^{172}, Val^{186}, Arg^{188}, Gln^{189}, Thr^{190}, Gln^{192}
			Papain like protease (PLPro)	-8.82	0.35	Cys^{155}, Asn^{156}, Lys^{157}, Glu^{161}, Leu^{162}, Gly^{163}, Glu^{167}, Tyr^{171}, Gln^{269}
			RNA-dependent RNA polymerase (RdRP)	-7.49	3.23	Tyr^{456}, Arg^{553}, Ala^{554}, Arg^{555}, Thr^{556}, Ala^{558}, Tyr^{619}, Pro^{620}, Lys^{621}, Cys^{622}, Asp^{623}, Arg^{624}, Lys^{676}, Thr^{680}, Ser^{681}, Ser^{682}
			Helicase	-7.72	2.201	Asn^{177}, Arg^{178}, Thr^{199}, Phe^{200}, Glu^{201}, Tyr^{515}, Asn^{516}, Ser^{517}, Asn^{519}, Ala^{520}, Thr^{530}, Gln^{531}, Thr^{532}, Asp^{534}
			E-channel proteins	-6.89	8.96	No binding site was found

Sr.	Ligands	PubChem ID	Proteins	Binding energy (kcal/mol)	Dissociation Constant (µM)	Active site residues
			Spike Open Protein	-7.07	6.6	Phe^{329}, Pro^{330}, Asn^{331}, Ile^{332}, Thr^{333}, Asn^{334}, Leu^{335}, Val^{362}, Gly^{526}, Pro^{527}, Lys^{528}, Lys^{529}, Ser^{530}, Gln^{580}
			Spike Close Protein	-7.26	4.81	Met^{740}, Tyr^{741}, Ile^{742}, Cys^{743}, Gly^{744}, Lys^{854}, Asn^{856}, Val^{963}, Leu^{966}, Ser^{967}, Ser^{975}, Val^{976}, Leu^{977}, Asn^{978}, Arg^{1000}
**98**	Rhodiolin	14778358	Main Protease (MPro)	-9.05	0.23	Thr^{25}, His^{41}, Cys^{44}, Thr^{45}, Ser^{46}, Met^{49}, Leu^{141}, Asn^{142}, Gly^{143}, Ser^{144}, Cys^{145}, His^{163}, Met^{165}, Glu^{166}, Leu^{167}, Pro^{168}, Arg^{188}, Gln^{189}, Thr^{190}, Gln^{192}
			Papain like protease (PLPro)	-9.26	0.16	Val^{187}, Gln^{195}, Thr^{197}, Glu^{203}, Met^{206}, Tyr^{207}, Met^{208}, Gly^{209}, Ile^{222}, Pro^{223}, Lys^{232}
			RNA-dependent RNA polymerase (RdRP)	-8.13	1.09	Asp^{452}, Tyr^{456}, Arg^{553}, Ala^{554}, Arg^{555}, Thr^{556}, Asp^{618}, Tyr^{619}, Pro^{620}, Lys^{621}, Cys^{622}, Asp^{623}, Arg^{624}, Ser^{759}, Asp^{760}, Asp^{761}, Cys^{813}, Ser^{814}
			Helicase	-8.53	0.561	Pro^{406}, Arg^{409}, Thr^{410}, Leu^{412}, Thr^{413}, Thr^{416}, Leu^{417}, Pro^{419}, Phe^{422}, Ser^{486}, Ala^{487}, Tyr^{515}, Asn^{516}, His^{554}, Asn^{557}, Asn^{559}, Arg^{560}
			E-channel proteins	-8.58	0.51	Leu^{28}, Val^{29}, Ala^{32}, Ile^{33}, Ala^{36}, Leu^{37}
			Spike Open Protein	-8.68	0.43	Arg^{102}, Gly^{103}, Trp^{104}, Ile^{119}, Asn^{121}, Val^{126}, Ile^{128}, Phe^{168}, Tyr^{170}, Ser^{172}, Arg^{190}, Phe^{192}, Ile^{203}, His^{207}, Leu^{226}, Val^{227}, Asp^{228}, Leu^{229}
			Spike Close Protein	-7.54	2.97	Ser^{46}, Val^{47}, Leu^{48}, Lys^{278}, Thr^{302}, Leu^{303}, Lys^{304}, Ser^{305}, Phe^{306}, Thr^{307}, Ala^{956}, Gln^{957}, Asn^{960}, Thr^{961}, Lys^{964}
			Main Protease (MPro)	-6.46	18.52	Met^{6}, Ala^{7}, Phe^{8}, Pro^{9}, Thr^{111}, Ser^{113}, Gln^{127}, Asp^{295}, Arg^{298}, Gln^{299}
			Papain like protease (PLPro)	-6.36	21.93	No binding site was found
			RNA-dependent RNA polymerase (RdRP)	-5.81	54.92	Gly^{616}, Trp^{617}, Asp^{618}, Asp^{761}, Ala^{762}, Val^{763}, Lys^{798}, Cys^{799}, Trp^{800}, Glu^{811}, Phe^{812}, Cys^{813}, Ser^{814}

Sr.	Ligands	PubChem ID	Proteins	Binding energy (kcal/mol)	Dissociation Constant (µM)	Active site residues
**99**	Salicylic acid	338	Helicase	-5.98	41.432	Val^{496}, Phe^{499}, Leu^{500}, Thr^{501}, Asn^{503}, Trp^{506}, Arg^{507}, Leu^{526}, Tyr^{543}, Ile^{545}, Leu^{573}
			E-channel proteins	-5.55	86.19	Leu^{51}, Pro^{54}, Tyr^{57}
			Spike Open Protein	-5.09	185.15	Met^{740}, Tyr^{741}, Gly^{744}, Asn^{856}, Leu^{966}, Ser^{975}, Val^{976}, Leu^{977}, Asn^{978}, Arg^{1000}
			Spike Close Protein	-6.25	26.14	Tyr^{38}, Asp^{40}, Lys^{41}, Val^{42}, Phe^{43}, Arg^{44}, Glu^{224}, Pro^{225}, Tyr^{279}, Gly^{283}
**100**	Secoisolariciresinol	65373	Main Protease (MPro)	-5.51	91.28	Trp^{218}, Phe^{219}, Leu^{220}, Asn^{221}, Arg^{222}, Phe^{223}, Glu^{270}, Leu^{271}, Asn^{274}, Gly^{275}, Met^{276}, Asn^{277}, Arg^{279}
			Papain like protease (PLPro)	-7.12	6.09	Arg^{183}, Leu^{185}, Leu^{199}, Glu^{203}, Met^{206}, Tyr^{207}, Met^{208}, Gly^{209}, Ile^{222}, Lys^{232}
			RNA-dependent RNA polymerase (RdRP)	-7.04	6.89	Asn^{404}, Val^{405}, Ala^{406}, Phe^{407}, Thr^{409}, Asp^{445}, Gly^{446}, Asn^{447}
			Helicase	-7.04	6.889	Ala^{4}, Val^{6}, Arg^{15}, Arg^{21}, Arg^{22}, Pro^{23}, Phe^{24}, Arg^{129}, Leu^{132}, Phe^{133}, Glu^{136}, Pro^{234}, Leu^{235}, Ser^{236}
			E-channel proteins	-5.93	44.85	Leu^{28}, Val^{29}, Ala^{32}, Ile^{33}
			Spike Open Protein	-5.41	108.43	Met^{740}, Tyr^{741}, Ile^{742}, Cys^{743}, Gly^{744}, Asn^{856}, Val^{963}, Leu^{966}, Ser^{967}, Ser^{975}, Val^{976}, Leu^{977}, Asn^{978}, Arg^{1000}
			Spike Close Protein	-6.15	31.2	Asn^{317}, Arg^{319}, Gly^{548}, Thr^{549}, Gly^{550}, Asp^{568}, Thr^{572}, Thr^{573}, Asp^{574}, Ile^{587}, Pro^{589}, Cys^{590}, Phe^{592}
**101**	Shikimic acid	8742	Main Protease (MPro)	-5.19	157.19	Trp^{218}, Phe^{219}, Leu^{220}, Asn^{221}, Ser^{267}, Glu^{270}, Leu^{271}, Asn^{274}, Gly^{275}, Met^{276}
			Papain like protease (PLPro)	-5.88	48.64	Cys^{155}, Asn^{156}, Lys^{157}, Glu^{161}, Glu^{167}, Tyr^{171}
			RNA-dependent RNA polymerase (RdRP)	-5.03	206.96	Glu^{136}, Cys^{139}, Phe^{157}, Lys^{159}, Lys^{160}, Asp^{161}, Trp^{162}, Tyr^{163}
			Helicase	-5.55	85.322	Leu^{405}, Pro^{406}, Leu^{412}, Thr^{416}, Leu^{417}, Pro^{419}, Phe^{422}, His^{554}, Asn^{557}, Asn^{559}, Arg^{560}
			E-channel proteins	-5.1	184.21	No binding site was found

Sr.	Ligands	PubChem ID	Proteins	Binding energy (kcal/mol)	Dissociation Constant (µM)	Active site residues
			Spike Open Protein	-5.2	154.82	Met^{740}, Tyr^{741}, Ile^{742}, Cys^{743}, Gly^{744}, Phe^{855}, Asn^{856}, Leu^{966}, Leu^{977}, Arg^{1000}
			Spike Close Protein	-5.62	76.07	Met^{740}, Tyr^{741}, Ile^{742}, Cys^{743}, Gly^{744}, Asn^{856}, Leu^{966}, Ser^{967}, Ser^{975}, Val^{976}, Leu^{977}, Asn^{978}, Asp^{979}, Arg^{1000}
**102**	Silyhermin	10389806	Main Protease (MPro)	-8.22	0.94	Thr^{24}, Thr^{25}, Thr^{26}, Leu^{27}, His^{41}, Val^{42}, Cys^{44}, Thr^{45}, Ser^{46}, Met^{49}, Asn^{142}, Gly^{143}, Met^{165}, Glu^{166}, Leu^{167}, Pro^{168}, Arg^{188}, Gln^{189}, Thr^{190}, Gln^{192}
			Papain like protease (PLPro)	-9.44	0.12	Arg^{166}, Val^{187}, Tyr^{207}, Met^{208}, Gly^{209}, Ile^{222}, Pro^{223}, Cys^{224}, Thr^{225}, Ala^{230}, Lys^{232}
			RNA-dependent RNA polymerase (RdRP)	-8.25	0.9	Tyr^{149}, Asn^{150}, Ala^{176}, Asn^{177}, Leu^{178}, Gly^{179}, Glu^{180}, Arg^{181}, Gln^{184}, Thr^{248}, Arg^{249}, Ala^{250}, Thr^{252}, Ala^{253}, Leu^{261}
			Helicase	-8.81	0.351	Pro^{175}, Asn^{177}, Asn^{179}, Tyr^{180}, Pro^{406}, Ala^{407}, Pro^{408}, Arg^{409}, Thr^{410}, Leu^{412}, Thr^{413}, Gly^{415}, Thr^{416}, Leu^{417}, Glu^{418}, Pro^{419}, Phe^{422}, His^{554}, Asn^{557}, Asn^{559}
			E-channel proteins	-8.43	0.66	Phe^{20}, Phe^{23}, Val^{24}, Leu^{27}, Ile^{46}, Leu^{51}, Pro^{54}, Tyr^{57}
			Spike Open Protein	-7.28	4.58	Ser^{730}, Met^{731}, Thr^{732}, Lys^{733}, Gln^{774}, Leu^{858}, Thr^{859}, Val^{860}, Leu^{861}, Pro^{862}, Pro^{863}, Asp^{867}, Ile^{870}, Leu^{959}, Pro^{1057}, His^{1058}
			Spike Close Protein	-8.05	1.25	Arg^{355}, Tyr^{396}, Pro^{426}, Asp^{428}, Phe^{429}, Thr^{430}, Lys^{462}, Pro^{463}, Phe^{464}, Ser^{514}, Phe^{515}, Glu^{516}, Leu^{517}, Leu^{518}
			Main Protease (MPro)	-8.71	0.41	Thr^{24}, Thr^{25}, Thr^{26}, Leu^{27}, His^{41}, Cys^{44}, Thr^{45}, Ser^{46}, Met^{49}, Gly^{143}, Cys^{145}, His^{164}, Met^{165}, Glu^{166}, Leu^{167}, Pro^{168}, Arg^{188}, Gln^{189}, Thr^{190}, Gln^{192}
			Papain like protease (PLPro)	-9.42	0.13	Pro^{59}, Arg^{65}, Val^{66}, Ala^{68}, Phe^{69}, Glu^{70}, Thr^{74}, Thr^{75}, Asp^{76}, Pro^{77}, Phe^{79}, Leu^{80}
			RNA-dependent RNA polymerase (RdRP)	-7.8	1.91	Arg^{305}, Glu^{474}, Asp^{477}, Asp^{481}, Cys^{482}, Tyr^{483}, Asp^{484}, Gly^{485}, Lys^{574}, Arg^{640}, Lys^{641}, Thr^{643}, Val^{737}, Thr^{739}, Val^{742}, Asn^{743}

Sr.	Ligands	PubChem ID	Proteins	Binding energy (kcal/mol)	Dissociation Constant (µM)	Active site residues
**103**	Silymarin	1548994	Helicase	-8.75	0.388	Ala^{4}, Val^{6}, Arg^{15}, Ile^{20}, Arg^{21}, Arg^{22}, Pro^{23}, Phe^{24}, Arg^{129}, Leu^{130}, Leu^{132}, Phe^{133}, Glu^{136}, Thr^{231}, Val^{232}, Pro^{234}, Leu^{235}, Ser^{236}
			E-channel proteins	-8.89	1.1	No binding site was found
			Spike Open Protein	-7.62	2.6	Ala^{27}, Thr^{63}, Trp^{64}, Phe^{65}, His^{66}, Ala^{67}, Pro^{82}, Asp^{138}, Pro^{139}, Phe^{140}, Thr^{240}, Leu^{241}, Leu^{242}, Tyr^{265}
			Spike Close Protein	-7.66	2.44	Tyr^{789}, Lys^{790}, Thr^{791}, Lys^{795}, Ser^{803}, Gln^{804}, Leu^{806}, Pro^{807}, Asp^{808}, Pro^{809}, Lys^{814}, Ser^{875}, Ala^{879}
**104**	Sinapic acid	637775	Main Protease (MPro)	-5.96	43.14	His^{41}, Ser^{46}, Met^{49}, Asn^{142}, Cys^{145}, His^{164}, Met^{165}, Glu^{166}, Pro^{168}, Arg^{188}, Gln^{189}, Thr^{190}, Gln^{192}
			Papain like protease (PLPro)	-7.41	3.7	Phe^{69}, His^{73}, Thr^{74}, Tyr^{154}, Tyr^{171}, Gln^{174}, His^{175}
			RNA-dependent RNA polymerase (RdRP)	-6.03	38.08	Lys^{411}, Pro^{412}, Gly^{413}, Asn^{414}, Phe^{441}, Tyr^{546}, Val^{844}, Asp^{845}, Asp^{846}
			Helicase	-6.01	39.653	Gln^{281}, Asp^{401}, Pro^{402}, Ala^{403}, Pro^{419}, Lys^{430}, Leu^{455}, Tyr^{457}, Asp^{458}, Asn^{459}, Asn^{559}
			E-channel proteins	-6.47	18.12	No binding site was found
			Spike Open Protein	-5.83	53.64	Asn^{99}, Ile^{101}, Arg^{102}, Gly^{103}, Trp^{104}, Ile^{119}, Asn^{121}, Val^{126}, Ile^{128}, Arg^{190}, Phe^{192}, Phe^{194}, Ile^{203}, His^{207}, Val^{227}
			Spike Close Protein	-5.91	46.94	Glu^{96}, Asn^{99}, Ile^{101}, Arg^{102}, Trp^{104}, Ile^{119}, Asn^{121}, Arg^{190}, Phe^{192}, Phe^{194}, Ile^{203}, Ser^{205}, His^{207}, Leu^{226}
			Main Protease (MPro)	-7.92	1.56	Phe^{8}, Lys^{102}, Val^{104}, Arg^{105}, Ile^{106}, Gln^{107}, Gln^{110}, Thr^{111}, Asn^{151}, Asp^{153}, Ser^{158}, Thr^{292}, Phe^{294}, Asp^{295}, Val^{297}, Arg^{298}, Val^{303}
			Papain like protease (PLPro)	-10.3	0.03	Lys^{157}, Glu^{161}, Leu^{162}, Gly^{163}, Asp^{164}, Glu^{167}, Pro^{247}, Pro^{248}, Tyr^{264}, Asn^{267}, Tyr^{268}, Gln^{269}, Tyr^{273}, Thr^{301}

Sr.	Ligands	PubChem ID	Proteins	Binding energy (kcal/mol)	Dissociation Constant (µM)	Active site residues
**105**	Solophenol A	66551634	RNA-dependent RNA polymerase (RdRP)	-6.86	9.38	Tyr^{32}, Ala^{46}, Lys^{47}, Tyr^{129}, His^{133}, Asp^{135}, Asn^{138}, Ala^{706}, Ser^{709}, Thr^{710}, Asp^{711}, Lys^{714}, Gly^{774}, Ser^{778}, Lys^{780}, Asn^{781}, Ser^{784}
			Helicase	-9.4	0.13	Pro^{175}, Leu^{176}, Asn^{177}, Arg^{178}, Thr^{199}, Phe^{200}, Glu^{201}, Lys^{202}, Gly^{203}, Asp^{204}, Val^{210}, Tyr^{211}, Arg^{212}, Val^{484}, Ser^{486}, Asn^{516}, Ser^{517}, Asn^{519}, Ala^{520}, Thr^{530}, Thr^{532}, Asp^{534}
			E-channel proteins	-8.87	0.31	Leu^{28}, Val^{29}, Leu^{31}, Ala^{32}, Thr^{35}
			Spike Open Protein	-6.28	24.8	Trp^{886}, Gly^{889}, Ala^{890}, Tyr^{904}, Ser^{1030}, Glu^{1031}, Leu^{1034}, Gly^{1035}, Gln^{1036}, Ser^{1037}, Lys^{1038}
			Spike Close Protein	-6.93	8.39	Ile^{105}, Leu^{118}, Val^{120}, Asn^{122}, Val^{127}, Lys^{129}, Phe^{133}, Gln^{134}, Phe^{135}, Cys^{136}, Pro^{139}, Phe^{140}, Leu^{141}, Leu^{241}
**106**	Stearic acid	5281	Main Protease (MPro)	-4.22	805.32	Arg^{217}, Trp^{218}, Phe^{219}, Leu^{220}, Asn^{221}, Arg^{222}, Phe^{223}, Glu^{270}, Leu^{271}, Asn^{274}, Gly^{275}
			Papain like protease (PLPro)	-5.69	68.05	Asp^{164}, Met^{208}, Pro^{247}, Pro^{248}, Tyr^{264}, Tyr^{268}, Tyr^{273}
			RNA-dependent RNA polymerase (RdRP)	-4.81	300.53	His^{133}, Asn^{705}, Ala^{706}, Ser^{709}, Thr^{710}, Phe^{766}, Ser^{768}, Ala^{771}, Ser^{772}, Gly^{774}, Val^{776}, Ser^{778}, Ile^{779}, Lys^{780}, Asn^{781}, Thr^{801}
			Helicase	-4.52	485.359	Gly^{3}, Ala^{4}, Cys^{5}, Val^{6}, Gln^{11}, Arg^{21}, Arg^{22}, Pro^{23}, Phe^{24}, Arg^{129}, Leu^{132}, Phe^{133}, Glu^{136}, Pro^{234}, Leu^{235}, Ser^{236}
			E-channel proteins	-5.48	95.54	No binding site was found
			Spike Open Protein	-3.38	3335.49	Arg^{34}, Leu^{189}, Thr^{208}, Pro^{209}, Ile^{210}, Asn^{211}, Leu^{212}, Pro^{217}, Gln^{218}, Gly^{219}, Ser^{221}
			Spike Close Protein	-4.94	237.68	Trp^{104}, Ile^{119}, Asn^{121}, Val^{126}, Ile^{128}, Phe^{168}, Tyr^{170}, Ser^{172}, Arg^{190}, Phe^{192}, Phe^{194}, Ile^{203}, Ser^{205}, Leu^{226}, Val^{227}, Leu^{229}
			Main Protease (MPro)	-4.86	276.21	Met^{6}, Ala^{7}, Phe^{8}, Ser^{113}, Gln^{127}, Phe^{291}, Asp^{295}, Arg^{298}, Gln^{299}
			Papain like protease (PLPro)	-5.1	181.74	No binding site was found
			RNA-dependent RNA polymerase (RdRP)	-5.08	187.66	Arg^{305}, Leu^{470}, Phe^{471}, Glu^{474}, Cys^{730}, Asn^{734}, Arg^{735}, Asp^{736}, Val^{737}, Asp^{738}, Val^{742}

Sr.	Ligands	PubChem ID	Proteins	Binding energy (kcal/mol)	Dissociation Constant (µM)	Active site residues
**107**	Succinic acid	1110	Helicase	-5.22	148.67	Trp^{114}, Tyr^{120}, Ala^{123}, Asn^{124}, Lys^{131}, Ala^{134}, Ala^{135}, Leu^{138}, Thr^{380}, Asn^{381}, Tyr^{382}, Arg^{409}, Leu^{417}, Tyr^{421}, Phe^{422}
			E-channel proteins	-4.05	1074.76	No binding site was found
			Spike Open Protein	-4.36	633.69	Met^{740}, Tyr^{741}, Ile^{742}, Cys^{743}, Gly^{744}, Leu^{966}, Ser^{975}, Val^{976}, Leu^{977}, Asn^{978}, Arg^{1000}
			Spike Close Protein	-4.63	404.48	Cys^{391}, Phe^{392}, Thr^{393}, Glu^{516}, Leu^{517}, Leu^{518}, His^{519}, Ala^{520}, Pro^{521}, Ala^{522}, Asn^{544}, Gly^{545}, Leu^{546}, Phe^{565}
**108**	Syringic acid	10742	Main Protease (MPro)	-5.44	102.9	Phe^{8}, Val^{104}, Ile^{106}, Gln^{110}, Thr^{111}, Asn^{151}, Ile^{152}, Asp^{153}, Ser^{158}, Thr^{292}, Phe^{294}, Asp^{295}, Arg^{298}
			Papain like protease (PLPro)	-6.65	13.44	Thr^{74}, Thr^{75}, Asp^{76}, Phe^{79}, Arg^{82}, Ala^{153}, Tyr^{154}, Asn^{156}, Tyr^{171}, Gln^{174}, His^{175}
			RNA-dependent RNA polymerase (RdRP)	-5.74	62.44	Met^{615}, Gly^{616}, Trp^{617}, Asp^{618}, Asp^{761}, Ala^{762}, Lys^{798}, Cys^{799}, Trp^{800}, His^{810}, Glu^{811}, Phe^{812}, Cys^{813}, Ser^{814}
			Helicase	-5.86	50.734	Ala^{4}, Val^{6}, Arg^{21}, Arg^{22}, Pro^{23}, Phe^{24}, Arg^{129}, Leu^{132}, Phe^{133}, Glu^{136}
			E-channel proteins	-5.19	156.66	Leu^{28}, Leu^{31}, Ala^{32}, Thr^{35}, Cys^{40}
			Spike Open Protein	-5.76	59.66	Tyr^{38}, Asp^{40}, Lys^{41}, Val^{42}, Phe^{43}, Arg^{44}, Glu^{224}, Pro^{225}, Tyr^{279}, Gly^{283}
			Spike Close Protein	-5.43	104.3	Tyr^{38}, Pro^{39}, Asp^{40}, Lys^{41}, Val^{42}, Phe^{43}, Arg^{44}, Glu^{224}, Pro^{225}, Tyr^{279}, Gly^{283}, Thr^{284}
			Main Protease (MPro)	-6.95	8.1	Thr^{25}, Thr^{26}, Leu^{27}, His^{41}, Cys^{44}, Thr^{45}, Ser^{46}, Met^{49}, Asn^{142}, Gly^{143}, Ser^{144}, Cys^{145}, Met^{165}, Glu^{166}, Leu^{167}, Arg^{188}, Gln^{189}, Thr^{190}, Gln^{192}
			Papain like protease (PLPro)	-7.86	1.75	Arg^{166}, Ser^{170}, Val^{202}, Glu^{203}, Met^{206}, Tyr^{207}, Met^{208}, Gly^{209}, Ile^{222}, Lys^{232}

Sr.	Ligands	PubChem ID	Proteins	Binding energy (kcal/mol)	Dissociation Constant (µM)	Active site residues
**109**	Tangeretin	68077	RNA-dependent RNA polymerase (RdRP)	-6.34	22.68	Phe^{35}, Ile^{37}, Tyr^{38}, Asn^{39}, Phe^{48}, Leu^{49}, Lys^{50}, Asp^{208}, Asn^{209}, Asp^{218}, Asp^{221}, Asn^{713}
			Helicase	-7.12	6.049	Ala^{135}, Leu^{138}, Lys^{139}, Glu^{142}, Arg^{178}, Asn^{179}, Cys^{309}, Ser^{310}, Met^{378}, Ala^{379}, Thr^{380}, Asn^{381}, Tyr^{382}, Asp^{383}, Ala^{407}, Pro^{408}, Arg^{409}
			E-channel proteins	-6.89	8.9	Leu^{31}, Ala^{32}, Leu^{34}, Thr^{35}, Ala^{36}, Cys^{40}
			Spike Open Protein	-5.66	71.35	Met^{740}, Ile^{742}, Cys^{743}, Gly^{744}, Asp^{745}, Phe^{855}, Asn^{856}, Val^{963}, Lys^{964}, Leu^{966}, Ser^{967}, Ser^{975}, Val^{976}, Leu^{977}, Asn^{978}, Arg^{1000}
			Spike Close Protein	-6.23	26.94	Ile^{119}, Asn^{121}, Val^{126}, Ile^{128}, Phe^{168}, Tyr^{170}, Ser^{172}, Phe^{192}, Ile^{203}, Ser^{205}, Leu^{226}, Val^{227}, Asp^{228}, Leu^{229}
**110**	Tetracosanoic acid	11197	Main Protease (MPro)	-4.51	498.64	Arg^{131}, Lys^{137}, Thr^{199}, Tyr^{237}, Asn^{238}, Tyr^{239}, Leu^{271}, Leu^{272}, Gly^{275}, Met^{276}, Ala^{285}, Leu^{286}, Leu^{287}, Glu^{288}, Asp^{289}, Glu^{290}
			Papain like protease (PLPro)	-5.49	95.06	Tyr^{207}, Met^{208}, Gly^{209}, Val^{220}, Ile^{222}, Pro^{223}, Lys^{232}, Tyr^{268}
			RNA-dependent RNA polymerase (RdRP)	-4.76	326.99	Lys^{411}, Pro^{412}, Gly^{413}, Asn^{414}, Phe^{415}, Ser^{501}, Asn^{543}, Leu^{544}, Lys^{545}, Tyr^{546}, Val^{557}, Asp^{845}, Asp^{846}, Ile^{847}
			Helicase	-4.54	471.631	Ala^{4}, Cys^{5}, Val^{6}, Arg^{15}, Arg^{21}, Arg^{22}, Pro^{23}, Phe^{24}, Arg^{129}, Leu^{132}, Phe^{133}, Glu^{136}, Pro^{234}, Leu^{235}, Ser^{236}
			E-channel proteins	-5.18	159.32	Leu^{19}, Ala^{22}, Phe^{23}, Phe^{26}
			Spike Open Protein	-4.67	378.71	Tyr^{789}, Lys^{790}, Thr^{791}, Pro^{792}, Ile^{794}, Lys^{795}, Asp^{796}, Phe^{797}, Gly^{798}, Thr^{883}, Ser^{884}, Ala^{893}, Leu^{894}, Gln^{895}, Ile^{896}, Pro^{897}, Phe^{898}, Ala^{899}
			Spike Close Protein	-3.71	1911.03	Trp^{886}, Gly^{889}, Ala^{890}, Tyr^{904}, Ser^{1030}, Glu^{1031}, L???^{034}, Gly^{1035}, Gln^{1036}, Lys^{1038}
			Main Protease (MPro)	-5.22	149.93	Trp^{218}, Phe^{219}, Asn^{221}, Ser^{267}, Glu^{270}, Leu^{271}, Asn^{274}, Gly^{275}, Met^{276}, Asn^{277}, Arg^{279}
			Papain like protease (PLPro)	-5.7	66.57	Leu^{185}, Val^{187}, Thr^{197}, Leu^{199}, Glu^{203}, Tyr^{207}, Met^{208}, Lys^{232}

Sr.	Ligands	PubChem ID	Proteins	Binding energy (kcal/mol)	Dissociation Constant (µM)	Active site residues
**111**	Thymoquinone	10281	RNA-dependent RNA polymerase (RdRP)	-5.18	160.94	His^{613}, Met^{615}, Phe^{766}, Ser^{768}, Ala^{771}, Ser^{772}, Ser^{778}, Ile^{779}, Lys^{780}, Thr^{801}, Thr^{803}
			Helicase	-5.73	63.288	Gln^{281}, Pro^{402}, Ala^{403}, Lys^{430}, Pro^{434}, Phe^{437}, Tyr^{457}, Asp^{458}
			E-channel proteins	-5.71	65.57	Leu^{31}, Ala^{32}, Leu^{34}, Thr^{35}, Cys^{40}
			Spike Open Protein	-4.74	338.22	Glu^{554}, Ser^{555}, Lys^{557}, Lys^{558}, Phe^{559}, Arg^{577}, Leu^{582}, Glu^{583}, Ile^{584}
			Spike Close Protein	-5.59	79.89	Trp^{104}, Ile^{119}, Asn^{121}, Val^{126}, Ile^{128}, Arg^{190}, Phe^{192}, Phe^{194}???Le^{203}, Ser^{205}, Leu^{226}, Val^{227}
**112**	Tyrosine	6057	Main Protease (MPro)	-5.96	42.71	Met^{6}, Ala^{7}, Phe^{8}, Pro^{9}, Gln^{127}, Phe^{291}, Asp^{295}, Arg^{298}, Gln^{299}, Val^{303}, Thr^{304}
			Papain like protease (PLPro)	-7.05	6.85	His^{73}, Thr^{74}, Thr^{75}, Asp^{76}, Phe^{79}, Arg^{82}, Ala^{153}, Tyr^{154}, Asn^{156}, His^{175}
			RNA-dependent RNA polymerase (RdRP)	-5.52	89.91	Glu^{136}, Gly^{137}, Asn^{138}, Cys^{139}, Asp^{140}, Lys^{143}, Phe^{157}, Lys^{159}, Lys^{160}, Asp^{161}, Trp^{162}, Tyr^{163}
			Helicase	-5.78	57.58	Pro^{406}, Ala^{407}, Pro^{408}, Arg^{409}, Leu^{412}, Thr^{416}, Leu^{417}, Pro^{419}, Phe^{422}, Asn^{557}, Asn^{559}, Arg^{560}
			E-channel proteins	-5.52	89.91	Leu^{31}, Thr^{35}, Cys^{40}, Cys^{43}, Cys^{44}, Val^{47}
			Spike Open Protein	-5.97	41.78	Tyr^{38}, Asp^{40}, Lys^{41}, Val^{42}, Phe^{43}, Arg^{44}, Glu^{224}, Pro^{225}, Tyr^{279}, Asn^{282}, Gly^{283}, Thr^{284}
			Spike Close Protein	-5.16	165.35	Asp^{420}, Tyr^{421}, Asn^{422}, Tyr^{423}, Lys^{424}, Arg^{454}, Lys^{462}, Glu^{465}, Arg^{466}, Asp^{467}
			Main Protease (MPro)	-6.06	36.02	Met^{6}, Ala^{7}, Phe^{8}, Pro^{9}, Gln^{127}, Asp^{295}, Arg^{298}, Gln^{299}, Gly^{302}, Val^{303}
			Papain like protease (PLPro)	-5.92	45.92	Leu^{162}, Gly^{163}, Asp^{164}, Pro^{248}, Tyr^{264}, Tyr^{268}, Tyr^{273}, Thr^{301}
			RNA-dependent RNA polymerase (RdRP)	-5.18	159.32	Pro^{323}, Phe^{326}, Gly^{327}, Tyr^{346}, Phe^{348}, Arg^{349}, Asn^{628}, Ser^{664}, Val^{675}, Lys^{676}, Pro^{677}

Sr.	Ligands	PubChem ID	Proteins	Binding energy (kcal/mol)	Dissociation Constant (µM)	Active site residues
**113**	Tyrosol	10393	Helicase	-5.34	122.441	Ala^{135}, Leu^{138}, Lys^{139}, Glu^{142}, Thr^{380}, Asn^{381}, Tyr^{382}, Ala^{407}, Pro^{408}, Arg^{409}
			E-channel proteins	-5.16	163.96	No binding site was found
			Spike Open Protein	-4.48	517.51	Gly^{908}, Ile^{909}, Gln^{1036}, Ser^{1037}, Lys^{1038}, Val^{1040}, Tyr^{1047}, His^{1048}
			Spike Close Protein	-5.21	150.95	Met^{740}, Tyr^{741}, Ile^{742}, Cys^{743}, Gly^{744}, Asn^{856}, Leu^{966}, Ser^{967}, Ser^{975}, Val^{976}, Leu^{977}, Asn^{978}, Arg^{1000}
**114**	Umbelliprenin	1781413	Main Protease (MPro)	-6.84	9.67	Lys^{5}, Arg^{131}, Lys^{137}, Thr^{199}, Tyr^{237}, Tyr^{239}, Leu^{271}, Leu^{272}, Gly^{275}, Met^{276}, Ala^{285}, Leu^{286}, Leu^{287}, Glu^{288}, Asp^{289}, Glu^{290}
			Papain like protease (PLPro)	-7.75	2.1	Arg^{166}, Ser^{170}, Leu^{185}, Val^{187}, Gln^{195}, Thr^{197}, Leu^{199}, Val^{202}, Glu^{203}, Met^{206}, Tyr^{207}, Met^{208}, Thr^{225}, Lys^{232}
			RNA-dependent RNA polymerase (RdRP)	-6.74	11.49	Thr^{76}, Phe^{77}, Tyr^{80}, Glu^{84}, Tyr^{87}, Asn^{88}, Lys^{91}, His^{99}, Asp^{100}, Phe^{101}, Phe^{102}, Pro^{112}
			Helicase	-6.35	22.263	Gly^{3}, Ala^{4}, Val^{6}, Arg^{21}, Arg^{22}, Pro^{23}, Phe^{24}, Arg^{129}, Leu^{132}, Phe^{133}, Glu^{136}, Pro^{234}, Leu^{235}, Ser^{236}
			E-channel proteins	-8.9	0.3	No binding site was found
			Spike Open Protein	-6.5	17.34	Ser^{884}, Gly^{885}, Trp^{886}, Thr^{887}, Ala^{890}, Leu^{894}, Ile^{896}, Gln^{901}, Tyr^{904}, Arg^{905}, Leu^{1034}, Gly^{1035}, Gln^{1036}
			Spike Close Protein	-8.12	1.11	Trp^{104}, Ile^{119}, Asn^{121}, Val^{126}, Ile^{128}, Phe^{168}, Tyr^{170}, Ser^{172}, Arg^{190}, Phe^{192}, Phe^{194}, Ile^{203}, Ser^{205}, Lys^{206}, His^{207}, Leu^{226}, Val^{227}, Leu^{229}
			Main Protease (MPro)	-5.16	165.07	Met^{6}, Ala^{7}, Phe^{8}, Pro^{9}, Gln^{127}, Ile^{152}, Asp^{153}, Tyr^{154}, Phe^{291}, Asp^{295}, Arg^{298}, Gln^{299}, Val^{303}, Thr^{304}
			Papain like protease (PLPro)	-5.4	110.28	No binding site was found
			RNA-dependent RNA polymerase (RdRP)	-4.88	265.24	Lys^{411}, Pro^{412}, Gly^{413}, Asn^{414}, Tyr^{546}, Asp^{845}, Asp^{846}, Ile^{847}cser^{15}

Sr.	Ligands	PubChem ID	Proteins	Binding energy (kcal/mol)	Dissociation Constant (µM)	Active site residues
**115**	Undecanoic acid	8180	Helicase	-4.68	371.12	Pro^{406}, Ala^{407}, Pro^{408}, Arg^{409}, Leu^{412}, Gly^{415}, Thr^{416}, Leu^{417}, Phe^{422}, His^{554}, Asn^{557}, Asn^{559}
			E-channel proteins	-5.1	182.97	Leu^{51}, Pro^{54}, Tyr^{57}, Ser^{60}, Arg^{61}
			Spike Open Protein	-5.09	187.35	Glu^{96}, Asn^{99}, Ile^{101}, Arg^{102}, Gly^{103}, Trp^{104}, Ile^{119}, Asn^{121}, Val^{126}, Ile^{128}, Arg^{190}, Phe^{192}, Phe^{194}, Ile^{203}, Ser^{205}, Leu^{226}, Val^{227}
			Spike Close Protein	-4.79	309.8	Glu^{96}, Ile^{101}, Arg^{102}, Trp^{104}, Ile^{119}, Asn^{121}, Val^{126}, Ile^{128}, Arg^{190}, Phe^{192}, Phe^{194}, Ile^{203}, Leu^{226}, Val^{227}
**116**	Vanillic acid	8468	Main Protease (MPro)	-5.35	120.39	Trp^{218}, Phe^{219}, Leu^{220}, Asn^{221}, Phe^{223}, Glu^{270}, Leu^{271}, Asn^{274}, Gly^{275}, Met^{276}
			Papain like protease (PLPro)	-6.6	14.65	No binding site was found
			RNA-dependent RNA polymerase (RdRP)	-5.59	80.56	Tyr^{456}, Arg^{555}, Thr^{556}, Val^{557}, Ala^{558}, Asp^{623}, Arg^{624}, Lys^{676}, Thr^{680}, Ser^{681}, Ser^{682}, Gly^{683}
			Helicase	-5.63	74.546	Ala^{4}, Val^{6}, Arg^{21}, Arg^{22}, Pro^{23}, Phe^{24}, Arg^{129}, Leu^{132}, Phe^{133}, Glu^{136}
			E-channel proteins	-5.55	85.18	Leu^{28}, Leu^{31}, Ala^{32}, Leu^{34}, Thr^{35}, Cys^{40}
			Spike Open Protein	-5.67	70.15	Met^{740}, Tyr^{741}, Ile^{742}, Cys^{743}, Gly^{744}, Ser^{746}, Leu^{966}, Ser^{967}, Ser^{975}, Val^{976}, Leu^{977}, Asn^{978}, Arg^{1000}
