# Supplementary material for: Effectiveness of Natural Antioxidants against SARS-CoV-2? Insights from the In-Silico World
Source: Antibiotics (Basel). 2021 Aug 20;10(8):1011. doi: 10.3390/antibiotics10081011 (PMC8388999; doi:10.3390/antibiotics10081011)
Supplement: Supplementary file 1 [file antibiotics-10-01011-s001.zip › Suppl Material/Table S2 a.pdf]

Table S2a **Glycyrrhizin docking to nonspecific human blood proteins**

PDB ID	Target	Classification	Energy kcal/m	Dissoc. constant [nM	Contacting receptor residues
3I7H	Ddb1 (damage specific DNA binding protein 1)	Protein binding	-11.36	4.6860894	Asn^{16}, Gly^{17}, Cys^{18}, Val^{19}, Thr^{20}, Glu^{65}, Leu^{66}, Thr^{118}, Ile^{121}, Ile^{123}, Ile^{124}, Asp^{125}, Pro^{126}, Asp^{166}, Lys^{168}, Phe^{169}, Tyr^{171}, Ser^{217}, Met^{218}, Ala^{221}, Val^{259}, Cys^{260}, His^{261}, Asn^{262}, Arg^{263}, Glu^{312}, Cys^{313}, Leu^{314}
4U7D	Human RECQ-like helicase	Hydrolase	-10.78	12.5782793	Phe^{87}, Lys^{88}, Leu^{89}, Arg^{93}, Thr^{115}, Gly^{116}, Gly^{118}, Lys^{119}, Ser^{120}, Leu^{121}, Tyr^{123}, Gln^{124}, Leu^{143}, Asp^{146}, Gln^{147}, Val^{150}, Leu^{154}, Asp^{219}, Glu^{220}, Leu^{253}, Gly^{377}, Ile^{378}, Asp^{379}, Pro^{381}, Arg^{407}
4FAD	PI3KA	Transferase	-10.66	15.4021875	Glu^{384}, Asn^{386}, Gln^{388}, Gln^{391}, Val^{393}, Gln^{396}, Asn^{430}, Gln^{432}, Tyr^{434}, Leu^{460}, Asn^{465}, Phe^{497}, Lys^{501}, Leu^{502}, Leu^{564}, Pro^{566}, Asn^{634}, Met^{1036}, Ser^{1044}, Lys^{1045}, Ile^{1048}
1W6K	Human OSC	Isomerase	-10.54	18.9238535	Arg^{8}, Arg^{9}, Arg^{10}, Gly^{11}, Pro^{13}, Tyr^{14}, Lys^{15}, Tyr^{54}, Leu^{373}, Ser^{644}, Ala^{645}, Gln^{646}, Gln^{648}, Leu^{682}, Asp^{686}, Trp^{687}, Pro^{688}, Gln^{689}, Glu^{690}, Asn^{691}, Arg^{708}
4NY0	Human focal adhesion kinase	Transferase	-10.52	19.3436641	Tyr^{42}, Arg^{86}, Ser^{88}, Val^{95}, Lys^{121}, Glu^{123}, Arg^{125}, Arg^{127}, Asp^{154}, Leu^{157}, Glu^{158}, Tyr^{251}, Arg^{252}, Phe^{253}, Lys^{255}, Glu^{256}, Cys^{257}, Phe^{258}, Ile^{336}, Asn^{339}, Leu^{343}
5FFG	Integrin alpha V Beta 6 head	Cell adhesion	-10.3	28.0414766	Asp^{24}, Phe^{25}, Phe^{26}, Val^{27}, Met^{34}, Ser^{100}, Lys^{101}, Gln^{102}, Asp^{162}, Phe^{163}, Thr^{164}, Lys^{165}, Val^{228}, Gln^{352}, Lys^{410}, Gly^{411}, Ala^{412}, Thr^{413}, Asp^{414}, Lys^{417}, Gly^{419}, Pro^{421}
2QFZ	Human tbc1 domain family member 22a	Hydrolase activator	-9.87	58.0400859	Arg^{216}, Leu^{235}, Ser^{236}, Gly^{237}, Lys^{369}, Asp^{372}, Gly^{373}, Ile^{374}, Gln^{375}, Asp^{376}, Lys^{388}, Tyr^{442}, Gln^{443}, Glu^{445}, Pro^{446}, Asp^{447}, Gly^{448}, Phe^{449}, Ser^{450}
3EHT	The extracellular domain of human corticotropin releasing factor receptor type 1 (crfr1)	Membrane protein	-9.82	63.3642891	Gln^{277}, Asp^{254}, Ala^{253}, Arg^{251}, Tyr^{250}, Asn^{249}, Gly^{248}, Tyr^{178}, Gly^{175}, Lys^{174}, Tyr^{173}, Ile^{171}, Ile^{20}, Met^{19}, Pro^{18}, Asn^{17}, Ile^{16}, Pro^{15}, Gln^{14}
6FFI	MGLUR5 in complex with MMPEP	Membrane protein	-9.82	63.9012891	Tyr^{572}, Leu^{635}, Gln^{647}, Ile^{651}, Glu^{888}, Val^{889}, Tyr^{890}, Leu^{891}, Ile^{892}, Cys^{893}, Thr^{895}, Thr^{896}, Asn^{897}, Val^{900}, Tyr^{952}, Asn^{956}, Tyr^{957}, Lys^{958}, Ile^{959}, Met^{962}
4WB7	Camp-dependent protein kinase a (catalytic alpha subunit)	Transferase	-9.72	74.6356797	Lys^{2}, Asp^{3}, Gln^{6}, Thr^{7}, Gln^{26}, Tyr^{30}, Pro^{38}, Gly^{39}, Glu^{42}, Lys^{43}, Glu^{46}, Ile^{47}, Asp^{322}, Asn^{326}, Phe^{336}, Asn^{341}, Asn^{344}, Asp^{345}, Asn^{348}, His^{349}, Lys^{350}
5T40	Human EXOG (hexog)	Hydrolase	-9.66	83.2898594	Arg^{109}, Lys^{110}, Cys^{112}, Lys^{113}, Phe^{114}, Arg^{138}, His^{140}, Pro^{143}, Ala^{144}, Gly^{145}, Phe^{158}, Asn^{171}, Ser^{172}, Trp^{175}, Asn^{176}, Glu^{179}, Phe^{307}, Leu^{311}, Arg^{314}
2XMS	Human NDRG2 protein	Signaling protein	-9.66	83.4305547	Lys^{162}, His^{170}, Thr^{173}, Ser^{177}, Glu^{181}, Met^{182}, Leu^{184}, Gly^{185}, His^{186}, Phe^{188}, Ser^{189}, Gln^{190}, Glu^{191}, Leu^{193}, Arg^{204}, Gln^{250}, Ala^{251}, Pro^{252}
3VJ9	The human squalene synthase	Transferase	-9.62	88.8068594	Leu^{76}, Arg^{77}, Asp^{80}, Asp^{84}, Met^{86}, Glu^{116}, Lys^{117}, Tyr^{171}, Val^{175}, Leu^{211}, Gln^{212}, Asn^{215}, Ile^{216}, Asp^{219}, Glu^{222}, Asp^{223}, Gly^{226}, Arg^{228}, Phe^{230}
2J0S	Exon junction	Hydrolase	-9.62	89.2576641	Lys^{25}, Val^{26}, Glu^{27}, Phe^{28}, Gln^{81}, Leu^{221}, Pro^{222}, His^{223}, Glu^{224}, Leu^{226}, Leu^{240}, Val^{241}, Lys^{242}, Arg^{243}, Asn^{357}, Arg^{358}, Glu^{359}, Tyr^{395}
2G6P	Truncated (delta 1-89) human methionine aminopeptidase type 1	Hydrolase	-9.6	92.4780625	Ser^{125}, Glu^{128}, Gln^{129}, Leu^{131}, Lys^{132}, Gly^{133}, Thr^{134}, Tyr^{195}, Tyr^{196}, His^{212}, Ser^{299}, Tyr^{300}, Cys^{301}, His^{310}, Thr^{311}, Ala^{312}, Asn^{314}, Thr^{348}, Gly^{352}, Trp^{353}
3MWE	Truncated human ATP-citrate lyase	Transferase	-9.53	103.3754453	Lys^{4}, Ala^{223}, Thr^{224}, Asp^{226}, Lys^{230}, Trp^{233}, Gly^{234}, Asp^{235}, Ile^{236}, Glu^{237}, Phe^{238}, Pro^{240}, Glu^{250}, Ala^{251}, Ile^{253}, Ala^{254}, Asp^{255}, Asp^{257}, Ala^{258}, Leu^{264}, Lys^{265}, Leu^{266}, Thr^{267}
3OE9	the chemokine CXCR4 receptor	Signaling protein, hydrolase	-9.5	108.5612031	Asp^{1010}, Glu^{1011}, Gly^{1012}, Arg^{1014}, Tyr^{1018}, Lys^{1019}, Tyr^{1024}, Thr^{1026}, Gly^{1028}, Ile^{1029}, Gly^{1030}, His^{1031}, Leu^{1032}, Asp^{1070}, Ala^{1073}, Ala^{1074}, Val^{1103}, Phe^{1104}, Gln^{1105}, Met^{1106}, Gly^{1107}, Glu^{1108}, Trp^{1138}, Thr^{1142}, Arg^{1145}
5FN3	Gamma secretase in class 1 of the apo-state ensemble	Hydrolase	-9.5	108.9282891	Asn^{55}, Val^{138}, Gln^{139}, Cys^{140}, Pro^{141}, Asn^{142}, Asp^{143}, Gly^{144}, Phe^{145}, His^{158}, Cys^{159}, Arg^{160}, Gln^{163}, Tyr^{173}, Asp^{336}, Thr^{643}, Trp^{648}
4UV8	LSD1(KDM1A)-CoRest	Transcription	-9.46	116.9303047	Gln^{228}, Thr^{230}, Phe^{231}, His^{250}, His^{253}, Ser^{254}, Glu^{257}, Arg^{258}, Tyr^{267}, Arg^{269}, Lys^{271}, Pro^{274}, Thr^{278}, Ser^{299}, Phe^{300}, Gly^{301}, Ala^{826}, Leu^{830}, Gly^{831}, Met^{833}
1ZXM	Human topo IIA ATPASE/AMP-PNP	Isomerase	-9.43	121.7641172	Gln^{60}, Met^{61}, Trp^{62}, Tyr^{72}, Arg^{241}, Phe^{308}, Gln^{309}, Gln^{310}, Ile^{311}, Ser^{320}, Lys^{321}, Gly^{322}, Gly^{323}, Arg^{324}, Asp^{327}, His^{354}, Lys^{357}, Asn^{380}
5A2E	Extracellular SRCR domains of human CD6	Immune system	-9.43	121.9698047	Asn^{49}, Gly^{50}, Ser^{51}, Thr^{57}, Leu^{97}, Ala^{98}, Pro^{99}, Pro^{100}, Arg^{150}, Arg^{152}, Ala^{160}, Leu^{161}, Arg^{162}, Arg^{200}, Gln^{201}, Leu^{202}, Gly^{203}, Tyr^{236}
5IOH	Repoman-PP1A (protein phosphatase 1, alpha isoform) holoenzyme	Hydrolase/protein binding	-9.42	124.8862031	His^{66}, Arg^{96}, Asn^{124}, His^{125}, Trp^{206}, Pro^{209}, Asp^{210}, Lys^{211}, Asp^{212}, Asp^{220}, Arg^{221}, Thr^{226}, His^{248}, Gln^{249}, Val^{250}, Glu^{256}, Phe^{258}, Tyr^{272}, Phe^{276}
4JKJ	The S18Y variant of ubiquitin carboxy-terminal hydrolase l1	Hydrolase	-9.32	146.8531875	Met^{1}, Gln^{2}, Leu^{55}, Thr^{56}, Ala^{57}, Glu^{60}, Thr^{85}, Ile^{86}, Gly^{87}, Asn^{88}, Cys^{90}, Asp^{155}, Lys^{157}, Val^{158}, Asn^{159}, Phe^{160}, His^{161}, Arg^{178}
6F39	C1R homodimer CUB1-EGF-CUB2	Hydrolase	-9.27	160.3245	Gly^{36}, Tyr^{37}, Arg^{38}, Gln^{91}, Gly^{92}, Asn^{93}, Asp^{125}, Leu^{126}, Asp^{127}, Glu^{128}, Ser^{133}, Asp^{139}, Pro^{140}, Gln^{141}, Pro^{142}, Gly^{154}, Tyr^{155}, Phe^{156}, Glu^{167}, Asp^{168}, Thr^{169}, His^{170}
1OE9	Myosin V motor	ATPASE/myosin	-9.16	192.0577969	Asn^{111}, Tyr^{113}, Ser^{165}, Gly^{166}, Ala^{167}, Gly^{168}, Lys^{169}, Thr^{170}, Val^{171}, Lys^{174}, Leu^{197}, Ala^{198}, Asn^{200}, Pro^{201}, Glu^{204}, Asn^{208}, Asn^{214}, Asn^{216}, Asn^{307}, Gln^{310}, Ala^{311}, Leu^{314}
2FM5	PDE4D2	Hydrolase	-9.16	192.3822344	Ile^{79}, Pro^{80}, Lys^{85}, Thr^{86}, Gln^{88}, Glu^{89}, Leu^{92}, Ala^{93}, Leu^{96}, Glu^{97}, Val^{99}, Leu^{112}, Ser^{113}, Arg^{116}, Thr^{119}, Val^{120}, His^{123}, Thr^{124}, Gln^{127}, Glu^{128}, Ile^{143}, Met^{147}
5WG5	Human GRK2	Transferase	-9.12	206.166625	Lys^{30}, Lys^{31}, Ile^{32}, Leu^{33}, Val^{180}, Asn^{183}, Ile^{184}, His^{185}, Leu^{186}, Asn^{189}, Asp^{190}, Lys^{210}, Ala^{211}, Asp^{212}, Glu^{616}, Arg^{617}, Lys^{618}, Gln^{633}, Cys^{634}, Asp^{635}, Ser^{636}, Asp^{637}, Leu^{640}
3HRZ	Human factor B	Immune system	-9.11	210.3848594	Thr^{326}, Lys^{327}, Tyr^{343}, Asp^{348}, Gly^{349}, Ser^{350}, Pro^{351}, Arg^{497}, Trp^{515}, Asp^{578}, Glu^{581}, Lys^{582}, Ser^{583}, Asp^{584}, Gly^{586}, Cys^{587}, Thr^{588}, Ala^{589}, Lys^{621}, Cys^{622}, Pro^{623}, Gln^{624}
4MWS	Human PPCA (trigonal crystal form 1)	Hydrolase	-9.08	219.4520781	Gly^{57}, Ser^{150}, Tyr^{151}, Tyr^{183}, Asp^{187}, Leu^{190}, Val^{191}, Phe^{220}, Tyr^{221}, Asp^{222}, Leu^{232}, Gln^{233}, Val^{235}, Ala^{236}, Gly^{240}, Ile^{246}, Tyr^{247}, Pro^{301}, Pro^{302}, Arg^{344}, Ala^{374}, Cys^{375}, His^{429}
5Z90	BRD4 bromodomain 1	Transcription	-9.05	230.8500625	Pro^{45}, Pro^{46}, Pro^{47}, Pro^{48}, Glu^{49}, Lys^{55}, Tyr^{98}, Ile^{101}, Lys^{102}, Thr^{103}, Pro^{104}, Met^{105}, Arg^{113}, Tyr^{118}, Tyr^{119}, Gln^{127}, Asp^{128}, Thr^{131}
3PUF	Human RNASE H2 complex	Hydrolase	-9.02	243.25025	Asp^{34}, Glu^{35}, Gly^{37}, Arg^{38}, Gly^{39}, Pro^{40}, Asp^{67}, Ser^{68}, Lys^{69}, Leu^{71}, Glu^{73}, Arg^{76}, Asp^{141}, Val^{143}, Asp^{169}, Ser^{179}, Lys^{183}, Arg^{186}, Asp^{187}, Gly^{207}, Ser^{208}, Tyr^{210}, Lys^{215}
4URW	RAS:SOS complex	Signaling protein	-9.01	250.7537813	Leu^{23}, Ile^{24}, Gln^{25}, Asn^{26}, His^{27}, Val^{29}, Glu^{31}, Tyr^{32}, Asp^{33}, Ile^{36}, Asp^{38}, Ser^{39}, Arg^{41}, Lys^{42}, Gln^{43}, Val^{44}, Val^{45}, Glu^{153}, Tyr^{157}
3EHI	Human thymidyalte synthase M190K	Transferase	-8.95	274.681875	Lys^{93}, Gly^{94}, Ser^{95}, Thr^{96}, Asn^{97}, Glu^{100}, Phe^{137}, Arg^{140}, His^{141}, Met^{149}, Glu^{150}, Ser^{151}, Asp^{152}, Tyr^{153}, Ser^{154}, Gln^{162}, Lys^{287}, Asp^{289}
5C5X	The S156E mutant of human aquaporin 5	Transport protein	-8.95	275.145875	Ala^{57}, Gln^{58}, Gly^{61}, Pro^{62}, Gly^{65}, Gly^{66}, Leu^{76}, Gln^{81}, Ile^{82}, Arg^{86}, Phe^{147}, Ser^{149}, Thr^{150}, Asp^{151}, Ser^{152}, Arg^{153}, Pro^{161}, Ala^{162}, Ile^{165}, Tyr^{243}, Pro^{245}

PDB ID	Target	Classification	Energy kcal/m	Dissoc. constant [nM	Contacting receptor residues
1IKN	IKAPPABALPHA/NF-KAPPAB complex	Transcription factor	-8.94	279.82925	Arg^{50}, Thr^{52}, Lys^{221}, Glu^{222}, Asp^{223}, Ile^{224}, Glu^{225}, Tyr^{227}, Glu^{234}, Ala^{235}, Arg^{236}, Gly^{237}, Ser^{238}, Phe^{239}, Ser^{240}, Gln^{241}, Arg^{273}, Pro^{275}
3UVU	Flap endonuclease 1 (FEN1)	Protein binding/peptide	-8.93	284.5923438	Leu^{104}, Ser^{105}, Arg^{106}, Glu^{107}, Pro^{110}, Ile^{112}, Asn^{146}, Ala^{148}, Ser^{149}, Gly^{150}, Thr^{151}, Ser^{152}, Thr^{155}, Trp^{184}, Asn^{188}, Gly^{191}, Asp^{192}, Trp^{231}, Asn^{235}, Arg^{238}, Tyr^{277}
4JXJ	ribosomal RNA small subunit methyltransferase a from rickettsia bellii	Transferase	-8.92	290.9057813	Ser^{38}, Gly^{39}, Leu^{40}, Glu^{41}, Ser^{44}, Asn^{45}, Lys^{110}, Thr^{112}, Ser^{133}, Ser^{134}, Val^{136}, Ala^{137}, Ser^{138}, Lys^{196}, Thr^{198}, Pro^{199}, Leu^{200}, Glu^{201}, Ile^{203}
5GNT	BDLP-like folding of Mitofusin 1	Hydrolase	-8.91	293.8667188	Val^{70}, Arg^{73}, Arg^{74}, His^{75}, Leu^{197}, Asp^{198}, Ala^{199}, Asp^{200}, Leu^{227}, Ser^{228}, Lys^{229}, Pro^{230}, Asn^{231}, Ser^{331}, Thr^{335}, Lys^{336}, Gln^{339}
4ZMV	Human P-cadherin (SS-X-dimer pocket I)	Cell adhesion	-8.87	316.5212188	Met^{0}, Asp^{1}, Trp^{2}, Val^{3}, Ile^{4}, Lys^{25}, Ser^{26}, Asn^{27}, Lys^{28}, Phe^{77}, Val^{88}, Glu^{89}, Asp^{90}, Pro^{91}, Met^{92}, Asn^{93}
4E+49	Carbonic anhydrase (CA)	Lyase	-8.85	323.5429688	His^{4}, Trp^{5}, Gly^{6}, Tyr^{7}, Gly^{8}, His^{10}, Asn^{11}, His^{15}, Lys^{18}, Asp^{19}, Gly^{63}, His^{64}, Lys^{170}, Phe^{231}, Asn^{232}, Glu^{236}, Pro^{237}, Glu^{238}, Glu^{239}
5HKJ	Single chain recombinant globular head of the complement system protein C1Q	Signaling protein	-8.81	346.7251563	Tyr^{49}, Ser^{68}, Ser^{70}, Arg^{75}, Arg^{76}, Ser^{77}, Leu^{98}, Gln^{99}, Leu^{100}, Asp^{104}, Val^{168}, Leu^{169}, Thr^{170}, Asn^{171}, Pro^{172}, Gly^{174}
5K10	Isocitrate dehydrogenase (IDH1)	Oxidoreductase	-8.8	355.6153438	Ile^{112}, Ile^{113}, Cys^{114}, Lys^{115}, Val^{125}, Lys^{126}, Pro^{127}, Leu^{201}, Ser^{202}, Lys^{203}, Gly^{204}, Trp^{205}, Gln^{242}, Lys^{243}, Ile^{244}, Arg^{338}, Asn^{357}, Glu^{360}, Glu^{361}, Ile^{364}, Glu^{368}
3GR4	Human pyruvate kinase M2	Transferase	-8.78	367.2042188	Phe^{26}, Leu^{308}, Lys^{311}, Met^{312}, Gly^{315}, Asn^{318}, Arg^{319}, Leu^{353}, Asp^{354}, Ile^{389}, Tyr^{390}, Gln^{393}, Leu^{394}, Glu^{397}, Leu^{398}, Leu^{401}, Arg^{445}
4NYH	Pir1 dual specificity phosphatase core	Hydrolase	-8.77	374.7173438	His^{30}, Ile^{31}, Pro^{32}, Glu^{33}, Arg^{34}, Lys^{36}, Lys^{64}, Tyr^{95}, Thr^{96}, Gln^{97}, Tyr^{99}, Gly^{118}, His^{119}, Thr^{153}, His^{154}, Arg^{158}
2Q7N	LIF receptor (domains 1-5)	CYTOKINE receptor/CYTOKINE	-8.76	377.893	Met^{15}, Arg^{16}, Trp^{18}, Asp^{40}, Arg^{41}, Ala^{57}, Leu^{58}, Ser^{59}, Pro^{60}, Glu^{81}, Gly^{113}, Ser^{114}, Ala^{115}, Leu^{116}, Pro^{117}, His^{118}, Ser^{147}, Gly^{148}, His^{180}, Phe^{181}
2QX4	Quinone reductase ii	Oxidoreductase	-8.74	394.8454063	Tyr^{67}, Tyr^{104}, Trp^{105}, Phe^{106}, Ser^{107}, Val^{108}, Ala^{110}, Lys^{113}, Met^{116}, Asp^{117}, Leu^{120}, Cys^{121}, Phe^{126}, Pro^{170}, Leu^{171}, Gly^{174}, Thr^{175}, Phe^{178}
4W9O	The FK1 domain of FKBP51	Isomerase	-8.71	413.9533125	Tyr^{57}, Phe^{67}, Asp^{68}, Phe^{77}, Gly^{84}, Gln^{85}, Val^{86}, Ile^{87}, Lys^{88}, Trp^{90}, Tyr^{111}, Ala^{112}, Tyr^{113}, Ile^{122}, Leu^{128}, Phe^{130}
4ZWJ	Rhodopsin bound to arrestin	Signaling protein	-8.7	418.8730625	Thr^{242}, Lys^{311}, Gln^{312}, Asn^{315}, Thr^{2063}, Glu^{2071}, Asp^{2072}, Val^{2075}, Phe^{2080}, Arg^{2081}, Asp^{2083}, Glu^{2149}, Lys^{2151}, Glu^{2162}, Asp^{2163}, Lys^{2164}, Ile^{2165}, Lys^{2167}
4IP9	Human serum amyloid A1	Protein binding	-8.69	423.8512813	Ala^{14}, Met^{17}, Trp^{18}, Tyr^{21}, Met^{24}, Asp^{33}, His^{37}, Ile^{58}, Arg^{62}, Ile^{65}, Gln^{66}, Phe^{69}, His^{71}, Asp^{79}
2UW9	PKB-Beta (AKT2)	Transferase	-8.69	428.1654063	Trp^{334}, Glu^{366}, Ile^{367}, Phe^{369}, Lys^{378}, Ser^{379}, Ala^{382}, Gly^{383}, Leu^{385}, Lys^{386}, Lys^{387}, Lys^{390}, Gln^{391}, Arg^{392}, Leu^{393}, Gly^{394}, Gly^{395}, Gly^{396}, Glu^{402}, His^{406}
4OJ2	Aquaporin	Transport protein	-8.68	433.2540625	Phe^{9}, Ala^{12}, Val^{56}, Gln^{57}, Gly^{60}, His^{61}, Ile^{62}, Ser^{63}, Gly^{64}, His^{80}, Val^{81}, Arg^{85}, Tyr^{89}, Ser^{148}, Thr^{149}, Glu^{151}, Pro^{160}, Ala^{161}
4GWN	Human mature MEPRIN Beta	Hydrolase	-8.67	442.1185625	Val^{374}, Glu^{375}, Ile^{377}, Lys^{378}, Glu^{379}, Ile^{380}, Pro^{381}, Gln^{386}, Leu^{387}, Tyr^{388}, His^{389}, Val^{390}, Thr^{391}, Cys^{427}, Pro^{428}, His^{429}, His^{430}, Ile^{431}, His^{433}, Phe^{453}
3I69	Apo Glutathione transferase A1-1 GIMF-helix	Transferase	-8.57	519.00875	Lys^{4}, Leu^{23}, Ala^{24}, Gly^{27}, Val^{28}, Glu^{29}, Phe^{30}, Glu^{31}, Glu^{32}, Phe^{34}, Thr^{193}, Lys^{196}, Phe^{197}, Gln^{199}, Pro^{200}, Gly^{201}, Ser^{202}, Pro^{203}
4GWG	6-phosphogluconate dehydrogenase apo-form	Oxidoreductase	-8.56	528.734625	Met^{194}, Ile^{197}, Cys^{198}, Tyr^{201}, Ile^{234}, Thr^{235}, Asn^{237}, Ile^{238}, Leu^{239}, Phe^{241}, Leu^{249}, Leu^{250}, Ile^{253}, Arg^{254}, Asp^{255}, Ser^{256}, Ala^{257}, Arg^{287}
3R4O	Heat shock protein 90	Chaperone	-8.56	530.5225	Arg^{46}, Glu^{47}, Ser^{50}, Asn^{51}, Ser^{52}, Asp^{54}, Ala^{55}, Lys^{58}, Ile^{96}, Met^{98}, Asn^{106}, Leu^{107}, Lys^{112}, Gly^{132}, Gln^{133}, Gly^{135}, Thr^{184}
5AIU	RNF4-ring domain, UBC13-UB (isopeptide crosslink)	Ligase/signaling protein	-8.51	579.189	Tyr^{143}, Val^{147}, Arg^{151}, Leu^{152}, Ile^{153}, Ile^{192}, Tyr^{193}, Ile^{194}, Gly^{195}, Ser^{196}, Val^{199}, Ser^{200}, Pro^{202}, His^{225}, Val^{226}, Phe^{227}, Thr^{244}
4KTV	MAT enzymes: MATA2B	Transferase	-8.46	630.1889375	Asp^{51}, Ala^{52}, Lys^{53}, Gln^{135}, Asp^{286}, Tyr^{287}, Thr^{288}, Val^{290}, Tyr^{320}, Ala^{321}, Ile^{322}, Gly^{323}, Val^{324}, Ser^{325}, Arg^{356}, Pro^{357}, Gly^{358}, Val^{361}, Arg^{362}, Lys^{367}
5HEX	Human hexokinase 2	Transferase	-8.43	658.4594375	Asp^{84}, Arg^{91}, Ser^{155}, Phe^{156}, Asp^{209}, Ile^{229}, Gly^{231}, Thr^{232}, Gly^{233}, Ser^{234}, Asn^{235}, Asp^{413}, Gly^{414}, Ser^{415}, Lys^{418}, Lys^{419}, Glu^{446}, Asp^{447}, Gly^{448}, Ser^{449}
3OHM	Activated G alpha Q	Signaling protein	-8.42	673.0668125	Ile^{91}, Met^{94}, Asp^{95}, Lys^{98}, Ile^{99}, Pro^{100}, Tyr^{101}, Lys^{102}, Tyr^{103}, Glu^{104}, Lys^{107}, Ala^{110}, Gln^{111}, Arg^{114}
6DK3	Human mitochondrial serine hydroxymethyltransferase 2	Transferase	-8.42	678.770875	Gln^{133}, Trp^{134}, Arg^{238}, Cys^{241}, Asp^{242}, Lys^{245}, Ala^{246}, His^{247}, Leu^{248}, Phe^{268}, Lys^{269}, Asp^{272}, Arg^{293}, Val^{296}, Lys^{297}, Ala^{298}, Tyr^{309}
4IA0	The PDE5A1 catalytic domain	Hydrolase	-8.41	684.5233125	Gln^{552}, Thr^{557}, Asp^{558}, Phe^{559}, Ser^{560}, Lys^{630}, Ala^{631}, Thr^{769}, Lys^{770}, Pro^{771}, Phe^{840}, Pro^{841}, Leu^{842}, Asp^{844}, Gly^{845}, Lys^{848}
5HZJ	Intersectin1 containing wildtype LOV2 domain	Signaling protein	-8.38	717.64975	Glu^{1275}, Lys^{1276}, Ala^{1279}, Val^{1283}, Asn^{1284}, Lys^{1286}, Glu^{1287}, Arg^{1474}, Gln^{1580}, Ala^{1581}, His^{1582}, Val^{1583}, Gln^{1584}, Cys^{1585}, Glu^{1586}, Gln^{1591}, Val^{1593}, Asn^{1653}, Gln^{1655}
2CZK	Human myo-inositol mono-phosphatase 2 (impa2)	Hydrolase	-8.34	775.5878125	Thr^{55}, Asp^{58}, His^{59}, Glu^{62}, Glu^{81}, Asp^{101}, Ile^{103}, Asp^{104}, Gly^{105}, Thr^{106}, Cys^{107}, Glu^{173}, Gly^{175}, Pro^{176}, Ser^{207}, Gln^{224}, Gly^{226}, Leu^{227}, His^{228}, Trp^{230}, Asp^{231}
4IRG	The ETS transcription factor ERG	DNA binding protein	-8.32	795.47425	Gly^{292}, Gln^{293}, Ile^{294}, Gln^{295}, Trp^{334}, Arg^{337}, Lys^{338}, Ser^{339}, Lys^{340}, Pro^{341}, Met^{343}, Lys^{347}, Leu^{348}, Arg^{350}, Ala^{351}, Tyr^{354}
1HF0	DNA-binding domain of OCT-1	Transcription	-8.29	841.0376875	Arg^{102}, Lys^{103}, Lys^{104}, Arg^{105}, Thr^{106}, Arg^{113}, Glu^{117}, Val^{144}, Val^{147}, Trp^{148}, Asn^{151}, Arg^{152}, Gln^{154}, Lys^{155}, Arg^{158}
2V5Y	The receptor protein tyrosine phosphatase mu ectodomain	Hydrolase	-8.23	921.2934375	Gln^{402}, Val^{403}, Gly^{404}, Ser^{430}, Pro^{431}, Tyr^{432}, Thr^{433}, Asn^{434}, Glu^{485}, Pro^{486}, Thr^{487}, Gln^{488}, Thr^{489}, Tyr^{490}, Gly^{491}, Val^{492}, Ile^{493}
3R6I	AKR1C3	Oxidoreductase	-8.21	967.5095625	Gln^{6}, Cys^{7}, His^{14}, Phe^{15}, Pro^{17}, Leu^{19}, Gly^{45}, Phe^{46}, Arg^{47}, Glu^{77}, Asp^{78}, Ile^{79}, Phe^{80}, Tyr^{110}, Asp^{112}, Lys^{161}, Val^{281}, Phe^{284}
5POQ	BRD1 in complex with N10974A	Gene regulation	-8.18	1010.912375	Phe^{99}, Asp^{100}, Ile^{103}, Arg^{124}, Asp^{125}, Gly^{127}, Gly^{128}, Leu^{131}, Arg^{132}, Arg^{135}, Arg^{136}, Asp^{139}
2CF9	Recombinant human thrombin	Hydrolase/hydrolase inhibitor	-8.11	1130.035625	Cys^{122}, Leu^{123}, Asp^{125}, Glu^{127}, Thr^{128}, Ser^{129}b, Ser^{203}, Phe^{204}a, Asn^{204}b, Arg^{206}, Tyr^{208}, Lys^{235}
3DOF	Complex of ARL2 and BART	Signaling protein/hydrolase	-8.05	1254.69675	Lys^{9}, Gln^{12}, Lys^{13}, Glu^{14}, Phe^{81}, Glu^{82}, Ser^{83}, Thr^{84}, Asp^{85}, Glu^{113}, Arg^{114}, Leu^{115}, Ala^{116}, Gly^{117}, Ala^{118}, Arg^{179}, Ile^{180}, Ala^{183}, Asp^{184}, His^{187}
5U7Z	Human acid ceramidase (ASAH1, aCDase)	Hydrolase	-8.05	1267.467375	Tyr^{59}, Val^{77}, Lys^{81}, Gly^{91}, Met^{94}, Gln^{95}, Asp^{98}, Glu^{99}, Lys^{117}, Ala^{120}, Ala^{121}, Asp^{124}, Ile^{125}, Pro^{126}, Leu^{127}, Glu^{129}
3BYI	Human rho GTPASE activating protein 15 (arhgap15)	Signaling protein	-8.03	1302.161875	Ile^{379}, Lys^{380}, Lys^{381}, Gln^{382}, Asp^{383}, Asn^{384}, Arg^{387}, Thr^{427}, Gln^{428}, Gln^{457}, Glu^{460}, Leu^{461}, Ser^{464}, Glu^{465}

PDB ID	Target	Classification	Energy kcal/m	Dissoc. constant [nM	Contacting receptor residues
5D1M	UBCH5B	Ligase	-7.96	1475.392875	Phe^{62}, Lys^{63}, Pro^{64}, Pro^{65}, Lys^{66}, Val^{67}, Ala^{68}, Ser^{83}, Ile^{84}, Leu^{86}, Leu^{89}, Arg^{90}, Ser^{91}, Gln^{92}, Trp^{93}
5L6D	The human METTL3-METTL14 complex	Transferase	-7.95	1500.506125	Asp^{395}, Pro^{396}, Pro^{397}, Trp^{398}, Ile^{400}, Pro^{405}, Tyr^{406}, Thr^{408}, Thr^{433}, Lys^{459}, Ile^{467}, Leu^{476}, Asn^{477}, His^{478}, Glu^{481}, Val^{507}, Thr^{510}, Ser^{511}, Lys^{513}, Phe^{534}
6PAX	Human PAX-6 paired domain-DNA complex	Gene regulation	-7.94	1513.222625	Asn^{6}, Gln^{7}, Leu^{8}, Phe^{12}, Val^{13}, Asn^{14}, Gly^{15}, Arg^{16}, Pro^{17}, Leu^{18}, Arg^{23}, Ser^{46}, Cys^{49}, Lys^{52}, Ile^{53}, Arg^{56}, Pro^{65}, Arg^{66}, Ile^{68}
2J8B	Human CD59	Lipid binding protein	-7.93	1536.3845	Phe^{42}, Cys^{45}, Asn^{46}, Phe^{47}, Leu^{59}, Thr^{60}, Tyr^{61}, Tyr^{62}, Cys^{63}, Gln^{74}, Leu^{75}, Glu^{76}, Asn^{77}
5W45	APOBEC3H	DNA binding protein	-7.91	1594.505875	Arg^{10}, Leu^{11}, Asn^{14}, Lys^{16}, Arg^{17}, Arg^{18}, Leu^{19}, Arg^{20}, Arg^{110}, Phe^{157}, Asn^{158}, Lys^{161}, Met^{162}, Glu^{165}
3WO2	Human interleukin-18	Immune system	-7.9	1613.45625	Asn^{14}, Leu^{15}, Asn^{16}, Cys^{127}, Lys^{129}, Glu^{130}, Arg^{131}, Phe^{134}, Lys^{139}, Asp^{142}, Glu^{143}, Asp^{146}, Arg^{147}, Ser^{148}
1CWF	Human cyclophilin A	Isomerase/immunosuppressant	-7.83	1812.73725	Leu^{39}, Thr^{41}, Gly^{42}, Gly^{45}, Phe^{46}, Gly^{47}, Tyr^{48}, Lys^{49}, Gly^{50}, Phe^{67}, Thr^{68}, Arg^{69}, His^{70}, Lys^{76}, Cys^{161}
5F6D	UBC9 (K48A/K49A/E54A)	Ligase	-7.75	2078.302	Lys^{65}, Ser^{71}, Pro^{72}, Lys^{74}, Tyr^{87}, Pro^{88}, Ser^{89}, Thr^{91}, Val^{92}, Cys^{93}, Leu^{94}, Ser^{95}, Leu^{97}, Glu^{98}, Glu^{99}, Lys^{101}, Ala^{129}
4A7U	Human I113T SOD1	Oxidoreductase	-7.75	2103.00225	Val^{5}, Cys^{6}, Val^{7}, Lys^{9}, Gly^{51}, Asp^{52}, Asn^{53}, Thr^{54}, Cys^{146}, Gly^{147}, Val^{148}, Ile^{149}, Gly^{150}
4MXV	Lymphotoxin alpha	Cytokine/immune system	-7.71	2219.709	His^{32}, Arg^{51}, Phe^{53}, Phe^{74}, Tyr^{76}, Gln^{78}, Tyr^{134}, His^{135}, Gly^{136}, Phe^{165}, Phe^{169}
5M3D	Tuning of CD81LEL (space group p31)	Cell adhesion	-7.71	2219.709	Asp^{117}, Ala^{120}, Lys^{121}, Lys^{124}, Gln^{125}, Asp^{128}, Leu^{131}, Gln^{132}, Ser^{159}, Ser^{160}, Thr^{163}, Asp^{189}, His^{191}, Gln^{192}, Asp^{195}
2IYB	Complex between the 3rd LIM domain of TES and the evh1 domain of MENA	Metal-binding	-7.71	2246.08975	Glu^{3}, His^{40}, Asn^{43}, Asn^{44}, Thr^{45}, Phe^{46}, Arg^{47}, Ala^{63}, Ile^{64}, Pro^{65}, Lys^{66}, Val^{110}, Leu^{111}, Ser^{113}
5OKF	Human 14-3-3 sigma with the hspb6 phosphopeptide	Signaling protein	-7.68	2354.78575	Glu^{17}, Tyr^{19}, Glu^{20}, Lys^{49}, Asn^{50}, Val^{51}, Gly^{53}, Gly^{54}, Gln^{55}, Arg^{56}, Ala^{57}, Arg^{237}, Arg^{238}, Ala^{239}, Sep^{240}, Pro^{242}
3ZR0	Human MTH1	Hydrolase	-7.62	2614.55625	Asp^{109}, Gln^{110}, Ile^{111}, Pro^{112}, Phe^{113}, Lys^{114}, Asp^{115}, Met^{116}, Pro^{118}, Ser^{121}, Tyr^{122}
1QE6	Interleukin-8	Immune system	-7.55	2902.9835	Leu^{25}, Val^{27}, Glu^{29}, Ser^{30}, Asn^{36}, Thr^{37}, Ile^{39}, Pro^{53}, Lys^{54}, Gln^{59}, Val^{62}, Glu^{63}, Leu^{66}
1Z3U	Angiopoietin-2 receptor binding domain	Signaling protein	-7.51	3116.24075	Thr^{364}, Asn^{365}, Arg^{368}, His^{389}, Tyr^{391}, Leu^{392}, Ser^{393}, Ser^{394}, Leu^{397}, Arg^{400}, His^{402}, Lys^{404}
4X1L	Mutation-induced destabilization of profilin 1 in ALS	Protein binding	-7.48	3289.1775	Tyr^{25}, Lys^{26}, Asp^{27}, Gly^{49}, Val^{52}, Gly^{53}, Asp^{81}, Phe^{84}, Lys^{105}, Thr^{106}, Asp^{107}, Lys^{108}
5H0V	H88A mutated human transthyretin	Transport protein	-7.36	4061.753	Pro^{11}, Thr^{60}, Glu^{61}, Glu^{62}, Phe^{64}, Val^{65}, Asp^{99}, Gly^{101}, Pro^{102}, Arg^{103}, Arg^{104}, Tyr^{105}
5UZP	Citrate synthase	Transferase	-7.34	4194.1185	Arg^{52}, Gly^{56}, Lys^{57}, Thr^{58}, Val^{59}, Val^{60}, Gly^{61}, Gln^{62}, Ile^{63}, Lys^{76}, Gly^{77}, Leu^{78}, Val^{79}, Phe^{444}, Pro^{445}
1JT3	Human acidic fibroblast growth factor	Hormone/growth factor	-7.32	4294.4035	Asn^{18}, Lys^{112}, Lys^{113}, Asn^{114}, Lys^{118}, Pro^{121}, Arg^{122}, His^{124}, Gln^{127}, Lys^{128}, Ala^{129}
4Y5O	CCM2 HHD in complex with MEKK3 NPB1	Transferase	-7.28	4633.277	Tyr^{325}, Gly^{328}, Ala^{329}, Ser^{330}, Ile^{331}, Pro^{358}, Glu^{359}, Lys^{360}, Asp^{361}, His^{364}
5VZ3	Growth factor	Signaling protein	-7.19	5348.0335	Asp^{5}, Pro^{11}, Gly^{12}, Arg^{16}, Leu^{17}, Arg^{53}, Ala^{54}, Lys^{107}, Asp^{108}, Cys^{109}, His^{110}
3H91	Human chromobox homolog 2 (CBX2)	Transcription	-7.19	5375.1815	Glu^{9}, Gln^{10}, Val^{11}, Phe^{12}, Arg^{23}, Trp^{33}, Asn^{41}, Ser^{42}, Trp^{43}, Glu^{44}, Pro^{45}, Glu^{47}, Asn^{48}, Leu^{50}
5N7E	DBL-homology domain of BCR-ABL	Signaling protein	-7.18	5448.2525	Lys^{8}, Leu^{9}, Glu^{10}, Val^{11}, Asp^{68}, Ile^{89}, Ile^{91}, Asn^{92}, Tyr^{93}, Arg^{94}
5FUG	A human YL1-H2A.Z-H2B complex	DNA binding protein	-7.13	5948.0375	Ile^{32}, Leu^{36}, Arg^{39}, Thr^{40}, Thr^{41}, Gly^{44}, Arg^{45}, Val^{46}, Ala^{50}, Tyr^{53}, Ser^{54}, Ile^{57}, Leu^{58}
2X1X	Structure of VEGF-C in complex with domains 2 and 3 of VEGFR2 in a tetragonal crystal form	Hormone/signaling protein	-7.06	6660.171	Arg^{161}, Cys^{162}, Gly^{163}, Gly^{164}, Cys^{165}, Cys^{166}, Glu^{169}, Leu^{171}, Gln^{172}, Cys^{173}, Met^{174}, Asn^{175}, His^{206}
3KLT	A vimentin fragment	Structural protein	-6.94	8169.2	Asp^{297}, Glu^{300}, Ala^{301}, Arg^{304}, Asn^{305}, Asp^{307}, Ala^{308}, Gln^{311}
1BL1	PTH receptor N-terminus fragment	Hormone receptor	-6.84	9671.203	Glu^{2}, Ala^{3}, Phe^{6}, Leu^{7}, Asn^{9}, Glu^{10}, Arg^{12}, Glu^{13}, Val^{16}, Ile^{23}, Val^{26}
