# Supplementary material for: Effectiveness of Natural Antioxidants against SARS-CoV-2? Insights from the In-Silico World
Source: Antibiotics (Basel). 2021 Aug 20;10(8):1011. doi: 10.3390/antibiotics10081011 (PMC8388999; doi:10.3390/antibiotics10081011)
Supplement: Supplementary file 1 [file antibiotics-10-01011-s001.zip › Suppl Material/Table S2 b.pdf]

Table S2b **Baicalin docking to nonspecific human blood proteins**

PDB ID	Targets	Classification	Energy (Kcal/mol)	Dissoc. constant [nM]	Contacting receptor residues
3I7H	Ddb1 (damage specific DNA binding	Protein binding	-9.89	56.2083555	Lys^{408}, Gly^{409}, Leu^{410}, Trp^{411}, Ser^{428}, Thr^{457}, Phe^{458}, Phe^{459}, Ser^{502}, Cys^{503}, Asn^{504}, Cys^{540}, Leu^{541}, Asp^{542}, Ile^{543}, Thr^{544}, Arg^{589}, Ser^{590}, Ile^{591}, Leu^{592}, Leu^{638}, Arg^{639}, Thr^{640}, Tyr^{678}
4WB7	Camp-dependent protein kinase a (c	Transferase	-9.67	82.3116016	Leu^{104}, Gly^{105}, Thr^{106}, Gly^{107}, Phe^{109}, Val^{112}, Ala^{125}, Lys^{127}, Met^{175}, Tyr^{177}, Val^{178}, Glu^{182}, Lys^{223}, Glu^{225}, Asn^{226}, Leu^{228}, Thr^{238}, Asp^{239}, Phe^{382}, Tyr^{385}
4NY0	Human focal adhesion kinase	Transferase	-9.06	228.1386875	Val^{95}, Trp^{97}, Arg^{125}, Ile^{126}, Arg^{127}, Leu^{129}, Phe^{147}, Glu^{158}, Tyr^{251}, Arg^{252}, Phe^{253}, Asp^{254}, Lys^{255}, Glu^{256}, Cys^{257}, Phe^{258}, Ile^{336}, Asn^{339}, Met^{340}, Leu^{343}, Tyr^{347}
3ZR0	Human MTH1	Hydrolase	-8.96	272.8336563	Arg^{5}, Tyr^{7}, Thr^{8}, Leu^{9}, Lys^{23}, Phe^{27}, Asn^{33}, Gly^{36}, Gly^{37}, Lys^{38}, Glu^{56}, Phe^{72}, Met^{81}, Val^{83}, Glu^{100}, Met^{101}
4IA0	The PDE5A1 catalytic domain	Hydrolase	-8.94	281.24975	Tyr^{612}, His^{613}, His^{617}, Val^{660}, Met^{681}, Glu^{682}, Thr^{723}, Asp^{724}, Leu^{725}, Ala^{726}, Asp^{764}, Leu^{765}, Ile^{768}, Val^{782}, Gln^{817}, Phe^{820}
1OE9	Myosin V motor	ATPASE/myosin	-8.9	300.385875	Ala^{11}, Arg^{12}, Arg^{63}, Asn^{64}, Pro^{65}, Asp^{66}, Ile^{67}, Ala^{757}, Glu^{760}, Lys^{761}, Arg^{763}, Ala^{764}, Leu^{767}, Arg^{768}, Cys^{771}
4GWN	Human mature MEPRIN Beta	Hydrolase	-8.88	309.651875	Gly^{123}, Cys^{124}, Trp^{125}, Ser^{126}, Ala^{148}, Thr^{149}, His^{152}, Glu^{153}, His^{156}, His^{162}, His^{186}, Asn^{187}, Val^{208}, Met^{209}, His^{210}, Tyr^{211}, Phe^{216}, Gly^{236}, Arg^{238}
4U7D	Human RECQ-like helicase	Hydrolase	-8.82	342.0749375	Lys^{293}, Pro^{294}, Ser^{295}, Thr^{297}, Gly^{419}, Phe^{420}, Gly^{421}, Phe^{424}, Arg^{425}, Ser^{428}, Thr^{511}, Pro^{512}, Leu^{513}, Ile^{516}, Glu^{540}, Glu^{543}, Thr^{566}, Ile^{567}, Ser^{568}
2CZK	Human myo-inositol mono-phospha	Hydrolase	-8.66	450.4035938	Asp^{101}, Ile^{103}, Asp^{104}, Gly^{105}, Thr^{106}, Cys^{107}, Asn^{108}, Glu^{173}, Ile^{174}, Gly^{175}, Pro^{176}, Lys^{177}, Arg^{178}, Leu^{183}, Gly^{205}, Leu^{227}, His^{228}, Trp^{230}, Asp^{231}
4FAD	PI3KA and MTOR	Transferase	-8.64	462.7325	Leu^{942}, Gly^{943}, Ile^{944}, Gly^{945}, Asp^{946}, Ile^{968}, Leu^{969}, Tyr^{972}, Val^{983}, Pro^{984}, Phe^{985}, Phe^{1031}, Met^{1034}, Leu^{1035}, Met^{1039}, Leu^{1042}, Glu^{1046}, Asp^{1047}, Tyr^{1050}
4MWS	Human PPCA (trigonal crystal form 1	Hydrolase	-8.62	482.6755	Gln^{363}, Ile^{364}, Glu^{392}, Glu^{415}, Phe^{416}, Ser^{417}, His^{418}, Ile^{419}, Ala^{420}, Arg^{445}, Phe^{446}, Lys^{449}, Gln^{450}, Pro^{451}, Tyr^{452}
5K10	Isocitrate dehydrogenase (IDH1)	Oxidoreductase	-8.61	488.412	Lys^{72}, Cys^{73}, Ala^{74}, Thr^{75}, Ile^{76}, Thr^{77}, Arg^{82}, Ser^{94}, Pro^{95}, Asn^{96}, Arg^{109}, Glu^{306}, Ala^{307}, Ala^{308}, His^{309}, Gly^{310}, Thr^{311}, Val^{312}, Thr^{313}, Arg^{314}
6FFI	MGLUR5 in complex with MMPEP	Membrane protein	-8.52	573.3531875	Leu^{673}, Ala^{674}, Ser^{676}, Lys^{677}, Ile^{680}, Asn^{745}, Asp^{749}, Val^{752}, Leu^{756}, Tyr^{765}, Ala^{770}, Arg^{773}, Ala^{774}, Lys^{920}, Asn^{923}, Val^{924}, Pro^{925}
2XMS	Human NDRG2 protein	Signaling protein	-8.51	580.167375	Tyr^{41}, Asn^{60}, Tyr^{61}, Lys^{62}, Phe^{65}, Gln^{66}, Phe^{69}, Gln^{70}, Arg^{83}, His^{85}, Asp^{87}, Met^{91}, Glu^{92}, Glu^{93}
5HZJ	Intersectin1 containing wildtype LO	Signaling protein	-8.43	660.6859375	Glu^{1287}, Pro^{1471}, Leu^{1588}, Ser^{1589}, Glu^{1590}, Gln^{1591}, Leu^{1592}, Val^{1593}, Ser^{1596}, Val^{1597}, Thr^{1598}, Asn^{1599}, Arg^{1604}, Thr^{1661}, Pro^{1662}, Phe^{1664}
2G6P	Truncated (delta 1-89) human methi	Hydrolase	-8.41	690.3245625	Glu^{128}, Gln^{129}, Leu^{131}, Lys^{132}, Gly^{133}, Tyr^{195}, Tyr^{196}, His^{212}, Tyr^{300}, Cys^{301}, His^{303}, Leu^{308}, His^{310}, Thr^{311}, Ala^{312}, Asn^{314}, Trp^{353}
1W6K	Human OSC	Isomerase	-8.37	729.8651875	Gly^{11}, Pro^{13}, Tyr^{14}, Tyr^{54}, Ala^{55}, Gly^{57}, Asp^{97}, Trp^{370}, Leu^{373}, Leu^{682}, Pro^{688}, Gln^{689}, Thr^{705}, Arg^{708}
3EHI	Human thymidyalte synthase M190I	Transferase	-8.37	731.0980625	Ile^{92}, Lys^{93}, Gly^{94}, Ser^{95}, Thr^{96}, Asn^{97}, Glu^{100}, Phe^{137}, Arg^{140}, His^{141}, Met^{149}, Glu^{150}, Ser^{151}, Asp^{152}, Tyr^{153}, Ser^{154}, Asp^{289}, Lys^{292}
3GR4	Human pyruvate kinase M2	Transferase	-8.36	741.0366875	Phe^{26}, Gly^{315}, Asn^{318}, Arg^{319}, Leu^{353}, Asp^{354}, Gly^{355}, Ile^{389}, Tyr^{390}, Gln^{393}, Leu^{394}, Glu^{397}, Leu^{398}, Leu^{401}, Arg^{445}
5IOH	Repoman-PP1A (protein phosphatas	Hydrolase/protein bi	-8.33	778.210375	His^{66}, Arg^{96}, His^{125}, Ile^{130}, Tyr^{134}, Trp^{206}, Arg^{221}, Val^{223}, His^{248}, Gln^{249}, Val^{250}, Tyr^{272}, Phe^{276}
2QX4	Quinone reductase ii	Oxidoreductase	-8.27	866.9810625	Ala^{10}, His^{11}, Gln^{12}, Ser^{16}, Phe^{17}, Asn^{18}, Tyr^{42}, Pro^{102}, Leu^{103}, Tyr^{104}, Pro^{109}, Ile^{111}, Thr^{147}, Thr^{148}, Gly^{149}, Ala^{191}, Pro^{192}, Glu^{193}
3EHT	The extracellular domain of human c	Membrane protein	-8.23	927.534375	His-^{285}, Asp-^{254}, Ala-^{253}, Arg-^{251}, Tyr-^{250}, Asn-^{249}, Tyr-^{178}, Lys-^{174}, Tyr-^{173}, Ile-^{20}, Met-^{19}, Pro-^{18}, Asn-^{17}, Ile-^{16}
5HEX	Human hexokinase 2	Transferase	-8.19	993.9931875	Ser^{155}, Phe^{156}, Pro^{157}, Asn^{208}, Asp^{209}, Thr^{210}, Thr^{213}, Ile^{229}, Thr^{232}, Gly^{233}, Ser^{234}, Asn^{235}, Glu^{260}, Gln^{291}, Glu^{294}, Asp^{413}, Gly^{448}, Ser^{449}
4JKJ	The S18Y variant of ubiquitin carbox	Hydrolase	-8.14	1081.518375	Gly^{72}, Gln^{73}, Val^{75}, Pro^{77}, Val^{79}, Tyr^{80}, Phe^{81}, Glu^{122}, Lys^{123}, Met^{124}, Ser^{125}, Pro^{126}, Arg^{129}, Phe^{181}, Pro^{182}
5T40	Human EXOG (hexog)	Hydrolase	-8.14	1085.175375	Arg^{109}, Ser^{137}, Arg^{138}, His^{140}, Pro^{143}, Ala^{144}, Gly^{145}, Gln^{166}, Phe^{168}, Asn^{171}, Ser^{172}, Trp^{175}, Asn^{176}, Glu^{179}, Leu^{311}, Arg^{314}
5L6D	The human METTL3-METTL14 compl	Transferase	-8.12	1120.539375	Asp^{395}, Pro^{396}, Pro^{397}, Trp^{398}, Ile^{400}, Pro^{405}, Tyr^{406}, Thr^{408}, Trp^{431}, Thr^{433}, Trp^{457}, Lys^{459}, Glu^{481}, Val^{507}, Arg^{508}, Ser^{509}, Thr^{510}, Ser^{511}, His^{512}, Lys^{513}
2QFZ	Human tbc1 domain family member	Hydrolase activator	-8.1	1151.211875	Phe^{329}, Lys^{369}, Leu^{370}, Asp^{372}, Gly^{373}, Ile^{374}, Gln^{375}, Trp^{439}, Tyr^{442}, Gln^{443}, Glu^{445}, Pro^{446}, Asp^{447}, Gly^{448}, Phe^{449}, Ser^{450}
2J0S	Exon junction	Hydrolase	-8.08	1186.72325	Thr^{30}, Ser^{62}, Ala^{63}, Ile^{64}, Arg^{67}, Ser^{82}, Gln^{83}, Thr^{86}, Ile^{239}, Leu^{240}, Val^{241}, Glu^{245}, Leu^{248}, Glu^{249}, Gly^{250}, Gly^{372}
2V5Y	The receptor protein tyrosine phosp	Hydrolase	-8.04	1280.368125	Gln^{402}, Val^{403}, Gly^{404}, Ser^{430}, Tyr^{432}, Thr^{433}, Asn^{434}, Glu^{485}, Pro^{486}, Thr^{487}, Gln^{488}, Thr^{489}, Tyr^{490}, Gly^{491}, Val^{492}
3R6I	AKR1C3	Oxidoreductase	-8.01	1335.549875	Gln^{6}, Cys^{7}, His^{14}, Phe^{15}, Met^{16}, Pro^{17}, Leu^{19}, Gly^{45}, Phe^{46}, Arg^{47}, His^{48}, Glu^{77}, Asp^{78}, Ile^{79}, Phe^{80}, Asp^{112}, Val^{281}, Phe^{284}
3MWE	Truncated human ATP-citrate lyase	Transferase	-8	1367.484	Val^{56}, Lys^{58}, Gly^{67}, Val^{72}, Val^{74}, Asn^{75}, Phe^{110}, Pro^{112}, His^{113}, Gln^{115}, Glu^{118}, Gly^{139}, Val^{140}, Asn^{203}, Pro^{204}, Leu^{215}, Asp^{216}
4OJ2	Aquaporin	Transport protein	-7.97	1431.2435	Leu^{28}, Ala^{31}, Leu^{32}, Asn^{33}, Leu^{38}, Pro^{39}, Ser^{40}, Val^{41}, Ile^{44}, Phe^{48}, Asp^{115}, Ala^{117}, Val^{118}, Asn^{119}, Ala^{120}, Ser^{122}, Ile^{176}, His^{177}, Thr^{179}, Gly^{180}, Arg^{187}
5GNT	BDLP-like folding of Mitofusin 1	Hydrolase	-7.9	1621.646625	Ala^{199}, Asp^{200}, Val^{201}, Phe^{202}, Leu^{227}, Ser^{228}, Lys^{229}, Pro^{230}, Asn^{231}, Arg^{279}, Cys^{327}, Ser^{331}, Lys^{334}, Thr^{335}
5WG5	Human GRK2	Transferase	-7.88	1680.155375	Val^{180}, Asn^{183}, Ile^{184}, His^{185}, Leu^{186}, Asp^{190}, Lys^{210}, Arg^{617}, Lys^{618}, Gln^{633}, Cys^{634}, Asp^{635}, Ser^{636}, Asp^{637}, Leu^{640}
5FN3	Gamma secretase in class 1 of the ap	Hydrolase	-7.87	1700.123625	Asn^{45}, Lys^{46}, Thr^{47}, Ala^{48}, Pro^{49}, Glu^{184}, Trp^{289}, Asn^{290}, Tyr^{545}, Gly^{547}, Asp^{548}, Gly^{549}, Pro^{550}, Leu^{551}, Gln^{552}, Asn^{562}, Trp^{653}, Ile^{656}
6PAX	Human PAX-6 paired domain-DNA co	Gene regulation	-7.82	1843.592625	Asn^{6}, Phe^{12}, Asn^{14}, Gly^{15}, Arg^{16}, Pro^{17}, Leu^{18}, Arg^{23}, Val^{45}, Ser^{46}, Cys^{49}, Lys^{52}, Ile^{53}, Arg^{56}, Pro^{65}, Arg^{66}, Ile^{68}
3PUF	Human RNASE H2 complex	Hydrolase	-7.82	1846.706875	Trp^{193}, Gln^{194}, Phe^{195}, Glu^{197}, Lys^{198}, Leu^{199}, Gln^{200}, Trp^{219}, His^{223}, Pro^{231}, Phe^{233}
6DK3	Human mitochondrial serine hydrox	Transferase	-7.8	1926.296875	Gln^{67}, Asn^{78}, Phe^{79}, Cys^{80}, Leu^{85}, Leu^{88}, Gly^{89}, Asn^{93}, His^{279}, Arg^{286}, His^{330}, Phe^{501}, His^{504}
5HKJ	Single chain recombinant globular h	Signaling protein	-7.79	1939.345875	Arg^{12}, Trp^{60}, Glu^{61}, Asn^{85}, Lys^{110}, Lys^{113}, Lys^{114}, Gly^{115}, His^{116}, Ile^{117}, Gln^{119}
4KTV	MAT enzymes: MATA2B	Transferase	-7.73	2167.87325	Lys^{88}, His^{89}, Gly^{91}, Asp^{93}, Asp^{94}, Ser^{96}, Lys^{97}, Arg^{168}, Arg^{169}, Gly^{171}, Pro^{174}, Trp^{175}, Leu^{176}, Arg^{177}, Asp^{210}
5C5X	The S156E mutant of human aquapo	Transport protein	-7.72	2186.2455	Lys^{2}, Lys^{3}, Glu^{4}, Gln^{81}, Ile^{82}, Ser^{83}, Arg^{86}, Leu^{230}, Ile^{238}, Gly^{241}, Thr^{242}, Tyr^{243}, Glu^{244}, Pro^{245}
6F39	C1R homodimer CUB1-EGF-CUB2	Hydrolase	-7.71	2215.96575	Thr^{35}, Gly^{36}, Tyr^{37}, Arg^{38}, Gln^{91}, Gly^{92}, Asn^{93}, Asp^{125}, Leu^{126}, Asp^{127}, Glu^{128}, Pro^{142}, Tyr^{155}, Phe^{156}, Asp^{168}, Thr^{169}, His^{170}
3HRZ	Human factor B	Immune system	-7.71	2227.2145	Val^{204}, Arg^{205}, Leu^{206}, Lys^{251}, Ser^{295}, Val^{296}, Val^{308}, Thr^{309}, Glu^{310}, Gln^{311}, Ser^{312}, Tyr^{563}, Val^{564}, Leu^{565}, Asn^{566}, Asp^{567}
4ZWJ	Rhodopsin bound to arrestin	Signaling protein	-7.71	2238.5205	Tyr^{43}, Met^{44}, Leu^{46}, Leu^{47}, Ile^{259}, Ala^{260}, Ile^{263}, Cys^{264}, Phe^{293}, Phe^{294}, Ser^{297}, Ala^{298}, Tyr^{301}, Ile^{305}
5Z90	BRD4 bromodomain 1	Transcription	-7.7	2257.4915	Pro^{45}, Pro^{48}, Glu^{49}, Lys^{55}, Tyr^{98}, Ile^{101}, Lys^{102}, Thr^{103}, Pro^{104}, Met^{105}, Arg^{113}, Tyr^{118}, Tyr^{119}, Asp^{128}, Thr^{131}
5D1M	UBCH5B	Ligase	-7.68	2338.9415	Arg^{72}, Ile^{73}, Tyr^{74}, His^{75}, Ile^{78}, Asn^{79}, Ser^{80}, Val^{120}, Glu^{122}, Ile^{123}, Ile^{126}, Lys^{133}, Arg^{136}, Ile^{137}, Glu^{140}, Trp^{141}

PDB ID	Targets	Classification	Energy (Kcal/mol)	Dissoc. constant [nM]	Contacting receptor residues
1IKN	IKAPPABALPHA/NF-KAPPAB complex	Transcription factor	-7.67	2370.73725	Lys^{28}, Ser^{51}, Thr^{52}, Asp^{53}, Lys^{221}, Glu^{222}, Asp^{223}, Ile^{224}, Glu^{225}, Arg^{236}, Gly^{237}, Ser^{238}, Phe^{239}, Ser^{240}, Gln^{241}, Pro^{275}
4UV8	LSD1(KDM1A)-CoRest	Transcription	-7.65	2456.273	Ala^{309}, Arg^{310}, Asp^{311}, Asn^{587}, Ala^{589}, Thr^{607}, Arg^{608}, Ala^{636}, Pro^{755}, Trp^{756}
3OE9	the chemokine CXCR4 receptor	Signaling protein, hy	-7.64	2515.0045	Trp^{94}, Tyr^{116}, Thr^{117}, Leu^{120}, Tyr^{121}, Asp^{171}, Arg^{188}, Phe^{199}, Gln^{200}, Gln^{202}, His^{203}, Tyr^{255}, Ile^{259}, Asp^{262}, Leu^{266}, His^{281}, Ile^{284}, Glu^{288}
3UVU	Flap endonuclease 1 (FEN1)	Protein binding/pept	-7.61	2654.5755	Ala^{148}, Ser^{149}, Gly^{150}, Thr^{155}, Asn^{188}, Gly^{191}, Asp^{192}, Trp^{231}, Asn^{235}, Arg^{238}, Glu^{266}, Ala^{269}, Asp^{270}, Trp^{273}, Tyr^{277}
3VJ9	The human squalene synthase	Transferase	-7.61	2654.5755	Ala^{301}, Ala^{302}, Cys^{303}, Tyr^{304}, Asn^{305}, Asn^{306}, Gln^{308}, Ala^{313}, Val^{314}, Lys^{315}, Ile^{316}, Arg^{317}, Gln^{320}, Tyr^{341}, Arg^{348}
1ZXM	Human topo IIA ATPASE/AMP-PNP	Isomerase	-7.58	2768.98325	Trp^{62}, Ile^{70}, Asn^{71}, Tyr^{72}, Arg^{73}, Met^{273}, Tyr^{274}, Lys^{276}, Asp^{277}, Lys^{278}, Lys^{306}, Gln^{309}
1Z3U	Angiopoietin-2 receptor binding do	Signaling protein	-7.58	2773.6605	Ile^{309}, Lys^{310}, Gly^{331}, Ser^{332}, Val^{333}, Arg^{337}, Glu^{341}, Val^{344}, Gly^{345}, Phe^{346}, Gly^{347}, Asn^{348}, Pro^{349}, Tyr^{353}, Trp^{354}, Leu^{355}, Gly^{356}
4GWG	6-phosphogluconate dehydrogenase	Oxidoreductase	-7.51	3126.77775	Met^{13}, Val^{127}, Ser^{128}, Gly^{129}, Gly^{130}, Glu^{131}, Glu^{132}, Lys^{183}, His^{186}, Asn^{187}, Glu^{190}, Phe^{230}, Gly^{364}, Cys^{365}, Ile^{366}, Arg^{368}
5U7Z	Human acid ceramidase (ASAH1, aCD	Hydrolase	-7.46	3396.366	Ile^{76}, Val^{77}, Leu^{80}, Glu^{129}, Ser^{132}, Phe^{133}, Asn^{134}, Ile^{135}, Phe^{136}, Tyr^{137}
4IRG	The ETS transcription factor ERG	DNA binding protein	-7.44	3548.72825	Gly^{292}, Gln^{293}, Ile^{294}, Gln^{295}, Leu^{296}, Trp^{334}, Lys^{338}, Lys^{340}, Met^{343}, Lys^{347}, Arg^{350}, Ala^{351}, Tyr^{354}, Tyr^{355}, Lys^{358}
4MXV	Lymphotoxin alpha	Cytokine/immune sys	-7.41	3695.43	Leu^{43}, Leu^{44}, Trp^{45}, Ser^{61}, Asn^{62}, Asn^{63}, Ser^{64}, Gln^{102}, Phe^{104}, Phe^{110}, His^{111}, Ser^{148}, Thr^{149}, His^{150}, Thr^{151}
4E+49	Carbonic anhydrase (CA)	Lyase	-7.4	3783.79075	Trp^{5}, Phe^{20}, Asn^{62}, His^{64}, Ala^{65}, Asn^{67}, Gln^{92}, His^{94}, His^{96}, Leu^{198}, Thr^{200}, Pro^{201}, Pro^{202}
1HF0	DNA-binding domain of OCT-1	Transcription	-7.39	3835.228	Asp^{41}, Phe^{42}, Ser^{43}, Thr^{46}, Arg^{49}, Asn^{59}, Lys^{62}, Leu^{63}, Arg^{102}, Lys^{103}, Lys^{104}, Arg^{105}
3OHM	Activated G alpha Q	Signaling protein	-7.34	4165.89825	Asp^{146}, Arg^{147}, Arg^{148}, Arg^{149}, Lys^{158}, Asp^{236}, Glu^{281}, Lys^{282}, Met^{284}, Tyr^{285}, Ser^{286}
3I69	Apo Glutathione transferase A1-1 Gl	Transferase	-7.3	4434.3505	Met^{108}, Pro^{110}, Phe^{111}, Cys^{112}, Pro^{113}, Pro^{114}, Glu^{210}, Val^{213}, Arg^{214}, Val^{216}, Tyr^{217}, Phe^{220}
4JXJ	ribosomal RNA small subunit methy	Transferase	-7.3	4471.931	Ile^{151}, Cys^{152}, Ala^{153}, Lys^{154}, Pro^{155}, Ser^{164}, Cys^{167}, Ser^{168}, Leu^{169}, Val^{173}, Leu^{200}, Thr^{257}, Pro^{258}, Asn^{259}
3DOF	Complex of ARL2 and BART	Signaling protein/hy	-7.26	4784.2675	Thr^{30}, Lys^{34}, Glu^{39}, Thr^{43}, Ile^{44}, Ser^{45}, Pro^{46}, Thr^{47}, Leu^{48}, Gly^{49}, Phe^{50}, Asn^{51}, Lys^{53}
5FFG	Integrin alpha V Beta 6 head	Cell adhesion	-7.25	4816.6765	Tyr^{18}, Phe^{21}, Pro^{41}, Trp^{93}, Ala^{96}, Pro^{110}, Leu^{111}, Gln^{156}, Phe^{159}, Pro^{174}, Trp^{179}, Tyr^{221}, Tyr^{224}
4URW	RAS:SOS complex	Signaling protein	-7.24	4923.5305	Pro^{34}, Thr^{35}, Ile^{36}, Glu^{37}, Ser^{39}, Tyr^{40}, Arg^{41}, Asp^{54}, Ile^{55}, Leu^{56}, Asp^{57}, Thr^{58}, Ala^{59}, Gln^{61}, Thr^{74}
5F6D	UBC9 (K48A/K49A/E54A)	Ligase	-7.22	5066.847	Pro^{79}, Pro^{80}, Leu^{81}, Phe^{82}, Glu^{132}, Arg^{147}, Ala^{150}, Gln^{151}, Lys^{154}, Phe^{155}
5UZP	Citrate synthase	Transferase	-7.12	6028.896	Gln^{45}, Arg^{191}, Thr^{192}, Tyr^{194}, Ser^{280}, Ala^{281}, Leu^{282}, Arg^{440}, Ala^{441}, Leu^{442}, Gly^{443}, Phe^{444}, Pro^{445}, Leu^{446}
3R4O	Heat shock protein 90	Chaperone	-7.01	7332.7505	Leu^{80}, Glu^{200}, Ile^{203}, Lys^{204}, Val^{207}, Lys^{208}, Ser^{211}, Gln^{212}, Tyr^{216}, Pro^{217}, Ile^{218}, Thr^{219}, Leu^{220}
5A2E	Extracellular SRCR domains of huma	Immune system	-7	7369.974	Ser^{52}, Ser^{53}, Cys^{54}, Glu^{157}, Arg^{159}, Arg^{225}, Trp^{238}, Asp^{239}, Cys^{240}, Pro^{241}, Gly^{242}, Leu^{243}, Pro^{244}
4A7U	Human I113T SOD1	Oxidoreductase	-6.99	7470.162	His^{48}, Thr^{58}, Gly^{61}, Pro^{62}, His^{63}, His^{80}, His^{120}, Lys^{136}, Thr^{137}, Asn^{139}, Ala^{140}, Gly^{141}, Ser^{142}, Arg^{143}
2UW9	PKB-Beta (AKT2)	Transferase	-6.98	7648.78	Trp^{334}, Phe^{350}, Glu^{365}, Glu^{366}, Ile^{367}, Phe^{369}, Lys^{378}, Ser^{379}, Ala^{382}, Gly^{383}, Leu^{385}, Lys^{386}, Lys^{387}, Gln^{391}, Arg^{392}, Leu^{393}, His^{406}
5FUG	A human YL1-H2A.Z-H2B complex	DNA binding protein	-6.92	8449.67	Ser^{73}, Lys^{74}, Asp^{75}, Lys^{77}, Val^{78}, Lys^{79}, Arg^{80}, Thr^{82}, Arg^{84}, His^{85}, Leu^{88}
1JT3	Human acidic fibroblast growth fact	Hormone/growth fac	-6.9	8710.316	Leu^{3}, Gly^{6}, Asn^{7}, Tyr^{8}, Lys^{12}, Leu^{46}, Ser^{47}, Ala^{48}, Glu^{49}, Ser^{50}, Val^{51}, Val^{54}, Glu^{87}
4IP9	Human serum amyloid A1	Protein binding	-6.9	8813.836	Asp^{16}, Arg^{19}, Tyr^{35}, Arg^{39}, Tyr^{42}, Asp^{43}, Lys^{46}, Arg^{47}, Asp^{91}, Pro^{92}, Asn^{93}, Arg^{96}, Lys^{103}, Tyr^{104}
4Y5O	CCM2 HHD in complex with MEKK3	Transferase	-6.89	8963.86	Leu^{299}, Tyr^{302}, Leu^{322}, Tyr^{325}, Arg^{326}, Asn^{327}, Leu^{353}, Phe^{356}, Ile^{357}, Pro^{358}
5W45	APOBEC3H	DNA binding protein	-6.84	9622.357	Leu^{11}, Gln^{12}, Asn^{14}, Lys^{16}, Arg^{18}, Leu^{19}, Arg^{20}, Pro^{25}, Lys^{27}, Thr^{81}, Trp^{82}, Arg^{110}
2FM5	PDE4D2	Hydrolase	-6.84	9671.203	Lys^{85}, Glu^{89}, Leu^{92}, Ala^{93}, Leu^{96}, Glu^{97}, Val^{99}, Thr^{119}, Val^{120}, His^{123}, Thr^{124}, Gln^{127}, Glu^{128}, Ile^{143}, Leu^{146}
2CF9	Recombinant human thrombin	Hydrolase/hydrolase	-6.77	10920.869	Leu^{60}, Ile^{90}, His^{91}, Pro^{92}, Arg^{93}, Tyr^{94}, Asn^{95}, Trp^{96}, Arg^{97}, Asp^{100}, Arg^{101}
3BYI	Human rho GTPASE activating prote	Signaling protein	-6.74	11468.708	Phe^{372}, Glu^{373}, Val^{376}, Glu^{377}, Lys^{380}, Lys^{381}, Glu^{444}, Thr^{445}, Gly^{446}, Asn^{447}, His^{451}, Tyr^{454}
5N7E	DBL-homology domain of BCR-ABL	Signaling protein	-6.69	12436.522	Tyr^{37}, Gln^{47}, Glu^{48}, Phe^{49}, Glu^{50}, Val^{51}, Pro^{52}, Lys^{55}, Thr^{57}, Ala^{58}, Thr^{59}, Ile^{60}, Ser^{61}
4W9O	The FK1 domain of FKBP51	Isomerase	-6.69	12584.328	Lys^{52}, Tyr^{54}, Arg^{73}, Glu^{75}, Pro^{76}, Phe^{77}, Val^{78}, Leu^{135}, Asp^{136}, Lys^{138}
5AIU	RNF4-ring domain, UBC13-UB (isope	Ligase/signaling prot	-6.64	13600.3	Tyr^{143}, Tyr^{193}, Ile^{194}, Gly^{195}, Ser^{196}, Thr^{198}, Val^{199}, Ser^{200}, Cys^{201}, Pro^{202}, His^{225}, Val^{226}
4X1L	Mutation-induced destabilization of	Protein binding	-6.61	14186.45	Gly^{3}, Trp^{4}, Tyr^{7}, Gly^{24}, Trp^{32}, Lys^{108}, Thr^{109}, Leu^{135}, Ser^{138}, Tyr^{140}
2Q7N	LIF receptor (domains 1-5)	CYTOKINE receptor/C	-6.61	14330.844	Phe^{100}, Trp^{198}, Asn^{201}, Thr^{202}, Thr^{204}, Asn^{205}, Val^{206}, Phe^{207}, Gln^{209}, Val^{274}, Tyr^{275}, Gly^{276}, Thr^{277}
5OKF	Human 14-3-3 sigma with the hspb6	Signaling protein	-6.6	14599.396	Trp^{59}, Glu^{66}, Gln^{67}, Asn^{70}, Arg^{85}, Ala^{132}, Glu^{133}, Val^{134}, Ala^{135}, Thr^{136}, Lys^{140}, Glu^{182}, Ile^{183}
4ZMV	Human P-cadherin (SS-X-dimer pock	Cell adhesion	-6.55	15698.361	Ser^{8}, Arg^{68}, Ala^{72}, Lys^{73}, Ile^{97}, Val^{98}, Thr^{99}, Asp^{100}, Asp^{137}, Tyr^{140}, Thr^{141}
5VZ3	Growth factor	Signaling protein	-6.54	16128.072	Pro^{36}, Glu^{38}, Val^{39}, Gln^{40}, Thr^{42}, Asn^{84}, Pro^{85}, Met^{86}, Val^{87}, Leu^{105}
3WO2	Human interleukin-18	Immune system	-6.48	17786.768	Gly^{25}, Asn^{26}, Arg^{27}, Pro^{28}, Lys^{79}, Ile^{80}, Ile^{81}, Ser^{82}, Phe^{83}, Glu^{128}, Glu^{130}, Lys^{135}, Ile^{137}, Leu^{138}, Lys^{139}
1QE6	Interleukin-8	Immune system	-6.44	18901.026	Tyr^{13}, Ser^{14}, Lys^{15}, Pro^{16}, Phe^{17}, His^{18}, Pro^{19}, Lys^{20}, Leu^{51}, Glu^{55}, Trp^{57}, Val^{61}, Lys^{64}
5M3D	Tuning of CD81LEL (space group p31	Cell adhesion	-6.44	19093.406	Thr^{161}, Asn^{184}, Leu^{185}, Phe^{186}, Lys^{187}, Glu^{188}, Asp^{189}, Gln^{192}, Asp^{195}, Asp^{196}, Lys^{201}
4NYH	Pir1 dual specificity phosphatase co	Hydrolase	-6.43	19484.064	Leu^{39}, Pro^{40}, Val^{41}, Gln^{43}, Pro^{67}, Glu^{68}, Glu^{69}, Cys^{70}, Phe^{71}, Asp^{75}, Lys^{79}, Glu^{82}, Gln^{83}
2X1X	Structure of VEGF-C in complex with	Hormone/signaling p	-6.43	19484.064	Asp^{139}, Val^{140}, Gly^{141}, Lys^{142}, Gly^{145}, Ala^{147}, Thr^{148}, Thr^{150}, Phe^{151}, Phe^{152}, Pro^{155}
2J8B	Human CD59	Lipid binding protein	-6.38	21199.716	Tyr^{4}, Asp^{12}, Lys^{14}, Lys^{66}, Asp^{67}, Leu^{68}, Cys^{69}, Phe^{71}, Asn^{72}, Glu^{73}, Gln^{74}
3H91	Human chromobox homolog 2 (CBX	Transcription	-6.37	21560.564	Val^{11}, Phe^{12}, Ala^{13}, Ala^{14}, Glu^{15}, Cys^{16}, Ile^{17}, Asn^{48}, Ile^{49}, Leu^{50}, Asp^{51}, Arg^{53}, Leu^{54}, Ala^{57}
2IYB	Complex between the 3rd LIM doma	Metal-binding	-6.35	22038.866	Tyr^{16}, Trp^{23}, Lys^{69}, Asn^{71}, Ala^{73}, Phe^{77}, Gln^{79}, Trp^{80}, Arg^{81}, Val^{86}
5H0V	H88A mutated human transthyretin	Transport protein	-6.12	32437.582	Ala^{19}, Val^{20}, Leu^{110}, Leu^{111}, Ser^{112}, Pro^{113}, Tyr^{114}, Ser^{115}, Tyr^{116}, Ser^{117}
1CWF	Human cyclophilin A	Isomerase/immunosu	-6.05	37001.832	Thr^{68}, Arg^{69}, His^{70}, Thr^{73}, Gly^{74}, Gly^{75}, Lys^{76}, Glu^{81}, Lys^{82}, Val^{6}
5POQ	BRD1 in complex with N10974A	Gene regulation	-6.01	39586.184	Leu^{92}, His^{93}, Glu^{96}, Gly^{128}, Arg^{132}, Arg^{135}, Arg^{136}, Asp^{139}
3KLT	A vimentin fragment	Structural protein	-5.81	55294.196	Asp^{125}, Arg^{126}, Phe^{232}, Arg^{233}, Leu^{234}, Lys^{235}, Lys^{236}, Gln^{239}
1BL1	PTH receptor N-terminus fragment	Hormone receptor	-5.8	55668.764	Tyr^{13}, Lys^{15}, Pro^{16}, Phe^{17}, His^{18}, Phe^{21}, Leu^{43}, Ser^{44}, Asp^{45}, Arg^{47}, Leu^{49}
