# Supplementary material for: Effectiveness of Natural Antioxidants against SARS-CoV-2? Insights from the In-Silico World
Source: Antibiotics (Basel). 2021 Aug 20;10(8):1011. doi: 10.3390/antibiotics10081011 (PMC8388999; doi:10.3390/antibiotics10081011)
Supplement: Supplementary file 1 [file antibiotics-10-01011-s001.zip › Suppl Material/Table S2 c.pdf]

Table S2c **Hesperidine docking to nonspecific human blood proteins**

PDB ID	Targets	Classification	Energy (kcal/mol)	Dissoc. constant [nM]	Contacting receptor residues
5FFG	Integrin alpha V Beta 6 head	Cell adhesion	-10.89	10.4293486	Phe^{21}, Val^{23}, Ala^{96}, Arg^{99}, Lys^{101}, Phe^{159}, Ser^{160}, Asp^{162}, Phe^{163}, Thr^{164}, Lys^{165}, Tyr^{224}, Ser^{225}, Val^{226}, Ala^{227}, Val^{228}, Asp^{237}, Phe^{278}, Ser^{279}, Val^{280}, Ala^{281}, Ala^{282}, Ala^{343}, Ile^{344}, Ala^{345}, Pro^{346}, Tyr^{407}, Ser^{408}, Met^{409}, Lys^{410}
4GWN	Human mature MEPRIN Beta	Hydrolase	-10.58	17.6884277	Cys^{124}, Trp^{125}, Ser^{126}, Thr^{149}, His^{152}, Glu^{153}, His^{156}, His^{162}, Arg^{184}, His^{186}, Asn^{187}, Val^{208}, Met^{209}, His^{210}, Tyr^{211}, Ser^{212}, Ala^{215}, Phe^{216}, Gly^{236}, Gln^{237}, Arg^{238}
3I7H	Ddb1 (damage specific DNA binding protein 1)	Protein binding	-10.27	29.4979043	Trp^{411}, Pro^{412}, Tyr^{422}, Ser^{502}, Cys^{503}, Asn^{504}, Cys^{540}, Leu^{541}, Asp^{542}, Ile^{543}, Thr^{544}, Pro^{545}, Leu^{546}, Arg^{589}, Ser^{590}, Ile^{591}, Leu^{592}, Met^{593}, Thr^{594}, Thr^{595}, Val^{637}, Arg^{639}, Thr^{640}, Arg^{642}, Cys^{680}, Pro^{681}
4UV8	LSD1(KDM1A)-CoRest	Transcription	-10.1	39.3009297	Gly^{330}, Ala^{331}, Val^{333}, Thr^{335}, Phe^{538}, Ala^{539}, Asn^{540}, Trp^{552}, Asp^{553}, Gln^{554}, Asp^{555}, Asp^{556}, Glu^{559}, His^{564}, Leu^{659}, Lys^{661}, Ser^{760}, Tyr^{761}, Ser^{762}, Tyr^{763}, Val^{764}, Tyr^{773}, Tyr^{807}, Pro^{808}, Ala^{809}, Thr^{810}, His^{812}
2CF9	Recombinant human thrombin	Hydrolase/hydrolase inhibitor	-9.94	51.8341992	Arg^{35}, Glu^{39}, Leu^{40}, Leu^{41}, Cys^{42}, His^{57}, Cys^{58}, Trp^{60}d, Lys^{60}f, Phe^{60}h, Asp^{189}, Ala^{190}, Cys^{191}, Glu^{192}, Ser^{195}, Val^{213}, Ser^{214}, Trp^{215}, Gly^{216}, Gly^{219}, Cys^{220}, Gly^{226}, Phe^{227}, Tyr^{228}
2FM5	PDE4D2	Hydrolase	-9.88	57.0686953	Tyr^{159}, His^{160}, His^{164}, His^{204}, Ser^{208}, Asn^{209}, Gln^{210}, Leu^{229}, Glu^{230}, Thr^{271}, Asp^{272}, Met^{273}, Ser^{274}, Asp^{318}, Leu^{319}, Asn^{321}, Pro^{322}, Tyr^{329}, Trp^{332}, Ile^{336}, Phe^{340}, Gln^{343}, Ser^{355}, Pro^{356}, Met^{357}, Cys^{358}, Gln^{369}, Phe^{372}
6FFI	MGLUR5 in complex with MMPEP	Membrane protein	-9.77	68.7114375	Leu^{635}, Gln^{647}, Arg^{648}, Ile^{651}, Gly^{652}, Met^{880}, Tyr^{890}, Leu^{891}, Ile^{892}, Cys^{893}, Asn^{894}, Thr^{895}, Thr^{896}, Gly^{899}, Val^{900}, Pro^{903}, Leu^{904}, Tyr^{952}, Asn^{956}, Tyr^{957}, Lys^{958}, Ile^{959}, Ile^{960}, Met^{962}
4WB7	Camp-dependent protein kinase a (catalytic alpha subunit)	Transferase	-9.46	116.7331172	Lys^{2}, Asp^{3}, Tyr^{4}, Thr^{7}, Glu^{42}, Lys^{43}, Glu^{46}, Glu^{49}, Ala^{50}, Phe^{336}, Asn^{341}, Asn^{348}, His^{349}, Lys^{350}, Phe^{352}, Ala^{353}
5HEX	Human hexokinase 2	Transferase	-9.36	136.8034375	Asp^{84}, Leu^{85}, Gly^{86}, Asn^{89}, Phe^{90}, Arg^{91}, Ile^{111}, Ser^{155}, Phe^{156}, Asp^{209}, Ile^{229}, Gly^{231}, Thr^{232}, Gly^{233}, Ser^{234}, Asn^{235}, Glu^{260}, Gly^{262}, Glu^{294}, Asp^{413}, Gly^{414}, Ser^{415}, Ser^{445}, Glu^{446}, Asp^{447}, Gly^{448}, Ser^{449}
4FAD	PI3KA	Transferase	-9.35	141.261625	Trp^{201}, Leu^{207}, Pro^{208}, Tyr^{210}, Leu^{211}, Arg^{277}, Glu^{279}, Gln^{291}, Trp^{292}, Arg^{294}, His^{295}, Lys^{298}, Asn^{299}, Asp^{788}, Arg^{849}, Glu^{852}, Glu^{856}, Leu^{865}, Pro^{866}, Tyr^{867}, Glu^{880}
4U7D	Human RECQ-like helicase	Hydrolase	-9.33	143.9087813	Phe^{87}, Lys^{88}, Leu^{89}, Arg^{93}, Thr^{115}, Gly^{116}, Gly^{118}, Lys^{119}, Ser^{120}, Leu^{121}, Tyr^{123}, Cys^{138}, Leu^{143}, Asp^{146}, Gln^{147}, Val^{150}, Leu^{154}, Asp^{219}, Glu^{220}, Leu^{253}, Gly^{377}, Ile^{378}, Asp^{379}, Arg^{407}
4NY0	Human focal adhesion kinase	Transferase	-9.23	171.2329219	Arg^{86}, Ser^{88}, Val^{95}, Arg^{125}, Ile^{126}, Arg^{127}, Leu^{129}, Phe^{147}, Gln^{150}, Asp^{154}, Glu^{158}, Tyr^{251}, Arg^{252}, Phe^{253}, Lys^{255}, Glu^{256}, Cys^{257}, Asn^{339}
4OJ2	Aquaporin	Transport protein	-9.18	186.9406719	His^{61}, Ile^{62}, Ser^{63}, Gly^{64}, His^{66}, Cys^{75}, Gly^{78}, Cys^{79}, His^{80}, Val^{81}, Arg^{85}, Tyr^{89}, Ile^{145}, Phe^{146}, Thr^{149}, Asp^{150}, Glu^{151}, Arg^{152}, Tyr^{219}, Asn^{220}, Tyr^{221}, Pro^{225}, Ala^{227}
1OE9	Myosin V motor	ATPASE/myosin	-9.15	195.9873594	Thr^{212}, Arg^{213}, Phe^{251}, Gln^{252}, Ala^{253}, Glu^{254}, Glu^{256}, Gln^{290}, Gly^{291}, Phe^{444}, Glu^{445}, Ile^{446}, Ser^{448}, Glu^{450}, Gln^{451}, Glu^{562}, Gly^{563}, Lys^{567}
4JXJ	Ribosomal RNA small subunit methyltransferase a from rickettsia bellii	Transferase	-9	252.4524063	Asn^{23}, Ile^{49}, Gly^{50}, Ile^{71}, Glu^{72}, Thr^{73}, Asp^{74}, Gln^{96}, Asp^{97}, Ala^{98}, Leu^{99}, Asn^{116}, Leu^{117}, Pro^{118}, Tyr^{119}, His^{120}, Ile^{121}
5C5X	The S156E mutant of human aquaporin 5	Transport protein	-8.87	314.9225625	Ser^{64}, Gly^{65}, His^{67}, Leu^{76}, Asn^{80}, Gln^{81}, Ile^{82}, Arg^{86}, Tyr^{90}, Ile^{146}, Phe^{147}, Thr^{150}, Asp^{151}, Ser^{152}, Arg^{153}, Phe^{222}, Asn^{228}, Ile^{238}, Tyr^{243}, Pro^{245}
5WG5	Human GRK2	Transferase	-8.87	314.9225625	Ile^{32}, Leu^{33}, Pro^{35}, Asn^{179}, Val^{180}, Asn^{183}, Ile^{184}, His^{185}, Leu^{186}, Lys^{210}, Asp^{212}, Trp^{517}, Trp^{576}, Glu^{616}, Arg^{617}, Lys^{618}, Gln^{633}, Cys^{634}, Asp^{635}, Ser^{636}, Asp^{637}, Leu^{640}
3R6I	AKR1C3	Oxidoreductase	-8.86	319.2036563	Tyr^{24}, Pro^{26}, Pro^{27}, Leu^{54}, Tyr^{55}, Asn^{56}, Trp^{86}, His^{117}, Ser^{118}, Met^{120}, Leu^{122}, Asn^{167}, Tyr^{216}, Arg^{226}, Trp^{227}, Phe^{306}, Phe^{311}, Pro^{318}
2QFZ	Human tbc1 domain family member 22a	Hydrolase activator	-8.77	374.7173438	Ser^{236}, Phe^{329}, Cys^{366}, Lys^{369}, Leu^{370}, Asp^{372}, Gly^{373}, Ile^{374}, Gln^{375}, Asp^{376}, Asn^{377}, Gly^{384}, Met^{387}, Lys^{388}, Trp^{439}, Tyr^{442}, Gln^{443}, Glu^{445}, Pro^{446}, Asp^{447}, Gly^{448}, Phe^{449}, Ser^{450}
4KTV	MAT enzymes: MATA2B	Transferase	-8.74	389.5498125	Glu^{27}, Gln^{36}, Asp^{43}, His^{89}, Glu^{148}, Val^{155}, His^{158}, Lys^{159}, Ala^{162}, Glu^{166}, Gln^{372}, Arg^{373}, Ala^{376}, Tyr^{377}, Gly^{378}, Phe^{380}, Gly^{381}, Arg^{382}
4MWS	Human PPCA (trigonal crystal form 1)	Hydrolase	-8.71	411.8625938	Leu^{67}, Thr^{68}, Leu^{74}, Val^{75}, Gln^{76}, Asn^{245}, Tyr^{247}, Asn^{248}, Ala^{251}, Pro^{252}, Ala^{254}, Cys^{303}, Thr^{304}, Asn^{305}, Ala^{308}, Met^{430}, Thr^{433}, Asp^{434}, Lys^{435}, Pro^{436}, Leu^{437}
4JKJ	The S18Y variant of ubiquitin carboxy-terminal hydrolase l1	Hydrolase	-8.7	419.5806563	Glu^{7}, Asn^{9}, Pro^{10}, Glu^{11}, Asn^{14}, Leu^{17}, Tyr^{18}, Val^{22}, Ala^{23}, Gly^{24}, Trp^{26}, Arg^{27}, Val^{29}, Asp^{30}, Glu^{109}
5FN3	Gamma secretase in class 1 of the apo- state ensemble	Hydrolase	-8.69	430.338875	Leu^{260}, Lys^{261}, Pro^{262}, Glu^{310}, Gln^{313}, Lys^{314}, Ala^{315}, Pro^{316}, Val^{318}, Thr^{517}, Arg^{520}, Trp^{533}, Asn^{573}, Leu^{574}, Thr^{575}, Gly^{576}, Thr^{577}, Val^{578}, Tyr^{600}, Arg^{622}, Ser^{623}, Thr^{624}
6DK3	Human mitochondrial serine hydroxymethyltransferase 2	Transferase	-8.66	448.88575	Gly^{99}, Asp^{113}, Glu^{116}, Leu^{117}, Gln^{120}, Arg^{121}, Leu^{124}, Glu^{125}, Pro^{131}, Gly^{135}, Val^{136}, Gln^{139}, Tyr^{292}, Lys^{294}, Glu^{312}, Asn^{316}
5HZJ	Intersectin1 containing wildtype LOV2 domain	Signaling protein	-8.58	512.912875	Gln^{1470}, Leu^{1588}, Ser^{1589}, Glu^{1590}, Gln^{1591}, Leu^{1592}, Val^{1593}, Ser^{1596}, Val^{1597}, Thr^{1598}, Asn^{1599}, Arg^{1604}, Phe^{1629}, Thr^{1661}, Pro^{1662}, Phe^{1664}, Asn^{1666}, Glu^{1667}, His^{1687}
1W6K	Human OSC	Isomerase	-8.54	552.453875	Asp^{220}, Trp^{221}, Ser^{227}, Thr^{228}, Tyr^{430}, Gln^{431}, Lys^{432}, Tyr^{434}, Arg^{435}, Gln^{436}, Met^{437}, Asn^{492}, Met^{493}, Arg^{494}, Asn^{495}, Pro^{496}, Thr^{505}, Lys^{506}, Arg^{507}
4ZWJ	Rhodopsin bound to arrestin	Signaling protein	-8.47	614.4345625	Lys^{66}, Lys^{67}, Arg^{69}, Thr^{70}, Pro^{71}, Tyr^{74}, Gly^{149}, Glu^{150}, Asn^{151}, His^{152}, Arg^{2067}, Gln^{2070}, Glu^{2071}, Asp^{2072}, Gln^{2138}, Asp^{2139}, Val^{2140}, Gly^{2141}, Lys^{2142}, Arg^{2292}
3HRZ	Human factor B	Immune system	-8.46	624.893125	Arg^{12}, Glu^{16}, Glu^{17}, Gln^{18}, Thr^{62}, Thr^{64}, Ser^{104}, Phe^{105}, Met^{128}, Asp^{129}, Asn^{463}, Lys^{464}, Lys^{466}, Ile^{467}, Phe^{468}, Asp^{491}, Met^{523}
5W45	APOBEC3H	DNA binding protein	-8.42	675.3426875	Leu^{11}, Gln^{12}, Asn^{14}, Lys^{16}, Arg^{17}, Arg^{18}, Leu^{19}, Arg^{20}, Pro^{22}, Tyr^{23}, Tyr^{24}, Pro^{25}, Arg^{26}, Lys^{27}, Thr^{81}, Trp^{82}, Arg^{110}
3EHT	The extracellular domain of human corticotropin releasing factor receptor type 1 (crfr1)	Membrane protein	-8.36	749.84375	His^{285}, Asp^{254}, Ala^{253}, Arg^{251}, Tyr^{250}, Asn^{249}, Gly^{248}, Tyr^{178}, Tyr^{173}, Ile^{171}, Glu^{21}, Ile^{20}, Met^{19}, Pro^{18}, Asn^{17}, Ile^{16}, Pro^{15}, Gln^{14}
2V5Y	The receptor protein tyrosine phosphatase mu ectodomain	Hydrolase	-8.34	775.5878125	Cys^{400}, Tyr^{401}, Gln^{402}, Val^{403}, Gly^{404}, Glu^{407}, Ser^{430}, Pro^{431}, Tyr^{432}, Thr^{433}, Asn^{434}, Glu^{485}, Gln^{488}, Thr^{489}, Tyr^{490}, Gly^{491}, Val^{492}, Ile^{493}
3VJ9	The human squalene synthase	Transferase	-8.24	918.18875	Glu^{165}, Asp^{169}, Glu^{202}, Asn^{205}, Ser^{206}, Leu^{209}, Lys^{213}, Arg^{238}, Tyr^{239}, Glu^{261}, Thr^{264}, Asn^{265}, His^{268}, His^{269}, Asp^{272}, Pro^{354}, Ser^{355}, Lys^{358}

PDB ID	Targets	Classification	Energy (kcal/mol)	Dissoc. constant [nM]	Contacting receptor residues
5T40	Human EXOG (hexog)	Hydrolase	-8.23	935.3950625	Asp^{108}, Arg^{109}, Lys^{110}, Cys^{112}, Phe^{114}, Ser^{137}, Arg^{138}, His^{140}, Pro^{143}, Ala^{144}, Gly^{145}, Phe^{158}, Phe^{168}, Asn^{176}, Glu^{179}, Arg^{183}, Arg^{314}
5UZP	Citrate synthase	Transferase	-8.21	952.9238125	Met^{72}, Arg^{73}, Gly^{74}, Met^{75}, Gly^{77}, Leu^{78}, His^{265}, Gly^{268}, Asn^{269}, Val^{270}, His^{273}, Leu^{300}, His^{301}, Gly^{348}, Val^{349}, Arg^{356}, Asn^{400}, Val^{401}, Asp^{402}, Arg^{428}, Glu^{447}
3MWE	Truncated human ATP-citrate lyase	Transferase	-8.21	962.623	Ser^{7}, Asp^{60}, Gln^{61}, Gly^{100}, Asn^{104}, Glu^{237}, Pro^{239}, Pro^{240}, Pro^{241}, Phe^{242}, Gly^{243}, Arg^{244}, Glu^{245}, Ala^{246}, Glu^{250}, Ala^{251}, Ala^{254}
2XMS	Human NDRG2 protein	Signaling protein	-8.18	1002.417063	Asp^{158}, Lys^{162}, His^{170}, Thr^{173}, Ser^{177}, Glu^{181}, Met^{182}, Gly^{185}, His^{186}, Ser^{189}, Gln^{190}, Leu^{193}, Trp^{220}, Tyr^{223}, Asn^{224}, Arg^{226}, Pro^{252}, His^{253}, Asp^{255}, Ala^{256}
5K10	Isocitrate dehydrogenase (IDH1)	Oxidoreductase	-8.11	1143.465875	Pro^{206}, Tyr^{208}, Tyr^{231}, Lys^{236}, Phe^{239}, Glu^{240}, Lys^{243}, Ile^{244}, Trp^{245}, Glu^{247}, Arg^{249}, Gln^{257}, Ser^{261}, Glu^{262}, Gly^{263}, Gly^{264}, Phe^{265}
2J0S	Exon junction	Hydrolase	-8.09	1172.784875	Pro^{114}, Thr^{115}, Arg^{116}, Gly^{142}, Thr^{163}, Pro^{164}, Gly^{165}, Arg^{166}, Phe^{197}, Gln^{200}, Asn^{285}, Thr^{286}, Lys^{287}, Gly^{309}, Asp^{310}, Arg^{316}, Thr^{334}, Asp^{335}, Val^{336}
3PUF	Human RNASE H2 complex	Hydrolase	-8.08	1186.72325	Glu^{73}, Arg^{186}, Asp^{187}, Val^{190}, Lys^{191}, Trp^{193}, Gln^{194}, Phe^{195}, Glu^{197}, Lys^{198}, Leu^{199}, Gln^{200}, Thr^{204}, Tyr^{206}, Gly^{207}, Ser^{208}, Trp^{219}, Phe^{233}
3DOF	Complex of ARL2 and BART	Signaling protein/hydrolase	-8.06	1233.697375	Lys^{9}, Gln^{12}, Glu^{82}, Ser^{83}, Thr^{84}, Asp^{85}, Arg^{114}, Leu^{115}, Ala^{116}, Gly^{117}, Ala^{118}, Ile^{176}, Arg^{179}, Ile^{180}, Thr^{182}, Ala^{183}, Glu^{186}, His^{187}
3BYI	Human rho GTPASE activating protein 15 (arhgap15)	Signaling protein	-8.04	1278.209	Ile^{278}, Arg^{289}, Glu^{290}, Trp^{296}, Phe^{297}, Gln^{300}, Glu^{303}, Ala^{304}, Val^{332}, Glu^{335}, Glu^{336}, Lys^{337}, Leu^{338}, Asn^{339}, Leu^{340}, Asp^{341}
1IKN	IKAPPABALPHA/NF-KAPPAB complex	Transcription factor	-8.04	1289.0415	Lys^{28}, Glu^{49}, Arg^{50}, Ser^{51}, Thr^{52}, Glu^{222}, Asp^{223}, Ile^{224}, Glu^{225}, Val^{226}, Glu^{234}, Ala^{235}, Arg^{236}, Gly^{237}, Ser^{238}, Phe^{239}, Pro^{275}
3EHI	Human thymidyalte synthase M190K	Transferase	-8.01	1337.806	Arg^{50}, Thr^{51}, Glu^{87}, Phe^{91}, Asp^{116}, Tyr^{135}, Asn^{183}, Pro^{184}, His^{196}, Gln^{214}, Ser^{216}, Gly^{217}, Asp^{218}, Gly^{222}, Phe^{225}, Asn^{226}, His^{256}, Tyr^{258}
6F39	C1R homodimer CUB1-EGF-CUB2	Hydrolase	-7.98	1421.61325	Thr^{35}, Gly^{36}, Tyr^{37}, Arg^{38}, Ser^{62}, Lys^{66}, Leu^{68}, Met^{89}, Ser^{90}, Gln^{91}, Gly^{92}, Asn^{93}, Lys^{94}, Met^{95}, Asp^{125}, Leu^{126}, Glu^{128}, Tyr^{155}
5AIU	RNF4-ring domain, UBC13-UB (isopeptide crosslink)	Ligase/signaling protein	-7.97	1440.938875	Tyr^{143}, Val^{147}, Gly^{150}, Arg^{151}, Leu^{152}, Ile^{153}, Ile^{192}, Tyr^{193}, Ile^{194}, Gly^{195}, Ser^{196}, Val^{199}, Ser^{200}, Cys^{201}, Pro^{202}, His^{225}, Val^{226}, Thr^{244}
2UW9	PKB-Beta (AKT2)	Transferase	-7.96	1455.60525	Ala^{382}, Gly^{383}, Lys^{386}, Lys^{390}, Gln^{391}, Arg^{392}, Leu^{393}, Gly^{394}, Gly^{395}, Gly^{396}, Pro^{397}, Ser^{398}, Glu^{402}, Glu^{405}, His^{406}, Arg^{407}
3GR4	Human pyruvate kinase M2	Transferase	-7.96	1460.527125	Arg^{255}, Asn^{264}, Ile^{265}, Lys^{266}, Ile^{267}, Glu^{285}, Ala^{286}, Ser^{287}, Asp^{288}, Gly^{321}, Lys^{322}, Pro^{323}, Lys^{433}, Ser^{434}, Arg^{436}, His^{439}, Arg^{443}, Asn^{456}, Gln^{462}, Tyr^{466}, Ser^{519}
3OE9	the chemokine CXCR4 receptor	Signaling protein, hydrolase	-7.91	1581.106375	Asp^{1010}, Glu^{1011}, Arg^{1014}, Tyr^{1018}, Lys^{1019}, Tyr^{1024}, Thr^{1026}, Gly^{1030}, His^{1031}, Leu^{1032}, Asp^{1070}, Ala^{1074}, Val^{1103}, Phe^{1104}, Gln^{1105}, Met^{1106}, Gly^{1107}, Trp^{1138}, Gln^{1141}, Thr^{1142}, Arg^{1145}
4URW	RAS:SOS complex	Signaling protein	-7.91	1594.505875	Ser^{17}, Ile^{21}, Tyr^{32}, Pro^{34}, Thr^{35}, Ile^{36}, Glu^{37}, Tyr^{40}, Arg^{41}, Asp^{54}, Ile^{55}, Leu^{56}, Asp^{57}, Thr^{58}, Ala^{59}, Gln^{61}
5POQ	BRD1 in complex with N10974A	Gene regulation	-7.9	1610.735375	Ile^{54}, Phe^{55}, Val^{59}, Glu^{63}, Val^{64}, Pro^{65}, Tyr^{67}, Met^{75}, Ile^{102}, Asn^{105}, Cys^{106}, Tyr^{109}, Asn^{110}, Ala^{111}, Thr^{114}, Phe^{116}
4ZMV	Human P-cadherin (SS-X-dimer pocket I)	Cell adhesion	-7.82	1846.706875	Ala^{147}, Tyr^{148}, Ser^{149}, Ile^{150}, His^{151}, Ser^{152}, Gln^{153}, Lys^{156}, Asp^{160}, Leu^{161}, Met^{162}, Phe^{163}, Thr^{164}, Ile^{165}, Arg^{167}, Gln^{192}, Gly^{198}, Thr^{202}
2CZK	Human myo-inositol mono-phosphatase 2 (impa2)	Hydrolase	-7.74	2113.67775	Arg^{146}, Gly^{147}, Gly^{149}, Arg^{156}, Leu^{157}, Arg^{158}, Val^{159}, Ser^{160}, Gly^{161}, Arg^{240}, Glu^{241}, Ala^{242}, Gly^{243}, Gly^{244}, Ile^{245}, Ser^{265}, Thr^{266}, Arg^{267}
3OHM	Activated G alpha Q	Signaling protein	-7.72	2197.34375	Ser^{53}, Ile^{56}, Lys^{57}, Arg^{60}, Gly^{64}, Ser^{65}, Tyr^{67}, Phe^{75}, Leu^{78}, Val^{184}, Pro^{185}, Thr^{186}, Thr^{187}, Ile^{190}, Tyr^{192}, Pro^{193}, Phe^{194}, Asp^{205}
5IOH	Repoman-PP1A (protein phosphatase 1, alpha isoform) holoenzyme	Hydrolase/protein binding	-7.71	2219.709	Arg^{96}, Asn^{124}, His^{125}, Ile^{130}, Tyr^{134}, Trp^{206}, Asp^{208}, Pro^{209}, Asp^{210}, Lys^{211}, Asn^{219}, Asp^{220}, Arg^{221}, Thr^{226}, Gln^{249}, Val^{251}, Glu^{256}, Phe^{258}, Phe^{276}
5L6D	The human METTL3-METTL14 complex	Transferase	-7.69	2315.37475	Tyr^{380}, Leu^{381}, Asp^{382}, Ser^{384}, Ile^{385}, Arg^{564}, Gln^{567}, Arg^{568}, Tyr^{569}, Pro^{570}, Asp^{571}, Ile^{573}, Ile^{574}, Ser^{575}, Lys^{576}
4IA0	The PDE5A1 catalytic domain	Hydrolase	-7.67	2378.7535	Leu^{554}, Lys^{555}, Asp^{558}, Phe^{559}, Ser^{560}, Phe^{561}, Ser^{562}, Glu^{565}, Leu^{566}, Thr^{769}, Lys^{770}, Pro^{771}, Tyr^{772}, Ile^{774}, Pro^{841}, Asp^{844}, Gly^{845}, Lys^{848}, Asn^{849}, Lys^{852}
4GWG	6-phosphogluconate dehydrogenase apo-form	Oxidoreductase	-7.66	2443.86725	Trp^{172}, Arg^{342}, Ala^{345}, Thr^{346}, Gly^{349}, Trp^{350}, Thr^{351}, Leu^{352}, Asn^{353}, Tyr^{354}, Gly^{355}, Gly^{356}, Lys^{376}, Phe^{379}, Asp^{380}, Pro^{383}, Gln^{386}
4IRG	The ETS transcription factor ERG	DNA binding protein	-7.65	2464.5785	Gly^{292}, Gln^{293}, Gln^{295}, Leu^{296}, Trp^{334}, Lys^{338}, Lys^{340}, Met^{343}, Lys^{347}, Leu^{348}, Arg^{350}, Ala^{351}, Tyr^{354}, Tyr^{355}, Lys^{358}
3R4O	Heat shock protein 90	Chaperone	-7.61	2632.2675	Arg^{46}, Glu^{47}, Ser^{50}, Asn^{51}, Asp^{54}, Ala^{55}, Lys^{58}, Asn^{106}, Leu^{107}, Lys^{112}, Gly^{132}, Gln^{133}, Gly^{135}, Val^{136}, Gly^{137}, Phe^{138}, His^{210}, Ser^{211}, Gln^{212}, Phe^{213}, Ile^{214}
1ZXM	Human topo IIA ATPASE/AMP-PNP	Isomerase	-7.58	2773.6605	Gln^{59}, Gln^{60}, Met^{61}, Trp^{62}, Tyr^{72}, Arg^{241}, Lys^{306}, Gly^{307}, Phe^{308}, Gln^{309}, Gln^{310}, Ile^{311}, Ala^{318}, Ser^{320}, Lys^{321}, Gly^{322}, Gly^{323}, Val^{326}
4E+49	Carbonic anhydrase (CA)	Lyase	-7.57	2820.87225	Tyr^{40}, Asp^{41}, Pro^{42}, Leu^{44}, Lys^{45}, Pro^{46}, Leu^{47}, Ser^{48}, Val^{49}, Ser^{50}, Lys^{80}, Gly^{81}, Gly^{82}, Glu^{187}, Ser^{188}, Leu^{189}, Asp^{190}, Tyr^{191}, Ser^{259}
1JT3	Human acidic fibroblast growth factor	Hormone/growth factor	-7.56	2888.32175	Asp^{28}, Gly^{29}, Thr^{30}, Val^{31}, Asp^{32}, Asp^{68}, Thr^{69}, Leu^{72}, Val^{73}, Tyr^{74}, Gly^{75}, Glu^{82}, Ser^{116}, Cys^{117}, Lys^{118}, Arg^{119}
5HKJ	Single chain recombinant globular head of the complement system protein C1Q	Signaling protein	-7.53	3048.61	Asn^{20}, Cys^{63}, Leu^{64}, Ser^{65}, Arg^{76}, Ser^{77}, Leu^{78}, Gly^{79}, Phe^{80}, Glu^{109}, Lys^{110}, Asp^{111}, Pro^{112}, Gln^{152}, Gln^{256}, Gly^{257}, Asp^{259}
4NYH	Pir1 dual specificity phosphatase core	Hydrolase	-7.5	3158.60325	His^{30}, Ile^{31}, Pro^{32}, Glu^{33}, Arg^{34}, Trp^{35}, Lys^{36}, Asp^{37}, Lys^{64}, Leu^{65}, Thr^{96}, Gln^{97}, Tyr^{99}, Thr^{153}, Arg^{158}
3WO2	Human interleukin-18	Immune system	-7.5	3180	Gly^{25}, Asn^{26}, Arg^{27}, Pro^{28}, Glu^{77}, Asn^{78}, Lys^{79}, Ile^{80}, Ile^{81}, Ser^{82}, Phe^{83}, Lys^{135}, Leu^{136}, Ile^{137}, Leu^{138}
2G6P	Truncated (delta 1-89) human methionine aminopeptidase type 1	Hydrolase	-7.49	3234.128	Gln^{113}, Arg^{114}, Pro^{115}, Asp^{116}, Ser^{127}, Glu^{143}, Asp^{144}, Glu^{146}, Gly^{147}, Leu^{150}, Tyr^{233}, Asn^{235}, Gly^{236}, Tyr^{237}, Lys^{307}, Leu^{308}
5H0V	H88A mutated human transthyretin	Transport protein	-7.48	3289.1775	Pro^{11}, Glu^{61}, Phe^{64}, Val^{65}, Glu^{66}, Ala^{97}, Asn^{98}, Asp^{99}, Gly^{101}, Pro^{102}, Arg^{103}, Arg^{104}, Tyr^{105}, Asn^{124}
2IYB	Complex between the 3rd LIM domain of TES and the evh1 domain of MENA	Metal-binding	-7.43	3560.72775	Val^{15}, Tyr^{16}, Asp^{17}, Asp^{18}, Ala^{19}, Val^{24}, Pro^{25}, Ala^{26}, Gly^{27}, Gly^{28}, Val^{59}, Ile^{60}, Asn^{61}, Arg^{84}, Gln^{85}, Val^{86}, Tyr^{87}
5Z90	BRD4 bromodomain 1	Transcription	-7.43	3560.72775	Pro^{45}, Pro^{46}, Pro^{48}, Glu^{49}, Asn^{52}, Lys^{55}, Tyr^{98}, Ile^{101}, Lys^{102}, Thr^{103}, Pro^{104}, Met^{105}, Arg^{113}, Tyr^{118}, Asp^{128}, Thr^{131}

PDB ID	Targets	Classification	Energy (kcal/mol)	Dissoc. constant [nM]	Contacting receptor residues
1Z3U	Angiopoietin-2 receptor binding domain	Signaling protein	-7.4	3751.99325	Arg^{328}, Ser^{332}, Val^{333}, Asp^{334}, Phe^{335}, Gln^{336}, Glu^{352}, Gly^{457}, Met^{458}, Tyr^{459}, Tyr^{460}, Pro^{461}, Gln^{464}, Lys^{468}, Asn^{470}
5A2E	Extracellular SRCR domains of human CD6	Immune system	-7.4	3751.99325	Asp^{291}, Ser^{292}, Trp^{294}, Arg^{314}, Pro^{315}, Lys^{316}, Gly^{317}, Ser^{351}, Gln^{352}, Ser^{353}, Leu^{354}, Ala^{356}, Arg^{357}
4A7U	Human I113T SOD1	Oxidoreductase	-7.39	3841.7065	Gly^{73}, Pro^{74}, Lys^{75}, Asp^{76}, Gly^{85}, Asn^{86}, Val^{87}, Thr^{88}, Asp^{96}, Val^{97}, Ser^{98}, Ile^{99}, Glu^{100}, Leu^{126}, Lys^{128}
5F6D	UBC9 (K48A/K49A/E54A)	Ligase	-7.36	4020.82775	Asp^{19}, His^{20}, Pro^{21}, Phe^{22}, Gly^{23}, Phe^{24}, Val^{25}, Pro^{46}, Gly^{47}, Ala^{48}, Ala^{49}, Ala^{54}, Gly^{55}, Gly^{56}, Leu^{57}, Lys^{110}, Leu^{114}, Gln^{117}, Pro^{157}
4IP9	Human serum amyloid A1	Protein binding	-7.34	4172.9355	Ala^{14}, Arg^{15}, Met^{17}, Trp^{18}, Tyr^{21}, Ile^{58}, Arg^{62}, Ile^{65}, Gln^{66}, Phe^{69}, Gly^{70}, His^{71}
2X1X	Structure of VEGF-C in complex with domains 2 and 3 of VEGFR2 in a tetragonal crystal form	Hormone/signaling protein	-7.29	4571.1365	Trp^{126}, Gln^{130}, Cys^{131}, Met^{132}, Pro^{133}, Cys^{165}, Cys^{166}, Asn^{167}, Ser^{168}, Leu^{171}, Thr^{207}, Ser^{208}, Cys^{209}, Arg^{210}, Cys^{211}, Met^{212}
4MXV	Lymphotoxin alpha	Cytokine/immune system	-7.25	4849.3055	Asn^{41}, Ser^{42}, Leu^{43}, Leu^{44}, Trp^{45}, Asn^{62}, Asn^{63}, Ser^{64}, Tyr^{96}, Gln^{102}, His^{111}, Ser^{148}, Thr^{149}, His^{150}, Thr^{151}, Asp^{152}, Gly^{153}, Ile^{154}
5OKF	Human 14-3-3 sigma with the hspb6 phosphopeptide	Signaling protein	-7.22	5075.406	Trp^{59}, Arg^{60}, Ser^{63}, Ser^{64}, Glu^{66}, Gln^{67}, Arg^{85}, Ala^{132}, Glu^{133}, Val^{134}, Ala^{135}, Lys^{140}, Tyr^{181}, Glu^{182}, Ile^{183}, Arg^{238}
5GNT	BDLP-like folding of Mitofusin 1	Hydrolase	-7.21	5231.9665	Leu^{197}, Ala^{199}, Asp^{200}, Val^{201}, Phe^{202}, Leu^{227}, Ser^{228}, Lys^{229}, Pro^{230}, Asn^{231}, Ser^{331}, Lys^{334}, Thr^{335}, Lys^{336}, Gln^{339}
2QX4	Quinone reductase ii	Oxidoreductase	-7.17	5522.3165	Ala^{152}, Glu^{153}, Tyr^{155}, Thr^{156}, Asp^{163}, Ser^{164}, Arg^{165}, Leu^{168}, Ala^{185}, Pro^{186}, Gln^{187}, Ile^{188}, Phe^{190}, Met^{203}, Ala^{206}, Arg^{210}
2Q7N	LIF receptor (domains 1-5)	CYTOKINE receptor/CYTOKINE	-7.15	5740.907	Thr^{11}, Thr^{12}, Asn^{13}, Asn^{14}, Arg^{16}, Val^{17}, Trp^{18}, Asp^{19}, Pro^{47}, Leu^{48}, Glu^{49}, Thr^{50}, Asn^{52}, Val^{53}, Lys^{54}, Glu^{89}
3ZR0	Human MTH1	Hydrolase	-7.13	5917.996	Arg^{31}, Leu^{108}, Asp^{109}, Ile^{111}, Pro^{112}, Phe^{113}, Lys^{114}, Asp^{115}, Met^{116}, Trp^{117}, Pro^{118}, Ser^{121}, Pro^{125}, Leu^{128}, Gln^{129}, Asp^{143}
5U7Z	Human acid ceramidase (ASAH1, aCDase)	Hydrolase	-7.11	6141.874	Lys^{74}, Val^{77}, Asn^{78}, Lys^{81}, Met^{94}, Gln^{95}, Asp^{98}, Ala^{120}, Ala^{121}, Val^{122}, Asp^{124}, Ile^{125}, Pro^{126}, Leu^{127}, Gly^{128}, Glu^{129}
3H91	Human chromobox homolog 2 (CBX2)	Transcription	-7.08	6428.241	Glu^{9}, Gln^{10}, Val^{11}, Phe^{12}, Arg^{23}, Lys^{26}, Glu^{28}, Trp^{33}, Ser^{42}, Trp^{43}, Glu^{44}, Pro^{45}, Glu^{47}, Asn^{48}, Leu^{50}
3UVU	Flap endonuclease 1 (FEN1)	Protein binding/peptide	-7.04	6900.4685	Val^{215}, Pro^{216}, Asp^{217}, Leu^{218}, Ser^{219}, Val^{257}, Arg^{258}, His^{261}, His^{262}, Asn^{263}, Gln^{297}
1CWF	Human cyclophilin A	Isomerase/immunosuppressant	-7.04	6912.125	Phe^{7}, Leu^{39}, Ser^{40}, Thr^{41}, Gly^{42}, Gly^{45}, Phe^{46}, Gly^{47}, Tyr^{48}, Lys^{49}, Gly^{50}, Phe^{67}, Thr^{68}, His^{70}, Lys^{76}, Asp^{160}, Cys^{161}, Gly^{162}, Gln^{163}
4Y5O	CCM2 HHD in complex with MEKK3 NPB1	Transferase	-7.03	7077.3995	Leu^{299}, Tyr^{302}, Leu^{322}, Tyr^{325}, Arg^{326}, Gly^{328}, Ala^{329}, Ser^{330}, Leu^{353}, Phe^{356}, Ile^{357}, Pro^{358}, Glu^{359}, Lys^{360}, Asp^{361}
5FUG	A human YL1-H2A.Z-H2B complex	DNA binding protein	-6.96	7964.9745	Leu^{68}, Asn^{71}, Ala^{72}, Ser^{73}, Lys^{74}, Asp^{75}, Leu^{76}, Lys^{77}, Val^{78}, Lys^{79}, Arg^{80}, His^{85}, Leu^{88}, Ala^{89}, Gly^{92}
5D1M	UBCH5B	Ligase	-6.91	8680.963	Leu^{13}, Ala^{14}, Pro^{17}, Gln^{20}, Cys^{21}, Ser^{22}, Ala^{23}, Gly^{24}, Gln^{34}, Ala^{35}, Thr^{36}, Met^{38}, Val^{49}, Phe^{51}
5VZ3	Growth factor	Signaling protein	-6.78	10738.092	Asp^{5}, Arg^{16}, Leu^{17}, Gln^{51}, Phe^{52}, Arg^{53}, Ala^{54}, Gln^{60}, Lys^{107}, Asp^{108}, Cys^{109}, His^{110}
6PAX	Human PAX-6 paired domain-DNA complex	Gene regulation	-6.71	12003.441	Pro^{73}, Arg^{74}, Val^{75}, Ala^{76}, Pro^{78}, Val^{81}, Ser^{119}, Arg^{122}, Val^{123}, Asn^{126}
1HF0	DNA-binding domain of OCT-1	Transcription	-6.6	14501.163	Glu^{109}, Asn^{111}, Ile^{112}, Val^{114}, Ala^{115}, Lys^{118}, Ser^{119}, Glu^{122}, Ile^{134}, Gln^{137}, Leu^{138}, Asn^{139}
1QE6	Interleukin-8	Immune system	-6.55	15698.361	Leu^{25}, Val^{27}, Glu^{29}, Ser^{30}, Ala^{35}, Asn^{36}, Thr^{37}, Ile^{39}, Pro^{53}, Lys^{54}, Gln^{59}, Val^{62}
1BL1	PTH receptor N-terminus fragment	Hormone receptor	-6.53	16347.319	Phe^{6}, Leu^{7}, Glu^{10}, Thr^{11}, Arg^{12}, Glu^{13}, Val^{16}, Ile^{23}, Tyr^{24}, Val^{26}, Gly^{27}
5N7E	DBL-homology domain of BCR-ABL	Signaling protein	-6.38	21021.562	Leu^{20}, Tyr^{37}, Phe^{49}, Glu^{50}, Val^{51}, Pro^{52}, Lys^{55}, Thr^{57}, Ala^{58}, Thr^{59}, Ile^{60}, Ser^{61}, Gly^{62}
4X1L	Mutation-induced destabilization of profilin 1 in ALS	Protein binding	-6.35	22001.7	Arg^{75}, Gly^{82}, Glu^{83}, Ser^{85}, Gly^{122}, Leu^{123}, Asn^{125}, Lys^{126}, Tyr^{129}, Glu^{130}
2J8B	Human CD59	Lipid binding protein	-6.26	25697.634	Asn^{5}, Cys^{6}, Pro^{7}, Asn^{8}, Pro^{9}, Tyr^{36}, Asn^{37}, Lys^{38}, Cys^{39}, Arg^{53}, Leu^{54}, Arg^{55}
5M3D	Tuning of CD81LEL (space group p31)	Cell adhesion	-6.26	25871.712	Asp^{128}, Leu^{131}, Gln^{132}, Val^{135}, Val^{136}, Gly^{158}, Ser^{159}, Ser^{160}, Leu^{162}, Thr^{163}, Thr^{166}, Thr^{167}, Leu^{170}, His^{191}
4W9O	The FK1 domain of FKBP51	Isomerase	-6.23	27123.732	Ile^{25}, Ser^{27}, Thr^{46}, Pro^{47}, Met^{48}, Ile^{49}, Leu^{81}, Asp^{91}, Ile^{92}, Ala^{95}, Thr^{96}
3I69	Apo Glutathione transferase A1-1 GIMF-helix	Transferase	-6.2	28774.302	Met^{94}, Tyr^{95}, Gly^{98}, Asp^{101}, Asn^{130}, Arg^{131}, Tyr^{132}, Pro^{134}, Ala^{135}, Phe^{136}, Lys^{138}, Val^{139}
3KLT	A vimentin fragment	Structural protein	-5.9	47182.032	Ala^{312}, Glu^{315}, Ser^{316}, Tyr^{319}, Arg^{320}, Val^{323}, Gln^{324}
